# Long‐term use of probiotics for the management of office and ambulatory blood pressure: A systematic review and meta‐analysis of randomized, controlled trials

**DOI:** 10.1002/fsn3.3069

**Published:** 2022-09-20

**Authors:** Tian‐Xue Zhao, Li Zhang, Ning Zhou, Dong‐Sheng Sun, Jian‐Hong Xie, Shao‐Kun Xu

**Affiliations:** ^1^ Department of Endocrinology Affiliated Hangzhou First People's Hospital Zhejiang University School of Medicine Hangzhou China; ^2^ Geriatric Medicine Center Department of Geriatric Medicine Zhejiang Provincial People's Hospital (Affiliated People's Hospital, Hangzhou Medical College) Hangzhou Zhejiang China

**Keywords:** blood pressure, meta‐analyses, probiotics, randomized controlled trials

## Abstract

Previous studies showed a controversial result on the relationship between probiotics treatment duration and blood pressure (BP). The present meta‐analysis is performed to summarize the effects of long‐term (≥8 weeks) use of probiotics on office and ambulatory BP using combined evidence from randomized, controlled trials. We searched PubMed, Embase, Cochrane library, and the ClinicalTrials.gov till January, 2021 to identify eligible articles. Primary outcomes were changes in office BP. In the presence of heterogeneity, a random‐effects model was used to calculate the combined treatment effect. Begg's funnel plots and Egger's regression test were used to assess the publication bias. Meta‐analysis of 26 trials in 1624 participants demonstrated that probiotic consumption significantly decreased office systolic BP by 2.18 mmHg (95% confidence interval [CI], −3.41 to −0.94 mmHg) and diastolic BP by 1.07 mmHg (95% CI, −1.72 to −0.41 mmHg). The analysis on ambulatory BP from three trials showed a similar reduction by −2.35/−1.61 mmHg (*p* ≤ .052). Subgroup analysis in hypertensive and diabetic patients showed a significant reduction in systolic and diastolic BP (*p* ≤ .02). The reductions in diabetic and hypertensive patients were comparatively larger than nondiabetic and normotensive patients (*p* ≥ .052). With the increase of age, baseline body mass index (BMI), treatment duration, and systolic BP, the effects of probiotics on BP did not increase significantly (*p*_trend_ ≥ .18). The present meta‐analysis suggests a beneficial effect of probiotics on BP by a modest degree, especially in the diabetes mellitus and hypertension. Prolonging the treatment duration could not improve the antihypertensive effect.

## Graphical abstract

Long‐term probiotics' consumption (≥8 weeks) had a beneficial effect on office and ambulatory blood pressure (BP), especially in the diabetes and hypertension, and the changes in BP were not associated with the increase of age, body mass index (BMI), and treatment duration.

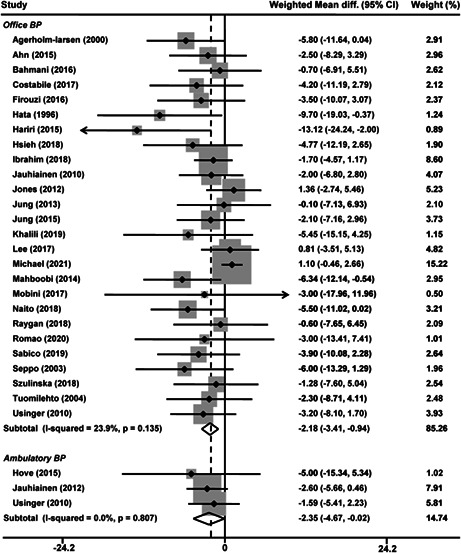

## 1 INTRODUCTION

Hypertension is one of the major risk factors for chronic diseases, including metabolic disorders, neurological diseases, and cardiovascular diseases (Forouzanfar et al., 2017; Kearney et al., 2005). To date, hypertension is largely considered as the result of the interaction of environmental factors and genetic factors (Ezzati & Riboli, 2013; Hu et al., 2004). In the last decades, studies on hypertension mainly focused on the role of diet and lifestyle modification (Appel et al., 1997; Aucott et al., 2005), inflammatory factors (Dinh et al., 2014), peripheral vascular remodeling (Lee, Dickhout, & Sandow, 2017), and sympathetic nervous system regulation (Oliveira‐Sales et al., 2016). While other factors of hypertension, especially the resident microbes in the human gastrointestinal tract, are not fully understood. Gut microbiota have been described recently to influence the host physiology. Animal and human studies have shown that the imbalance in the abundance, diversity, and evenness of certain microbiota species is associated with hypertension (Li et al., 2017; Marques et al., 2017; Mell et al., 2015; Yang, Santisteban, et al., 2015). Thus, there has been a growing interest in using probiotics to correct gut microbiota disturbances (Durgan et al., 2016) and to control blood pressure (BP) (Khalesi et al., 2014). Previous studies on probiotics have confirmed that daily supplementation with probiotics improved office systolic/diastolic BP by 1.79 to 3.56/1.26 to 2.38 mmHg (Chi et al., 2020; Dong et al., 2013; Khalesi et al., 2014; Liang et al., 2021; Qi et al., 2020). However, analysis based on the duration of intervention showed a controversial result among studies. Khalesi et al. (2014) and Chi et al. (2020) found that longer treatment duration (>8 weeks) resulted in a more prominent reduction in BP. Recent randomized, controlled trials have also suggested a consistent BP modulation capability with a duration more than 21 weeks (Hsieh et al., 2018; Seppo et al., 2003). However, Liang et al. (2021) and Qi et al. (2020) concluded in their analysis that the beneficial effect of probiotics' supplementation on BP could only last for a short‐term time of 8 or 10 weeks, but when the treatment duration lasted for more than 10 weeks, the reduction was not significant. In view of the notion that the duration of probiotic intervention might affect the effect of BP improvement, we conducted this meta‐analysis to investigate the effect of long‐term probiotic consumption (≥8 weeks) on BP.

## 2 METHODS

### 2.1 Literature search

Our meta‐analysis strictly followed the Prefered Reporting Items for Systematic Reviews and Meta‐Analysis (PRISMA) guidelines (Page et al., 2021). We searched through PubMed, Embase, Cochrane Library, and ClinicalTrials.gov databases until January, 2021 for relevant studies, using the terms: *microbiota*, *gut microbiota*, *probiotics*, *fermented milk*, *Kefir*, *Lactobacillus*, *Streptococcus*, *bifidobacter*, *blood pressure and hypertension*, *normotension*, and *metabolism syndrome* (Table S1). Moreover, we manually scanned the reference lists in the identified articles. The protocol of this systematic review was registered on PROSPERO (The International Prospective Register of Systematic Reviews) with the registration number CRD42022312574.

Eligible studies were included if they met the inclusion criteria: accessible full articles in English; a randomized, controlled trial; included adults more than 18 years of age; used probiotic products with live bacteria in the intervention group, and used placebo products in the control group; had an intervention duration of not less than 8 weeks; absence of pregnancy or breast‐feeding. We excluded studies that mentioned receiving probiotic‐rich diet or consuming probiotics with other functional ingredients, such as inulin, vitamins, and fermentable fibers. Studies that used other study designs or duplicate publications were also excluded.

The two reviewers (Tian‐Xue Zhao and Li Zhang) independently completed the initial screening of the eligible articles based on the titles. By analyzing the abstracts and full text, the studies were selected through agreement; otherwise, negotiated with a third investigator (Dong‐Sheng Sun or Shao‐Kun Xu) to resolve the disagreement.

### 2.2 Data extraction and quality assessment

Tian‐Xue Zhao and Li Zhang used a standard form for data extraction and analysis. For studies not reporting standard deviation (SD), we calculated SD from sample size and standard error. If means and SDs were unavailable, we contact the author to obtain the related data.

Assessment of risk bias was conducted by Ning Zhou based on the Cochrane Risk of Bias tool, which included: random sequence, allocation concealment, blinding of subjects and researchers, incomplete outcome data, selecting reporting of outcomes, and other bias.

### 2.3 Data analysis

The Stata software (Version 14.0, StataCorp LP, College Station, TX, USA) was used for data management and statistical analysis. The net effect of probiotics on BP was defined as the weight mean difference of changes between the probiotic and control groups. We used Cochran's *Q* test and *I* ^2^ statistic to test the between‐study heterogeneity. In the presence of heterogeneity between studies' population and BP analysis, a random‐effects model was conducted; otherwise, a fixed‐effects model was used. A *p* value <.05 was considered statistically significant. The Begg's funnel plots and Egger's regression tests were used to assess the potential publication bias at the *p* < .10 level of significance. In case of heterogeneity, subgroup analyses, sensitivity analyses, and meta‐regression were further inspected. The studies were divided into three tertiles for further statistical analysis according to their initial age, treatment duration, baseline body mass index (BMI), and systolic BP, respectively. We then performed tests for linear trend by entering the median value of each quartile as a continuous variable in the models.

## 3 RESULTS

### 3.1 Characteristics of included studies

**FIGURE 1:**
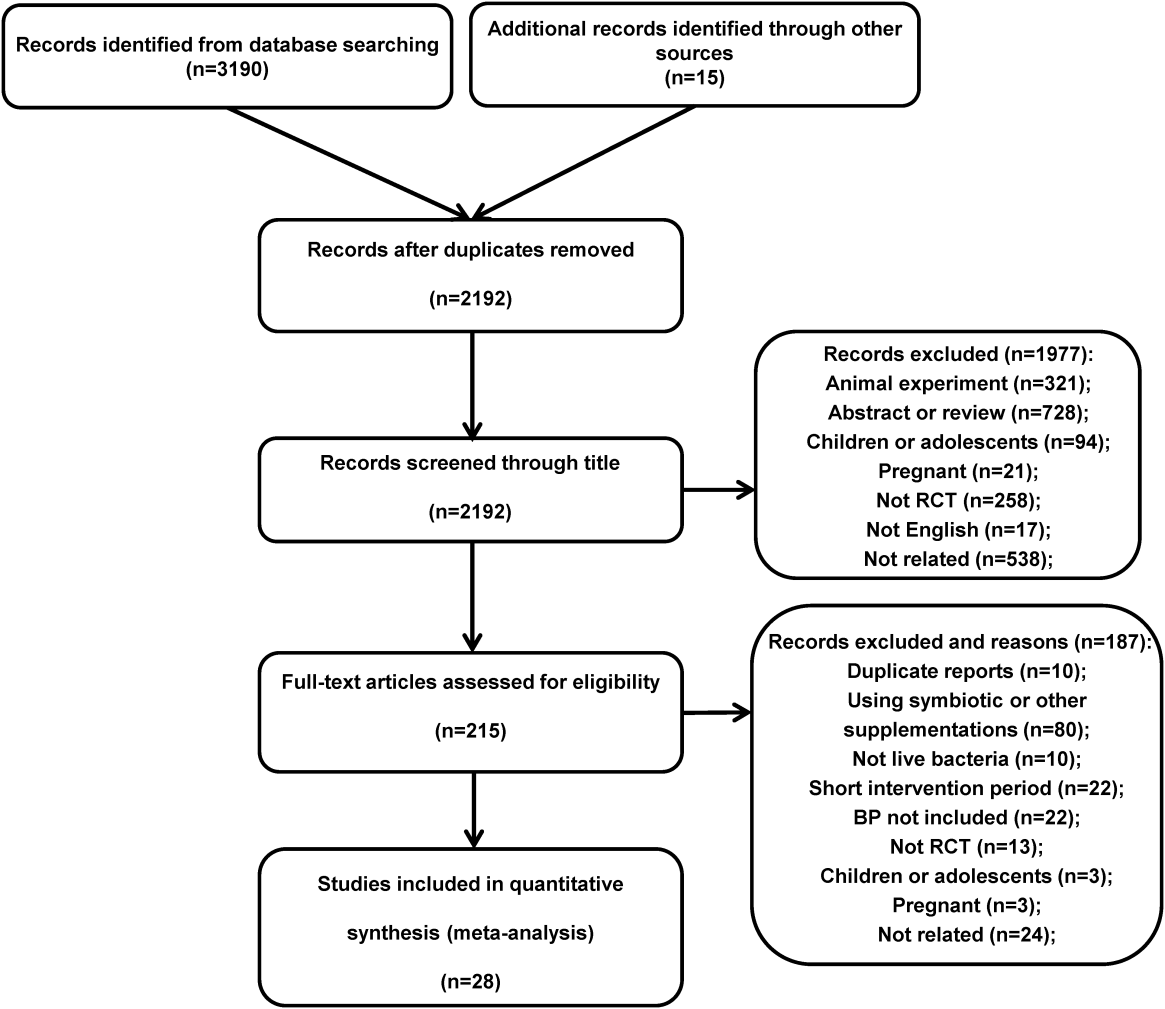
Flow diagram of the study selection. BP, blood pressure; RCT, randomized, controlled trial

Figure 1 presents the flow diagram of the literature search. 3205 related records were preliminarily retrieved, and 2192 records remained after 1013 duplicate records were removed. 321 animal studies, 728 meeting abstracts, case reports, reviews, and letters, 258 not randomized control studies, 94 included children or adolescents, 21 included pregnant women, 17 not in English, and 538 irrelevant articles were removed from the 2192 records after reviewing the titles. Of the 215 full‐text retrieved articles, 10 were duplicate reports, 80 had a co‐intervention of other supplementations or using symbiotic, 10 used inactivated bacteria, 22 had short intervention duration, 22 did not include BP in outcomes, 13 were not randomized control studies, 3 included children or adolescents, 3 included pregnant women, and 24 were not related, leaving 28 eligible original articles in the analysis (full reference lists of excluded studies are presented in Table S2).

The characteristics of 28 included studies are presented in Table 1. The articles were published between 1996 and 2021. Of the 28 included randomized, controlled studies, 26 studies reported a double‐blind design (Agerholm‐Larsen et al., 2000; Ahn et al., 2015; Bahmani et al., 2016; Costabile et al., 2017; Firouzi et al., 2017; Hariri et al., 2015; Hove et al., 2015; Hsieh et al., 2018; Ibrahim et al., 2018; Jauhiainen et al., 2010; Jauhiainen et al., 2012; Jones et al., 2012; Jung et al., 2013; Jung et al., 2015; Khalili et al., 2019; Lee, Lee, et al., 2017; Mahboobi et al., 2014; Michael et al., 2021; Mobini et al., 2017; Naito et al., 2018; Raygan et al., 2018; Sabico et al., 2019; Seppo et al., 2003; Szulinska et al., 2018; Tuomilehto et al., 2004; Usinger et al., 2010), 1 reported cross‐over design (Tuomilehto et al., 2004), 22 studies reported a specific daily dose of the supplement (Agerholm‐Larsen et al., 2000; Ahn et al., 2015; Bahmani et al., 2016; Costabile et al., 2017; Firouzi et al., 2017; Hariri et al., 2015; Hata et al., 1996; Hsieh et al., 2018; Ibrahim et al., 2018; Jones et al., 2012; Jung et al., 2013; Jung et al., 2015; Khalili et al., 2019; Lee, Lee, et al., 2017; Mahboobi et al., 2014; Michael et al., 2021; Mobini et al., 2017; Naito et al., 2018; Raygan et al., 2018; Romao et al., 2020; Sabico et al., 2019; Szulinska et al., 2018), and 23 studies were conducted in all genders (Agerholm‐Larsen et al., 2000; Ahn et al., 2015; Bahmani et al., 2016; Costabile et al., 2017; Firouzi et al., 2017; Hariri et al., 2015; Hata et al., 1996; Hsieh et al., 2018; Jauhiainen et al., 2010; Jauhiainen et al., 2012; Jones et al., 2012; Jung et al., 2013; Jung et al., 2015; Khalili et al., 2019; Lee, Lee, et al., 2017; Mahboobi et al., 2014; Michael et al., 2021; Mobini et al., 2017; Raygan et al., 2018; Sabico et al., 2019; Seppo et al., 2003; Tuomilehto et al., 2004; Usinger et al., 2010). Among these studies, 5 were conducted in Iran (Bahmani et al., 2016; Hariri et al., 2015; Khalili et al., 2019; Mahboobi et al., 2014; Raygan et al., 2018), 4 in Finland (Jauhiainen et al., 2010; Jauhiainen et al., 2012; Seppo et al., 2003; Tuomilehto et al., 2004) and Korea (Ahn et al., 2015; Jung et al., 2013; Jung et al., 2015; Lee, Lee, et al., 2017), 3 in Denmark (Agerholm‐Larsen et al., 2000; Hove et al., 2015; Usinger et al., 2010), 2 in Japan (Hata et al., 1996; Naito et al., 2018) and Malaysia (Firouzi et al., 2017; Ibrahim et al., 2018), and one each in Brazil (Romao et al., 2020), Bulgaria (Michael et al., 2021), Czech (Jones et al., 2012), Poland (Szulinska et al., 2018), Saudi Arabia (Sabico et al., 2019), Sweden (Mobini et al., 2017), United Kingdom (Costabile et al., 2017), and Taiwan district (Hsieh et al., 2018). Of the 28 studies, 8 included patients with diabetes mellitus (Bahmani et al., 2016; Firouzi et al., 2017; Hariri et al., 2015; Hove et al., 2015; Hsieh et al., 2018; Khalili et al., 2019; Mobini et al., 2017; Sabico et al., 2019), 6 included patients with hypertension (Hata et al., 1996; Jauhiainen et al., 2010; Jauhiainen et al., 2012; Seppo et al., 2003; Tuomilehto et al., 2004; Usinger et al., 2010), 7 included overweight and obese subjects (Agerholm‐Larsen et al., 2000; Jung et al., 2013; Jung et al., 2015; Michael et al., 2021; Mobini et al., 2017; Naito et al., 2018; Szulinska et al., 2018), and 4 included patients with hyperlipidemia (Ahn et al., 2015; Costabile et al., 2017; Firouzi et al., 2017; Jones et al., 2012). And among the 28 studies, 13 studies used single species of probiotics as intervention treatment (Costabile et al., 2017; Hariri et al., 2015; Hove et al., 2015; Hsieh et al., 2018; Jauhiainen et al., 2010; Jauhiainen et al., 2012; Jones et al., 2012; Jung et al., 2013; Khalili et al., 2019; Naito et al., 2018; Seppo et al., 2003; Tuomilehto et al., 2004; Usinger et al., 2010), while other 12 studies used more than 1 species of probiotics (Agerholm‐Larsen et al., 2000; Ahn et al., 2015; Firouzi et al., 2017; Hata et al., 1996; Jung et al., 2015; Lee, Lee, et al., 2017; Mahboobi et al., 2014; Michael et al., 2021; Raygan et al., 2018; Romao et al., 2020; Sabico et al., 2019; Szulinska et al., 2018). The source of probiotics varied between studies, 10 studies used fermented milk or yoghurt (Agerholm‐Larsen et al., 2000; Hata et al., 1996; Hove et al., 2015; Jauhiainen et al., 2010; Jauhiainen et al., 2012; Lee, Lee, et al., 2017; Naito et al., 2018; Seppo et al., 2003; Tuomilehto et al., 2004; Usinger et al., 2010), 8 studies used encapsulated supplement (Costabile et al., 2017; Hsieh et al., 2018; Jones et al., 2012; Jung et al., 2013; Khalili et al., 2019; Mahboobi et al., 2014; Michael et al., 2021; Raygan et al., 2018), 8 studies used probiotic powder (Ahn et al., 2015; Firouzi et al., 2017; Ibrahim et al., 2018; Jung et al., 2015; Mobini et al., 2017; Romao et al., 2020; Sabico et al., 2019; Szulinska et al., 2018), 1 used soy milk (Hariri et al., 2015), and the other 1 used probiotic bread (Bahmani et al., 2016). In addition, 25 studies only reported changes in office systolic and diastolic BP (Agerholm‐Larsen et al., 2000; Ahn et al., 2015; Bahmani et al., 2016; Costabile et al., 2017; Firouzi et al., 2017; Hariri et al., 2015; Hata et al., 1996; Hsieh et al., 2018; Ibrahim et al., 2018; Jauhiainen et al., 2010; Jones et al., 2012; Jung et al., 2013; Jung et al., 2015; Khalili et al., 2019; Lee, Lee, et al., 2017; Mahboobi et al., 2014; Michael et al., 2021; Mobini et al., 2017; Naito et al., 2018; Raygan et al., 2018; Romao et al., 2020; Sabico et al., 2019; Seppo et al., 2003; Szulinska et al., 2018; Tuomilehto et al., 2004), 1 study by Usinger et al. reported both office and ambulatory BP changes (Usinger et al., 2010), and 2 studies by Hove et al. and Jauhiainen et al. only reported changes in ambulatory BP (Hove et al., 2015; Jauhiainen et al., 2012). Mean age of these studies changed from 37.8 to 76.5 years and the duration of intervention ranged from 8 to 37 weeks, with a median of 12 weeks.

**TABLE 1:** Characteristics of the included studies

Study	Duration (weeks)	Population	Location	Age	No. of probiotics/control (No. of users of antihypertensive medication)	Probiotic type	Changes from baseline SBP/DBP (mmHg)	
							Probiotic	Control
Office BP								
Agerholm‐Larsen et al. (2000)	8	Overweight	Denmark	37.8	16/14	*Streptococcus thermophilus*, *Enterococcus faecium*	−8.00/−4.00	−2.20/−1.50
Ahn et al. (2015)	12	Hypertriglyceridemic	Korea	54.1	46/46	*L. plantarum*, *L. curvatus*	−1.60/−0.30	0.90/1.50
Bahmani et al. (2016)	8	DM	Iran	52.0	25/26	*L. sporogenes*	−6.40/−3.80	−5.70/−5.20
Costabile et al. (2017)	12	Hypercholesterolemic	United Kingdom	52.3	23/23	*L. plantarum*	0.50/0.00	4.70/2.40
Firouzi et al. (2017)	12	DM, Hyperlipidemia	Malaysia	52.9	48/53	*L. acidophilus*, *L. casei*, *L. lactis*, *B. bifidum*, *B. longum*, *B. infantis*	−8.10/−2.90	−4.60/−0.40
Hariri et al. (2015)	8	DM	Iran	56.9	20/20	*L. planetarium* A7	−12.37/−6.75	0.75/−1.50
Hata et al. (1996)	8	Hypertension	Japan	76.5	17/13 (16/10)	*L. helveticus*, *Saccharomyces cerevisiae*	−14.10/−6.60	−4.40/−2.20
Hsieh et al. (2018)	24	DM	Taiwan	52.3	22/22	*L. reuteri*	−2.82/−0.91	1.95/0.36
Ibrahim et al. (2018)	12	Male	Malaysia	23.0	10/10	*L. acidophilus*, *L. casei*, *L. lactis*, *B. bifidum*, *B. infantis*, *B. longum*	0.70/2.40	2.40/1.40
Jauhiainen et al. (2010)	24	Hypertension	Finland	49.0	45/44	*L. helveticus*	−4.60/−3.70	−2.60/−1.70
Jones et al. (2012)	9	Hypercholesterolemic	Czech	50.6	62/62	*L. reuteri*	0.18/−1.46	−1.18/−0.16
Jung et al. (2013)	12	Overweight, Obese,	Korea	/	28/29	*L. gasseri*	1.20/0.60	1.30/0.40
Jung et al. (2015)	12	Overweight	Korea	40.1	49/46	*L. plantarum*, *L. curvatus*	−1.90/−2.40	0.20/0.70
Khalili et al. (2019)	8	DM	Iran	44.0	20/20	*L. casei*	−4.95/−3.00	0.50/1.50
Lee, Lee, et al. (2017)	12	Age > 60, Nondiabetic	Korea	65.7	73/79	*L. paracasei*, *B. lactis*, heat‐treated *L. plantarum*	−1.42/−1.41	−2.23/−0.20
Mahboobi et al. (2014)	8	Prediabetes	Iran	51.0	28/27	*L. casei*, *L. acidophilus*, *L. rhamnosus*, *L. bulgaricus*, *B. breve*, *B. longum*, *Streptococcus thermophilus*	−3.10/−0.33	3.24/2.77
Michael et al. (2021)	37	Overweight	Bulgaria	52.4	35/35	*L. acidophilus*, *L. plantarum*, *B. bifidum*, *B. animalis* subsp. *lactis*	1.02/−0.74	−0.08/−0.66
Mobini et al. (2017)	12	DM, Obesity	Sweden	64.0	14/15	*L. reuteri*	−3.00/−2.00	0.00/−1.00
Naito et al. (2018)	8	Obese Prediabetic	Japan	46.6	48/50	*L. casei*	−3.70/−2.60	1.80/1.60
Raygan et al. (2018)	12	DM, CHD	Iran	60.7	30/30	*B. bifidum*, *L. casei*, *L. acidophilus*	−1.90/−1.70	−1.30/−1.10
Romao et al. (2020)	8	Hypertension	Brazil	43.3	19/17 (17/16)	*L. casei*, *L. rhamnosus*, *L. acidophilus*, *B. lactis*	−5.00/−2.00	−2.00/2.00
Sabico et al. (2019)	24	DM	Saudi Arabia	48.0	31/30	*B. bifidum*, *B. lactis*, *L. acidophilus*, *L. brevis*, *L. casei*, *L. salivarius*, *L. lactis*	−4.20/−2.60	−0.30/−1.30
Seppo et al. (2003)	21	Hypertension	Finland	50.9	22/17 (9/7)	*L. helveticus*	−15.40/−9.30	−9.40/−5.50
Szulinska et al. (2018)	12	Obese postmenopausal women	Poland	55.2	23/24	*B. bifidum*, *B. lactis*, *L. acidophilus*, *L. brevis*, *L. casei*, *L. salivarius*, *L. lactis*	−3.40/−0.52	−2.12/−1.79
Tuomilehto et al. (2004)	10	Mild hypertension	Finland	51.3	30/29	*L. helveticus*	−15.80/−10.30	−13.50/−9.80
Usinger et al. (2010)	8	Prehypertensive and borderline hypertensive subjects	Denmark	54.0	29/30	*L. helveticus*	−7.00/−3.40	−3.80/−1.70
Ambulatory BP								
Hove et al. (2015)	12	DM	Denmark	58.5	23/18	*L. helveticus*	−3.00/−2.00	2.00/1.00
Jauhiainen et al. (2012)	12	Hypertension	Finland	49.0	45/44	*L. helveticus*	−2.30/−1.80	0.30/−0.50
Usinger et al. (2010)	8	Prehypertensive and borderline hypertensive subjects	Denmark	54.0	29/30	*L. helveticus*	−2.7/−1.8	−1.1/−0.7

Abbreviations: BP, blood pressure; CHD, coronary heart disease; DM, diabetes mellitus; DBP, diastolic blood pressure; SBP, systolic blood pressure.

### 3.2 Risk of bias

According to the Cochrane Risk of Bias tool, all included studies had a fair to good study quality (Figure S1).

### 3.3 Main outcome

**FIGURE 2:**
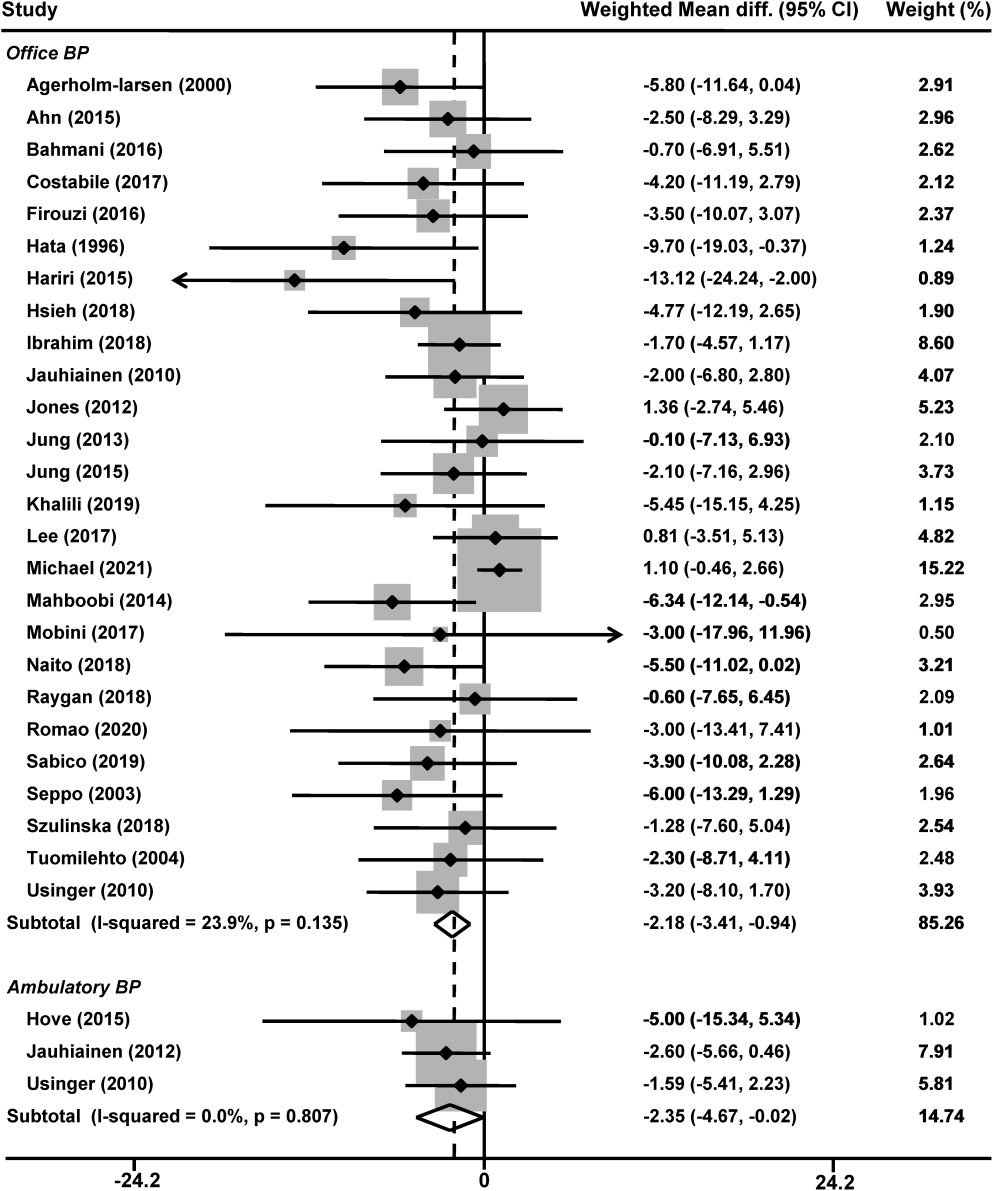
Effects of probiotics on office and ambulatory systolic blood pressure. CI, confidence interval

**FIGURE 3:**
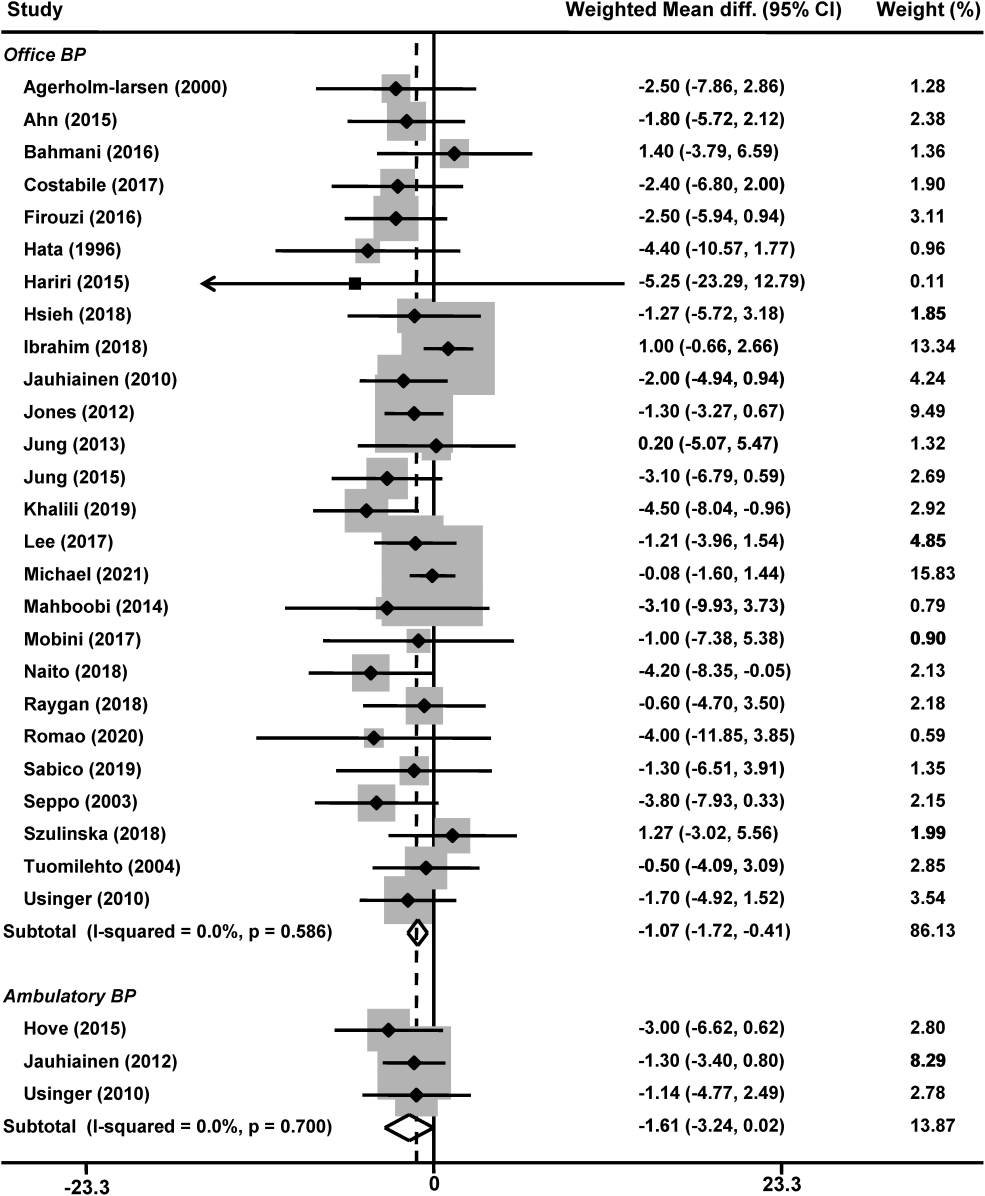
Effects of probiotics on office and ambulatory diastolic blood pressure. CI, confidence interval

Twenty‐six studies reported office BP changes (Agerholm‐Larsen et al., 2000; Ahn et al., 2015; Bahmani et al., 2016; Costabile et al., 2017; Firouzi et al., 2017; Hariri et al., 2015; Hata et al., 1996; Hsieh et al., 2018; Ibrahim et al., 2018; Jauhiainen et al., 2010; Jones et al., 2012; Jung et al., 2013; Jung et al., 2015; Khalili et al., 2019; Lee, Lee, et al., 2017; Mahboobi et al., 2014; Michael et al., 2021; Mobini et al., 2017; Naito et al., 2018; Raygan et al., 2018; Romao et al., 2020; Sabico et al., 2019; Seppo et al., 2003; Szulinska et al., 2018; Tuomilehto et al., 2004; Usinger et al., 2010), with 21 reporting a reduction in systolic (Agerholm‐Larsen et al., 2000; Ahn et al., 2015; Bahmani et al., 2016; Firouzi et al., 2017; Hariri et al., 2015; Hata et al., 1996; Hsieh et al., 2018; Jauhiainen et al., 2010; Jung et al., 2015; Khalili et al., 2019; Lee, Lee, et al., 2017; Mahboobi et al., 2014; Mobini et al., 2017; Naito et al., 2018; Raygan et al., 2018; Romao et al., 2020; Sabico et al., 2019; Seppo et al., 2003; Szulinska et al., 2018; Tuomilehto et al., 2004; Usinger et al., 2010) and 23 in diastolic (Agerholm‐Larsen et al., 2000; Ahn et al., 2015; Bahmani et al., 2016; Firouzi et al., 2017; Hariri et al., 2015; Hata et al., 1996; Hsieh et al., 2018; Jauhiainen et al., 2010; Jones et al., 2012; Jung et al., 2015; Khalili et al., 2019; Lee, Lee, et al., 2017; Mahboobi et al., 2014; Michael et al., 2021; Mobini et al., 2017; Naito et al., 2018; Raygan et al., 2018; Romao et al., 2020; Sabico et al., 2019; Seppo et al., 2003; Szulinska et al., 2018; Tuomilehto et al., 2004; Usinger et al., 2010) BP after probiotic intervention, ranging from −1.42 to −15.80/−0.30 to −10.30 mmHg. The meta‐analysis of the 26 studies showed that probiotic supplementation significantly decreased office systolic BP by −2.18 mmHg (95% confidence interval [CI], −3.41 to −0.94 mmHg, *p* = .0005, Figure 2) compared with the control group. No statistical heterogeneity was observed between the studies (*I* ^2^ = 23.9%, *p* = .14). The overall changes of office diastolic BP between probiotic and control groups were − 1.07 mmHg (95% CI, −1.72 to −0.41 mmHg, *p* = .001, Figure 3), without an indication of heterogeneity (*I* ^2^ = 0, *p* = .59). Ambulatory BP was reported in 3 studies (Hove et al., 2015; Jauhiainen et al., 2012; Usinger et al., 2010). In a fixed‐effects model (*I* ^2^ = 0%, *p* = .81), the weight mean difference in systolic BP of probiotic group was −2.35 mmHg (*p* = .048, Figure 2) compared with control group. While, the probiotic group did not significantly influence ambulatory diastolic BP (−1.61 mmHg, *p* = .052; *I* ^2^ = 0%, *p* = .70, Figure 3).

In addition, with the increase of age, baseline BMI, treatment duration, and baseline office systolic BP, the effects of probiotics on BP did not increase significantly (*p*_trend_ ≥ .18).

### 3.4 Subgroup and sensitivity analysis

We performed subgroup analysis on office BP according to diabetic (*n* = 8) and hypertensive (*n* = 6) status. Office BP was significantly reduced by −3.49/−1.88 mmHg in diabetic patients (*p* ≤ .02, *I* ^2^ = 0%, *p* ≥ .71; Table 2); and in nondiabetic patients (*n* = 11), the reductions were significant with an absolute value up to 1.83/1.65 mmHg (*p* ≤ .02; *I* ^2^ = 0%, *p* ≥ .60; Table 2). Although the reduction of office BP was relatively larger in diabetic patients, the difference in changes did not reach a statistical significance between diabetic and nondiabetic patients (*p* ≥ .30). Subgroup analysis with hypertensive patients (*n* = 6) showed a significant reduction in office systolic/diastolic BP by −3.55/−2.14 mmHg (*p* ≤ .009; *I* ^2^ = 0%, *p* ≥ .75; Table 2), which was comparatively greater than in normotensive patients (*n* = 12, −1.78/−1.07 mmHg, *p* ≤ .07; Table 2). Similarly, the differences in office BP were not significant between hypertensive and normotensive patients (*p* ≥ .052). Further analysis showed that by using the probiotics, the BP of hypertension patients who used antihypertensive drugs decreased by 6.38/3.99 mmHg (*p* = .01), while the BP of untreated hypertension patients decreased by 2.52/1.50 mmHg (*p* ≥ .10). No significant difference was observed between the two groups (*p* ≥ .18). When limiting analysis to studies reported a BMI reduction after probiotic supplementation, pooled results showed no meaningful improvement in both systolic and diastolic BP (−1.41/−1.01 mmHg, *p* ≥ .06). Similar results were also observed in patients with a stable and increased BMI after consuming probiotics (*p* ≤ .45). Subgroup analysis based on sex showed that no significant changes in systolic or diastolic BP were observed among the studies (*p* ≥ .10, Table 2).

**TABLE 2:** Results of subgroup analysis of included trials

Subgroup analysis	Trials (*n*)	Systolic blood pressure				Diastolic blood pressure			
		Change	*p* ^a^	*I* ^2^	*p* ^b^	Change	*p* ^a^	*I* ^2^	*p* ^b^
Diabetes mellitus									
Yes	8	−3.49 (−6.20, −0.79)	.01	0	.71	−1.88 (−3.51, −0.25)	.02	0	.73
No	11	−1.83 (−3.42, −0.24)	.02	0	.60	−1.65 (−2.67, −0.64)	.001	0	.91
Hypertension									
Yes	6	−3.55 (−6.14, −0.96)	.007	0	.75	−2.14 (−3.74, −0.54	.009	0	.82
No	12	−1.78 (−3.44, −0.12)	.04	28.0	.17	−1.07 (−2.23, 0.09)	.07	30.0	.15
Sex									
Male	2	−2.84 (−6.25, 0.57)	.10	30.2	.23	−1.24 (−6.28, 3.81)	.63	80.8	.02
Female	2	−1.74 (−7.15, 3.66)	.53	0	.78	−0.30 (−5.02, 4.42)	.90	24.9	.25
Body mass index									
Decrease	13	−1.41 (−3.17, 0.36)	.12	30.1	.14	−1.01 (−2.07, 0.04)	.06	23.4	.21
Increase	4	−3.23 (−6.73, 0.26)	.07	37.1	.19	−2.22 (−4.12, −0.32)	.02	0	.70
Stable	3	−3.12 (−7.05, 0.82)	.12	0	.69	−1.01 (−3.62, 1.60)	.45	0	.97
Source of probiotics									
Dairy products	8	−3.22 (−5.28, −1.16)	.002	6.5	.38	−2.11 (−3.40, −0.82)	.001	0	.84
Powder	16	−0.93 (−2.14, 0.28)	.13	5.8	.39	−0.77 (−1.54, 0.01)	.054	1.3	.44
Species of probiotics									
Single	13	−2.61 (−4.36, −0.87)	.003	0	.55	−1.86 (−2.89, −0.84)	.0004	0	.20
Two or more	12	−2.50 (−4.04, −0.95)	.002	0	.67	−0.72 (−1.74, 0.29)	.16	0	.45

^a^ The *p* value is given for the comparison between the probiotic and control groups.

^b^ The *p* value is given for the heterogeneity between studies.

Trials using single species of probiotics showed a significant reduction by −2.61/−1.86 mmHg (*n* = 13, *p* ≤ .003, Table 2), compared with control group. Pooled analysis of studies with more than 1 species of probiotics (*n* = 12) showed a significant reduction in systolic BP by 2.50 mmHg (*p* = .002), but not diastolic BP (*p* = .16). Trials using dairy products as the source of probiotics (*n* = 8) showed a significant reduction in BP by 3.22/2.11 mmHg (*p* ≤ .002), with no presence of heterogeneity (*I* ^2^ ≤ 6.5%, *p* ≥ .38), while using probiotic powder (*n* = 16) did not show a marked reduction in both systolic and diastolic BP (−0.93/−0.77 mmHg, *p* ≥ .054).

### 3.5 Publication bias

Publication bias across 26 studies with office BP was observed for the overall meta‐analysis of systolic BP changes (Begg's test, *p* = .005 and Egger's test, *p* = .001), we further performed trim‐and‐fill method to recalculate the mean changes and the result remained alternative (*p* < .0001). When trials with extreme results were excluded (Jones et al., 2012; Lee, Lee, et al., 2017; Michael et al., 2021), the pooled effect from systolic BP slightly changed to −3.20 mmHg (95% CI, −4.46 to −1.94 mmHg, *p* < .0001). No significant publication bias was observed for diastolic BP.

## 4 DISCUSSION

The present meta‐analysis examined the effect of long‐term consumption of probiotics on office and ambulatory BP. The results showed that probiotic supplementation could significantly reduce office systolic and diastolic BP by 2.18/1.07 mmHg. The corresponding changes in ambulatory BP were of similar size, while only systolic BP had reached statistical significance. The effect was particularly prominent in hypertensive and diabetic patients. Treatment duration, age, BMI, sex, and systolic BP level might not affect the BP‐lowering effect.

There is increasing evidence from both experimental and clinical studies that different kinds of factors, such as diet and obesity, are related to hypertension by affecting gut microbiota (Hao et al., 2016; Linz et al., 2012; Petriz et al., 2014). The balance, richness, and diversity of gut microbiota were significantly changed in hypertensive patients compared with healthy controls (Li et al., 2017; Marques et al., 2017; Yang, Santisteban, et al., 2015). Studies have shown that a high intake of fruits and vegetables is significantly linked with a lower risk of cardiovascular mortality and a lower BP level (Alonso et al., 2004; Miura et al., 2004), which is currently believed to be partly related to the changing species and the function of gut microbiota (Marques et al., 2017). To date, several potential mechanisms have been suggested about how the probiotics regulate BP, including the changes of short‐chain fatty acids (Yang, Santisteban, et al., 2015) and polyphenols (Ahren et al., 2015), the improvement of immune system (Schiffrin, 2010) and inflammatory responses (Toral et al., 2014), the regulation of insulin sensitivity (Lye et al., 2009), sympathetic nerve activity (Tanida et al., 2014), and angiotensin‐converting enzymes (Thushara et al., 2016; Yang, Jiang, et al., 2015). Thus, a great interest has been raised in using probiotic supplementation to correct microbiota disturbances and control high BP. Pooled analysis on the antihypertensive effect of probiotics and their fermented products has shown positive results. In these meta‐analyses, the office systolic and diastolic BP reduced by 2.05 to 3.56/1.09 to 2.38 mmHg (Chi et al., 2020; Dong et al., 2013; Khalesi et al., 2014; Qi et al., 2020), which was in agreement with the findings of our study. However, different from previous analyses, the intervention duration of studies in our current analysis was restricted to ≥8 weeks. We further calculated the tertiles of treatment duration and found that with the increase of treatment duration, no further improvement in the size of BP reduction was observed. Similarly, Qi et al. reported in their analysis that the effect in controlling BP could only last for 8 or 10 weeks, but not for a long‐term time (Qi et al., 2020). Considering that 8 of 23 included studies by Qi et al. used probiotics combined with other functional ingredients which might influence the effect of BP regulation, our study provided further evidence of the long‐term effect of probiotic supplementation on BP. Taking the results of our study and these previous studies together, we speculate that it may take a short time for the probiotics to establish a homeostasis in the host gut and we are planning to further carry out the relevant research work. In addition, our study found a larger but nonsignificant reduction of office BP in diabetic and hypertensive patients compared with nondiabetic and normotensive adults. The nonsignificant reduction revealed that further supplementation of probiotic products in healthy subjects could also improve blood pressure significantly. Moreover, findings from the subgroup analysis indicated that the reduction in BP may be greater in patients using antihypertensive drugs, and the impact of unbalanced antihypertensive drug treatment between the hypertensive and normotensive patients may explain the difference. However, the data available on the metabolism of antihypertensive drugs and the effects of probiotics are still preliminary, so further studies are needed to clarify the relationship.

Another important finding of this meta‐analysis was the preferential ambulatory BP lowering of the probiotics supplementation. All studies included in our analysis showed a beneficial effect on ambulatory BP compared with placebo supplementation (Hove et al., 2015; Jauhiainen et al., 2012; Usinger et al., 2010). The differences in BP changes could be the consequence of discrepancies between the probiotic sources, combined with different study designs in terms of probiotic dosages, duration of treatment, and races. Pooled results of our study showed that the between‐treatment difference in ambulatory systolic/diastolic blood pressure tended to be statistically significant (*p* ≤ .052), in favor of the probiotic supplementation. However, due to the small number of included studies, the difference is relatively small. More large‐scale clinical trials are needed to explore the effect of probiotics on the improvement of ambulatory blood pressure.

There seems to be no relationship between BP‐lowering effect of probiotics and body weight reduction in our study. Of the 28 studies involved in our analysis, 13 studies reported a reduction of BMI after consuming probiotics (Ahn et al., 2015; Firouzi et al., 2017; Hariri et al., 2015; Hata et al., 1996; Ibrahim et al., 2018; Jones et al., 2012; Jung et al., 2013; Jung et al., 2015; Khalili et al., 2019; Michael et al., 2021; Mobini et al., 2017; Romao et al., 2020; Szulinska et al., 2018), another 4 studies showed that probiotic intervention did not change BMI together with a pure reduction in BP (Agerholm‐Larsen et al., 2000; Costabile et al., 2017; Lee, Lee, et al., 2017; Naito et al., 2018). Previous studies confirmed that a reduction in body weight or BMI could reduce BP (Sharafedtinov et al., 2013; Szulinska et al., 2018); however, whether there is a relationship between BMI and the improvement of BP after probiotics intervention is still controversial. Khalesi et al. showed in their meta‐analysis that no significant reduction of body weight was observed after probiotics consumption (Khalesi et al., 2014). Hariri et al. also found in type 2 diabetic patients that the supplementation with probiotic soy milk (containing *Lactobacillus planetarium A7*) reduced both systolic and diastolic BP, but did not change the anthropometric parameters (especially BMI)(Hariri et al., 2015). On the other hand, a study from Hendijani et al. reported that the effectiveness of probiotics consumption on BP in obese diabetic patients was less significant (Hendijani & Akbari, 2018). These observations suggested that the probiotics could lower the BP beyond body weight changes. However, further studies are required to verify this issue.

The effects of *Lactobacillus* on human health, such as the regulation of the gut microbiota populations and immune pathways, have been widely studied (Brisbin et al., 2010; Ou et al., 2011). It is surprising that most of the studies in the present analysis used *Lactobacilli* as a supplementation. Animal studies showed that using different species of *Lactobacillus* could reduce BP by increasing the level of nitric oxide (NO), decreasing levels of endothelin (ET), and secreting angiotensin‐converting enzyme inhibitory peptides (Yang, Jiang, et al., 2015). Randomized, controlled clinical trial showed that the supplementation with probiotics containing *Lactobacilli* significantly reduced BP in hypertensive and diabetic patients (Hata et al., 1996; Hsieh et al., 2018; Usinger et al., 2010). However, most of the current studies have used the mixture of probiotics as a supplementation, and the lack of trials focused on specific species of probiotics made it insufficient to analyze the effect of different species on BP control. Thus, further researches are needed to clarify which probiotics have a better antihypertensive effect.

Our study has to be interpreted with the context of its limitations. First, the present analysis, similar to other meta‐analyses, could only extract BP data from reported studies, and hence cannot specifically exclude confounding factors such as blood lipids and heart rate changes, which have a synergistic effect on BP. Second, a relatively small number of hypertensive patients were included in the analysis. The low baseline BP might have an impact on the extent of BP reduction. Third, most trials had a relatively small sample size (*n* < 60). Chance finding is possible.

## 5 CONCLUSIONS

In conclusion, the results of our study suggest that long‐term probiotics consumption (≥8 weeks) had a beneficial effect on BP and the changes in BP were not associated with the increase of age, BMI, and treatment duration. We believe that with more good design, large sample size, and long follow‐up time clinical trials in the future, probiotics consumption could play a big role in the long‐term management of hypertension.

## CONFLICT OF INTEREST

The authors declare no conflicts of interest.

## Supporting information


Figure S1

Table S1

Table S2
