# Supplementary figures and images for: Long‐term use of probiotics for the management of office and ambulatory blood pressure: A systematic review and meta‐analysis of randomized, controlled trials
Source: Food Sci Nutr. 2022 Sep 20;11(1):101–13. doi: 10.1002/fsn3.3069 (PMC9834877; doi:10.1002/fsn3.3069)

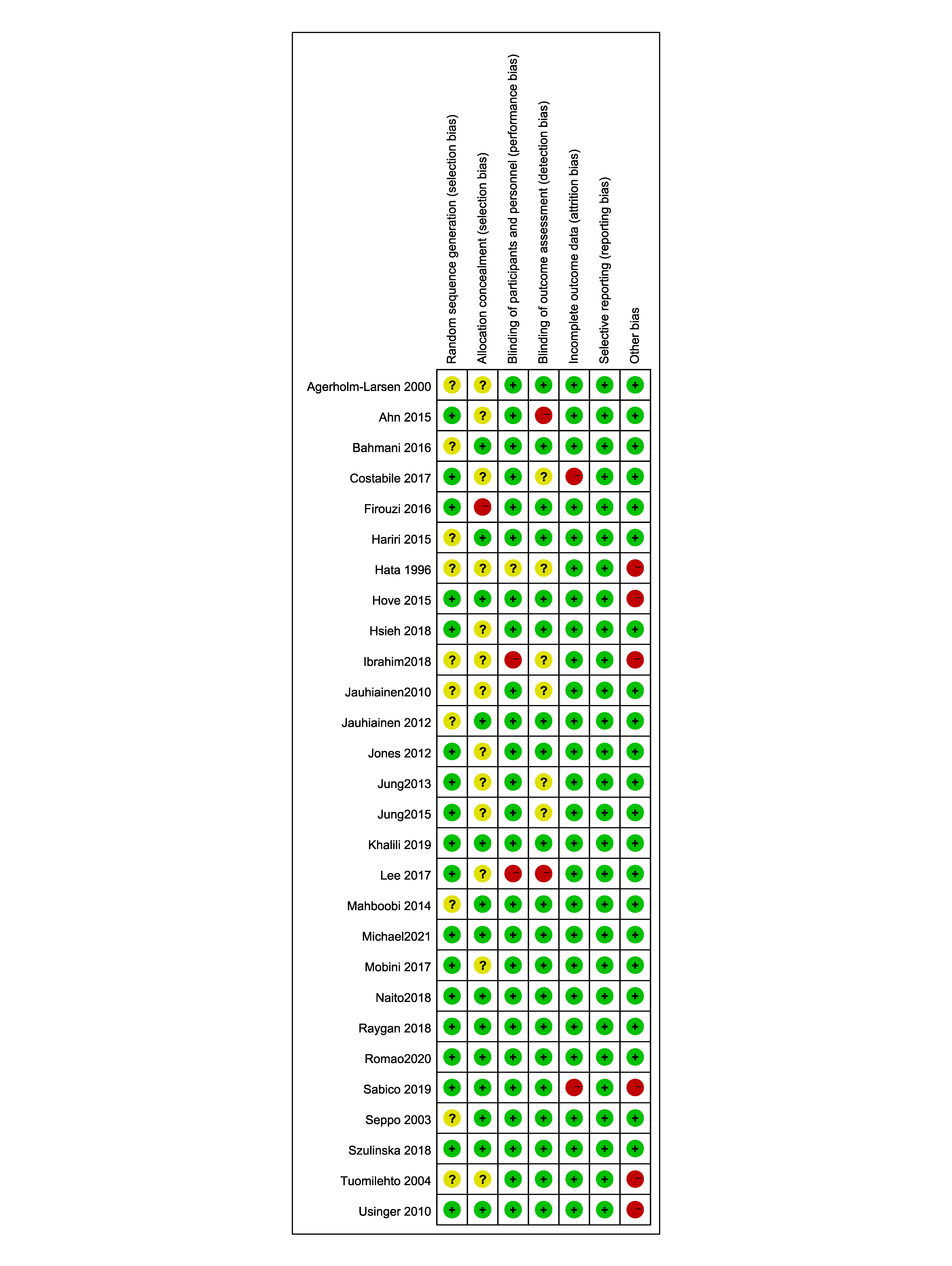

Supplement: Supplementary file 1 — Figure S1 [file FSN3-11-101-s001.tiff]
