# Supplementary material for: Long‐term use of probiotics for the management of office and ambulatory blood pressure: A systematic review and meta‐analysis of randomized, controlled trials
Source: Food Sci Nutr. 2022 Sep 20;11(1):101–13. doi: 10.1002/fsn3.3069 (PMC9834877; doi:10.1002/fsn3.3069)
Supplement: Supplementary file 2 — Table S1 [file FSN3-11-101-s003.docx]

Supplementary Table 1. Search strategy.

| Pubmed |  | (((((((((microbiota) OR (gut microbiota)) OR (probiotics)) OR (fermented milk)) OR (Kefir)) OR (Lactobacillus)) OR (streptococcus)) OR (bifidobacteria)) AND (blood pressure)) AND ((clinicalstudy[Filter] OR clinicaltrial[Filter] OR controlledclinicaltrial[Filter] OR randomizedcontrolledtrial[Filter]) AND (fft[Filter]) AND (humans[Filter])) Filters: Full text, Clinical Study, Clinical Trial, Controlled Clinical Trial, Randomized Controlled Trial, Humans |
| --- | --- | --- |
| Embase |  | (‘microbiota’/mj OR gut microbiota’:ti OR ‘probiotics’:ti OR ‘fermented milk’:ti OR ‘Kefir’:ti OR . . . OR ‘Lactobacillus’:ti OR‘streptococcus’:ti OR ‘bifidobacter’:ti) AND (‘blood pressure’/mj OR ‘hypertension’:ti OR ‘normotension’:ti OR ‘metabolism syndrome’:ti OR ‘arterial hypertension’:ti) |
| Cochrane Library |  | ((MeSH descriptor: [Microbiota] explode all trees) OR (MeSH descriptor: [Gut microbiota] explode all trees) OR (MeSH descriptor: [Probiotics] explode all trees) OR (MeSH descriptor: [Fermented milk] explode all trees) OR (MeSH descriptor: [Kefir] explode all trees) OR (MeSH descriptor: [Lactobacillus] explode all trees) OR (MeSH descriptor: [Streptococcus] explode all trees) OR (MeSH descriptor: [Bifidobacter] explode all trees) OR ((Microbiota OR Gut microbiota OR Probiotics OR Fermented milk OR Kefir OR Lactobacillus OR Streptococcus OR Bifidobacter):ti)) AND ((MeSH descriptor: [Blood pressure] explode all trees) OR (MeSH descriptor: [Hypertension] explode all trees) OR (MeSH descriptor: [Normotension] explode all trees) OR (MeSH descriptor: [Metabolism syndrome] explode all trees) OR ((Blood pressure OR Hypertension OR Normotension OR Metabolism syndrome):ti)) |
