# Supplementary material for: Long‐term use of probiotics for the management of office and ambulatory blood pressure: A systematic review and meta‐analysis of randomized, controlled trials
Source: Food Sci Nutr. 2022 Sep 20;11(1):101–13. doi: 10.1002/fsn3.3069 (PMC9834877; doi:10.1002/fsn3.3069)
Supplement: Supplementary file 3 — Table S2 [file FSN3-11-101-s002.docx]

Supplementary Table 2. The reference lists of excluded articles.

[1] DU H, Xu X M, Xu T, et al. [Effects of gut microbiota on five absorbed components of Berberis kansuensis in rat serum by HPLC-QqQ-MS] . Zhongguo Zhong Yao Za Zhi, 2020,45(2):418-424.

[2] DU HX, Xiao G X, DU XL, et al. [New effect of G-protein coupled receptors on blood pressure regulation] . Zhongguo Zhong Yao Za Zhi, 2021,46(1):6-14.

[3] Aagaard K, Petrosino J, Keitel W, et al. The Human Microbiome Project strategy for comprehensive sampling of the human microbiome and why it matters . FASEB J, 2013,27(3):1012-1022.

[4] Abais-Battad J M, Mattson D L. Influence of dietary protein on Dahl salt-sensitive hypertension: a potential role for gut microbiota . Am J Physiol Regul Integr Comp Physiol, 2018,315(5):R907-R914.

[5] Abais-Battad J M, Saravia F L, Lund H, et al. Dietary influences on the Dahl SS rat gut microbiota and its effects on salt-sensitive hypertension and renal damage . Acta Physiol (Oxf), 2021,232(4):e13662.

[6] Abbasalizad F M, Vajdi M. Gut microbiota-associated trimethylamine N-oxide and increased cardiometabolic risk in adults: a systematic review and dose-response meta-analysis . Nutr Rev, 2021,79(9):1022-1042.

[7] Abegaz S B. Human ABO Blood Groups and Their Associations with Different Diseases . Biomed Res Int, 2021,2021:6629060.

[8] Abenavoli L, Boccuto L, Federico A, et al. Diet and Non-Alcoholic Fatty Liver Disease: The Mediterranean Way . Int J Environ Res Public Health, 2019,16(17).

[9] Abman S H, Kinsella J P, Schaffer M S, et al. Inhaled nitric oxide in the management of a premature newborn with severe respiratory distress and pulmonary hypertension . Pediatrics, 1993,92(4):606-609.

[10] Abramson O, Dagan R, Tal A, et al. Severe complications of measles requiring intensive care in infants and young children . Arch Pediatr Adolesc Med, 1995,149(11):1237-1240.

[11] Abruzzo A, Bigucci F, Cerchiara T, et al. Mucoadhesive chitosan/gelatin films for buccal delivery of propranolol hydrochloride . Carbohydr Polym, 2012,87(1):581-588.

[12] Adams C, Sawh F, Green-Johnson J M, et al. Characterization of casein-derived peptide bioactivity: Differential effects on angiotensin-converting enzyme inhibition and cytokine and nitric oxide production . J Dairy Sci, 2020,103(7):5805-5815.

[13] Afessa B, Greaves W L, Frederick W R. Pneumococcal bacteremia in adults: a 14-year experience in an inner-city university hospital . Clin Infect Dis, 1995,21(2):345-351.

[14] Africa J A, Newton K P, Schwimmer J B. Lifestyle Interventions Including Nutrition, Exercise, and Supplements for Nonalcoholic Fatty Liver Disease in Children . Dig Dis Sci, 2016,61(5):1375-1386.

[15] Afzaal M, Saeed F, Anjum F, et al. Nutritional and ethnomedicinal scenario of koumiss: A concurrent review . Food Sci Nutr, 2021,9(11):6421-6428.

[16] Agarkova E Y, Kruchinin A G, Glazunova O A, et al. Whey Protein Hydrolysate and Pumpkin Pectin as Nutraceutical and Prebiotic Components in a Functional Mousse with Antihypertensive and Bifidogenic Properties . Nutrients, 2019,11(12).

[17] Aguilar A. Hypertension: Microbiota under pressure . Nat Rev Nephrol, 2017,13(1):3.

[18] Agustí A. Predicting the future from the past . Eur Respir J, 2017,49(1).

[19] Ahmad A F, Dwivedi G, O'Gara F, et al. The gut microbiome and cardiovascular disease: current knowledge and clinical potential . Am J Physiol Heart Circ Physiol, 2019,317(5):H923-H938.

[20] Ahmad A F, Ward N C, Dwivedi G. The gut microbiome and heart failure . Curr Opin Cardiol, 2019,34(2):225-232.

[21] Ahmadi S, Mainali R, Nagpal R, et al. Dietary Polysaccharides in the Amelioration of Gut Microbiome Dysbiosis and Metabolic Diseases . Obes Control Ther, 2017,4(3).

[22] Ahmadian E, Rahbar S Y, Hosseiniyan K S, et al. Pre-Eclampsia: Microbiota possibly playing a role . Pharmacol Res, 2020,155:104692.

[23] Ahmadmehrabi S, Tang W. Gut microbiome and its role in cardiovascular diseases . Curr Opin Cardiol, 2017,32(6):761-766.

[24] Ahmed A A, Salih M A, Ahmed H S. Post-endemic acute bacterial meningitis in Sudanese children . East Afr Med J, 1996,73(8):527-532.

[25] Ahmed S, Spence J D. Sex differences in the intestinal microbiome: interactions with risk factors for atherosclerosis and cardiovascular disease . Biol Sex Differ, 2021,12(1):35.

[26] Ahn Y, Nam M H, Kim E. Relationship Between the Gastrointestinal Side Effects of an Anti-Hypertensive Medication and Changes in the Serum Lipid Metabolome . Nutrients, 2020,12(1).

[27] Ahrens A P, Culpepper T, Saldivar B, et al. A Six-Day, Lifestyle-Based Immersion Program Mitigates Cardiovascular Risk Factors and Induces Shifts in Gut Microbiota, Specifically Lachnospiraceae, Ruminococcaceae, Faecalibacterium prausnitzii: A Pilot Study . Nutrients, 2021,13(10).

[28] Ahtesh F B, Stojanovska L, Apostolopoulos V. Anti-hypertensive peptides released from milk proteins by probiotics . Maturitas, 2018,115:103-109.

[29] Aida Z, Lamia A, Souheil Z, et al. Meningitis due to Streptococcus equi in a 73 year old woman with an osteodural defect . IDCases, 2020,21:e779.

[30] Aihara K, Kajimoto O, Hirata H, et al. Effect of powdered fermented milk with Lactobacillus helveticus on subjects with high-normal blood pressure or mild hypertension . J Am Coll Nutr, 2005,24(4):257-265.

[31] Ajebli M, Amssayef A, Akdad M, et al. Chronic Diseases and COVID-19: A Review . Endocr Metab Immune Disord Drug Targets, 2021,21(10):1781-1803.

[32] Al K S, Reichert B, Shatat I F. The Microbiome and Blood Pressure: Can Microbes Regulate Our Blood Pressure? . Front Pediatr, 2017,5:138.

[33] Alade G O, Ayanbadejo P O, Umeizudike K A, et al. Association of Elevated C-Reactive Protein with Severe Periodontitis in Hypertensive Patients in Lagos, Nigeria: A Pilot Study . Contemp Clin Dent, 2018,9(Suppl 1):S95-S99.

[34] Alba C M, Daya M, Franck C. Tart Cherries and health: Current knowledge and need for a better understanding of the fate of phytochemicals in the human gastrointestinal tract . Crit Rev Food Sci Nutr, 2019,59(4):626-638.

[35] Aldarhami A, Felek A, Sharma V, et al. Purification and characterization of nisin P produced by a strain of Streptococcus gallolyticus . J Med Microbiol, 2020,69(4):605-616.

[36] Aldás I, Menéndez R, Méndez R, et al. Early and Late Cardiovascular Events in Patients Hospitalized for Community-Acquired Pneumonia . Arch Bronconeumol (Engl Ed), 2020,56(9):551-558.

[37] Aldred M A. Food for Thought: The Emerging Role of Intestinal Microbiota in Pulmonary Arterial Hypertension . Am J Respir Cell Mol Biol, 2022,66(4):361-362.

[38] Aleixandre A, Miguel M, Muguerza B. [Peptides with antihypertensive activity from milk and egg proteins] . Nutr Hosp, 2008,23(4):313-318.

[39] Alfawaz H A, Aljumah A A. What improves minimal hepatic encephalopathy: probiotic yogurt, protein restriction or nonabsorbable disaccharides? . Saudi J Gastroenterol, 2012,18(3):153-154.

[40] Alhajri N, Khursheed R, Ali M T, et al. Cardiovascular Health and The Intestinal Microbial Ecosystem: The Impact of Cardiovascular Therapies on The Gut Microbiota . Microorganisms, 2021,9(10).

[41] Alherbish A, Charrois T L, Ackman M L, et al. The prevalence of natural health product use in patients with acute cardiovascular disease . PLoS One, 2011,6(5):e19623.

[42] Ali A, Goldberg R N, Suguihara C, et al. Effects of ATP-magnesium chloride on the cardiopulmonary manifestations of group B streptococcal sepsis in the piglet . Pediatr Res, 1996,39(4 Pt 1):609-615.

[43] Ali B, Khan K Y, Majeed H, et al. Soymilk-Cow's milk ACE-inhibiting enzyme modified cheese . Food Chem, 2017,237:1083-1091.

[44] Ali Z. Neonatal meningitis: a 3-year retrospective study at the Mount Hope Women’s Hospital, Trinidad, West Indies . J Trop Pediatr, 1995,41(2):109-111.

[45] Alidoost M, Conte G A, Chaudry R, et al. A Unique Presentation of Spontaneous Compartment Syndrome due to Acquired Hemophilia A and Associated Malignancy: Case Report and Literature Review . World J Oncol, 2020,11(2):72-75.

[46] Alipour H, Gazerani P, Heidari M, et al. Modulatory Effect of Probiotic Lactobacillus rhamnosus PB01 on Mechanical Sensitivity in a Female Diet-Induced Obesity Model . Pain Res Manag, 2021,2021:5563959.

[47] Allen E M, Rowin M, Pappas J B, et al. Hemodynamic effects of N-acetylamrinone in a porcine model of group B streptococcal sepsis . Drug Metab Dispos, 1996,24(9):1028-1031.

[48] Aller M A, Arias N, Blanco-Rivero J, et al. Hepatic encephalopathy: Sometimes more portal than hepatic . J Gastroenterol Hepatol, 2019,34(3):490-494.

[49] Allison S J. Hypertension: Salt: the microbiome, immune function and hypertension . Nat Rev Nephrol, 2018,14(2):71.

[50] Almasanu B P, Owensby J R, Pavlakis S G, et al. Spinal cord infarction in meningitis: polygenic risk factors . Pediatr Neurol, 2005,32(2):124-126.

[51] Almeida C, Barata P, Fernandes R. The influence of gut microbiota in cardiovascular diseases-a brief review . Porto Biomed J, 2021,6(1):e106.

[52] Almutairi D M, Alqahtani R M, Alshareef N, et al. Deep Neck Space Infections: A Retrospective Study of 183 Cases at a Tertiary Hospital . Cureus, 2020,12(2):e6841.

[53] Alpern E R, Alessandrini E A, McGowan K L, et al. Serotype prevalence of occult pneumococcal bacteremia . Pediatrics, 2001,108(2):E23.

[54] Al-Rubaye H, Perfetti G, Kaski J C. The Role of Microbiota in Cardiovascular Risk: Focus on Trimethylamine Oxide . Curr Probl Cardiol, 2019,44(6):182-196.

[55] Alsahhar J S, Rahimi R S. Updates on the pathophysiology and therapeutic targets for hepatic encephalopathy . Curr Opin Gastroenterol, 2019,35(3):145-154.

[56] Al-Sweih N, Maiyegun S, Diejomaoh M, et al. Streptococcus agalactiae (Group B Streptococci) carriage in late pregnancy in Kuwait . Med Princ Pract, 2004,13(1):10-14.

[57] Altemani F, Barrett H L, Callaway L K, et al. Reduced Abundance of Nitrate-Reducing Bacteria in the Oral Microbiota of Women with Future Preeclampsia . Nutrients, 2022,14(6).

[58] Altemani F, Barrett H L, Gomez-Arango L, et al. Pregnant women who develop preeclampsia have lower abundance of the butyrate-producer Coprococcus in their gut microbiota . Pregnancy Hypertens, 2021,23:211-219.

[59] Alu'Datt M H, Al-U'Datt D, Alhamad M N, et al. Characterization and biological properties of peptides isolated from dried fermented cow milk products by RP-HPLC: Amino acid composition, antioxidant, antihypertensive, and antidiabetic properties . J Food Sci, 2021,86(7):3046-3060.

[60] Alvareza M D, Subramaniam A, Tang Y, et al. Obesity as an independent risk factor for group B streptococcal colonization . J Matern Fetal Neonatal Med, 2017,30(23):2876-2879.

[61] Alzahrani H S, Jackson K G, Hobbs D A, et al. The role of dietary nitrate and the oral microbiome on blood pressure and vascular tone . Nutr Res Rev, 2021,34(2):222-239.

[62] Alzand B S, Meeder J G, Koster A. Purulent pericarditis, an uncommon entity in modern practice: a case report . Neth Heart J, 2006,14(9):309-311.

[63] Amabebe E, Anumba D O. Diabetogenically beneficial gut microbiota alterations in third trimester of pregnancy . Reprod Fertil, 2021,2(1):R1-R12.

[64] Amar J. [Host-microbiota crosstalk and cardiovascular diseases] . Presse Med, 2018,47(9):775-779.

[65] Amar J. Microbiota-Host Crosstalk: A Bridge Between Cardiovascular Risk Factors, Diet, and Cardiovascular Disease . Am J Hypertens, 2018,31(8):941-944.

[66] Ambalavanan N, Bulger A, Ware J, et al. Hemodynamic effects of levcromakalim in neonatal porcine pulmonary hypertension . Biol Neonate, 2001,80(1):74-80.

[67] Ambalavanan N, Philips J R, Bulger A, et al. Endothelin-A receptor blockade in porcine pulmonary hypertension . Pediatr Res, 2002,52(6):913-921.

[68] Amedei A, Morbidelli L. Circulating Metabolites Originating from Gut Microbiota Control Endothelial Cell Function . Molecules, 2019,24(21).

[69] Amirou M, Lombart D, Thomas K, et al. [Recurring peritonitis due to Streptococcus from the upper respiratory tract in a saxophone player under peritoneal dialysis] . Ann Biol Clin (Paris), 2012,70(2):207-209.

[70] Amorim F G, Coitinho L B, Dias A T, et al. Identification of new bioactive peptides from Kefir milk through proteopeptidomics: Bioprospection of antihypertensive molecules . Food Chem, 2019,282:109-119.

[71] Ampatzoglou A, Atwal K K, Maidens C M, et al. Increased whole grain consumption does not affect blood biochemistry, body composition, or gut microbiology in healthy, low-habitual whole grain consumers . J Nutr, 2015,145(2):215-221.

[72] Anderson G, Mazzoccoli G. Left Ventricular Hypertrophy: Roles of Mitochondria CYP1B1 and Melatonergic Pathways in Co-Ordinating Wider Pathophysiology . Int J Mol Sci, 2019,20(16).

[73] Anderson J J, Nieman D C. Diet Quality-The Greeks Had It Right! . Nutrients, 2016,8(10).

[74] Anderson M E, Burnette T M, Geiser D R, et al. Magnesium attenuates pulmonary hypertension due to hypoxia and group B streptococci . J Appl Physiol (1985), 1994,77(2):751-756.

[75] Andreeva-Gateva P A, Mihaleva I D, Dimova I I. Type 2 diabetes mellitus and cardiovascular risk; what the pharmacotherapy can change through the epigenetics . Postgrad Med, 2020,132(2):109-125.

[76] Angelino D, Berhow M, Ninfali P, et al. Caecal absorption of vitexin-2-O-xyloside and its aglycone apigenin, in the rat . Food Funct, 2013,4(9):1339-1345.

[77] Angeloni C, Businaro R, Vauzour D. The role of diet in preventing and reducing cognitive decline . Curr Opin Psychiatry, 2020,33(4):432-438.

[78] Angelov A, Yaneva-Marinova T, Gotcheva V. Oats as a matrix of choice for developing fermented functional beverages . J Food Sci Technol, 2018,55(7):2351-2360.

[79] Angoorani P, Ejtahed H S, Hasani-Ranjbar S, et al. Gut microbiota modulation as a possible mediating mechanism for fasting-induced alleviation of metabolic complications: a systematic review . Nutr Metab (Lond), 2021,18(1):105.

[80] Angstwurm K, Freyer D, Dirnagl U, et al. Tumour necrosis factor alpha induces only minor inflammatory changes in the central nervous system, but augments experimental meningitis . Neuroscience, 1998,86(2):627-634.

[81] Angstwurm K, Reuss S, Freyer D, et al. Induced hypothermia in experimental pneumococcal meningitis . J Cereb Blood Flow Metab, 2000,20(5):834-838.

[82] Anker-Ladefoged C, Langkamp T, Mueller-Alcazar A. The Potential Impact of Selected Bacterial Strains on the Stress Response . Healthcare (Basel), 2021,9(5).

[83] Annalisa N, Alessio T, Claudette T D, et al. Gut microbioma population: an indicator really sensible to any change in age, diet, metabolic syndrome, and life-style . Mediators Inflamm, 2014,2014:901308.

[84] Ansari A S, Dennis B B, Shah D, et al. An unusual case of infective pneumocephalus: case report of pneumocephalus exacerbated by continuous positive airway pressure . BMC Emerg Med, 2018,18(1):2.

[85] Anselmi G, Gagliardi L, Egidi G, et al. Gut Microbiota and Cardiovascular Diseases: A Critical Review . Cardiol Rev, 2021,29(4):195-204.

[86] Anyasi T A, Jideani A, Mchau G. Functional Properties and Postharvest Utilization of Commercial and Noncommercial Banana Cultivars . Compr Rev Food Sci Food Saf, 2013,12(5):509-522.

[87] Aoyagi Y, Park S, Matsubara S, et al. Habitual intake of fermented milk products containing Lactobacillus casei strain Shirota and a reduced risk of hypertension in older people . Benef Microbes, 2017,8(1):23-29.

[88] Arab J P, Martin-Mateos R M, Shah V H. Gut-liver axis, cirrhosis and portal hypertension: the chicken and the egg . Hepatol Int, 2018,12(Suppl 1):24-33.

[89] Arabi S M, Bahrami L S, Rahnama I, et al. Impact of synbiotic supplementation on cardiometabolic and anthropometric indices in patients with metabolic syndrome: A systematic review and meta-analysis of randomized controlled trials . Pharmacol Res, 2022,176:106061.

[90] Aran A, Lin L, Finn L A, et al. Post-streptococcal antibodies are associated with metabolic syndrome in a population-based cohort . PLoS One, 2011,6(9):e25017.

[91] Arias-Mutis O J, Marrachelli V G, Ruiz-Saurí A, et al. Development and characterization of an experimental model of diet-induced metabolic syndrome in rabbit . PLoS One, 2017,12(5):e178315.

[92] Aron-Wisnewsky J, Prifti E, Belda E, et al. Major microbiota dysbiosis in severe obesity: fate after bariatric surgery . Gut, 2019,68(1):70-82.

[93] Arushothy R, Ramasamy H, Hashim R, et al. Multidrug-resistant Streptococcus pneumoniae causing invasive pneumococcal disease isolated from a paediatric patient . Int J Infect Dis, 2020,90:219-222.

[94] Aryal S, Alimadadi A, Manandhar I, et al. Machine Learning Strategy for Gut Microbiome-Based Diagnostic Screening of Cardiovascular Disease . Hypertension, 2020,76(5):1555-1562.

[95] Ascione T, Di Flumeri G, Boccia G, et al. Infections in patients affected by liver cirrhosis: an update . Infez Med, 2017,25(2):91-97.

[96] Ashworth A, Cutler C, Farnham G, et al. Dietary intake of inorganic nitrate in vegetarians and omnivores and its impact on blood pressure, resting metabolic rate and the oral microbiome . Free Radic Biol Med, 2019,138:63-72.

[97] Asoodeh A, Zardini H Z, Chamani J. Identification and characterization of two novel antimicrobial peptides, temporin-Ra and temporin-Rb, from skin secretions of the marsh frog (Rana ridibunda) . J Pept Sci, 2012,18(1):10-16.

[98] Aspri M, Leni G, Galaverna G, et al. Bioactive properties of fermented donkey milk, before and after in vitro simulated gastrointestinal digestion . Food Chem, 2018,268:476-484.

[99] Astrup A. Yogurt and dairy product consumption to prevent cardiometabolic diseases: epidemiologic and experimental studies . Am J Clin Nutr, 2014,99(5 Suppl):1235S-1242S.

[100] Astrup A, Geiker N, Magkos F. Effects of Full-Fat and Fermented Dairy Products on Cardiometabolic Disease: Food Is More Than the Sum of Its Parts . Adv Nutr, 2019,10(5):924S-930S.

[101] Atikel Y Ö, Bakkaloğlu S A, Paglialonga F, et al. Influenza and pneumococcus vaccination rates in pediatric dialysis patients in Europe: recommendations vs reality A European Pediatric Dialysis Working Group and European Society for Pediatric Nephrology Dialysis Working Group study . Turk J Med Sci, 2021,51(6):2881-2886.

[102] Attaye I, Warmbrunn M V, Boot A, et al. A Systematic Review and Meta-analysis of Dietary Interventions Modulating Gut Microbiota and Cardiometabolic Diseases-Striving for New Standards in Microbiome Studies . Gastroenterology, 2022.

[103] Auten G M, Preheim L C, Sookpranee M, et al. High-pressure liquid chromatography and microbiological assay of serum ofloxacin levels in adults receiving intravenous and oral therapy for skin infections . Antimicrob Agents Chemother, 1991,35(12):2558-2561.

[104] Avery E G, Bartolomaeus H, Maifeld A, et al. The Gut Microbiome in Hypertension: Recent Advances and Future Perspectives . Circ Res, 2021,128(7):934-950.

[105] Ávila-Escalante M L, Coop-Gamas F, Cervantes-Rodríguez M, et al. The effect of diet on oxidative stress and metabolic diseases-Clinically controlled trials . J Food Biochem, 2020,44(5):e13191.

[106] Avolio E, Gualtieri P, Romano L, et al. Obesity and Body Composition in Man and Woman: Associated Diseases and the New Role of Gut Microbiota . Curr Med Chem, 2020,27(2):216-229.

[107] Axelrod R S, Havas H F, Murasko D M, et al. Effect of the mixed bacterial vaccine on the immune response of patients with non-small cell lung cancer and refractory malignancies . Cancer, 1988,61(11):2219-2230.

[108] Aya V, Flórez A, Perez L, et al. Association between physical activity and changes in intestinal microbiota composition: A systematic review . PLoS One, 2021,16(2):e247039.

[109] Aydin S, Ugur K, Aydin S. Could excessive production of tyramine by the microbiota be a reason for essential hypertension? . Biosci Microbiota Food Health, 2018,37(4):77-78.

[110] Aydin S, Ustebay S, Baykus Y, et al. May probable cause of hypertension in hypertensive disorders of pregnancy be over expressing tyramine depending deterioration of microbiota composition . Med Hypotheses, 2019,122:139-140.

[111] Azegami T, Yuki Y, Hayashi K, et al. Intranasal vaccination against angiotensin II type 1 receptor and pneumococcal surface protein A attenuates hypertension and pneumococcal infection in rodents . J Hypertens, 2018,36(2):387-394.

[112] Aziz S M, Pauly T H, Gillespie M N. Intrinsic microbicidal activity and pulmonary hypertension in isolated newborn piglet lungs . Pediatr Res, 1993,34(1):32-37.

[113] Azizi N F, Kumar M R, Yeap S K, et al. Kefir and Its Biological Activities . Foods, 2021,10(6).

[114] Baena-Monroy T, Moreno-Maldonado V, Franco-Martínez F, et al. Candida albicans, Staphylococcus aureus and Streptococcus mutans colonization in patients wearing dental prosthesis . Med Oral Patol Oral Cir Bucal, 2005,10 Suppl 1:E27-E39.

[115] Baffy G. Potential mechanisms linking gut microbiota and portal hypertension . Liver Int, 2019,39(4):598-609.

[116] Baglie S, Del R A, Motta R H, et al. Plasma and salivary amoxicillin concentrations and effect against oral microorganisms . Int J Clin Pharmacol Ther, 2007,45(10):556-562.

[117] Baharestani M M. Negative pressure wound therapy in the adjunctive management of necrotizing fascitis: examining clinical outcomes . Ostomy Wound Manage, 2008,54(4):44-50.

[118] Bajaj J S. Review article: potential mechanisms of action of rifaximin in the management of hepatic encephalopathy and other complications of cirrhosis . Aliment Pharmacol Ther, 2016,43 Suppl 1:11-26.

[119] Baker L, Carlson R. Streptococcus acidominimus isolated from a multiloculated empyema in a critically ill adult man with pneumonia: case report and review of literature . Heart Lung, 2008,37(4):308-310.

[120] Balasubramanian H, Patole S. Early probiotics to prevent childhood metabolic syndrome: A systematic review . World J Methodol, 2015,5(3):157-163.

[121] Baldwin D S. Poststreptococcal glomerulonephritis. A progressive disease? . Am J Med, 1977,62(1):1-11.

[122] Balejko E, Balejko J, Plust D. Assessment of the Effect of Dietary Modifications and Bioenteric Intragastric Balloon Treatment on the Changes of Some Morphological and Biochemical Parameters in Obese Patients . Ann Nutr Metab, 2018,73(4):290-301.

[123] Balliett M, Burke J R. Changes in anthropometric measurements, body composition, blood pressure, lipid profile, and testosterone in patients participating in a low-energy dietary intervention . J Chiropr Med, 2013,12(1):3-14.

[124] Balvers M, van den Born B H, Levin E, et al. Impact drugs targeting cardiometabolic risk on the gut microbiota . Curr Opin Lipidol, 2021,32(1):38-54.

[125] Bandel J W, Goldberg R N, Suguihara C, et al. Effects of anti-CD18 monoclonal antibody, R15.7, on the cardiopulmonary manifestations of group B streptococcal sepsis in piglets . Biol Neonate, 2000,78(2):121-128.

[126] Bao Z, Chi Y. In Vitro and In Vivo Assessment of Angiotensin-Converting Enzyme (ACE) Inhibitory Activity of Fermented Soybean Milk by Lactobacillus casei Strains . Curr Microbiol, 2016,73(2):214-219.

[127] Barakat B, Almeida M. Biochemical and immunological changes in obesity . Arch Biochem Biophys, 2021,708:108951.

[128] Barbadoro P, Ponzio E, Coccia E, et al. Association between hypertension, oral microbiome and salivary nitric oxide: A case-control study . Nitric Oxide, 2021,106:66-71.

[129] Barber T M, Kyrou I, Randeva H S, et al. Mechanisms of Insulin Resistance at the Crossroad of Obesity with Associated Metabolic Abnormalities and Cognitive Dysfunction J]. Int J Mol Sci, 2021,22(2).

[130] Barefield E S, Hicks T P, Philips J R. Thromboxane and pulmonary morphometry in the development of the pulmonary hypertensive response to group B streptococcus . Crit Care Med, 1994,22(3):506-514.

[131] Barke R A, Dunn D L, Dalmasso A, et al. Enterococcal sepsis and lung microvascular injury in sheep . Arch Surg, 1990,125(4):437-440.

[132] Barna I, Nyúl D, Szentes T, et al. [Review of the relation between gut microbiome, metabolic disease and hypertension] . Orv Hetil, 2018,159(9):346-351.

[133] Barreiro T J, Asiimwe D D, Gemmel D, et al. Catastrophic chest pain: blinded by cardiopulmonary disease . BMJ Case Rep, 2015,2015.

[134] Barreto F M, Colado S A, Morimoto H K, et al. Beneficial effects of Lactobacillus plantarum on glycemia and homocysteine levels in postmenopausal women with metabolic syndrome . Nutrition, 2014,30(7-8):939-942.

[135] Barrington K J, Etches P C, Schulz R, et al. The hemodynamic effects of inhaled nitric oxide and endogenous nitric oxide synthesis blockade in newborn piglets during infusion of heat-killed group B streptococci . Crit Care Med, 2000,28(3):800-808.

[136] Barron M, Atkinson S N, Kirby J, et al. Sleeve gastrectomy prevents hypertension associated with unique shifts in the gut microbiome . Surg Endosc, 2021,35(10):5461-5467.

[137] Barrows I R, Ramezani A, Raj D S. Inflammation, Immunity, and Oxidative Stress in Hypertension-Partners in Crime? . Adv Chronic Kidney Dis, 2019,26(2):122-130.

[138] Barth R F, Maximilian B L, Cao L, et al. An Obesity Paradox: Increased Body Mass Index Is Associated with Decreased Aortic Atherosclerosis . Curr Hypertens Rep, 2017,19(7):55.

[139] Barthow C, Hood F, Crane J, et al. A randomised controlled trial of a probiotic and a prebiotic examining metabolic and mental health outcomes in adults with pre-diabetes . BMJ Open, 2022,12(3):e55214.

[140] Barthow C, Hood F, McKinlay E, et al. Food 4 Health - He Oranga Kai: Assessing the efficacy, acceptability and economic implications of Lactobacillus rhamnosus HN001 and β-glucan to improve glycated haemoglobin, metabolic health, and general well-being in adults with pre-diabetes: study protocol for a 2 × 2 factorial design, parallel group, placebo-controlled randomized controlled trial, with embedded qualitative study and economic analysis . Trials, 2019,20(1):464.

[141] Bartley A, Yang T, Arocha R, et al. Increased Abundance of Lactobacillales in the Colon of Beta-Adrenergic Receptor Knock Out Mouse Is Associated With Increased Gut Bacterial Production of Short Chain Fatty Acids and Reduced IL17 Expression in Circulating CD4(+) Immune Cells. Front Physiol, 2018,9:1593.

[142] Bartolomaeus H, Avery E G, Bartolomaeus T, et al. Blood pressure changes correlate with short-chain fatty acid production potential shifts under a synbiotic intervention . Cardiovasc Res, 2020,116(7):1252-1253.

[143] Bartolomaeus H, Balogh A, Yakoub M, et al. Short-Chain Fatty Acid Propionate Protects From Hypertensive Cardiovascular Damage . Circulation, 2019,139(11):1407-1421.

[144] Bartolomaeus H, Markó L, Wilck N, et al. Precarious Symbiosis Between Host and Microbiome in Cardiovascular Health. Hypertension, 2019,73(5):926-935.

[145] Basic-Jukic N, Juric I, Furic-Cunko V, et al. Central nervous system infections in renal transplant recipients . Transpl Infect Dis, 2020,22(4):e13341.

[146] Baspinar B, Güldaş M. Traditional plain yogurt: a therapeutic food for metabolic syndrome? Crit Rev Food Sci Nutr, 2021,61(18):3129-3143.

[147] Bateman R M, Sharpe M D, Jagger J E, et al. 36th International Symposium on Intensive Care and Emergency Medicine : Brussels, Belgium. 15-18 March 2016 . Crit Care, 2016,20(Suppl 2):94.

[148] Batista K S, de Albuquerque J G, Vasconcelos M, et al. Probiotics and prebiotics: potential prevention and therapeutic target for nutritional management of COVID-19? Nutr Res Rev, 2021:1-18.

[149] Batista R P, Ferreira C R. Streptococcus agalactiae septicemia in a patient with diabetes and hepatic cirrhosis. Autops Case Rep, 2015,5(4):35-43.

[150] Batson B N, Baliga R. Post-streptococcal hypertensive encephalopathy with normal urinalysis. Pediatr Nephrol, 2003,18(1):73.

[151] Battaglini D, Pimentel-Coelho P M, Robba C, et al. Gut Microbiota in Acute Ischemic Stroke: From Pathophysiology to Therapeutic Implications. Front Neurol, 2020,11:598.

[152] Battisha A, Madoukh B, Altibi A, et al. A rare presentation of Austrian syndrome with septic arthritis in an immunocompetent female . Egypt Heart J, 2019,71(1):9.

[153] Battson M L, Lee D M, Weir T L, et al. The gut microbiota as a novel regulator of cardiovascular function and disease. J Nutr Biochem, 2018,56:1-15.

[154] Baverstock R J, Carey C. Priapism as the Presenting Complaint in Fatal Group A Streptococcal Induced Disseminated Intravascular Coagulation. CJEM, 2018,20(S2):S6-S8.

[155] Bawdon R E, Lu Y S, Brater D C. High-pressure liquid chromatographic assay and pharmacokinetics of HR 810 after intramuscular injection in rabbits. Antimicrob Agents Chemother, 1985,27(4):436-438.

[156] Bayat A, Azizi-Soleiman F, Heidari-Beni M, et al. Effect of Cucurbita ficifolia and Probiotic Yogurt Consumption on Blood Glucose, Lipid Profile, and Inflammatory Marker in Type 2 Diabetes. Int J Prev Med, 2016,7:30.

[157] Beale A L, Kaye D M, Marques F Z. The role of the gut microbiome in sex differences in arterial pressure. Biol Sex Differ, 2019,10(1):22.

[158] Beale A L, O'Donnell J A, Nakai M E, et al. The Gut Microbiome of Heart Failure With Preserved Ejection Fraction . J Am Heart Assoc, 2021,10(13):e20654.

[159] Beath S V, Kelly D A. Total Parenteral Nutrition-Induced Cholestasis: Prevention and Management. Clin Liver Dis, 2016,20(1):159-176.

[160] Beaulieu J, Millette E, Trottier E, et al. Regulatory function of a malleable protein matrix as a novel fermented whey product on features defining the metabolic syndrome. J Med Food, 2010,13(3):509-519.

[161] Beckers K F, Sones J L. Maternal microbiome and the hypertensive disorder of pregnancy, preeclampsia. Am J Physiol Heart Circ Physiol, 2020,318(1):H1-H10.

[162] Becquet O, Pasche J, Gatti H, et al. Acute post-streptococcal glomerulonephritis in children of French Polynesia: a 3-year retrospective study . Pediatr Nephrol, 2010,25(2):275-280.

[163] Beermann C, Hartung J. Physiological properties of milk ingredients released by fermentation. Food Funct, 2013,4(2):185-199.

[164] Bégué P, Floret D, Mallet E, et al. Pharmacokinetics and clinical evaluation of cefotaxime in children suffering with purulent meningitis. J Antimicrob Chemother, 1984,14 Suppl B:161-165.

[165] Bejjanki H, Koratala A. Rothia mucilaginosa bacteremia in end-stage renal disease and solid organ transplant: the need for raised awareness. Infez Med, 2019,27(1):82-84.

[166] Bélanger V, Benmoussa A, Napartuk M, et al. The Role of Oxidative Stress and Inflammation in Cardiometabolic Health of Children During Cancer Treatment and Potential Impact of Key Nutrients. Antioxid Redox Signal, 2021,35(4):293-318.

[167] Belei O, Olariu L, Dobrescu A, et al. The relationship between non-alcoholic fatty liver disease and small intestinal bacterial overgrowth among overweight and obese children and adolescents. J Pediatr Endocrinol Metab, 2017,30(11):1161-1168.

[168] Beltrán-Barrientos L M, García H S, Hernández-Mendoza A, et al. Invited review: Effect of antihypertensive fermented milks on gut microbiota. J Dairy Sci, 2021,104(4):3779-3788.

[169] Beltrán-Barrientos L M, Hernández-Mendoza A, Torres-Llanez M J, et al. Invited review: Fermented milk as antihypertensive functional food. J Dairy Sci, 2016,99(6):4099-4110.

[170] Belz S, Nau H. Determination of folate patterns in mouse plasma, erythrocytes, and embryos by HPLC coupled with a microbiological assay. Anal Biochem, 1998,265(1):157-166.

[171] Bengmark S. Gut microbiota, immune development and function. Pharmacol Res, 2013,69(1):87-113.

[172] Bengmark S. Obesity, the deadly quartet and the contribution of the neglected daily organ rest - a new dimension of un-health and its prevention. Hepatobiliary Surg Nutr, 2015,4(4):278-288.

[173] Benhayoun M, Llor J, Van-Den-Abbeele T, et al. [Bilateral jugular thrombosis in Lemierre syndrome]. Arch Pediatr, 2003,10(12):1071-1074.

[174] Bennet L, Cowie R V, Stone P R, et al. The neural and vascular effects of killed Su-Streptococcus pyogenes (OK-432) in preterm fetal sheep. Am J Physiol Regul Integr Comp Physiol, 2010,299(2):R664-R672.

[175] Bentubo H D, Mantovani A, Yamashita J T, et al. Yeasts of the genital region of patients attending the dermatology service at Hospital São Paulo, Brazil . Rev Iberoam Micol, 2015,32(4):229-234.

[176] Bergdahl U, Berge T, Johansson S. Pulmonary haemosiderosis and glomerulonephritis. Acta Med Scand, 1969,186(3):199-207.

[177] Berger J I, Gibson R L, Clarke W R, et al. Effect of amrinone during group B Streptococcus-induced pulmonary hypertension in piglets. Pediatr Pulmonol, 1993,16(5):303-310.

[178] Berger J I, Gibson R L, Redding G J, et al. Effect of inhaled nitric oxide during group B streptococcal sepsis in piglets. Am Rev Respir Dis, 1993,147(5):1080-1086.

[179] BERGMANN F, REITLER R, CHAIMOVITZ M, et al. Pharmacological study of a new antibiotic of bacillary origin. Br J Pharmacol Chemother, 1960,15(2):313-318.

[180] Berkowitz E, Kopelman Y, Kadosh D, et al. "More Guts Than Brains?"-The Role of Gut Microbiota in Idiopathic Intracranial Hypertension . J Neuroophthalmol, 2021.

[181] Bernal-Pacheco O, Román G C. Environmental vascular risk factors: new perspectives for stroke prevention . J Neurol Sci, 2007,262(1-2):60-70.

[182] Bernard B K, Nakamura Y, Bando I, et al. Studies of the toxicological potential of tripeptides (L-valyl-L-prolyl-L-proline and L-isoleucyl-L-prolyI-L-proline): II. Introduction. Int J Toxicol, 2005,24 Suppl 4:5-11.

[183] Bernardi M, Moreau R, Angeli P, et al. Mechanisms of decompensation and organ failure in cirrhosis: From peripheral arterial vasodilation to systemic inflammation hypothesis . J Hepatol, 2015,63(5):1272-1284.

[184] Bescos R, Ashworth A, Cutler C, et al. Effects of Chlorhexidine mouthwash on the oral microbiome. Sci Rep, 2020,10(1):5254.

[185] Beyi A F, Mochel J P, Magnin G, et al. Comparisons of plasma and fecal pharmacokinetics of danofloxacin and enrofloxacin in healthy and Mannheimia haemolytica infected calves. Sci Rep, 2022,12(1):5107.

[186] Bezek K, Petelin A, Pražnikar J, et al. Obesity Measures and Dietary Parameters as Predictors of Gut Microbiota Phyla in Healthy Individuals. Nutrients, 2020,12(9).

[187] Bhalla K, Gupta A, Nanda S, et al. Epidemiology and clinical outcomes of acute glomerulonephritis in a teaching hospital in North India. J Family Med Prim Care, 2019,8(3):934-937.

[188] Bhat M, Arendt B M, Bhat V, et al. Implication of the intestinal microbiome in complications of cirrhosis. World J Hepatol, 2016,8(27):1128-1136.

[189] Bier A, Braun T, Khasbab R, et al. A High Salt Diet Modulates the Gut Microbiota and Short Chain Fatty Acids Production in a Salt-Sensitive Hypertension Rat Model. Nutrients, 2018,10(9).

[190] Bier D M. Growth in the first two years of life. Nestle Nutr Workshop Ser Pediatr Program, 2008,61:135-144.

[191] Biernat M M, Urbaniak-Kujda D, Dybko J, et al. Fecal microbiota transplantation in the treatment of intestinal steroid-resistant graft-versus-host disease: two case reports and a review of the literature. J Int Med Res, 2020,48(6):1220725245.

[192] Birlutiu V, Birlutiu R M, Costache V S. Viridans streptococcal infective endocarditis associated with fixed orthodontic appliance managed surgically by mitral valve plasty: A case report. Medicine (Baltimore), 2018,97(27):e11260.

[193] Biscetti F, Nardella E, Cecchini A L, et al. The Role of the Microbiota in the Diabetic Peripheral Artery Disease. Mediators Inflamm, 2019,2019:4128682.

[194] Bitar N, Claes R, Van der Auwera P. Concentrations of ofloxacin in serum and cerebrospinal fluid of patients without meningitis receiving the drug intravenously and orally. Antimicrob Agents Chemother, 1989,33(10):1686-1690.

[195] BJORNEBOE M, SCHWARTZ M. Investigations concerning the changes in serum proteins during immunization; the cause of hypoalbuminemia with high gamma globulin values. J Exp Med, 1959,110(2):259-270.

[196] Bjørnshave A, Hermansen K. Effects of dairy protein and fat on the metabolic syndrome and type 2 diabetes. Rev Diabet Stud, 2014,11(2):153-166.

[197] BLACKWELL B, MABBITT L A. TYRAMINE IN CHEESE RELATED TO HYPERTENSIVE CRISES AFTER MONOAMINE-OXIDASE INHIBITION . Lancet, 1965,1(7392):938-940.

[198] Bland R D. Edema formation in the lungs and its relationship to neonatal respiratory distress. Acta Paediatr Scand Suppl, 1983,305:92-99.

[199] Bland R D. Edema formation in the newborn lung. Clin Perinatol, 1982,9(3):593-611.

[200] Blaženović I, Oh Y T, Li F, et al. Effects of Gut Bacteria Depletion and High-Na(+) and Low-K(+) Intake on Circulating Levels of Biogenic Amines. Mol Nutr Food Res, 2019,63(4):e1801184.

[201] Blekkenhorst L C, Bondonno N P, Liu A H, et al. Nitrate, the oral microbiome, and cardiovascular health: a systematic literature review of human and animal studies. Am J Clin Nutr, 2018,107(4):504-522.

[202] Blesa E, Aliño M, Barat J M, et al. Microbiology and physico-chemical changes of dry-cured ham during the post-salting stage as affected by partial replacement of NaCl by other salts. Meat Sci, 2008,78(1-2):135-142.

[203] Block G, Jensen C D, Norkus E P, et al. Usage patterns, health, and nutritional status of long-term multiple dietary supplement users: a cross-sectional study . Nutr J, 2007,6:30.

[204] Bloom S L, Cox S M, Bawdon R E, et al. Ampicillin for neonatal group B streptococcal prophylaxis: how rapidly can bactericidal concentrations be achieved? Am J Obstet Gynecol, 1996,175(4 Pt 1):974-976.

[205] Bloomgarden Z. Diabetes and branched-chain amino acids: What is the link? J Diabetes, 2018,10(5):350-352.

[206] Blumberg J B, Basu A, Krueger C G, et al. Impact of Cranberries on Gut Microbiota and Cardiometabolic Health: Proceedings of the Cranberry Health Research Conference 2015 . Adv Nutr, 2016,7(4):759S-770S.

[207] Blyth C C, Robertson P W, Rosenberg A R. Post-streptococcal glomerulonephritis in Sydney: a 16-year retrospective review. J Paediatr Child Health, 2007,43(6):446-450.

[208] Bødker B, Hvidman L, Weber T, et al. Maternal deaths in Denmark 2002-2006. Acta Obstet Gynecol Scand, 2009,88(5):556-562.

[209] Boillot A, Demmer R T, Mallat Z, et al. Periodontal microbiota and phospholipases: the Oral Infections and Vascular Disease Epidemiology Study (INVEST). Atherosclerosis, 2015,242(2):418-423.

[210] Boixeda R, Almagro P, Díez-Manglano J, et al. Bacterial flora in the sputum and comorbidity in patients with acute exacerbations of COPD. Int J Chron Obstruct Pulmon Dis, 2015,10:2581-2591.

[211] Bondonno C P, Liu A H, Croft K D, et al. Antibacterial mouthwash blunts oral nitrate reduction and increases blood pressure in treated hypertensive men and women. Am J Hypertens, 2015,28(5):572-575.

[212] Borghi C, Strocchi E. [Heart-gut interactions] . G Ital Cardiol (Rome), 2018,19(4):203-208.

[213] Borrelli A, Bonelli P, Tuccillo F M, et al. Role of gut microbiota and oxidative stress in the progression of non-alcoholic fatty liver disease to hepatocarcinoma: Current and innovative therapeutic approaches . Redox Biol, 2018,15:467-479.

[214] Boshuizen M, van Bruggen R, Zaat S A, et al. Development of a model for anemia of inflammation that is relevant to critical care. Intensive Care Med Exp, 2019,7(Suppl 1):47.

[215] Bottoli I, Beharry K, Modanlou H D, et al. Effect of group B streptococcal meningitis on retinal and choroidal blood flow in newborn pigs. Invest Ophthalmol Vis Sci, 1995,36(7):1231-1239.

[216] Boucher J H, Hilmas D E, Liu C T, et al. Myocardial depression during Diplococcus pneumoniae infection in monkeys. Proc Soc Exp Biol Med, 1974,145(1):112-116.

[217] Bouglé D, Bouhallab S. Dietary bioactive peptides: Human studies. Crit Rev Food Sci Nutr, 2017,57(2):335-343.

[218] Boukobza M, Duval X, Laissy J P. Mycotic intracranial aneurysms rupture presenting as pure acute subdural hematoma in infectious endocarditis. Report of 2 cases and review of the literature. J Clin Neurosci, 2019,62:222-225.

[219] Boulton A A, Cookson B, Paulton R. Hypertensive crisis in a patient on MAOI antidepressants following a meal of beef liver . Can Med Assoc J, 1970,102(13):1394-1395.

[220] Bouskraoui M, Benbachir M, Abid A. [Streptococcus uberis endocarditis in an infant with atrioventricular defect]. Arch Pediatr, 1999,6(4):481.

[221] Boutin S, Hildebrand D, Boulant S, et al. Host factors facilitating SARS-CoV-2 virus infection and replication in the lungs. Cell Mol Life Sci, 2021,78(16):5953-5976.

[222] Bowdy B D, Aziz S M, Marple S L, et al. Organ-specific disposition of group B streptococci in piglets: evidence for a direct interaction with target cells in the pulmonary circulation. Pediatr Res, 1990,27(4 Pt 1):344-348.

[223] Bowdy B D, Marple S L, Pauly T H, et al. Oxygen radical-dependent bacterial killing and pulmonary hypertension in piglets infected with group B streptococci. Am Rev Respir Dis, 1990,141(3):648-653.

[224] Boyer O, Baudouin V, Bérard E, et al. [Idiopathic nephrotic syndrome]. Arch Pediatr, 2017,24(12):1338-1343.

[225] Braeken D C, Franssen F M, von Baum H, et al. Bacterial aetiology and mortality in COPD patients with CAP: results from the German Competence Network, CAPNETZ . Int J Tuberc Lung Dis, 2017,21(2):236-243.

[226] Brandão I, Martins M J, Monteiro R. Metabolically Healthy Obesity-Heterogeneity in Definitions and Unconventional Factors. Metabolites, 2020,10(2).

[227] Brandt C T, Maciel D T, Caneca O A, et al. Autotransplant of spleen tissue in children with schistosomiasis: evaluation of splenic function after splenosis . Mem Inst Oswaldo Cruz, 2001,96 Suppl:117-122.

[228] Brantsaeter A L, Myhre R, Haugen M, et al. Intake of probiotic food and risk of preeclampsia in primiparous women: the Norwegian Mother and Child Cohort Study . Am J Epidemiol, 2011,174(7):807-815.

[229] Bräutigam H H, Knothe H, Rangoonwala R. Impact of cefotaxime and ceftriaxone on the bowel and vaginal flora after single-dose prophylaxis in vaginal hysterectomy. Drugs, 1988,35 Suppl 2:163-168.

[230] Bravo M, Combes T, Martinez F O, et al. Lactobacilli Isolated From Wild Boar (Sus scrofa) Antagonize Mycobacterium bovis Bacille Calmette-Guerin (BCG) in a Species-Dependent Manner. Front Microbiol, 2019,10:1663.

[231] Bressack M A, Morton N S, Hortop J. Group B streptococcal sepsis in the piglet: effects of fluid therapy on venous return, organ edema, and organ blood flow. Circ Res, 1987,61(5):659-669.

[232] Breuninger T A, Wawro N, Breuninger J, et al. Associations between habitual diet, metabolic disease, and the gut microbiota using latent Dirichlet allocation. Microbiome, 2021,9(1):61.

[233] Brocker C N, Velenosi T, Flaten H K, et al. Metabolomic profiling of metoprolol hypertension treatment reveals altered gut microbiota-derived urinary metabolites. Hum Genomics, 2020,14(1):10.

[234] Bronzato S, Durante A. Dietary Supplements and Cardiovascular Diseases. Int J Prev Med, 2018,9:80.

[235] Brosa M H, Subirà O, Gomà G M, et al. Ulcerative granuloma of the eyelid as the initial manifestation of granulomatosis with polyangiitis (Wegener's granulomatosis): A case report . Orbit, 2017,36(4):243-246.

[236] Brouwer-Brolsma E M, van Woudenbergh G J, Oude E S, et al. Intake of different types of dairy and its prospective association with risk of type 2 diabetes: The Rotterdam Study . Nutr Metab Cardiovasc Dis, 2016,26(11):987-995.

[237] Brown J H, Orcutt M L. A STUDY OF BACILLUS PYOGENES . J Exp Med, 1920,32(2):219-248.

[238] Brown J S, Gilliland S M, Basavanna S, et al. phgABC, a three-gene operon required for growth of Streptococcus pneumoniae in hyperosmotic medium and in vivo . Infect Immun, 2004,72(8):4579-4588.

[239] Brown J, Yazdi F, Jodari-Karimi M, et al. Obstructive Sleep Apnea and Hypertension: Updates to a Critical Relationship. Curr Hypertens Rep, 2022:1-12.

[240] Brown R F, Kinnick M D, Morin J J, et al. Synthesis and biological evaluation of a series of parenteral 3'-quaternary ammonium cephalosporins . J Med Chem, 1990,33(8):2114-2121.

[241] Browne D T, Aguilo-Seara G, DeFranzo A J. The Black Locust Tree: Toxalbumin-Induced Tissue Necrosis of the Upper Extremity. Cureus, 2020,12(11):e11758.

[242] Browning M B, Dempsey D, Guiza V, et al. Multilayer vascular grafts based on collagen-mimetic proteins. Acta Biomater, 2012,8(3):1010-1021.

[243] Bruning J, Chapp A, Kaurala G A, et al. Gut Microbiota and Short Chain Fatty Acids: Influence on the Autonomic Nervous System. Neurosci Bull, 2020,36(1):91-95.

[244] Brunser O, Gotteland M, Cruchet S. Functional fermented milk products. Nestle Nutr Workshop Ser Pediatr Program, 2007,60:235-250.

[245] Bryan N S. Functional Nitric Oxide Nutrition to Combat Cardiovascular Disease. Curr Atheroscler Rep, 2018,20(5):21.

[246] Bryan N S, Tribble G, Angelov N. Oral Microbiome and Nitric Oxide: the Missing Link in the Management of Blood Pressure. Curr Hypertens Rep, 2017,19(4):33.

[247] Bs S, Thankappan B, Mahendran R, et al. Evaluation of GABA Production and Probiotic Activities of Enterococcus faecium BS5. Probiotics Antimicrob Proteins, 2021,13(4):993-1004.

[248] Bu J, Wang Z. Cross-Talk between Gut Microbiota and Heart via the Routes of Metabolite and Immunity. Gastroenterol Res Pract, 2018,2018:6458094.

[249] Bubalo J S, Munar M Y, Cherala G, et al. Daptomycin pharmacokinetics in adult oncology patients with neutropenic fever. Antimicrob Agents Chemother, 2009,53(2):428-434.

[250] Buendia J R, Li Y, Hu F B, et al. Regular Yogurt Intake and Risk of Cardiovascular Disease Among Hypertensive Adults. Am J Hypertens, 2018,31(5):557-565.

[251] Buerger A N, Dillon D T, Schmidt J, et al. Gastrointestinal dysbiosis following diethylhexyl phthalate exposure in zebrafish (Danio rerio): Altered microbial diversity, functionality, and network connectivity. Environ Pollut, 2020,265(Pt B):114496.

[252] Bührer C, Merker G, Falke K, et al. Dose-response to inhaled nitric oxide in acute hypoxemic respiratory failure of newborn infants: a preliminary report . Pediatr Pulmonol, 1995,19(5):291-298.

[253] Burleigh M C, Liddle L, Monaghan C, et al. Salivary nitrite production is elevated in individuals with a higher abundance of oral nitrate-reducing bacteria . Free Radic Biol Med, 2018,120:80-88.

[254] Burleigh M, Liddle L, Muggeridge D J, et al. Dietary nitrate supplementation alters the oral microbiome but does not improve the vascular responses to an acute nitrate dose. Nitric Oxide, 2019,89:54-63.

[255] Burnham P, Dadhania D, Heyang M, et al. Urinary cell-free DNA is a versatile analyte for monitoring infections of the urinary tract . Nat Commun, 2018,9(1):2412.

[256] Bütikofer U, Meyer J, Sieber R, et al. Occurrence of the angiotensin-converting enzyme inhibiting tripeptides Val-Pro-Pro and Ile-Pro-Pro in different cheese varieties of Swiss origin. J Dairy Sci, 2008,91(1):29-38.

[257] Cabrera C, Vicens P, Torrente M. Modifiable Risk Factors for Dementia: The Role of Gut Microbiota. Curr Alzheimer Res, 2021,18(13):993-1009.

[258] Caffarelli C, Santamaria F, Vottero A, et al. Progress in pediatrics in 2013: choices in allergology, endocrinology, gastroenterology, hypertension, infectious diseases, neonatology, neurology, nutrition and respiratory tract illnesses . Ital J Pediatr, 2014,40:62.

[259] Cai T T, Ye X L, Yong H J, et al. Fecal microbiota transplantation relieve painful diabetic neuropathy: A case report. Medicine (Baltimore), 2018,97(50):e13543.

[260] Cai Y, Juszczak H M, Cope E K, et al. The microbiome in obstructive sleep apnea. Sleep, 2021,44(8).

[261] Cakir M, Cekiç O, Pekel G, et al. Pars plana vitrectomy results of exogenous endophthalmitis in children . Eur J Ophthalmol, 2010,20(2):424-428.

[262] Calderón-Pérez L, Gosalbes M J, Yuste S, et al. Gut metagenomic and short chain fatty acids signature in hypertension: a cross-sectional study . Sci Rep, 2020,10(1):6436.

[263] Calderón-Pérez L, Llauradó E, Companys J, et al. Interplay between dietary phenolic compound intake and the human gut microbiome in hypertension: A cross-sectional study . Food Chem, 2021,344:128567.

[264] Callejo M, Barberá J A, Duarte J, et al. Impact of Nutrition on Pulmonary Arterial Hypertension. Nutrients, 2020,12(1).

[265] Callejo M, Mondejar-Parreño G, Barreira B, et al. Pulmonary Arterial Hypertension Affects the Rat Gut Microbiome. Sci Rep, 2018,8(1):9681.

[266] Camacho F, Macedo A, Malcata F. Potential Industrial Applications and Commercialization of Microalgae in the Functional Food and Feed Industries: A Short Review. Mar Drugs, 2019,17(6).

[267] Campbell P T, Tong S, Geard N, et al. Longitudinal Analysis of Group A Streptococcus emm Types and emm Clusters in a High-Prevalence Setting: Relationship between Past and Future Infections. J Infect Dis, 2020,221(9):1429-1437.

[268] Canale M P, Noce A, Di Lauro M, et al. Gut Dysbiosis and Western Diet in the Pathogenesis of Essential Arterial Hypertension: A Narrative Review. Nutrients, 2021,13(4).

[269] Canani R B, Costanzo M D, Leone L, et al. Epigenetic mechanisms elicited by nutrition in early life. Nutr Res Rev, 2011,24(2):198-205.

[270] Cano S J, Mestra C F, Nieto J, et al. Teeth infection may "shunt" through Fontan in high-altitude conditions . Ann Transl Med, 2018,6(7):118.

[271] Cao R Y, Zheng Y, Zhang Y, et al. Berberine on the Prevention and Management of Cardiometabolic Disease: Clinical Applications and Mechanisms of Action. Am J Chin Med, 2021,49(7):1645-1666.

[272] Capurso L. Thirty Years of Lactobacillus rhamnosus GG: A Review. J Clin Gastroenterol, 2019,53 Suppl 1:S1-S41.

[273] Caracciolo B, Xu W, Collins S, et al. Cognitive decline, dietary factors and gut-brain interactions . Mech Ageing Dev, 2014,136-137:59-69.

[274] Caraceni P, Vargas V, Solà E, et al. The Use of Rifaximin in Patients With Cirrhosis. Hepatology, 2021,74(3):1660-1673.

[275] Carbone A, Lieu A, Mouhat B, et al. Spondylodiscitis complicating infective endocarditis. Heart, 2020,106(24):1914-1918.

[276] Carceller L F, de la Torre E M, Porto A R, et al. Acute glomerulonephritis associated with pneumonia: a review of three cases . Pediatr Nephrol, 2010,25(1):161-164.

[277] Carnevale R, Raparelli V, Nocella C, et al. Gut-derived endotoxin stimulates factor VIII secretion from endothelial cells. Implications for hypercoagulability in cirrhosis. J Hepatol, 2017,67(5):950-956.

[278] Carpenter D, Larkin H, Chang A, et al. Superoxide dismutase and catalase do not affect the pulmonary hypertensive response to group B streptococcus in the lamb. Pediatr Res, 2001,49(2):181-188.

[279] Carrión V F, Bertomeu G V. [Lung toxicity due to thalidomide]. Arch Bronconeumol, 2002,38(10):492-494.

[280] Carsenti-Etesse H, Farinotti R, Durant J, et al. Pharmacokinetic parameters and killing rates in serum of volunteers receiving amoxicillin, cefadroxil or cefixime alone or associated with niflumic acid or paracetamol . Eur J Drug Metab Pharmacokinet, 1998,23(3):357-366.

[281] Carter C S, Morgan D, Verma A, et al. Therapeutic Delivery of Ang(1-7) via Genetically Modified Probiotic: A Dosing Study. J Gerontol A Biol Sci Med Sci, 2020,75(7):1299-1303.

[282] Cason C A, Dolan K T, Sharma G, et al. Plasma microbiome-modulated indole- and phenyl-derived metabolites associate with advanced atherosclerosis and postoperative outcomes. J Vasc Surg, 2018,68(5):1552-1562.

[283] Castellani C, Singer G, Kaiser M, et al. Neuroblastoma causes alterations of the intestinal microbiome, gut hormones, inflammatory cytokines, and bile acid composition. Pediatr Blood Cancer, 2017,64(8).

[284] Castillo V, Figueroa F, González-Pizarro K, et al. Probiotics and Prebiotics as a Strategy for Non-Alcoholic Fatty Liver Disease, a Narrative Review . Foods, 2021,10(8).

[285] Cataldo P G, Villena J, Elean M, et al. Immunomodulatory Properties of a γ-Aminobutyric Acid-Enriched Strawberry Juice Produced by Levilactobacillus brevis CRL 2013. Front Microbiol, 2020,11:610016.

[286] Cerletti C, Esposito S, Iacoviello L. Edible Mushrooms and Beta-Glucans: Impact on Human Health. Nutrients, 2021,13(7).

[287] Chai H T, Tan B L, Yen H T, et al. Infective endocarditis caused by Streptococcus bovis complicated by a superior mesenteric artery mycotic aneurysm and systemic septic emboli in a patient with colon diverticulitis . Int J Infect Dis, 2010,14 Suppl 3:e317-e318.

[288] Chakraborty S, Mandal J, Cheng X, et al. Diurnal Timing Dependent Alterations in Gut Microbial Composition Are Synchronously Linked to Salt-Sensitive Hypertension and Renal Damage . Hypertension, 2020,76(1):59-72.

[289] Chakraborty S, Mandal J, Yang T, et al. Metabolites and Hypertension: Insights into Hypertension as a Metabolic Disorder: 2019 Harriet Dustan Award . Hypertension, 2020,75(6):1386-1396.

[290] Chalkiadakis G E, Gonnianakis C, Tsatsakis A, et al. Preincisional single-dose ceftriaxone for the prophylaxis of surgical wound infection . Am J Surg, 1995,170(4):353-355.

[291] Chamarthi G, Clapp W L, Bejjanki H, et al. Infection-related Glomerulonephritis and C3 Glomerulonephritis - Similar Yet Dissimilar: A Case Report and Brief Review of Current Literature . Cureus, 2020,12(2):e7127.

[292] Chambers E S, Preston T, Frost G, et al. Role of Gut Microbiota-Generated Short-Chain Fatty Acids in Metabolic and Cardiovascular Health. Curr Nutr Rep, 2018,7(4):198-206.

[293] Champion C J, Xu J. The impact of metagenomic interplay on the mosquito redox homeostasis. Free Radic Biol Med, 2017,105:79-85.

[294] Chan M, Baxter H, Larsen N, et al. Impact of botanical fermented foods on metabolic biomarkers and gut microbiota in adults with metabolic syndrome and type 2 diabetes: a systematic review protocol . BMJ Open, 2019,9(7):e29242.

[295] Chang B J, Park S U, Jang Y S, et al. Effect of functional yogurt NY-YP901 in improving the trait of metabolic syndrome . Eur J Clin Nutr, 2011,65(11):1250-1255.

[296] Chang N N, Murray C K, Houck P M, et al. Blood culture and susceptibility results and allergy history do not influence fluoroquinolone use in the treatment of community-acquired pneumonia . Pharmacotherapy, 2005,25(1):59-66.

[297] Chang V H, Chiu T H, Fu S C. In vitro anti-inflammatory properties of fermented pepino (Solanum muricatum) milk by γ-aminobutyric acid-producing Lactobacillus brevis and an in vivo animal model for evaluating its effects on hypertension . J Sci Food Agric, 2016,96(1):192-198.

[298] Chang Y, Chen Y, Zhou Q, et al. Short-chain fatty acids accompanying changes in the gut microbiome contribute to the development of hypertension in patients with preeclampsia . Clin Sci (Lond), 2020,134(2):289-302.

[299] Chaplin A, Carpéné C, Mercader J. Resveratrol, Metabolic Syndrome, and Gut Microbiota . Nutrients, 2018,10(11).

[300] Charan N B, Mudumbi R V, Hawk P, et al. Streptococcus pneumoniae-induced pulmonary hypertension and systemic hypotension in anesthetized sheep . J Appl Physiol (1985), 1994,77(5):2071-2078.

[301] Charitos I A, Topi S, Castellaneta F, et al. Current Issues and Perspectives in Patients with Possible Sepsis at Emergency Departments . Antibiotics (Basel), 2019,8(2).

[302] Charlton K E, Steyn K, Levitt N S, et al. A food-based dietary strategy lowers blood pressure in a low socio-economic setting: a randomised study in South Africa. Public Health Nutr, 2008,11(12):1397-1406.

[303] Chaudhari S N, McCurry M D, Devlin A S. Chains of evidence from correlations to causal molecules in microbiome-linked diseases . Nat Chem Biol, 2021,17(10):1046-1056.

[304] Chaudhuri K, Gonzales J, Jesurun C A, et al. Anaphylactic shock in pregnancy: a case study and review of the literature. Int J Obstet Anesth, 2008,17(4):350-357.

[305] Chelsom J, Halstensen A, Haga T, et al. Necrotising fasciitis due to group A streptococci in western Norway: incidence and clinical features . Lancet, 1994,344(8930):1111-1115.

[306] Chen C H, Lin C L, Kao C H. Irritable Bowel Syndrome Is Associated with an Increased Risk of Dementia: A Nationwide Population-Based Study . PLoS One, 2016,11(1):e144589.

[307] Chen H E, Lin Y J, Lin I C, et al. Resveratrol prevents combined prenatal N(G)-nitro-L-arginine-methyl ester (L-NAME) treatment plus postnatal high-fat diet induced programmed hypertension in adult rat offspring: interplay between nutrient-sensing signals, oxidative stress and gut microbiota . J Nutr Biochem, 2019,70:28-37.

[308] Chen H Q, Gong J Y, Xing K, et al. Pharmacomicrobiomics: Exploiting the Drug-Microbiota Interactions in Antihypertensive Treatment. Front Med (Lausanne), 2021,8:742394.

[309] Chen H, Zhao M, Li J. [Effects of Streptococcus sanguis on blood composition, blood pressure and cardiac dysfunction in rabbis] . Zhonghua Kou Qiang Yi Xue Za Zhi, 2001,36(5):354-356.

[310] Chen J J, Wang R, Li X F, et al. Bifidobacterium longum supplementation improved high-fat-fed-induced metabolic syndrome and promoted intestinal Reg I gene expression . Exp Biol Med (Maywood), 2011,236(7):823-831.

[311] Chen J, Qin Q, Yan S, et al. Gut Microbiome Alterations in Patients With Carotid Atherosclerosis. Front Cardiovasc Med, 2021,8:739093.

[312] Chen L, Alcazar J, Yang T, et al. Optimized cultural conditions of functional yogurt for γ-aminobutyric acid augmentation using response surface methodology . J Dairy Sci, 2018,101(12):10685-10693.

[313] Chen L, Bai J, Peng D, et al. SZB120 Exhibits Immunomodulatory Effects by Targeting eIF2α to Suppress Th17 Cell Differentiation. J Immunol, 2021,206(5):953-962.

[314] Chen L, Chen D Q, Liu J R, et al. Unilateral ureteral obstruction causes gut microbial dysbiosis and metabolome disorders contributing to tubulointerstitial fibrosis . Exp Mol Med, 2019,51(3):1-18.

[315] Chen L, Wang L, Li J, et al. Antihypertensive potential of fermented milk: the contribution of lactic acid bacteria proteolysis system and the resultant angiotensin-converting enzyme inhibitory peptide . Food Funct, 2021,12(22):11121-11131.

[316] Chen S Y, Wu C Y, Tsai I J, et al. Nonenteropathic hemolytic uremic syndrome: the experience of a medical center. Pediatr Neonatol, 2011,52(2):73-77.

[317] Chen W Y, Liang G Y, Zheng Z L, et al. UPLC-ESI-Q-TOF/MS Based Metabolic Profiling of Protosappanin B in Rat Plasma, Bile, Feces, Urine and Intestinal Bacteria Samples . Curr Drug Metab, 2021,22(6):491-499.

[318] Chen X, Eslamfam S, Fang L, et al. Maintenance of Gastrointestinal Glucose Homeostasis by the Gut-Brain Axis. Curr Protein Pept Sci, 2017,18(6):541-547.

[319] Chen X, Li P, Liu M, et al. Gut dysbiosis induces the development of pre-eclampsia through bacterial translocation . Gut, 2020,69(3):513-522.

[320] Chen Y F, Zhao W J, Wu R N, et al. Proteome analysis of Lactobacillus helveticus H9 during growth in skim milk . J Dairy Sci, 2014,97(12):7413-7425.

[321] Chen Y M, Liu Y, Zhou R F, et al. Associations of gut-flora-dependent metabolite trimethylamine-N-oxide, betaine and choline with non-alcoholic fatty liver disease in adults . Sci Rep, 2016,6:19076.

[322] Chen Y R, Zheng H M, Zhang G X, et al. High Oscillospira abundance indicates constipation and low BMI in the Guangdong Gut Microbiome Project . Sci Rep, 2020,10(1):9364.

[323] Chen Y, Chen M, Zhang Y, et al. Broad-Spectrum Neutralization of Pore-Forming Toxins with Human Erythrocyte Membrane-Coated Nanosponges . Adv Healthc Mater, 2018,7(13):e1701366.

[324] Chen Y, Li C, Xue J, et al. Characterization of angiotensin-converting enzyme inhibitory activity of fermented milk produced by Lactobacillus helveticus . J Dairy Sci, 2015,98(8):5113-5124.

[325] Chen Y, Luo L, Hu S, et al. The chemistry, processing, and preclinical anti-hyperuricemia potential of tea: a comprehensive review . Crit Rev Food Sci Nutr, 2022:1-26.

[326] Chen Z Y, Peng C, Jiao R, et al. Anti-hypertensive nutraceuticals and functional foods. J Agric Food Chem, 2009,57(11):4485-4499.

[327] Chen Z, Wu C, Cao X, et al. Risk factors for neonatal group B streptococcus vertical transmission: a prospective cohort study of 1815 mother-baby pairs . J Perinatol, 2018,38(10):1309-1317.

[328] Cheng M C, Pan T M. Prevention of hypertension-induced vascular dementia by Lactobacillus paracasei subsp. paracasei NTU 101-fermented products . Pharm Biol, 2017,55(1):487-496.

[329] Chi C, Li C, Wu D, et al. Effects of Probiotics on Patients with Hypertension: a Systematic Review and Meta-Analysis . Curr Hypertens Rep, 2020,22(5):34.

[330] Chia J S, Chang L Y, Shun C T, et al. A 60-kilodalton immunodominant glycoprotein is essential for cell wall integrity and the maintenance of cell shape in Streptococcus mutans . Infect Immun, 2001,69(11):6987-6998.

[331] Chiang S S, Pan T M. Beneficial effects of Lactobacillus paracasei subsp. paracasei NTU 101 and its fermented products . Appl Microbiol Biotechnol, 2012,93(3):903-916.

[332] Chiaw T H, San T R, Le TJ. An adult with truncus arteriosus and unilateral pulmonary hypertension. Congenit Heart Dis, 2007,2(6):433-437.

[333] Chihara D, Sakamoto T, Murakami G, et al. [An overwhelming post-splenectomy infection with toxic shock syndrome by group B Streptococcus] . Rinsho Ketsueki, 2010,51(4):253-257.

[334] Chiu H H, Tsai I L, Lu Y S, et al. Development of an LC-MS/MS method with protein G purification strategy for quantifying bevacizumab in human plasma . Anal Bioanal Chem, 2017,409(28):6583-6593.

[335] Choi M S, Yu J S, Yoo H H, et al. The role of gut microbiota in the pharmacokinetics of antihypertensive drugs. Pharmacol Res, 2018,130:164-171.

[336] Choi Y, Bose S, Shin N R, et al. Lactate-Fortified Puerariae Radix Fermented by Bifidobacterium breve Improved Diet-Induced Metabolic Dysregulation via Alteration of Gut Microbial Communities . Nutrients, 2020,12(2).

[337] Chomarat M, Panteix G, Guillaumond B, et al. Tonsillar diffusion kinetics of amoxycillin after oral administration of 1 g to adults . Eur J Drug Metab Pharmacokinet, 1997,22(2):141-144.

[338] Chong N C, Duboc D, Rainteau D, et al. Circulating bile acids concentration is predictive of coronary artery disease in human . Sci Rep, 2021,11(1):22661.

[339] Chorney S R, Buzi A, Rizzi M D. The Role of Endoscopic Sinus Surgery in Children Undergoing External Drainage of Non-Medial Subperiosteal Orbital Abscess . Am J Rhinol Allergy, 2021,35(3):288-295.

[340] Chou S H, Prabhu S J, Crothers K, et al. Thoracic diseases associated with HIV infection in the era of antiretroviral therapy: clinical and imaging findings . Radiographics, 2014,34(4):895-911.

[341] Christensen K L, Hedemann M S, Jørgensen H, et al. Liquid chromatography-mass spectrometry based metabolomics study of cloned versus normal pigs fed either restricted or ad libitum high-energy diets . J Proteome Res, 2012,11(7):3573-3580.

[342] Christofides E A. POINT: Artificial Sweeteners and Obesity-Not the Solution and Potentially a Problem. Endocr Pract, 2021,27(10):1052-1055.

[343] Chu C Q, Yu L L, Qi G Y, et al. Can dietary patterns prevent cognitive impairment and reduce Alzheimer's disease risk: Exploring the underlying mechanisms of effects. Neurosci Biobehav Rev, 2022,135:104556.

[344] Cicero A F, Colletti A. Nutraceuticals and Blood Pressure Control: Results from Clinical Trials and Meta-Analyses. High Blood Press Cardiovasc Prev, 2015,22(3):203-213.

[345] Cicero A, Colletti A. Nutraceuticals and Dietary Supplements to Improve Quality of Life and Outcomes in Heart Failure Patients. Curr Pharm Des, 2017,23(8):1265-1272.

[346] Cicero A, Colletti A, Bajraktari G, et al. Lipid-lowering nutraceuticals in clinical practice: position paper from an International Lipid Expert Panel . Nutr Rev, 2017,75(9):731-767.

[347] Cicero A, Colletti A, von Haehling S, et al. Nutraceutical support in heart failure: a position paper of the International Lipid Expert Panel (ILEP) . Nutr Res Rev, 2020,33(1):155-179.

[348] Cillóniz C, Ewig S, Polverino E, et al. Microbial aetiology of community-acquired pneumonia and its relation to severity . Thorax, 2011,66(4):340-346.

[349] Ciornei R T. Prevention of Severe Coronavirus Disease 2019 Outcomes by Reducing Low-Grade Inflammation in High-Risk Categories. Front Immunol, 2020,11:1762.

[350] Clair C, Augsburger A, Birrer P, et al. Assessing the efficacy and impact of a personalised smoking cessation intervention among type 2 diabetic smokers: study protocol for an open-label randomised controlled trial (DISCGO-RCT) . BMJ Open, 2020,10(11):e40117.

[351] Clarke K A, Dew T P, Watson R E, et al. Green tea catechins and their metabolites in human skin before and after exposure to ultraviolet radiation . J Nutr Biochem, 2016,27:203-210.

[352] Clary E M, Bruch S M, Lau C L, et al. Effects of pneumoperitoneum on hemodynamic and systemic immunologic responses to peritonitis in pigs . J Surg Res, 2002,108(1):32-38.

[353] Claus S P, Ellero S L, Berger B, et al. Colonization-induced host-gut microbial metabolic interaction . mBio, 2011,2(2):e210-e271.

[354] Clemente M G, Mandato C, Poeta M, et al. Pediatric non-alcoholic fatty liver disease: Recent solutions, unresolved issues, and future research directions . World J Gastroenterol, 2016,22(36):8078-8093.

[355] Cloetens L, Ulmius M, Johansson-Persson A, et al. Role of dietary beta-glucans in the prevention of the metabolic syndrome. Nutr Rev, 2012,70(8):444-458.

[356] Clos-Garcia M, Andrés-Marin N, Fernández-Eulate G, et al. Gut microbiome and serum metabolome analyses identify molecular biomarkers and altered glutamate metabolism in fibromyalgia . EBioMedicine, 2019,46:499-511.

[357] Clouse K, Shehabi A, Suleimat A M, et al. High prevalence of Group B Streptococcus colonization among pregnant women in Amman, Jordan . BMC Pregnancy Childbirth, 2019,19(1):177.

[358] Coates A M, Hill A M, Tan S Y. Nuts and Cardiovascular Disease Prevention . Curr Atheroscler Rep, 2018,20(10):48.

[359] Cohnen M, Lüthen R, Däubener W, et al. Lack of portosystemic bacterial translocation in patients with liver cirrhosis after placement of transjugular shunt . Eur J Clin Microbiol Infect Dis, 2003,22(5):310-312.

[360] Coimbra R S, Loquet G, Leib S L. Limited efficacy of adjuvant therapy with dexamethasone in preventing hearing loss due to experimental pneumococcal meningitis in the infant rat . Pediatr Res, 2007,62(3):291-294.

[361] Colaco N A, Wang T S, Ma Y, et al. Transmethylamine-N-Oxide Is Associated With Diffuse Cardiac Fibrosis in People Living With HIV . J Am Heart Assoc, 2021,10(16):e20499.

[362] Colás-Tomás T, Pérez-Trigo S. Delayed-onset Endophthalmitis Following Implantation of a XEN45 Glaucoma Device: A Case Report. J Glaucoma, 2018,27(10):936-938.

[363] Cold F, Svensson C K, Petersen A M, et al. Long-Term Safety Following Faecal Microbiota Transplantation as a Treatment for Recurrent Clostridioides difficile Infection Compared with Patients Treated with a Fixed Bacterial Mixture: Results from a Retrospective Cohort Study . Cells, 2022,11(3).

[364] Coleman M, Orvis A, Wu T Y, et al. A Broad Spectrum Chemokine Inhibitor Prevents Preterm Labor but Not Microbial Invasion of the Amniotic Cavity or Neonatal Morbidity in a Non-human Primate Model . Front Immunol, 2020,11:770.

[365] Collet E, Diebold P, Paccaud D, et al. [A 6-week-old infant with failure to thrive: insidious presentation of group B streptococcal ventriculitis] . Arch Pediatr, 2009,16(4):360-363.

[366] Collin H L, Uusitupa M, Niskanen L, et al. Caries in patients with non-insulin-dependent diabetes mellitus . Oral Surg Oral Med Oral Pathol Oral Radiol Endod, 1998,85(6):680-685.

[367] Colombo D F, Lew J L, Pedersen C A, et al. Optimal timing of ampicillin administration to pregnant women for establishing bactericidal levels in the prophylaxis of Group B Streptococcus . Am J Obstet Gynecol, 2006,194(2):466-470.

[368] Companys J, Pedret A, Valls R M, et al. Fermented dairy foods rich in probiotics and cardiometabolic risk factors: a narrative review from prospective cohort studies . Crit Rev Food Sci Nutr, 2021,61(12):1966-1975.

[369] Conte J J, Golden J A, Kelly M G, et al. Steady-state serum and intrapulmonary pharmacokinetics and pharmacodynamics of tigecycline . Int J Antimicrob Agents, 2005,25(6):523-529.

[370] Conte J J, Golden J A, McIver M, et al. Intrapulmonary pharmacodynamics of high-dose levofloxacin in subjects with chronic bronchitis or chronic obstructive pulmonary disease . Int J Antimicrob Agents, 2007,30(5):422-427.

[371] Contreras A, Herrera J A, Soto J E, et al. Periodontitis is associated with preeclampsia in pregnant women. J Periodontol, 2006,77(2):182-188.

[372] Cook A L, St C M, Sams R. Use of florfenicol in non-human primates. J Med Primatol, 2004,33(3):127-133.

[373] Cook K L, Chappell M C. Gut dysbiosis and hypertension: is it cause or effect?. J Hypertens, 2021,39(9):1768-1770.

[374] Cookson T A. Bacterial-Induced Blood Pressure Reduction: Mechanisms for the Treatment of Hypertension via the Gut. Front Cardiovasc Med, 2021,8:721393.

[375] Coppa G V, Gabrielli O, Zampini L, et al. Oligosaccharides in 4 different milk groups, Bifidobacteria, and Ruminococcus obeum . J Pediatr Gastroenterol Nutr, 2011,53(1):80-87.

[376] Cornejo M A, Ortiz R M. Body mass cycling and predictors of body mass regain and its impact on cardiometabolic health . Metabolism, 2021,125:154912.

[377] Cortés-Martín A, Iglesias-Aguirre C E, Meoro A, et al. There is No Distinctive Gut Microbiota Signature in the Metabolic Syndrome: Contribution of Cardiovascular Disease Risk Factors and Associated Medication. Microorganisms, 2020,8(3).

[378] Corteville C, Fassnacht M, Bueter M. [Surgery as pluripotent instrument for metabolic disease. What are the mechanisms?] . Chirurg, 2014,85(11):963-968.

[379] Coutinho-Wolino K S, de F C L, de Oliveira L V, et al. Can diet modulate trimethylamine N-oxide (TMAO) production? What do we know so far?. Eur J Nutr, 2021,60(7):3567-3584.

[380] Covert R F, Schreiber M D. Three different strains of heat-killed group B beta-hemolytic streptococcus cause different pulmonary and systemic hemodynamic responses in conscious neonatal lambs . Pediatr Res, 1993,33(4 Pt 1):373-379.

[381] Cox A J, Watts A M, Zhang P, et al. Effects of short-term supplementation with bovine lactoferrin and/or immunoglobulins on body mass and metabolic measures: a randomised controlled trial . Int J Food Sci Nutr, 2017,68(2):219-226.

[382] Cox A J, Zhang P, Bowden D W, et al. Increased intestinal permeability as a risk factor for type 2 diabetes. Diabetes Metab, 2017,43(2):163-166.

[383] Creus-Cuadros A, Tresserra-Rimbau A, Quifer-Rada P, et al. Associations between Both Lignan and Yogurt Consumption and Cardiovascular Risk Parameters in an Elderly Population: Observations from a Cross-Sectional Approach in the PREDIMED Study. J Acad Nutr Diet, 2017,117(4):609-622.

[384] Cromwell E A, Osborne J, Unnasch T R, et al. Predicting the environmental suitability for onchocerciasis in Africa as an aid to elimination planning . PLoS Negl Trop Dis, 2021,15(7):e8824.

[385] Crosland W, Ahmed M, Booker J, et al. Pulmonary endarterectomy for pulmonary hypertension from septic emboli . Ann Thorac Surg, 2015,99(5):1814-1816.

[386] Cui M, Qi C, Yang L, et al. A pregnancy complication-dependent change in SIgA-targeted microbiota during third trimester . Food Funct, 2020,11(2):1513-1524.

[387] Curini L, Amedei A. Cardiovascular Diseases and Pharmacomicrobiomics: A Perspective on Possible Treatment Relevance . Biomedicines, 2021,9(10).

[388] Curone G, Filipe J, Cremonesi P, et al. What we have lost: Mastitis resistance in Holstein Friesians and in a local cattle breed . Res Vet Sci, 2018,116:88-98.

[389] Curtis J, Kim G, Wehr N B, et al. Group B streptococcal phospholipid causes pulmonary hypertension . Proc Natl Acad Sci U S A, 2003,100(9):5087-5090.

[390] Curtis J, Kim G, Wehr N B, et al. Group B Streptococcus, phospholipids and pulmonary hypertension . J Perinatol, 2011,31 Suppl 1(Suppl 1):S24-S28.

[391] Cyprian F, Sohail M U, Abdelhafez I, et al. SARS-CoV-2 and immune-microbiome interactions: Lessons from respiratory viral infections . Int J Infect Dis, 2021,105:540-550.

[392] Czerniuk M R, Surma S, Romańczyk M, et al. Unexpected Relationships: Periodontal Diseases: Atherosclerosis-Plaque Destabilization? From the Teeth to a Coronary Event . Biology (Basel), 2022,11(2).

[393] Czikora I, Alli A A, Sridhar S, et al. Epithelial Sodium Channel-α Mediates the Protective Effect of the TNF-Derived TIP Peptide in Pneumolysin-Induced Endothelial Barrier Dysfunction . Front Immunol, 2017,8:842.

[394] Da F C L, de Souza M J, de Medeiros S I, et al. Beverages Rich in Resveratrol and Physical Activity Attenuate Metabolic Changes Induced by High-Fat Diet . J Am Coll Nutr, 2021,40(6):485-495.

[395] Da S M, Converso T R, Gonçalves V M, et al. Conjugation of PspA4Pro with Capsular Streptococcus pneumoniae Polysaccharide Serotype 14 Does Not Reduce the Induction of Cross-Reactive Antibodies . Clin Vaccine Immunol, 2017,24(8).

[396] Da S R M, Siqueira E S, Brant C Q, et al. Prospective study of bacteremia rate after elastic band ligation and sclerotherapy of esophageal varices in patients with hepatosplenic schistosomiasis . Gastrointest Endosc, 1997,46(4):321-323.

[397] Dabarera M C, Athiththan L V, Perera R P. Antihypertensive peptides from curd . Ayu, 2015,36(2):214-219.

[398] Dabrowska K, Hehre D, Ladino J, et al. Effects of intermittent nebulization of NONOate, DPTA/NO, on group B Streptococcus-induced pulmonary hypertension in newborn piglets . Neonatology, 2011,99(1):57-64.

[399] Dabrowska K, Hehre D, Young K C, et al. Effects of a nebulized NONOate, DPTA/NO, on group B streptococcus-induced pulmonary hypertension in newborn piglets . Pediatr Res, 2005,57(3):378-383.

[400] Dabur R, Shirolkar A, Mishra V, et al. Non-invasive Qualitative Urinary Metabolomic Profiling Discriminates Gut Microbiota Derived Metabolites in the Moderate and Chronic Alcoholic Cohorts. Curr Pharm Biotechnol, 2017,18(14):1175-1189.

[401] Dahan S, Segal Y, Shoenfeld Y. Dietary factors in rheumatic autoimmune diseases: a recipe for therapy? . Nat Rev Rheumatol, 2017,13(6):348-358.

[402] Dahl W J, Agro N C, Eliasson Å M, et al. Health Benefits of Fiber Fermentation . J Am Coll Nutr, 2017,36(2):127-136.

[403] Dahlberg C J, Ou J J, Babish J G, et al. A 13-week low glycemic load diet and lifestyle modification program combining low glycemic load protein shakes and targeted nutraceuticals improved weight loss and cardio-metabolic risk factors . Can J Physiol Pharmacol, 2017,95(12):1414-1425.

[404] Dahmash N S, Chowdhury M N. Re-evaluation of pneumonia requiring admission to an intensive care unit: a prospective study . Thorax, 1994,49(1):71-76.

[405] Dale H F, Madsen L, Lied G A. Fish-derived proteins and their potential to improve human health. Nutr Rev, 2019.

[406] Daley D K, Myrie S B. Extra-skeletal effects of dietary calcium: Impact on the cardiovascular system, obesity, and cancer . Adv Food Nutr Res, 2021,96:1-25.

[407] Daliri E B, Lee B H, Oh D H. Current Perspectives on Antihypertensive Probiotics. Probiotics Antimicrob Proteins, 2017,9(2):91-101.

[408] Daliri E B, Ofosu F K, Chelliah R, et al. Development of a Soy Protein Hydrolysate with an Antihypertensive Effect . Int J Mol Sci, 2019,20(6).

[409] Dalmeijer G W, Struijk E A, van der Schouw Y T, et al. Dairy intake and coronary heart disease or stroke--a population-based cohort study . Int J Cardiol, 2013,167(3):925-929.

[410] Damaskos C, Litos A, Dimitroulis D, et al. Cardiovascular Effects of Metabolic Surgery on Type 2 Diabetes . Curr Cardiol Rev, 2020,16(4):275-284.

[411] Dan X, Mushi Z, Baili W, et al. Differential Analysis of Hypertension-Associated Intestinal Microbiota . Int J Med Sci, 2019,16(6):872-881.

[412] Dan X, Mushi Z, Baili W, et al. Erratum: Differential Analysis of Hypertension-Associated Intestinal Microbiota . Int J Med Sci, 2021,18(16):3748.

[413] Daniel H. Diet and Gut Microbiome and the "Chicken or Egg" Problem . Front Nutr, 2021,8:828630.

[414] Daoust L, Pilon G, Marette A. Perspective: Nutritional Strategies Targeting the Gut Microbiome to Mitigate COVID-19 Outcomes . Adv Nutr, 2021,12(4):1074-1086.

[415] Dasinger J H, Abais-Battad J M, Mattson D L. Influences of environmental factors during preeclampsia . Am J Physiol Regul Integr Comp Physiol, 2020,319(1):R26-R32.

[416] Dasinger J H, Fehrenbach D J, Abais-Battad J M. Dietary Protein: Mechanisms Influencing Hypertension and Renal Disease. Curr Hypertens Rep, 2020,22(2):13.

[417] Davidson S J, Barrett H L, Price S A, et al. Probiotics for preventing gestational diabetes . Cochrane Database Syst Rev, 2021,4(4):D9951.

[418] Davila V J, Stone W, Duncan A A, et al. A multicenter experience with the surgical treatment of infected abdominal aortic endografts . J Vasc Surg, 2015,62(4):877-883.

[419] Davis D W, Crew J, Planinic P, et al. Associations of Dietary Bioactive Compounds with Maternal Adiposity and Inflammation in Gestational Diabetes: An Update on Observational and Clinical Studies . Int J Environ Res Public Health, 2020,17(20).

[420] Davis K, de Oliveira L N, Da S A I, et al. Morbidity and mortality of rheumatic heart disease and acute rheumatic fever in the inpatient setting in Timor-Leste . J Paediatr Child Health, 2021,57(9):1391-1396.

[421] Dawson K P, Richardson W W. Acute nephritis in fifty children: clinical and immunological studies. Aust N Z J Med, 1977,7(4):373-379.

[422] Dawwas G K, Brensinger C M, Vajravelu R K, et al. Long-term Outcomes Following Multiply Recurrent Clostridioides difficile Infection and Fecal Microbiota Transplantation . Clin Gastroenterol Hepatol, 2022,20(4):806-816.

[423] de Beer F M, Aslami H, Hoeksma J, et al. Plasma-derived human C1-esterase inhibitor does not prevent mechanical ventilation-induced pulmonary complement activation in a rat model of Streptococcus pneumoniae pneumonia . Cell Biochem Biophys, 2014,70(2):795-803.

[424] de Brito A J, de Sousa V P, Cavalcanti N M, et al. New Insights on the Use of Dietary Polyphenols or Probiotics for the Management of Arterial Hypertension . Front Physiol, 2016,7:448.

[425] De Bruyne T, Steenput B, Roth L, et al. Dietary Polyphenols Targeting Arterial Stiffness: Interplay of Contributing Mechanisms and Gut Microbiome-Related Metabolism. Nutrients, 2019,11(3).

[426] de Buhr N, Martens A, Meurer M, et al. In vivo oxygen measurement in cerebrospinal fluid of pigs to determine physiologic and pathophysiologic oxygen values during CNS infections . BMC Neurosci, 2021,22(1):45.

[427] de Castro R, Sato H H. Biologically active peptides: Processes for their generation, purification and identification and applications as natural additives in the food and pharmaceutical industries. Food Res Int, 2015,74:185-198.

[428] de Faire U, Frostegård J. Natural antibodies against phosphorylcholine in cardiovascular disease. Ann N Y Acad Sci, 2009,1173:292-300.

[429] De Filippis A, Ullah H, Baldi A, et al. Gastrointestinal Disorders and Metabolic Syndrome: Dysbiosis as a Key Link and Common Bioactive Dietary Components Useful for their Treatment . Int J Mol Sci, 2020,21(14).

[430] de Goffau M C, Lager S, Salter S J, et al. Recognizing the reagent microbiome . Nat Microbiol, 2018,3(8):851-853.

[431] de Joode A A, Rheineck L A, Verburg F A, et al. [Bacterial meningitis following spinal anaesthesia] . Ned Tijdschr Geneeskd, 2006,150(5):263-265.

[432] de la Cuesta-Zuluaga J, Corrales-Agudelo V, Carmona J A, et al. Body size phenotypes comprehensively assess cardiometabolic risk and refine the association between obesity and gut microbiota . Int J Obes (Lond), 2018,42(3):424-432.

[433] de la Cuesta-Zuluaga J, Mueller N T, Álvarez-Quintero R, et al. Higher Fecal Short-Chain Fatty Acid Levels Are Associated with Gut Microbiome Dysbiosis, Obesity, Hypertension and Cardiometabolic Disease Risk Factors . Nutrients, 2018,11(1).

[434] de la Visitación N, Robles-Vera I, Moleón J, et al. Gut Microbiota Has a Crucial Role in the Development of Hypertension and Vascular Dysfunction in Toll-like Receptor 7-Driven Lupus Autoimmunity . Antioxidants (Basel), 2021,10(9).

[435] de la Visitación N, Robles-Vera I, Moleón-Moya J, et al. Probiotics Prevent Hypertension in a Murine Model of Systemic Lupus Erythematosus Induced by Toll-Like Receptor 7 Activation . Nutrients, 2021,13(8).

[436] de la Visitación N, Robles-Vera I, Toral M, et al. Protective Effects of Probiotic Consumption in Cardiovascular Disease in Systemic Lupus Erythematosus . Nutrients, 2019,11(11).

[437] de la Visitación N, Robles-Vera I, Toral M, et al. Gut microbiota contributes to the development of hypertension in a genetic mouse model of systemic lupus erythematosus . Br J Pharmacol, 2021,178(18):3708-3729.

[438] de la Visitación N, Robles-Vera I, Toral M, et al. Lactobacillus fermentum CECT5716 prevents renal damage in the NZBWF1 mouse model of systemic lupus erythematosus . Food Funct, 2020,11(6):5266-5274.

[439] De Minicis S, Antonini F, Belfiori V, et al. Small bowel diverticulitis with severe anemia and abdominal pain . World J Clin Cases, 2015,3(5):462-465.

[440] de Morais C L, Pinheiro S S, Martino H S, et al. Sorghum (Sorghum bicolor L.): Nutrients, bioactive compounds, and potential impact on human health . Crit Rev Food Sci Nutr, 2017,57(2):372-390.

[441] De Oliveira D M, Hartley-Tassell L, Everest-Dass A, et al. Blood Group Antigen Recognition via the Group A Streptococcal M Protein Mediates Host Colonization . mBio, 2017,8(1).

[442] de Oliveira Y, Cavalcante R, Cavalcanti N M, et al. Oral administration of Lactobacillus fermentum post-weaning improves the lipid profile and autonomic dysfunction in rat offspring exposed to maternal dyslipidemia . Food Funct, 2020,11(6):5581-5594.

[443] de Waard M, Brands B, Kouwenhoven S, et al. Optimal nutrition in lactating women and its effect on later health of offspring: A systematic review of current evidence and recommendations (EarlyNutrition project) . Crit Rev Food Sci Nutr, 2017,57(18):4003-4016.

[444] Del B C, Bernardi S, Cherubini A, et al. A polyphenol-rich dietary pattern improves intestinal permeability, evaluated as serum zonulin levels, in older subjects: The MaPLE randomised controlled trial . Clin Nutr, 2021,40(5):3006-3018.

[445] Del C F, Manco M, Gardini S, et al. Fecal microbiota signatures of insulin resistance, inflammation, and metabolic syndrome in youth with obesity: a pilot study . Acta Diabetol, 2021,58(8):1009-1022.

[446] Del M T, Goldberg R N, Suguihara C, et al. Effects of pentoxifylline on the cardiovascular manifestations of group B streptococcal sepsis in the piglet . Pediatr Res, 1992,31(6):596-600.

[447] Del P R, Pietropaoli D, Monaco A, et al. Non-pharmacological Strategies Against Systemic Inflammation: Molecular Basis and Clinical Evidence . Curr Pharm Des, 2020,26(22):2620-2629.

[448] Dela C T, Stewart D L, Robinson T W, et al. The use of a second course of extracorporeal membrane oxygenation in neonatal patients . ASAIO J, 1996,42(3):230-232.

[449] Dellamonica P, Bernard E, Etesse H, et al. The diffusion of pefloxacin into bone and the treatment of osteomyelitis. J Antimicrob Chemother, 1986,17 Suppl B:93-102.

[450] Demers B, Simor A E, Vellend H, et al. Severe invasive group A streptococcal infections in Ontario, Canada: 1987-1991 . Clin Infect Dis, 1993,16(6):792-800, 801-802.

[451] Depommier C, Everard A, Druart C, et al. Supplementation with Akkermansia muciniphila in overweight and obese human volunteers: a proof-of-concept exploratory study . Nat Med, 2019,25(7):1096-1103.

[452] Derkach A, Sampson J, Joseph J, et al. Effects of dietary sodium on metabolites: the Dietary Approaches to Stop Hypertension (DASH)-Sodium Feeding Study . Am J Clin Nutr, 2017,106(4):1131-1141.

[453] Derrick C W, Reeves M S, Dillon H J. Complement in overt and asymptomatic nephritis after skin infection . J Clin Invest, 1970,49(6):1178-1187.

[454] Desbonnet L, Garrett L, Clarke G, et al. The probiotic Bifidobacteria infantis: An assessment of potential antidepressant properties in the rat. J Psychiatr Res, 2008,43(2):164-174.

[455] Desvarieux M, Demmer R T, Jacobs D J, et al. Periodontal bacteria and hypertension: the oral infections and vascular disease epidemiology study (INVEST) . J Hypertens, 2010,28(7):1413-1421.

[456] Desvarieux M, Demmer R T, Rundek T, et al. Periodontal microbiota and carotid intima-media thickness: the Oral Infections and Vascular Disease Epidemiology Study (INVEST) . Circulation, 2005,111(5):576-582.

[457] Di Renzo L, Gualtieri P, Romano L, et al. Role of Personalized Nutrition in Chronic-Degenerative Diseases. Nutrients, 2019,11(8).

[458] Dias P, Pourová J, Vopršalová M, et al. 3-Hydroxyphenylacetic Acid: A Blood Pressure-Reducing Flavonoid Metabolite. Nutrients, 2022,14(2).

[459] Dieng M T, Ndiaye B, Ndiaye A M. [Scabies complicated by acute glomerulonephritis in children: 114 cases observed in two years in a pediatric service in Dakar] . Dakar Med, 1998,43(2):201-204.

[460] Diez A M, Urso R, Rantsiou K, et al. Spoilage of blood sausages morcilla de Burgos treated with high hydrostatic pressure . Int J Food Microbiol, 2008,123(3):246-253.

[461] Dimitrov Z, Chorbadjiyska E, Gotova I, et al. Selected adjunct cultures remarkably increase the content of bioactive peptides in Bulgarian white brined cheese . Biotechnol Biotechnol Equip, 2015,29(1):78-83.

[462] Dinakis E, Nakai M, Gill P A, et al. The Gut Microbiota and Their Metabolites in Human Arterial Stiffness . Heart Lung Circ, 2021,30(11):1716-1725.

[463] Ding R X, Goh W R, Wu R N, et al. Revisit gut microbiota and its impact on human health and disease . J Food Drug Anal, 2019,27(3):623-631.

[464] Dite P, Blaho M, Bojkova M, et al. Nonalcoholic Fatty Pancreas Disease: Clinical Consequences. Dig Dis, 2020,38(2):143-149.

[465] Dixon A, Robertson K, Yung A, et al. Efficacy of Probiotics in Patients of Cardiovascular Disease Risk: a Systematic Review and Meta-analysis . Curr Hypertens Rep, 2020,22(9):74.

[466] Dixon P M, Railton D I, McGorum B C. Equine pulmonary disease: a case control study of 300 referred cases. Part 1: Examination techniques, diagnostic criteria and diagnoses. Equine Vet J, 1995,27(6):416-421.

[467] Docherty N G, Le Roux C W. Physiological adaptations following Roux-en-Y gastric bypass and the identification of targets for bariatric mimetic pharmacotherapy . Curr Opin Pharmacol, 2015,25:23-29.

[468] Dodge W F, Spargo B H, Bass J A, et al. The relationship between the clinical and pathologic features of poststreptococcal glomerulonephritis. A study of the early natural history . Medicine (Baltimore), 1968,47(3):227-267.

[469] Doghri Y, Chetaneau F, Rhimi M, et al. Sildenafil citrate long-term treatment effects on cardiovascular reactivity in a SHR experimental model of metabolic syndrome . PLoS One, 2019,14(11):e223914.

[470] Doki N, Suyama M, Sasajima S, et al. Clinical impact of pre-transplant gut microbial diversity on outcomes of allogeneic hematopoietic stem cell transplantation . Ann Hematol, 2017,96(9):1517-1523.

[471] Domínguez G K, Cruz G A, Márquez H G, et al. [The antihypertensive effect of fermented milks] . Rev Argent Microbiol, 2014,46(1):58-65.

[472] Donderski R, Grajewska M, Mikucka A, et al. Achromobacter xylosoxidans Relapsing Peritonitis and Streptococcus suis Peritonitis in Peritoneal Dialysis Patients: A Report of Two Cases . Case Rep Nephrol, 2018,2018:9454520.

[473] Donertas A B, Oliveira A C, Malphurs W L, et al. Central Administration of Hydrogen Sulfide Donor NaHS Reduces Iba1-Positive Cells in the PVN and Attenuates Rodent Angiotensin II Hypertension . Front Neurosci, 2021,15:690919.

[474] Donertas A B, Zubcevic J. Gut microbiota and neuroinflammation in pathogenesis of hypertension: A potential role for hydrogen sulfide . Pharmacol Res, 2020,153:104677.

[475] Dong J Y, Szeto I M, Makinen K, et al. Effect of probiotic fermented milk on blood pressure: a meta-analysis of randomised controlled trials . Br J Nutr, 2013,110(7):1188-1194.

[476] Dong T S, Jacobs J P, Hussain S K. Microbial Profiles of Cirrhosis in the Human Small Intestine. Curr Gastroenterol Rep, 2019,21(10):50.

[477] Dong Y, Xu M, Chen L, et al. Probiotic Foods and Supplements Interventions for Metabolic Syndromes: A Systematic Review and Meta-Analysis of Recent Clinical Trials . Ann Nutr Metab, 2019,74(3):224-241.

[478] Dorvilus P, Edoo-Sowah R. Streptococcus milleri: a cause of pyogenic liver abscess . J Natl Med Assoc, 2001,93(7-8):276-277.

[479] Dowd A J, Kronlund L, Parmar C, et al. A 12-Week Pilot Exercise Program for Inactive Adults With Celiac Disease: Study Protocol . Glob Adv Health Med, 2019,8:544893649.

[480] Drachman R, Aladjem M, Vardy P A. Natural history of an acute glomerulonephritis epidemic in children. An 11- to 12-year follow-up . Isr J Med Sci, 1982,18(5):603-607.

[481] Drago F, Gariazzo L, Cioni M, et al. The microbiome and its relevance in complex wounds. Eur J Dermatol, 2019,29(1):6-13.

[482] Drago L, Fassina M C, De Vecchi E, et al. Lack of in vitro and in vivo selection of bacterial resistance by roxithromycin . Chemotherapy, 2000,46(3):160-165.

[483] Drake C W, Hunt R J, Koch G G. Three-year tooth loss among black and white older adults in North Carolina . J Dent Res, 1995,74(2):675-680.

[484] Drapala A, Szudzik M, Chabowski D, et al. Heart Failure Disturbs Gut-Blood Barrier and Increases Plasma Trimethylamine, a Toxic Bacterial Metabolite . Int J Mol Sci, 2020,21(17).

[485] Dreher M L. A Comprehensive Review of Almond Clinical Trials on Weight Measures, Metabolic Health Biomarkers and Outcomes, and the Gut Microbiota . Nutrients, 2021,13(6).

[486] Drouet A, Le Moigne F, Donat A, et al. [Brain abscess as the first clinical manifestation of isolated pulmonary arteriovenous malformation without Rendu-Osler disease] . Rev Neurol (Paris), 2011,167(1):29-34.

[487] Drouin-Chartier J P, Brassard D, Tessier-Grenier M, et al. Systematic Review of the Association between Dairy Product Consumption and Risk of Cardiovascular-Related Clinical Outcomes . Adv Nutr, 2016,7(6):1026-1040.

[488] Druml W. [Intestinal cross-talk : The gut as motor of multiple organ failure] . Med Klin Intensivmed Notfmed, 2018,113(6):470-477.

[489] Dubey L, Krasinski K, Hernanz-Schulman M. Osteomyelitis secondary to trauma or infected contiguous soft tissue. Pediatr Infect Dis J, 1988,7(1):26-34.

[490] Dubinski P, Czarzasta K, Cudnoch-Jedrzejewska A. The Influence of Gut Microbiota on the Cardiovascular System Under Conditions of Obesity and Chronic Stress . Curr Hypertens Rep, 2021,23(5):31.

[491] Duca E, Teodorovici G, Radu C, et al. A new nephritogenic streptococcus. J Hyg (Lond), 1969,67(4):691-698.

[492] Dueker S R, Lin Y, Jones A D, et al. Determination of blood folate using acid extraction and internally standardized gas chromatography-mass spectrometry detection . Anal Biochem, 2000,283(2):266-275.

[493] Durgan D J. Obstructive Sleep Apnea-Induced Hypertension: Role of the Gut Microbiota. Curr Hypertens Rep, 2017,19(4):35.

[494] Durgan D J. Evidence for a gut-immune-vascular axis in the development of hypertension. Acta Physiol (Oxf), 2019,227(1):e13338.

[495] Durgan D J, Ganesh B P, Cope J L, et al. Role of the Gut Microbiome in Obstructive Sleep Apnea-Induced Hypertension . Hypertension, 2016,67(2):469-474.

[496] Duseja A, Acharya S K, Mehta M, et al. High potency multistrain probiotic improves liver histology in non-alcoholic fatty liver disease (NAFLD): a randomised, double-blind, proof of concept study . BMJ Open Gastroenterol, 2019,6(1):e315.

[497] Dutkiewicz J, Zając V, Sroka J, et al. Streptococcus suis: a re-emerging pathogen associated with occupational exposure to pigs or pork products. Part II - Pathogenesis. Ann Agric Environ Med, 2018,25(1):186-203.

[498] Duttaroy A K. Role of Gut Microbiota and Their Metabolites on Atherosclerosis, Hypertension and Human Blood Platelet Function: A Review . Nutrients, 2021,13(1).

[499] Ebel B, Lemetais G, Beney L, et al. Impact of probiotics on risk factors for cardiovascular diseases. A review. Crit Rev Food Sci Nutr, 2014,54(2):175-189.

[500] Ebringer L, Ferencík M, Krajcovic J. Beneficial health effects of milk and fermented dairy products--review. Folia Microbiol (Praha), 2008,53(5):378-394.

[501] Echols R, Weinstein M P, O'Keeffe B, et al. Comparative crossover assessment of serum bactericidal activity and pharmacokinetics of ciprofloxacin and ofloxacin . J Antimicrob Chemother, 1994,33(1):111-118.

[502] Edmands W M, Petrick L, Barupal D K, et al. compMS2Miner: An Automatable Metabolite Identification, Visualization, and Data-Sharing R Package for High-Resolution LC-MS Data Sets . Anal Chem, 2017,89(7):3919-3928.

[503] Edmonds A, Ludema C, Eron J J, et al. Effects of Health Insurance Interruption on Loss of Hypertension Control in Women With and Women Without HIV. J Womens Health (Larchmt), 2017,26(12):1292-1301.

[504] Edwards J D, Schofield P M. Myocardial depression in streptococcal cellulitis. Br Med J (Clin Res Ed), 1984,288(6420):816-817.

[505] Edwards J M, Watson N, Focht C, et al. Group B Streptococcus (GBS) Colonization and Disease among Pregnant Women: A Historical Cohort Study . Infect Dis Obstet Gynecol, 2019,2019:5430493.

[506] Ehlers P I, Kivimäki A S, Turpeinen A M, et al. High blood pressure-lowering and vasoprotective effects of milk products in experimental hypertension . Br J Nutr, 2011,106(9):1353-1363.

[507] Ehrenstein B P, Salzberger B, Glück T. [New developments in the diagnosis and therapy of acute bacterial meningitis] . Med Klin (Munich), 2005,100(6):325-333.

[508] Eicken A, Schöber J G, Roos R, et al. Cerebrospinal fluid penetration of cefmenoxime in children with bacterial meningitis . Infection, 1991,19(6):406-408.

[509] Ejtahed H S, Ardeshirlarijani E, Tabatabaei-Malazy O, et al. Effect of probiotic foods and supplements on blood pressure: a systematic review of meta-analyses studies of controlled trials . J Diabetes Metab Disord, 2020,19(1):617-623.

[510] Elijovich F, Laffer C L, Sahinoz M, et al. The Gut Microbiome, Inflammation, and Salt-Sensitive Hypertension. Curr Hypertens Rep, 2020,22(10):79.

[511] Elikowski W, Fertała N, Zawodna-Marszałek M, et al. Concomitance of COVID-19 and legionnaires' disease - a case series . Pol Merkur Lekarski, 2022,50(295):30-36.

[512] Elkhtab E, El-Alfy M, Shenana M, et al. New potentially antihypertensive peptides liberated in milk during fermentation with selected lactic acid bacteria and kombucha cultures. J Dairy Sci, 2017,100(12):9508-9520.

[513] Elliott B, Brunham R C, Laga M, et al. Maternal gonococcal infection as a preventable risk factor for low birth weight . J Infect Dis, 1990,161(3):531-536.

[514] Elshamly M, Nour M O, Omar A. Clinical presentations and outcome of severe community-acquired pneumonia . Egypt J Chest Dis Tuberc, 2016,65(4):831-839.

[515] Ensink J M, Klein W R, Barneveld A, et al. Clinical efficacy of ampicillin, pivampicillin and procaine penicillin G in a soft tissue infection model in ponies . J Vet Pharmacol Ther, 1996,19(6):445-453.

[516] Ensink J M, Klein W R, Barneveld A, et al. Distribution of penicillins into subcutaneous tissue chambers in ponies . J Vet Pharmacol Ther, 1996,19(6):439-444.

[517] Eren Z, Gurol Y, Sonmezoglu M, et al. [Saccharomyces cerevisiae fungemia in an elderly patient following probiotic treatment] . Mikrobiyol Bul, 2014,48(2):351-355.

[518] Ericson U, Hellstrand S, Brunkwall L, et al. Food sources of fat may clarify the inconsistent role of dietary fat intake for incidence of type 2 diabetes . Am J Clin Nutr, 2015,101(5):1065-1080.

[519] Eslamparast T, Tandon P, Raman M. Dietary Composition Independent of Weight Loss in the Management of Non-Alcoholic Fatty Liver Disease . Nutrients, 2017,9(8).

[520] Estadella D, Da P O D N, Oyama L M, et al. Lipotoxicity: effects of dietary saturated and transfatty acids. Mediators Inflamm, 2013,2013:137579.

[521] Ettinger G, MacDonald K, Reid G, et al. The influence of the human microbiome and probiotics on cardiovascular health. Gut Microbes, 2014,5(6):719-728.

[522] Evseev V A. [Functional characteristics of experimental allergy caused by Streptococcus] . Biull Eksp Biol Med, 1965,59(6):50-53.

[523] Ewaschuk J B, Zello G A, Naylor J M. Lactobacillus GG does not affect D-lactic acidosis in diarrheic calves, in a clinical setting . J Vet Intern Med, 2006,20(3):614-619.

[524] Ewig S, Klapdor B, Pletz M W, et al. Nursing-home-acquired pneumonia in Germany: an 8-year prospective multicentre study . Thorax, 2012,67(2):132-138.

[525] Ewles M, Mannu R, Fox C, et al. LC-MS/MS strategies for therapeutic antibodies and investigation into the quantitative impact of antidrug-antibodies . Bioanalysis, 2016,8(24):2565-2579.

[526] F S T T, Grześkowiak L M, Salminen S, et al. Faecal levels of Bifidobacterium and Clostridium coccoides but not plasma lipopolysaccharide are inversely related to insulin and HOMA index in women . Clin Nutr, 2013,32(6):1017-1022.

[527] Faber L, Nissen P. [Pulmonary valve endocarditis, bilateral deep vein thrombosis amd recurrent pulmonary emboli] . Dtsch Med Wochenschr, 1993,118(47):1714-1721.

[528] Fabian E, Elmadfa I. The effect of daily consumption of probiotic and conventional yoghurt on oxidant and anti-oxidant parameters in plasma of young healthy women. Int J Vitam Nutr Res, 2007,77(2):79-88.

[529] Fabian E, Majchrzak D, Dieminger B, et al. Influence of probiotic and conventional yoghurt on the status of vitamins B1, B2 and B6 in young healthy women . Ann Nutr Metab, 2008,52(1):29-36.

[530] Fabris M, Zago S, Tosolini R, et al. Anti-DFS70 antibodies: a useful biomarker in a pediatric case with suspected autoimmune disease . Pediatrics, 2014,134(6):e1706-e1708.

[531] Falaleeva M, Zurek O W, Watkins R L, et al. Transcription of the Streptococcus pyogenes hyaluronic acid capsule biosynthesis operon is regulated by previously unknown upstream elements . Infect Immun, 2014,82(12):5293-5307.

[532] Fallucca F, Fontana L, Fallucca S, et al. Gut microbiota and Ma-Pi 2 macrobiotic diet in the treatment of type 2 diabetes . World J Diabetes, 2015,6(3):403-411.

[533] Fan N, Peng L, Xia Z, et al. Helicobacter pylori Infection Is Not Associated with Non-alcoholic Fatty Liver Disease: A Cross-Sectional Study in China . Front Microbiol, 2018,9:73.

[534] Fändriks L. Roles of the gut in the metabolic syndrome: an overview. J Intern Med, 2017,281(4):319-336.

[535] Fardella C E, Claverie X, Vignolo P, et al. T235 variant of the angiotensinogen gene and blood pressure in the Chilean population . J Hypertens, 1998,16(6):829-833.

[536] Fardy P W, Matheson B H, Reed R W. Experimental nephritis due to type specific streptococci. VI. Further characterization of type 12 nephrotoxin . Can J Microbiol, 1969,15(6):555-561.

[537] Farré N, Farré R, Gozal D. Sleep Apnea Morbidity: A Consequence of Microbial-Immune Cross-Talk?. Chest, 2018,154(4):754-759.

[538] Fasullo M, Rau P, Liu D Q, et al. Proton pump inhibitors increase the severity of hepatic encephalopathy in cirrhotic patients . World J Hepatol, 2019,11(6):522-530.

[539] Fava F, Rizzetto L, Tuohy K M. Gut microbiota and health: connecting actors across the metabolic system. Proc Nutr Soc, 2019,78(2):177-188.

[540] Fayed A E. Review article: health benefits of some physiologically active ingredients and their suitability as yoghurt fortifiers . J Food Sci Technol, 2015,52(5):2512-2521.

[541] Fazili T, Riddell S, Kiska D, et al. Streptococcus anginosus Group Bacterial Infections . Am J Med Sci, 2017,354(3):257-261.

[542] Fei N, Bernabé B P, Lie L, et al. The human microbiota is associated with cardiometabolic risk across the epidemiologic transition. PLoS One, 2019,14(7):e215262.

[543] Feng G F, Liu S, Pi Z F, et al. Comprehensive characterization of in vivo metabolic profile of Polygalae radix based on ultra-high-performance liquid chromatography-tandem mass spectrometry . J Pharm Biomed Anal, 2019,165:173-181.

[544] Feng R, Shou J W, Zhao Z X, et al. Transforming berberine into its intestine-absorbable form by the gut microbiota. Sci Rep, 2015,5:12155.

[545] Ferdinand K C, Graham R M. Disparities in hypertension and cardiovascular disease in African Americans: Is the answer in the gut microbiota?. Int J Cardiol, 2018,271:340-342.

[546] Ferguson J F, Aden L A, Barbaro N R, et al. High dietary salt-induced dendritic cell activation underlies microbial dysbiosis-associated hypertension . JCI Insight, 2019,5(13).

[547] Fernandes M F, de Oliveira S, Portovedo M, et al. Effect of Short Chain Fatty Acids on Age-Related Disorders . Adv Exp Med Biol, 2020,1260:85-105.

[548] Fernandez M A, Panahi S, Daniel N, et al. Yogurt and Cardiometabolic Diseases: A Critical Review of Potential Mechanisms . Adv Nutr, 2017,8(6):812-829.

[549] Ferreira M, Salgueiro A B, Estrada J, et al. [Lúpus erythematosus] . Acta Med Port, 2008,21(2):199-204.

[550] Ferreira-Lazarte A, Fernández J, Gallego-Lobillo P, et al. Behaviour of citrus pectin and modified citrus pectin in an azoxymethane/dextran sodium sulfate (AOM/DSS)-induced rat colorectal carcinogenesis model. Int J Biol Macromol, 2021,167:1349-1360.

[551] Fialho A, Fialho A, Thota P, et al. Small Intestinal Bacterial Overgrowth Is Associated with Non-Alcoholic Fatty Liver Disease . J Gastrointestin Liver Dis, 2016,25(2):159-165.

[552] Fialho A, Fialho A, Thota P, et al. Higher visceral to subcutaneous fat ratio is associated with small intestinal bacterial overgrowth . Nutr Metab Cardiovasc Dis, 2016,26(9):773-777.

[553] File T M. The science of selecting antimicrobials for community-acquired pneumonia (CAP) . J Manag Care Pharm, 2009,15(2 Suppl):S5-S11.

[554] Fiore M T, Pearlman M D, Chapman R L, et al. Maternal and transplacental pharmacokinetics of cefazolin . Obstet Gynecol, 2001,98(6):1075-1079.

[555] Fiorucci S, Zampella A, Cirino G, et al. Decoding the vasoregulatory activities of bile acid-activated receptors in systemic and portal circulation: role of gaseous mediators . Am J Physiol Heart Circ Physiol, 2017,312(1):H21-H32.

[556] Fireman P. Otitis media and eustachian tube dysfunction: connection to allergic rhinitis . J Allergy Clin Immunol, 1997,99(2):S787-S797.

[557] Flamaing J, De Backer W, Van Laethem Y, et al. Pneumococcal lower respiratory tract infections in adults: an observational case-control study in primary care in Belgium . BMC Fam Pract, 2015,16:66.

[558] Flasche S, Edmunds W J, Miller E, et al. The impact of specific and non-specific immunity on the ecology of Streptococcus pneumoniae and the implications for vaccination . Proc Biol Sci, 2013,280(1771):20131939.

[559] Flenley D C. Chronic obstructive pulmonary disease. Dis Mon, 1988,34(9):537-599.

[560] Foisy S M, Spahis S, Delvin E, et al. Glycomacropeptide: A Bioactive Milk Derivative to Alleviate Metabolic Syndrome Outcomes . Antioxid Redox Signal, 2021,34(3):201-222.

[561] Folchetti L G, Silva I T, Almeida-Pititto B, et al. Nutritionists' Health Study cohort: a web-based approach of life events, habits and health outcomes . BMJ Open, 2016,6(8):e12081.

[562] Fousekis F S, Mitselos I V, Christodoulou D K. New insights into intestinal failure-associated liver disease in adults: A comprehensive review of the literature . Saudi J Gastroenterol, 2021,27(1):3-12.

[563] Fraga C G, Croft K D, Kennedy D O, et al. The effects of polyphenols and other bioactives on human health. Food Funct, 2019,10(2):514-528.

[564] Franceschi C, Ostan R, Santoro A. Nutrition and Inflammation: Are Centenarians Similar to Individuals on Calorie-Restricted Diets? . Annu Rev Nutr, 2018,38:329-356.

[565] Franco-Paredes C, Evans J, Jurado R. Diabetes insipidus due to Streptococcus pneumoniae meningitis . Arch Intern Med, 2001,161(8):1114-1115.

[566] Franco-Paredes C, Lammoglia L, Hernández I, et al. Epidemiology and outcomes of bacterial meningitis in Mexican children: 10-year experience (1993-2003) . Int J Infect Dis, 2008,12(4):380-386.

[567] Frankenfeld C L. Cardiometabolic risk and gut microbial phytoestrogen metabolite phenotypes . Mol Nutr Food Res, 2017,61(1).

[568] Freeman R M, Simpson D C, Derrick C W. Streptococcal gangrene in an infant with congenital lymphedema . South Med J, 1973,66(7):830-832.

[569] Friedrich M, Goluch-Koniuszy Z. Assessment of influence of pro-health nutrition education and resulting changes of nutrition behavior of women aged 65-85 on their body content . Prz Menopauzalny, 2015,14(4):223-230.

[570] Frolov E P, Kozlov V K, Shatilova N V, et al. [Role of some neurohumoral mechanisms in the regulation of immuno-allergic processes] . Fiziol Zh SSSR Im I M Sechenova, 1971,57(8):1203-1213.

[571] Frondelius K, Borg M, Ericson U, et al. Lifestyle and Dietary Determinants of Serum Apolipoprotein A1 and Apolipoprotein B Concentrations: Cross-Sectional Analyses within a Swedish Cohort of 24,984 Individuals . Nutrients, 2017,9(3).

[572] Frösen J, Hallikainen J, Pyysalo M, et al. Letter by Frösen et al Regarding Article "Potential Influences of Gut Microbiota on the Formation of Intracranial Aneurysm" . Hypertension, 2019,74(2):e22-e23.

[573] Fu B C, Hullar M, Randolph T W, et al. Associations of plasma trimethylamine N-oxide, choline, carnitine, and betaine with inflammatory and cardiometabolic risk biomarkers and the fecal microbiome in the Multiethnic Cohort Adiposity Phenotype Study . Am J Clin Nutr, 2020,111(6):1226-1234.

[574] Fuglsang A, Rattray F P, Nilsson D, et al. Lactic acid bacteria: inhibition of angiotensin converting enzyme in vitro and in vivo . Antonie Van Leeuwenhoek, 2003,83(1):27-34.

[575] Fuhrer J A, Mihatsch M J, Streuli R A. Poststreptococcal glomerulonephritis flare-up in a patient with varicella infection . Klin Wochenschr, 1990,68(23):1198-1201.

[576] Fukui H. Gut-liver axis in liver cirrhosis: How to manage leaky gut and endotoxemia . World J Hepatol, 2015,7(3):425-442.

[577] Fukui H, Wiest R. Changes of Intestinal Functions in Liver Cirrhosis. Inflamm Intest Dis, 2016,1(1):24-40.

[578] Furiasse D, Gasparotto A M, Monterisi A, et al. [Pneumonia caused byCorynebacterium pseudodiphtheriticum] . Rev Argent Microbiol, 2016,48(4):290-292.

[579] Furuie H, Saisho Y, Yoshikawa T, et al. Intrapulmonary pharmacokinetics of S-013420, a novel bicyclolide antibacterial, in healthy Japanese subjects . Antimicrob Agents Chemother, 2010,54(2):866-870.

[580] Galla S, Chakraborty S, Cheng X, et al. Exposure to Amoxicillin in Early Life Is Associated With Changes in Gut Microbiota and Reduction in Blood Pressure: Findings From a Study on Rat Dams and Offspring . J Am Heart Assoc, 2020,9(2):e14373.

[581] Galla S, Chakraborty S, Cheng X, et al. Disparate effects of antibiotics on hypertension . Physiol Genomics, 2018,50(10):837-845.

[582] Galla S, Chakraborty S, Mell B, et al. Microbiotal-Host Interactions and Hypertension . Physiology (Bethesda), 2017,32(3):224-233.

[583] Gandhi A, Cui Y, Zhou M, et al. Effect of KCl substitution on bacterial viability of Escherichia coli (ATCC 25922) and selected probiotics . J Dairy Sci, 2014,97(10):5939-5951.

[584] Gandhi A, Shah N P. Cell growth and proteolytic activity of Lactobacillus acidophilus, Lactobacillus helveticus, Lactobacillus delbrueckii ssp. bulgaricus, and Streptococcus thermophilus in milk as affected by supplementation with peptide fractions . Int J Food Sci Nutr, 2014,65(8):937-941.

[585] Ganesh B P, Nelson J W, Eskew J R, et al. Prebiotics, Probiotics, and Acetate Supplementation Prevent Hypertension in a Model of Obstructive Sleep Apnea . Hypertension, 2018,72(5):1141-1150.

[586] Gao B, Friedman E S, Regunathan R, et al. Gut Microbiota and Host Cometabolism Are Altered by Patiromer-Induced Changes in Serum and Stool Potassium . Kidney Int Rep, 2021,6(3):821-829.

[587] Gao K, Pi Y, Mu C L, et al. Antibiotics-induced modulation of large intestinal microbiota altered aromatic amino acid profile and expression of neurotransmitters in the hypothalamus of piglets . J Neurochem, 2018,146(3):219-234.

[588] Gao Q, Xu L, Cai J. New drug targets for hypertension: A literature review . Biochim Biophys Acta Mol Basis Dis, 2021,1867(3):166037.

[589] García-Arroyo F E, Gonzaga G, Muñoz-Jiménez I, et al. Probiotic supplements prevented oxonic acid-induced hyperuricemia and renal damage . PLoS One, 2018,13(8):e202901.

[590] García-Lezana T, Raurell I, Bravo M, et al. Restoration of a healthy intestinal microbiota normalizes portal hypertension in a rat model of nonalcoholic steatohepatitis . Hepatology, 2018,67(4):1485-1498.

[591] García-Mena J, Corona-Cervantes K, Cuervo-Zanatta D, et al. Gut microbiota in a population highly affected by obesity and type 2 diabetes and susceptibility to COVID-19 . World J Gastroenterol, 2021,27(41):7065-7079.

[592] Garcia-Perez I, Villaseñor A, Wijeyesekera A, et al. Urinary metabolic phenotyping the slc26a6 (chloride-oxalate exchanger) null mouse model. J Proteome Res, 2012,11(9):4425-4435.

[593] García-Ríos A, Camargo G A, Perez-Jimenez F, et al. Gut microbiota: A new protagonist in the risk of cardiovascular disease? Clin Investig Arterioscler, 2019,31(4):178-185.

[594] Garcia-Rios A, Torres-Peña J D, Perez-Jimenez F, et al. Gut Microbiota: A New Marker of Cardiovascular Disease. Curr Pharm Des, 2017,23(22):3233-3238.

[595] Garcia-Vidal C, Ardanuy C, Tubau F, et al. Pneumococcal pneumonia presenting with septic shock: host- and pathogen-related factors and outcomes . Thorax, 2010,65(1):77-81.

[596] Gardana C, Canzi E, Simonetti P. R(-)-O-desmethylangolensin is the main enantiomeric form of daidzein metabolite produced by human in vitro and in vivo . J Chromatogr B Analyt Technol Biomed Life Sci, 2014,953-954:30-37.

[597] Gare J, Kanoute A, Meda N, et al. Periodontal Conditions and Pathogens Associated with Pre-Eclampsia: A Scoping Review . Int J Environ Res Public Health, 2021,18(13).

[598] Garnier A, Peuchmaur M, Deschênes G. [Postinfectious acute glomerulonephritis] . Nephrol Ther, 2009,5(2):97-101.

[599] Gąsiorowski K, Brokos J B, Sochocka M, et al. Current and Near-Future Treatment of Alzheimer's Disease. Curr Neuropharmacol, 2021.

[600] Gasmi B A, Gasmi A, Doşa A, et al. Association between the gut and oral microbiome with obesity. Anaerobe, 2021,70:102248.

[601] Gasperotti M, Masuero D, Guella G, et al. Development of a targeted method for twenty-three metabolites related to polyphenol gut microbial metabolism in biological samples, using SPE and UHPLC-ESI-MS/MS . Talanta, 2014,128:221-230.

[602] Gatarek P, Kaluzna-Czaplinska J. Trimethylamine N-oxide (TMAO) in human health . EXCLI J, 2021,20:301-319.

[603] Gattringer R, Urbauer E, Traunmüller F, et al. Pharmacokinetics of telithromycin in plasma and soft tissues after single-dose administration to healthy volunteers . Antimicrob Agents Chemother, 2004,48(12):4650-4653.

[604] Gawałko M, Agbaedeng T A, Saljic A, et al. Gut microbiota, dysbiosis and atrial fibrillation. Arrhythmogenic mechanisms and potential clinical implications. Cardiovasc Res, 2021.

[605] Gazmuri R J, de Gomez C A. From a pressure-guided to a perfusion-centered resuscitation strategy in septic shock: Critical literature review and illustrative case . J Crit Care, 2020,56:294-304.

[606] Ge H J, Zhang Z K, Xiao J X, et al. Release of Leu-Pro-Pro from corn gluten meal by fermentation with a Lactobacillus helveticus strain . J Sci Food Agric, 2022,102(3):1095-1104.

[607] Ge X, Zheng L, Zhuang R, et al. The Gut Microbial Metabolite Trimethylamine N-Oxide and Hypertension Risk: A Systematic Review and Dose-Response Meta-analysis . Adv Nutr, 2020,11(1):66-76.

[608] Gedgaudas R, Bajaj J S, Skieceviciene J, et al. Circulating microbiome in patients with portal hypertension . Gut Microbes, 2022,14(1):2029674.

[609] Geerdes-Fenge H F. [Travel-associated pneumonias] . Pneumologie, 2014,68(10):685-695.

[610] Geiger A J, Ward S H, Williams C C, et al. Short communication: Effects of increasing protein and energy in the milk replacer with or without direct-fed microbial supplementation on growth and performance of preweaned Holstein calves . J Dairy Sci, 2014,97(11):7212-7219.

[611] Genade S, Moolman J A, Lochner A. Opioid receptor stimulation acts as mediator of protection in ischaemic preconditioning . Cardiovasc J S Afr, 2001,12(1):8-16.

[612] Gencer B, Li X S, Gurmu Y, et al. Gut Microbiota-Dependent Trimethylamine N-oxide and Cardiovascular Outcomes in Patients With Prior Myocardial Infarction: A Nested Case Control Study From the PEGASUS-TIMI 54 Trial . J Am Heart Assoc, 2020,9(10):e15331.

[613] Georgalaki M, Zoumpopoulou G, Anastasiou R, et al. Lactobacillus kefiranofaciens: From Isolation and Taxonomy to Probiotic Properties and Applications . Microorganisms, 2021,9(10).

[614] George A K, Singh M, Pushpakumar S, et al. Dysbiotic 1-carbon metabolism in cardiac muscle remodeling . J Cell Physiol, 2020,235(3):2590-2598.

[615] Georgieva M, Kagedan L, Lu Y J, et al. Antigenic Variation in Streptococcus pneumoniae PspC Promotes Immune Escape in the Presence of Variant-Specific Immunity . mBio, 2018,9(2).

[616] Gerlini A, Colomba L, Furi L, et al. The role of host and microbial factors in the pathogenesis of pneumococcal bacteraemia arising from a single bacterial cell bottleneck . PLoS Pathog, 2014,10(3):e1004026.

[617] Ghattamaneni N, Panchal S K, Brown L. An improved rat model for chronic inflammatory bowel disease. Pharmacol Rep, 2019,71(1):149-155.

[618] Gheblawi M, Wang K, Oudit G Y. ACE2 (Angiotensin-Converting Enzyme 2)-Mediated Protection From Pulmonary Hypertension: Lung-Gut Axis at Center Stage . Hypertension, 2020,76(1):28-29.

[619] Gheyath B, Akram A, Gong R, et al. A 70-Year-Old Man With Conspicuous Thigh Pain and ST-Segment Elevations. Chest, 2021,159(6):e403-e407.

[620] Ghike S M. Metabolic syndrome - A truly psychosomatic disorder? A global hypothesis. Med Hypotheses, 2016,97:46-53.

[621] Ghosh G, Jesudian A B. Small Intestinal Bacterial Overgrowth in Patients With Cirrhosis. J Clin Exp Hepatol, 2019,9(2):257-267.

[622] Ghosh T S, Arnoux J, O'Toole P W. Metagenomic analysis reveals distinct patterns of gut lactobacillus prevalence, abundance, and geographical variation in health and disease . Gut Microbes, 2020,12(1):1-19.

[623] Giannelli V, Di Gregorio V, Iebba V, et al. Microbiota and the gut-liver axis: bacterial translocation, inflammation and infection in cirrhosis . World J Gastroenterol, 2014,20(45):16795-16810.

[624] Gibson R L, Berger J I, Redding G J, et al. Effect of nitric oxide synthase inhibition during group B streptococcal sepsis in neonatal piglets . Pediatr Res, 1994,36(6):776-783.

[625] Gibson R L, Redding G J, Henderson W R, et al. Group B streptococcus induces tumor necrosis factor in neonatal piglets. Effect of the tumor necrosis factor inhibitor pentoxifylline on hemodynamics and gas exchange . Am Rev Respir Dis, 1991,143(3):598-604.

[626] Gibson R L, Redding G J, Truog W E, et al. Isogenic group B streptococci devoid of capsular polysaccharide or beta-hemolysin: pulmonary hemodynamic and gas exchange effects during bacteremia in piglets . Pediatr Res, 1989,26(3):241-245.

[627] Gibson R L, Truog W E, Henderson W J, et al. Group B streptococcal sepsis in piglets: effect of combined pentoxifylline and indomethacin pretreatment . Pediatr Res, 1992,31(3):222-227.

[628] Gibson R L, Truog W E, Redding G J. Thromboxane-associated pulmonary hypertension during three types of gram-positive bacteremia in piglets . Pediatr Res, 1988,23(6):553-556.

[629] Gibson R L, Truog W E, Redding G J. Hypoxic pulmonary vasoconstriction during and after infusion of group B Streptococcus in neonatal piglets. Vascular pressure-flow analysis . Am Rev Respir Dis, 1988,137(4):774-778.

[630] Giménez L A, Camacho D J, Vila C J, et al. [Hemolytic-uraemic syndrome. A review of 58 cases]. An Pediatr (Barc), 2008,69(4):297-303.

[631] Glass G E, Sheil F, Ruston J C, et al. Necrotising soft tissue infection in a UK metropolitan population . Ann R Coll Surg Engl, 2015,97(1):46-51.

[632] Glassman M S, Berezin S, Boyle J T. Bacterial peritonitis and sepsis presenting as acute gastrointestinal bleeding in patients with portal hypertension . Pediatr Emerg Care, 1993,9(1):19-22.

[633] Glickman-Simon R, Wallace J. Acupuncture for knee osteoarthritis, chasteberry for premenstrual syndrome, probiotics for irritable bowel syndrome, yoga for hypertension, and trigger point dry needling for plantar fasciitis . Explore (NY), 2015,11(2):157-161.

[634] Glimåker M, Johansson B, Halldorsdottir H, et al. [Intracranial pressure targeted treatment in acute bacterial meningitis increased survival] . Lakartidningen, 2014,111(51-52):2288-2291.

[635] Gøbel R J, Larsen N, Jakobsen M, et al. Probiotics to adolescents with obesity: effects on inflammation and metabolic syndrome . J Pediatr Gastroenterol Nutr, 2012,55(6):673-678.

[636] GOEING H. [Experimental studies on the toxin of dysentery bacteria. VIII. On the importance of metabolism and circulation factors for the endotoxin death of different species]. Arb Paul Ehrlich Inst Georg Speyer Haus Ferdinand Blum Inst Frankf A M, 1962,57:80-88.

[637] Goel A, Gupta M, Aggarwal R. Gut microbiota and liver disease . J Gastroenterol Hepatol, 2014,29(6):1139-1148.

[638] Goeser F, Schlabe S, Ruiner C E, et al. Non-invasive fecal microbiota transplantation for recurrent Clostridium difficile infection in a patient presenting with hypertensive disorder post interventionem . Z Gastroenterol, 2016,54(10):1143-1146.

[639] Gohlke A, Ingelmann C J, Nürnberg G, et al. Bioavailability of quercetin from its aglycone and its glucorhamnoside rutin in lactating dairy cows after intraduodenal administration . J Dairy Sci, 2013,96(4):2303-2313.

[640] Goitein K J, Shapiro M. Intracranial pressure and cerebral perfusion pressure in experimental streptococcus pneumoniae meningitis . Res Exp Med (Berl), 1992,192(1):41-47.

[641] Goldberg J J, Pankey J W, Politis I, et al. Effect of oxygen tension on killing of Escherichia coli by bovine polymorphonuclear neutrophil leucocytes in vitro . J Dairy Res, 1995,62(2):331-338.

[642] Goldberg R N, Suguihara C, Martinez O, et al. The role of leukotrienes in the late hemodynamic manifestations of group B streptococcal sepsis in piglets . Prostaglandins Leukot Essent Fatty Acids, 1988,33(3):191-198.

[643] Goldberg R N, Suguihara C, Streitfeld M M, et al. Effects of a leukotriene antagonist on the early hemodynamic manifestations of group B streptococcal sepsis in piglets . Pediatr Res, 1986,20(10):1004-1008.

[644] Golden A R, Baxter M, Adam H J, et al. PCV-15 and PPSV-23 coverage of invasive and respiratory tract Streptococcus pneumoniae, including MDR and XDR isolates: CANWARD 2007-20 . J Antimicrob Chemother, 2022.

[645] Goldman J A, Yeshaya A, Peleg D, et al. Severe pneumococcal peritonitis complicating IUD: case report and review of the literature . Obstet Gynecol Surv, 1986,41(11):672-674.

[646] Golubeva A V, Crampton S, Desbonnet L, et al. Prenatal stress-induced alterations in major physiological systems correlate with gut microbiota composition in adulthood . Psychoneuroendocrinology, 2015,60:58-74.

[647] Gomez-Arango L F, Barrett H L, McIntyre H D, et al. Increased Systolic and Diastolic Blood Pressure Is Associated With Altered Gut Microbiota Composition and Butyrate Production in Early Pregnancy . Hypertension, 2016,68(4):974-981.

[648] Gómez-Hurtado I, Such J, Francés R. Microbiome and bacterial translocation in cirrhosis. Gastroenterol Hepatol, 2016,39(10):687-696.

[649] Gómez-Hurtado I, Such J, Sanz Y, et al. Gut microbiota-related complications in cirrhosis. World J Gastroenterol, 2014,20(42):15624-15631.

[650] Gómez-Juaristi M, Martínez-López S, Sarria B, et al. Bioavailability of hydroxycinnamates in an instant green/roasted coffee blend in humans. Identification of novel colonic metabolites. Food Funct, 2018,9(1):331-343.

[651] Gominak S C. Vitamin D deficiency changes the intestinal microbiome reducing B vitamin production in the gut. The resulting lack of pantothenic acid adversely affects the immune system, producing a "pro-inflammatory" state associated with atherosclerosis and autoimmunity . Med Hypotheses, 2016,94:103-107.

[652] González-Barrio R, Truchado P, García-Villalba R, et al. Metabolism of oak leaf ellagitannins and urolithin production in beef cattle . J Agric Food Chem, 2012,60(12):3068-3077.

[653] González-Domínguez R, Jáuregui O, Mena P, et al. Quantifying the human diet in the crosstalk between nutrition and health by multi-targeted metabolomics of food and microbiota-derived metabolites . Int J Obes (Lond), 2020,44(12):2372-2381.

[654] Gonzalez-Gonzalez C, Gibson T, Jauregi P. Novel probiotic-fermented milk with angiotensin I-converting enzyme inhibitory peptides produced by Bifidobacterium bifidum MF 20/5 . Int J Food Microbiol, 2013,167(2):131-137.

[655] Goodrich J K, Davenport E R, Beaumont M, et al. Genetic Determinants of the Gut Microbiome in UK Twins. Cell Host Microbe, 2016,19(5):731-743.

[656] Gordon J H, LaMonte M J, Genco R J, et al. Is the Oral Microbiome Associated with Blood Pressure in Older Women?. High Blood Press Cardiovasc Prev, 2019,26(3):217-225.

[657] Gouni-Berthold I, Schulte D M, Krone W, et al. The whey fermentation product malleable protein matrix decreases TAG concentrations in patients with the metabolic syndrome: a randomised placebo-controlled trial . Br J Nutr, 2012,107(11):1694-1706.

[658] Goyal A, Nimmakayala K R, Zonszein J. Is there a paradox in obesity?. Cardiol Rev, 2014,22(4):163-170.

[659] Grant M M, Jönsson D. Next Generation Sequencing Discoveries of the Nitrate-Responsive Oral Microbiome and Its Effect on Vascular Responses . J Clin Med, 2019,8(8).

[660] Grant S B, Mudd S, Goldman A. A FURTHER EXPERIMENTAL STUDY ON EXCITATION OF INFECTIONS OF THE THROAT. J Exp Med, 1920,32(1):87-112.

[661] Gras-Le G C, Boscher C, Godon N, et al. Therapeutic amoxicillin levels achieved with oral administration in term neonates . Eur J Clin Pharmacol, 2007,63(7):657-662.

[662] Grgurevic I, Bozin T, Mikus M, et al. Hepatocellular Carcinoma in Non-Alcoholic Fatty Liver Disease: From Epidemiology to Diagnostic Approach . Cancers (Basel), 2021,13(22).

[663] Griffiths B B, Rhee H. Effects of haemolysins of groups A and B streptococci on cardiovascular system. Microbios, 1992,69(278):17-27.

[664] Griffiths M W, Tellez A M. Lactobacillus helveticus: the proteolytic system. Front Microbiol, 2013,4:30.

[665] Groot H E, van de Vegte Y J, Verweij N, et al. Human genetic determinants of the gut microbiome and their associations with health and disease: a phenome-wide association study. Sci Rep, 2020,10(1):14771.

[666] Grylls A, Seidler K, Neil J. Link between microbiota and hypertension: Focus on LPS/TLR4 pathway in endothelial dysfunction and vascular inflammation, and therapeutic implication of probiotics. Biomed Pharmacother, 2021,137:111334.

[667] Grzelak-Błaszczyk K, Milala J, Kosmala M, et al. Onion quercetin monoglycosides alter microbial activity and increase antioxidant capacity . J Nutr Biochem, 2018,56:81-88.

[668] Guarner-Lans V, Ramírez-Higuera A, Rubio-Ruiz M E, et al. Early Programming of Adult Systemic Essential Hypertension. Int J Mol Sci, 2020,21(4).

[669] Guchelaar H J, Schultz M J, van der Poll T, et al. Pharmacokinetic-pharmacodynamic modeling of the inhibitory effect of erythromycin on tumour necrosis factor-alpha and interleukin-6 production . Fundam Clin Pharmacol, 2001,15(6):419-424.

[670] Gui D D, Luo W, Yan B J, et al. Effects of gut microbiota on atherosclerosis through hydrogen sulfide . Eur J Pharmacol, 2021,896:173916.

[671] Guimarães K, Braga V A, Noronha S, et al. Lactiplantibacillus plantarum WJL administration during pregnancy and lactation improves lipid profile, insulin sensitivity and gut microbiota diversity in dyslipidemic dams and protects male offspring against cardiovascular dysfunction in later life . Food Funct, 2020,11(10):8939-8950.

[672] Guirro M, Costa A, Gual-Grau A, et al. Multi-omics approach to elucidate the gut microbiota activity: Metaproteomics and metagenomics connection . Electrophoresis, 2018,39(13):1692-1701.

[673] Gunasekaran K, Krishnamurthy S, Mahadevan S, et al. Clinical Characteristics and Outcome of Post-Infectious Glomerulonephritis in Children in Southern India: A Prospective Study . Indian J Pediatr, 2015,82(10):896-903.

[674] Gunter E W, Bowman B A, Caudill S P, et al. Results of an international round robin for serum and whole-blood folate. Clin Chem, 1996,42(10):1689-1694.

[675] Guo J, Guo X, Sun Y, et al. Application of omics in hypertension and resistant hypertension. Hypertens Res, 2022.

[676] Guo Y P, Chen M Y, Shao L, et al. Quantification of Panax notoginseng saponins metabolites in rat plasma with in vivo gut microbiota-mediated biotransformation by HPLC-MS/MS . Chin J Nat Med, 2019,17(3):231-240.

[677] Guo Y, Crnkovic C M, Won K J, et al. Commensal Gut Bacteria Convert the Immunosuppressant Tacrolimus to Less Potent Metabolites. Drug Metab Dispos, 2019,47(3):194-202.

[678] Guo Y, Li X, Wang Z, et al. Gut Microbiota Dysbiosis in Human Hypertension: A Systematic Review of Observational Studies . Front Cardiovasc Med, 2021,8:650227.

[679] Gupta A, Agrawal S R, Sivarajan K, et al. A microbiological study of anterior nasal packs in epistaxis. Indian J Otolaryngol Head Neck Surg, 1999,51(1):42-46.

[680] Gurney M A, Laubitz D, Ghishan F K, et al. Pathophysiology of Intestinal Na(+)/H(+) exchange . Cell Mol Gastroenterol Hepatol, 2017,3(1):27-40.

[681] Guss J D, Ziemian S N, Luna M, et al. The effects of metabolic syndrome, obesity, and the gut microbiome on load-induced osteoarthritis . Osteoarthritis Cartilage, 2019,27(1):129-139.

[682] Gustot T, Stadlbauer V, Laleman W, et al. Transition to decompensation and acute-on-chronic liver failure: Role of predisposing factors and precipitating events . J Hepatol, 2021,75 Suppl 1:S36-S48.

[683] Gutiérrez F, Masiá M. Improving outcomes of elderly patients with community-acquired pneumonia. Drugs Aging, 2008,25(7):585-610.

[684] Gutiérrez L, Vargas D, Ocampo L, et al. Plasma concentrations resulting from florfenicol preparations given to pigs in their drinking water . J Anim Sci, 2011,89(9):2926-2931.

[685] Gutiérrez-Cuevas J, Sandoval-Rodriguez A, Meza-Rios A, et al. Molecular Mechanisms of Obesity-Linked Cardiac Dysfunction: An Up-Date on Current Knowledge . Cells, 2021,10(3).

[686] Gvozdenović L, Pasternak J, Milovanović S, et al. [Streptococcal toxic shock syndrome] . Med Pregl, 2010,63(7-8):550-553.

[687] Györik S, Menafoglio A. Images in clinical medicine. Elevated jugular venous pressure. N Engl J Med, 2006,355(11):e10.

[688] Haase S, Wilck N, Haghikia A, et al. The role of the gut microbiota and microbial metabolites in neuroinflammation. Eur J Immunol, 2020,50(12):1863-1870.

[689] Hadavand S, Ghafoorimehr F, Rajabi L, et al. Frequency of Group B Streptococcal Colonization in Pregnant Women Aged 35- 37 Weeks in Clinical Centers of Shahed University, Tehran, Iran . Iran J Pathol, 2015,10(2):120-126.

[690] Haghikia A, Li X S, Liman T G, et al. Gut Microbiota-Dependent Trimethylamine N-Oxide Predicts Risk of Cardiovascular Events in Patients With Stroke and Is Related to Proinflammatory Monocytes . Arterioscler Thromb Vasc Biol, 2018,38(9):2225-2235.

[691] Haitsma J J, Schultz M J, Hofstra J J, et al. Ventilator-induced coagulopathy in experimental Streptococcus pneumoniae pneumonia . Eur Respir J, 2008,32(6):1599-1606.

[692] Halawa A, Kim J, Trobe J D. Retinal, Optic Nerve, and Cerebral Infarction in Odontogenic Lemierre Syndrome. J Neuroophthalmol, 2021.

[693] Haluzík M, Kratochvílová H, Haluzíková D, et al. Gut as an emerging organ for the treatment of diabetes: focus on mechanism of action of bariatric and endoscopic interventions . J Endocrinol, 2018,237(1):R1-R17.

[694] Hamad A, Ozkan M H, Uma S. Trimethylamine-N-oxide (TMAO) Selectively Disrupts Endothelium-Dependent Hyperpolarization-Type Relaxations in a Time-Dependent Manner in Rat Superior Mesenteric Artery . Biol Pharm Bull, 2021,44(9):1220-1229.

[695] Hamilçıkan Ş, Can E, Büke Ö, et al. Pentoxifylline Treatment of Very Low Birth Weight Neonates with Nosocomial Sepsis. Am J Perinatol, 2017,34(8):795-800.

[696] Hamme V, Sannier F, Piot J M, et al. Goat whey fermentation by Kluyveromyces marxianus and Lactobacillus rhamnosus release tryptophan and tryptophan-lactokinin from a cryptic zone of alpha-lactalbumin . J Dairy Res, 2009,76(3):379-383.

[697] Hammerman C, Aramburo M J. Effects of hyperventilation on prostacyclin formation and on pulmonary vasodilation after group B beta-hemolytic streptococci-induced pulmonary hypertension. Pediatr Res, 1991,29(3):282-287.

[698] Hammerman C, Aramburo M J, Choi J H. Prostanoid inhibition and group B hemolytic streptococci (GBS) induced neutropenia in newborn piglets . Prostaglandins Leukot Essent Fatty Acids, 1990,41(2):125-130.

[699] Hammerman C, Aramburo M J, Choi J H. Prostaglandin E1 selectively reduces group B beta-hemolytic streptococci-induced pulmonary hypertension in newborn piglets . Am J Dis Child, 1989,143(3):343-347.

[700] Hammerman C, Komar K, Abu-Khudair H. Hypoxic vs septic pulmonary hypertension. Selective role of thromboxane mediation. Am J Dis Child, 1988,142(3):319-325.

[701] Hammerman C, Komar K, Abu-Khudair H, et al. Oxygen transport in newborn piglets with pulmonary hypertension . Crit Care Med, 1988,16(8):773-778.

[702] Hamoud A R, Weaver L, Stec D E, et al. Bilirubin in the Liver-Gut Signaling Axis . Trends Endocrinol Metab, 2018,29(3):140-150.

[703] Hamouda M, Mrabet I, Dhia N B, et al. Acute post-infectious glomerulonephritis in adults: a single center report . Saudi J Kidney Dis Transpl, 2014,25(3):567-571.

[704] Han C, Jiang Y H, Li W, et al. Study on the Antihypertensive Mechanism of Astragalus membranaceus and Salvia miltiorrhiza Based on Intestinal Flora-Host Metabolism . Evid Based Complement Alternat Med, 2019,2019:5418796.

[705] Hancock-Allen J B, Janelle S J, Lujan K, et al. Outbreak of group A Streptococcus infections in an outpatient wound clinic-Colorado, 2014 . Am J Infect Control, 2016,44(10):1133-1138.

[706] Hänninen, Kaartinen K, Rauma A L, et al. Antioxidants in vegan diet and rheumatic disorders . Toxicology, 2000,155(1-3):45-53.

[707] Hantson P, Duprez T. Late Recovery from Severe Streptococcus pneumoniae Comatose Meningitis with Concomitant Diffuse Subcortical Cytotoxic Edema and Cortical Hypometabolism . Case Rep Neurol Med, 2018,2018:9439021.

[708] Hao Y, Wang Y, Xi L, et al. A Nested Case-Control Study of Association between Metabolome and Hypertension Risk. Biomed Res Int, 2016,2016:7646979.

[709] Hara H, Mihara M, Todokoro T. Necrotizing Fasciitis Occurred in the Lymphedematous leg. Int J Low Extrem Wounds, 2021:2133272758.

[710] Harikumar R, Pramod K, Pushpa M, et al. Gastric lymphoma presenting as phlegmonous gastritis. J Gastrointest Cancer, 2007,38(1):24-27.

[711] Harrison D G, Coffman T M, Wilcox C S. Pathophysiology of Hypertension: The Mosaic Theory and Beyond. Circ Res, 2021,128(7):847-863.

[712] Harrison R K, Egede L E, Palatnik A. Peripartum infectious morbidity in women with preeclampsia. J Matern Fetal Neonatal Med, 2021,34(8):1215-1220.

[713] Haruta M, Yoshida Y, Yamakawa R. Pediatric endogenous Haemophilus influenzae endophthalmitis with presumed hyposplenism . Int Med Case Rep J, 2017,10:7-9.

[714] Hasegawa T, Hashikawa S N, Nakamura T, et al. Factors determining prognosis in streptococcal toxic shock-like syndrome: results of a nationwide investigation in Japan . Microbes Infect, 2004,6(12):1073-1077.

[715] Hashimoto T, Sato H, Shikata F, et al. Response by Hashimoto et al to Letter Regarding Article "Potential Influences of Gut Microbiota on the Formation of Intracranial Aneurysm" . Hypertension, 2019,74(2):e24-e25.

[716] Hashimoto Y, Nakajima H, Hata S, et al. Effect of probiotics, Bifidobacterium bifidum G9-1, on gastrointestinal symptoms in patients with type 2 diabetes mellitus: study protocol for open-label, single-arm, exploratory research trial (Big STAR study) . J Clin Biochem Nutr, 2020,67(3):223-227.

[717] Hassan A W. A study of abnormal cannabidiols system-mediated cardiovascular protection in disrupted gut/brain axis associated depression . J Biochem Mol Toxicol, 2021,35(12):e22930.

[718] Hassan M, Moghadamrad S, Sorribas M, et al. Paneth cells promote angiogenesis and regulate portal hypertension in response to microbial signals . J Hepatol, 2020,73(3):628-639.

[719] Hauck W, Samlalsingh-Parker J, Glibetic M, et al. Deregulation of cyclooxygenase and nitric oxide synthase gene expression in the inflammatory cascade triggered by experimental group B streptococcal meningitis in the newborn brain and cerebral microvessels . Semin Perinatol, 1999,23(3):250-260.

[720] Hautaniemi E J, Tikkakoski A J, Tahvanainen A, et al. Effect of fermented milk product containing lactotripeptides and plant sterol esters on haemodynamics in subjects with the metabolic syndrome--a randomised, double-blind, placebo-controlled study . Br J Nutr, 2015,114(3):376-386.

[721] Hawiger J. Heartfelt sepsis: microvascular injury due to genomic storm . Kardiol Pol, 2018,76(8):1203-1216.

[722] Hayes M, Ross R P, Fitzgerald G F, et al. Putting microbes to work: dairy fermentation, cell factories and bioactive peptides. Part I: overview. Biotechnol J, 2007,2(4):426-434.

[723] Hayes M, Stanton C, Fitzgerald G F, et al. Putting microbes to work: dairy fermentation, cell factories and bioactive peptides. Part II: bioactive peptide functions. Biotechnol J, 2007,2(4):435-449.

[724] He F, Li Y. The gut microbial composition in polycystic ovary syndrome with insulin resistance: findings from a normal-weight population. J Ovarian Res, 2021,14(1):50.

[725] He J, Zhang F, Han Y. Effect of probiotics on lipid profiles and blood pressure in patients with type 2 diabetes: A meta-analysis of RCTs . Medicine (Baltimore), 2017,96(51):e9166.

[726] He M, Shi B. Gut microbiota as a potential target of metabolic syndrome: the role of probiotics and prebiotics. Cell Biosci, 2017,7:54.

[727] He S, Jiang H, Zhuo C, et al. Trimethylamine/Trimethylamine-N-Oxide as a Key Between Diet and Cardiovascular Diseases. Cardiovasc Toxicol, 2021,21(8):593-604.

[728] He Y J, You C G. The Potential Role of Gut Microbiota in the Prevention and Treatment of Lipid Metabolism Disorders . Int J Endocrinol, 2020,2020:8601796.

[729] Heidari V, Habibi Z, Hojjati M A, et al. Different Behavior and Response of Staphylococcus Epidermidis and Streptococcus Pneumoniae to a Ventriculoperitoneal Shunt: An in vitro Study . Pediatr Neurosurg, 2017,52(4):257-260.

[730] Heidari Z, Rashidi P F N, Clark C, et al. Dairy products consumption and the risk of hypertension in adults: An updated systematic review and dose-response meta-analysis of prospective cohort studies . Nutr Metab Cardiovasc Dis, 2021,31(7):1962-1975.

[731] Heinig K, Wirz T, Schick E, et al. Bioanalysis of therapeutic peptides: differentiating between total and anti-drug antibody bound drug using liquid chromatography-tandem mass spectrometry quantitation . J Chromatogr A, 2013,1316:69-77.

[732] Hellerqvist C G, Rojas J, Green R S, et al. Studies on group B beta-hemolytic Streptococcus. I. Isolation and partial characterization of an extracellular toxin. Pediatr Res, 1981,15(6):892-898.

[733] Hellerqvist C G, Sundell H, Gettins P. Molecular basis for group B beta-hemolytic streptococcal disease . Proc Natl Acad Sci U S A, 1987,84(1):51-55.

[734] Hemming V G, O'Brien W F, Fischer G W, et al. Studies of short-term pulmonary and peripheral vascular responses induced in oophorectomized sheep by the infusion of a group B streptococcal extract . Pediatr Res, 1984,18(3):266-269.

[735] Henao-Mejia J, Elinav E, Jin C, et al. Inflammasome-mediated dysbiosis regulates progression of NAFLD and obesity. Nature, 2012,482(7384):179-185.

[736] Hendijani F, Akbari V. Probiotic supplementation for management of cardiovascular risk factors in adults with type II diabetes: A systematic review and meta-analysis . Clin Nutr, 2018,37(2):532-541.

[737] Hendy P, Ding N. Probiotics for secondary prevention of hepatic encephalopathy. Frontline Gastroenterol, 2015,6(4):230-231.

[738] Henninger J, Eliasson B, Smith U, et al. Identification of markers that distinguish adipose tissue and glucose and insulin metabolism using a multi-modal machine learning approach . Sci Rep, 2021,11(1):17050.

[739] Hernandez-Rodas M C, Valenzuela R, Videla L A. Relevant Aspects of Nutritional and Dietary Interventions in Non-Alcoholic Fatty Liver Disease . Int J Mol Sci, 2015,16(10):25168-25198.

[740] Herzberg M C, Meyer M W. Effects of oral flora on platelets: possible consequences in cardiovascular disease. J Periodontol, 1996,67(10 Suppl):1138-1142.

[741] Herzberg M C, Meyer M W. Effects of Oral Flora on Platelets: Possible Consequences in Cardiovascular Disease. J Periodontol, 1996,67 Suppl 10S:1138-1142.

[742] Herzberg M C, Weyer M W. Dental plaque, platelets, and cardiovascular diseases . Ann Periodontol, 1998,3(1):151-160.

[743] Hess A L, Benítez-Páez A, Blædel T, et al. The effect of inulin and resistant maltodextrin on weight loss during energy restriction: a randomised, placebo-controlled, double-blinded intervention. Eur J Nutr, 2020,59(6):2507-2524.

[744] Hid E J, Mosele J I, Prince P D, et al. ( -)-Epicatechin and cardiometabolic risk factors: a focus on potential mechanisms of action . Pflugers Arch, 2022,474(1):99-115.

[745] Hildebrandt T, Scheuch E, Weitschies W, et al. Measurement of abomasal conditions (pH, pressure and temperature) in healthy and diarrheic dairy calves using a wireless ambulatory capsule . Livest Sci, 2017,203:41-47.

[746] Hinojosa-Nogueira D, Pérez-Burillo S, Pastoriza D L C S, et al. Green and white teas as health-promoting foods . Food Funct, 2021,12(9):3799-3819.

[747] Hinrichsen F, Hamm J, Westermann M, et al. Microbial regulation of hexokinase 2 links mitochondrial metabolism and cell death in colitis . Cell Metab, 2021,33(12):2355-2366.

[748] Hirose K, Okabe H, Yoshizumi T, et al. A case report of bacteremia manifesting as an overwhelming postsplenectomy infection due to Streptococcus pneumoniae post vaccination . Surg Case Rep, 2016,2(1):48.

[749] Hirota T, Nonaka A, Matsushita A, et al. Milk casein-derived tripeptides, VPP and IPP induced NO production in cultured endothelial cells and endothelium-dependent relaxation of isolated aortic rings . Heart Vessels, 2011,26(5):549-556.

[750] Hmami F, Oulmaati A, Mahmoud M, et al. [Neonatal group A streptococcal meningitis and portal vein thrombosis: a casual association?]. Arch Pediatr, 2014,21(9):1020-1023.

[751] Hobbs J K, Meier E, Pluvinage B, et al. Molecular analysis of an enigmatic Streptococcus pneumoniae virulence factor: The raffinose-family oligosaccharide utilization system . J Biol Chem, 2019,294(46):17197-17208.

[752] Hoehn T, Huebner J, Paboura E, et al. Effect of therapeutic concentrations of nitric oxide on bacterial growth in vitro . Crit Care Med, 1998,26(11):1857-1862.

[753] Hoffmann O M, Becker D, Weber J R. Bacterial hydrogen peroxide contributes to cerebral hyperemia during early stages of experimental pneumococcal meningitis . J Cereb Blood Flow Metab, 2007,27(11):1792-1797.

[754] Hoffmann O, Braun J S, Becker D, et al. TLR2 mediates neuroinflammation and neuronal damage. J Immunol, 2007,178(10):6476-6481.

[755] Högman C F, Gong J. Studies of one invasive and two noninvasive methods for detection of bacterial contamination of platelet concentrates. Vox Sang, 1994,67(4):351-355.

[756] Holm S E, Jönsson J, Braun D. Presence of a vaso-active factor in a nephritogenic group A, type 12 streptococcal strain . Acta Pathol Microbiol Scand, 1967,69(4):549-556.

[757] Hong W, Mo Q, Wang L, et al. Changes in the gut microbiome and metabolome in a rat model of pulmonary arterial hypertension . Bioengineered, 2021,12(1):5173-5183.

[758] Honour J W. Historical perspective: gut dysbiosis and hypertension. Physiol Genomics, 2015,47(10):443-446.

[759] Hord N G. Eukaryotic-microbiota crosstalk: potential mechanisms for health benefits of prebiotics and probiotics . Annu Rev Nutr, 2008,28:215-231.

[760] Horne D W, Patterson D, Said H M. Aging: effect on hepatic metabolism and transport of folate in the rat. Am J Clin Nutr, 1989,50(2):359-363.

[761] Hoshal V J. Intravenous catheters and infection . Surg Clin North Am, 1972,52(6):1407-1417.

[762] Hosoki S, Saito S, Tonomura S, et al. Oral Carriage of Streptococcus mutans Harboring the cnm Gene Relates to an Increased Incidence of Cerebral Microbleeds . Stroke, 2020,51(12):3632-3639.

[763] Hove-Skovsgaard M, Gaardbo J C, Kolte L, et al. HIV-infected persons with type 2 diabetes show evidence of endothelial dysfunction and increased inflammation . BMC Infect Dis, 2017,17(1):234.

[764] Hoyles L, Jiménez-Pranteda M L, Chilloux J, et al. Metabolic retroconversion of trimethylamine N-oxide and the gut microbiota . Microbiome, 2018,6(1):73.

[765] Hrncir T, Hrncirova L, Kverka M, et al. Gut Microbiota and NAFLD: Pathogenetic Mechanisms, Microbiota Signatures, and Therapeutic Interventions. Microorganisms, 2021,9(5).

[766] Hsu C N, Chan J, Wu K, et al. Altered Gut Microbiota and Its Metabolites in Hypertension of Developmental Origins: Exploring Differences between Fructose and Antibiotics Exposure . Int J Mol Sci, 2021,22(5).

[767] Hsu C N, Chan J, Yu H R, et al. Targeting on Gut Microbiota-Derived Metabolite Trimethylamine to Protect Adult Male Rat Offspring against Hypertension Programmed by Combined Maternal High-Fructose Intake and Dioxin Exposure . Int J Mol Sci, 2020,21(15).

[768] Hsu C N, Chang-Chien G P, Lin S, et al. Targeting on Gut Microbial Metabolite Trimethylamine-N-Oxide and Short-Chain Fatty Acid to Prevent Maternal High-Fructose-Diet-Induced Developmental Programming of Hypertension in Adult Male Offspring . Mol Nutr Food Res, 2019,63(18):e1900073.

[769] Hsu C N, Hou C Y, Chan J, et al. Hypertension Programmed by Perinatal High-Fat Diet: Effect of Maternal Gut Microbiota-Targeted Therapy . Nutrients, 2019,11(12).

[770] Hsu C N, Hou C Y, Chang-Chien G P, et al. Maternal resveratrol therapy protected adult rat offspring against hypertension programmed by combined exposures to asymmetric dimethylarginine and trimethylamine-N-oxide . J Nutr Biochem, 2021,93:108630.

[771] Hsu C N, Hou C Y, Chang-Chien G P, et al. Maternal N-Acetylcysteine Therapy Prevents Hypertension in Spontaneously Hypertensive Rat Offspring: Implications of Hydrogen Sulfide-Generating Pathway and Gut Microbiota . Antioxidants (Basel), 2020,9(9).

[772] Hsu C N, Hou C Y, Hsu W H, et al. Cardiovascular Diseases of Developmental Origins: Preventive Aspects of Gut Microbiota-Targeted Therapy . Nutrients, 2021,13(7).

[773] Hsu C N, Hou C Y, Hsu W H, et al. Early-Life Origins of Metabolic Syndrome: Mechanisms and Preventive Aspects . Int J Mol Sci, 2021,22(21).

[774] Hsu C N, Hou C Y, Lee C T, et al. The Interplay between Maternal and Post-Weaning High-Fat Diet and Gut Microbiota in the Developmental Programming of Hypertension . Nutrients, 2019,11(9).

[775] Hsu C N, Hou C Y, Lee C T, et al. Maternal 3,3-Dimethyl-1-Butanol Therapy Protects Adult Male Rat Offspring against Hypertension Programmed by Perinatal TCDD Exposure . Nutrients, 2021,13(9).

[776] Hsu C N, Hung C H, Hou C Y, et al. Perinatal Resveratrol Therapy to Dioxin-Exposed Dams Prevents the Programming of Hypertension in Adult Rat Offspring . Antioxidants (Basel), 2021,10(9).

[777] Hsu C N, Lin Y J, Hou C Y, et al. Maternal Administration of Probiotic or Prebiotic Prevents Male Adult Rat Offspring against Developmental Programming of Hypertension Induced by High Fructose Consumption in Pregnancy and Lactation . Nutrients, 2018,10(9).

[778] Hsu C N, Tain Y L. Amino Acids and Developmental Origins of Hypertension. Nutrients, 2020,12(6).

[779] Hsu C N, Tain Y L. Animal Models for DOHaD Research: Focus on Hypertension of Developmental Origins. Biomedicines, 2021,9(6).

[780] Hsu C N, Tain Y L. Preventing Developmental Origins of Cardiovascular Disease: Hydrogen Sulfide as a Potential Target? . Antioxidants (Basel), 2021,10(2).

[781] Hsu C N, Yu H R, Chan J, et al. The Impact of Gut Microbiome on Maternal Fructose Intake-Induced Developmental Programming of Adult Disease . Nutrients, 2022,14(5).

[782] Hsu J F, Chu S M, Huang Y C, et al. Predictors of clinical and microbiological treatment failure in neonatal bloodstream infections . Clin Microbiol Infect, 2015,21(5):482-489.

[783] Hu C, Wang P, Yang Y, et al. Chronic Intermittent Hypoxia Participates in the Pathogenesis of Atherosclerosis and Perturbs the Formation of Intestinal Microbiota . Front Cell Infect Microbiol, 2021,11:560201.

[784] Hu D K, Liu Y, Li X Y, et al. In vitro expression of Streptococcus pneumoniae ply gene in human monocytes and pneumocytes . Eur J Med Res, 2015,20(1):52.

[785] Hu W, Jin R, Zhang J, et al. The critical roles of platelet activation and reduced NO bioavailability in fatal pulmonary arterial hypertension in a murine hemolysis model . Blood, 2010,116(9):1613-1622.

[786] Hu X F, Zhang W Y, Wen Q, et al. Fecal microbiota transplantation alleviates myocardial damage in myocarditis by restoring the microbiota composition . Pharmacol Res, 2019,139:412-421.

[787] Hu X, Zhang H, Lu H, et al. The Effect of Probiotic Treatment on Patients Infected with the H7N9 Influenza Virus. PLoS One, 2016,11(3):e151976.

[788] Huang H W, Hsu C P, Wang C Y. Healthy expectations of high hydrostatic pressure treatment in food processing industry . J Food Drug Anal, 2020,28(1):1-13.

[789] Huang J, Liao J, Fang Y, et al. Six-Week Exercise Training With Dietary Restriction Improves Central Hemodynamics Associated With Altered Gut Microbiota in Adolescents With Obesity . Front Endocrinol (Lausanne), 2020,11:569085.

[790] Huang L, Cai M, Li L, et al. Gut microbiota changes in preeclampsia, abnormal placental growth and healthy pregnant women . BMC Microbiol, 2021,21(1):265.

[791] Huang M, Zhu L, Jin Y, et al. Association between Helicobacter Pylori Infection and Systemic Arterial Hypertension: A Meta-Analysis. Arq Bras Cardiol, 2021,117(4):626-636.

[792] Huang Y C, Huang L T, Sheen J M, et al. Resveratrol treatment improves the altered metabolism and related dysbiosis of gut programed by prenatal high-fat diet and postnatal high-fat diet exposure . J Nutr Biochem, 2020,75:108260.

[793] Huang Y H, Lin T Y, Wong K S, et al. Hemolytic uremic syndrome associated with pneumococcal pneumonia in Taiwan . Eur J Pediatr, 2006,165(5):332-335.

[794] Huang Y, Lin F, Tang R, et al. Gut Microbial Metabolite Trimethylamine N-Oxide Aggravates Pulmonary Hypertension. Am J Respir Cell Mol Biol, 2022,66(4):452-460.

[795] Huang Y, Zheng H, Tan K, et al. Circulating metabolomics profiling reveals novel pathways associated with cognitive decline in patients with hypertension . Ther Adv Neurol Disord, 2020,13:1279169541.

[796] Huart J, Cirillo A, Saint-Remy A, et al. The faecal abundance of short chain fatty acids is increased in men with a non-dipping blood pressure profile . Acta Cardiol, 2021:1-4.

[797] Huart J, Cirillo A, Taminiau B, et al. Human Stool Metabolome Differs upon 24 h Blood Pressure Levels and Blood Pressure Dipping Status: A Prospective Longitudinal Study . Metabolites, 2021,11(5).

[798] Huart J, Krzesinski J M, Jouret F. [Arguments for a role of the gut microbiota in the pathophysiology of hypertension]. Rev Med Liege, 2020,75(9):588-592.

[799] Huart J, Leenders J, Taminiau B, et al. Gut Microbiota and Fecal Levels of Short-Chain Fatty Acids Differ Upon 24-Hour Blood Pressure Levels in Men . Hypertension, 2019,74(4):1005-1013.

[800] Huddleston K W, Lyrene R K, Dew A, et al. Influence of prostaglandin D2 on hemodynamic effects of group B streptococcus in neonatal lambs . Dev Pharmacol Ther, 1986,9(4):260-265.

[801] Hüsing-Kabar A, Meister T, Köhler M, et al. Is de novo hepatocellular carcinoma after transjugular intrahepatic portosystemic shunt increased? . United European Gastroenterol J, 2018,6(3):413-421.

[802] Hussin F S, Chay S Y, Hussin A, et al. GABA enhancement by simple carbohydrates in yoghurt fermented using novel, self-cloned Lactobacillus plantarum Taj-Apis362 and metabolomics profiling . Sci Rep, 2021,11(1):9417.

[803] Hütt P, Songisepp E, Rätsep M, et al. Impact of probiotic Lactobacillus plantarum TENSIA in different dairy products on anthropometric and blood biochemical indices of healthy adults . Benef Microbes, 2015,6(3):233-243.

[804] Huttunen R, Hurme M, Laine J, et al. Endothelial nitric oxide synthase G894T (GLU298ASP) polymorphism is associated with hypotension in patients with E. coli bacteremia but not in bacteremia caused by a gram-positive organism . Shock, 2009,31(5):448-453.

[805] Huttunen R, Syrjänen J, Aittoniemi J, et al. High activity of indoleamine 2,3 dioxygenase enzyme predicts disease severity and case fatality in bacteremic patients . Shock, 2010,33(2):149-154.

[806] Huttunen R, Syrjänen J, Vuento R, et al. Plasma level of soluble urokinase-type plasminogen activator receptor as a predictor of disease severity and case fatality in patients with bacteraemia: a prospective cohort study . J Intern Med, 2011,270(1):32-40.

[807] Hyde E R, Luk B, Cron S, et al. Characterization of the rat oral microbiome and the effects of dietary nitrate . Free Radic Biol Med, 2014,77:249-257.

[808] Ibrahim N S, Ooi F K, Chen C K, et al. Effects of probiotics supplementation and circuit training on immune responses among sedentary young males . J Sports Med Phys Fitness, 2018,58(7-8):1102-1109.

[809] Ihara M, Yamamoto Y. Emerging Evidence for Pathogenesis of Sporadic Cerebral Small Vessel Disease. Stroke, 2016,47(2):554-560.

[810] Imani F A, Mahmoodzadeh H H, Nourani M R, et al. Probiotic as a novel treatment strategy against liver disease . Hepat Mon, 2013,13(2):e7521.

[811] Inenaga C, Hokamura K, Nakano K, et al. A Potential New Risk Factor for Stroke: Streptococcus Mutans With Collagen-Binding Protein . World Neurosurg, 2018,113:e77-e81.

[812] Íñiguez M, Pérez-Matute P, Villoslada-Blanco P, et al. ACE Gene Variants Rise the Risk of Severe COVID-19 in Patients With Hypertension, Dyslipidemia or Diabetes: A Spanish Pilot Study . Front Endocrinol (Lausanne), 2021,12:688071.

[813] Íñiguez M, Pérez-Matute P, Villoslada-Blanco P, et al. Corrigendum: ACE Gene Variants Rise the Risk of Severe COVID-19 in Patients With Hypertension, Dyslipidemia or Diabetes: A Spanish Pilot Study . Front Endocrinol (Lausanne), 2021,12:771445.

[814] Inoue K, Shirai T, Ochiai H, et al. Blood-pressure-lowering effect of a novel fermented milk containing gamma-aminobutyric acid (GABA) in mild hypertensives . Eur J Clin Nutr, 2003,57(3):490-495.

[815] Inoue R, Ohue-Kitano R, Tsukahara T, et al. Prediction of functional profiles of gut microbiota from 16S rRNA metagenomic data provides a more robust evaluation of gut dysbiosis occurring in Japanese type 2 diabetic patients . J Clin Biochem Nutr, 2017,61(3):217-221.

[816] Irazuzta J E, Pretzlaff R, Rowin M, et al. Hypothermia as an adjunctive treatment for severe bacterial meningitis. Brain Res, 2000,881(1):88-97.

[817] Isab A A, Wazeer M I. Solid and solution NMR studies of the complexation of Ag+ with the trans isomer of captopril: biological activities of this high blood pressure drug along with its Ag+ complex . Spectrochim Acta A Mol Biomol Spectrosc, 2006,65(1):191-195.

[818] Iseri K, Iyoda M, Yamamoto Y, et al. Streptococcal Infection-related Nephritis (SIRN) Manifesting Membranoproliferative Glomerulonephritis Type I . Intern Med, 2016,55(6):647-650.

[819] İşeri N M, Dinleyici M, Kılıç Ö, et al. [Meningitis Due to Streptococcus pneumoniae Serotype 24B in a Patient with Cochlear Implant Previously Vaccinated with the Pneumococcal Vaccine] . Mikrobiyol Bul, 2019,53(4):451-456.

[820] Ishikawa T, Zhu B L, Li D R, et al. An autopsy case of internal jugular vein thrombophlebitis involving sepsis following blunt neck injury. J Forensic Leg Med, 2008,15(2):114-117.

[821] Ishimwe J A. Maternal microbiome in preeclampsia pathophysiology and implications on offspring health . Physiol Rep, 2021,9(10):e14875.

[822] Ishimwe J A, Akinleye A, Johnson A C, et al. Gestational gut microbial remodeling is impaired in a rat model of preeclampsia superimposed on chronic hypertension . Physiol Genomics, 2021,53(3):125-136.

[823] Ishimwe J A, Dola T, Ertuglu L A, et al. Bile acids and salt-sensitive hypertension: a role of the gut-liver axis . Am J Physiol Heart Circ Physiol, 2022,322(4):H636-H646.

[824] Ishiyama Y, Hoshide S, Mizuno H, et al. Constipation-induced pressor effects as triggers for cardiovascular events . J Clin Hypertens (Greenwich), 2019,21(3):421-425.

[825] Ismael S, Silvestre M P, Vasques M, et al. A Pilot Study on the Metabolic Impact of Mediterranean Diet in Type 2 Diabetes: Is Gut Microbiota the Key? . Nutrients, 2021,13(4).

[826] Ito A, Ishida T, Tachibana H, et al. Predictors and usefulness of targeted therapy for pneumococcal community-acquired pneumonia diagnosed by the urinary antigen test: a prospective, observational cohort study . Diagn Microbiol Infect Dis, 2021,101(1):115457.

[827] Ito M, Kusuhara S, Yokoi W, et al. Streptococcus thermophilus fermented milk reduces serum MDA-LDL and blood pressure in healthy and mildly hypercholesterolaemic adults . Benef Microbes, 2017,8(2):171-178.

[828] Ivey K L, Chan A T, Izard J, et al. Role of Dietary Flavonoid Compounds in Driving Patterns of Microbial Community Assembly . mBio, 2019,10(5).

[829] Ivey K L, Hodgson J M, Kerr D A, et al. The effect of yoghurt and its probiotics on blood pressure and serum lipid profile; a randomised controlled trial . Nutr Metab Cardiovasc Dis, 2015,25(1):46-51.

[830] Izumi T, Hyodo T, Kikuchi Y, et al. An adult with acute poststreptococcal glomerulonephritis complicated by hemolytic uremic syndrome and nephrotic syndrome . Am J Kidney Dis, 2005,46(4):e59-e63.

[831] Jääskeläinen T, Kärkkäinen O, Heinonen S, et al. No association in maternal serum levels of TMAO and its precursors in pre-eclampsia and in non-complicated pregnancies . Pregnancy Hypertens, 2022,28:74-80.

[832] Jabczyk M, Nowak J, Hudzik B, et al. Microbiota and Its Impact on the Immune System in COVID-19-A Narrative Review. J Clin Med, 2021,10(19).

[833] Jackson N R, Zeigler K, Torrez M, et al. New Mexico's COVID-19 Experience . Am J Forensic Med Pathol, 2021,42(1):1-8.

[834] Jacobs R F, Sowell M K, Moss M M, et al. Septic shock in children: bacterial etiologies and temporal relationships . Pediatr Infect Dis J, 1990,9(3):196-200.

[835] Jaffee I S. Adenotonsillectomy as the treatment of serious medical conditions: five case reports . Laryngoscope, 1974,84(7):1135-1141.

[836] Jagau H, Behrens I K, Steinert M, et al. Pneumococcus Infection of Primary Human Endothelial Cells in Constant Flow. J Vis Exp, 2019(152).

[837] Jahn S, Bauer B, Schwab J, et al. Immune restoration in children after partial splenectomy . Immunobiology, 1993,188(4-5):370-378.

[838] Jaimee G, Halami P M. Emerging resistance to aminoglycosides in lactic acid bacteria of food origin-an impending menace . Appl Microbiol Biotechnol, 2016,100(3):1137-1151.

[839] Jain S, Mankad S V. Echocardiographic assessment of mitral stenosis: echocardiographic features of rheumatic mitral stenosis . Cardiol Clin, 2013,31(2):177-191.

[840] Jäkälä P, Vapaatalo H. Antihypertensive Peptides from Milk Proteins . Pharmaceuticals (Basel), 2010,3(1):251-272.

[841] Jakobsdottir G, Nyman M, Fåk F. Designing future prebiotic fiber to target metabolic syndrome. Nutrition, 2014,30(5):497-502.

[842] Jama H A, Beale A, Shihata W A, et al. The effect of diet on hypertensive pathology: is there a link via gut microbiota-driven immunometabolism?. Cardiovasc Res, 2019,115(9):1435-1447.

[843] Jama H A, Fiedler A, Tsyganov K, et al. Manipulation of the gut microbiota by the use of prebiotic fibre does not override a genetic predisposition to heart failure . Sci Rep, 2020,10(1):17919.

[844] Jama H A, Kaye D M, Marques F Z. The gut microbiota and blood pressure in experimental models. Curr Opin Nephrol Hypertens, 2019,28(2):97-104.

[845] Jama H A, Marques F Z. Don't Take It With a Pinch of Salt: How Sodium Increases Blood Pressure via the Gut Microbiota. Circ Res, 2020,126(7):854-856.

[846] Jama H A, Muralitharan R R, Xu C, et al. Rodent models of hypertension. Br J Pharmacol, 2022,179(5):918-937.

[847] Jama H, Kaye D M, Marques F Z. Population-Based Gut Microbiome Associations With Hypertension. Circ Res, 2018,123(11):1185-1187.

[848] Jammal M H, Guidon J, Chiche L, et al. [Salmonella bredney: a rare cause of mycotic aneurysm] . Rev Med Interne, 2011,32(1):e12-e14.

[849] Jaramillo A, Contreras A, Lafaurie G I, et al. Association of metabolic syndrome and chronic periodontitis in Colombians. Clin Oral Investig, 2017,21(5):1537-1544.

[850] Jauhiainen T, Korpela R. Milk peptides and blood pressure . J Nutr, 2007,137(3 Suppl 2):825S-829S.

[851] Jauhiainen T, Vapaatalo H, Poussa T, et al. Lactobacillus helveticus fermented milk lowers blood pressure in hypertensive subjects in 24-h ambulatory blood pressure measurement . Am J Hypertens, 2005,18(12 Pt 1):1600-1605.

[852] Javaudin F, Desce N, Le Bastard Q, et al. Impact of pre-hospital vital parameters on the neurological outcome of out-of-hospital cardiac arrest: Results from the French National Cardiac Arrest Registry . Resuscitation, 2018,133:5-11.

[853] Jawaro T, Yang A, Dixit D, et al. Management of Hepatic Encephalopathy: A Primer . Ann Pharmacother, 2016,50(7):569-577.

[854] Jaworska K, Bielinska K, Gawrys-Kopczynska M, et al. TMA (trimethylamine), but not its oxide TMAO (trimethylamine-oxide), exerts haemodynamic effects: implications for interpretation of cardiovascular actions of gut microbiome . Cardiovasc Res, 2019,115(14):1948-1949.

[855] Jaworska K, Huc T, Gawrys M, et al. An In Vivo Method for Evaluating the Gut-Blood Barrier and Liver Metabolism of Microbiota Products . J Vis Exp, 2018(140).

[856] Jenkins D J, Kendall C W, Hamidi M, et al. Effect of antibiotics as cholesterol-lowering agents. Metabolism, 2005,54(1):103-112.

[857] Jennings A, Koch M, Bang C, et al. Microbial Diversity and Abundance of Parabacteroides Mediate the Associations Between Higher Intake of Flavonoid-Rich Foods and Lower Blood Pressure . Hypertension, 2021,78(4):1016-1026.

[858] Jensen A B, Ajslev T A, Brunak S, et al. Long-term risk of cardiovascular and cerebrovascular disease after removal of the colonic microbiota by colectomy: a cohort study based on the Danish National Patient Register from 1996 to 2014 . BMJ Open, 2015,5(12):e8702.

[859] Jensen E T, Bertoni A G, Crago O L, et al. Rationale, design and baseline characteristics of the Microbiome and Insulin Longitudinal Evaluation Study (MILES) . Diabetes Obes Metab, 2020,22(11):1976-1984.

[860] Jeznach-Steinhagen A, Ostrowska J, Czerwonogrodzka-Senczyna A, et al. Dietary and Pharmacological Treatment of Nonalcoholic Fatty Liver Disease . Medicina (Kaunas), 2019,55(5).

[861] Ji Y, Chung Y M, Park S, et al. Dose-dependent and strain-dependent anti-obesity effects of Lactobacillus sakei in a diet induced obese murine model . PeerJ, 2019,7:e6651.

[862] Jia G, Sowers J R. Hypertension in Diabetes: An Update of Basic Mechanisms and Clinical Disease. Hypertension, 2021,78(5):1197-1205.

[863] Jia Q, Xie Y, Lu C, et al. Endocrine organs of cardiovascular diseases: Gut microbiota. J Cell Mol Med, 2019,23(4):2314-2323.

[864] Jia W, Zhen J, Liu A, et al. Long-Term Vegan Meditation Improved Human Gut Microbiota. Evid Based Complement Alternat Med, 2020,2020:9517897.

[865] Jiang Q, Chen Q, Zhang T, et al. The Antihypertensive Effects and Potential Molecular Mechanism of Microalgal Angiotensin I-Converting Enzyme Inhibitor-Like Peptides: A Mini Review . Int J Mol Sci, 2021,22(8).

[866] Jiang S, Shui Y, Cui Y, et al. Gut microbiota dependent trimethylamine N-oxide aggravates angiotensin II-induced hypertension . Redox Biol, 2021,46:102115.

[867] Jiang X, Li X, Zhu C, et al. The target cells of anthocyanins in metabolic syndrome. Crit Rev Food Sci Nutr, 2019,59(6):921-946.

[868] Jin L, Shi X, Yang J, et al. Gut microbes in cardiovascular diseases and their potential therapeutic applications . Protein Cell, 2021,12(5):346-359.

[869] Jin M, Qian Z, Yin J, et al. The role of intestinal microbiota in cardiovascular disease. J Cell Mol Med, 2019,23(4):2343-2350.

[870] Jing C, Xiao N, Yu H J, et al. [Effect of umbilical moxibustion on phlegm damp constitution and intestinal flora]. Zhongguo Zhen Jiu, 2021,41(12):1360-1364.

[871] Jing Y, Zhou H, Lu H, et al. Associations Between Peripheral Blood Microbiome and the Risk of Hypertension. Am J Hypertens, 2021,34(10):1064-1070.

[872] Jmeian Y, El R Z. Tandem affinity monolithic microcolumns with immobilized protein A, protein G', and antibodies for depletion of high abundance proteins from serum samples: integrated microcolumn-based fluidic system for simultaneous depletion and tryptic digestion . J Proteome Res, 2007,6(3):947-954.

[873] Jochberger S, Luckner G, Mayr V D, et al. Course of vasopressin and copeptin plasma concentrations in a patient with severe septic shock . Anaesth Intensive Care, 2006,34(4):498-500.

[874] Johansson I, Nilsson L M, Esberg A, et al. Dairy intake revisited - associations between dairy intake and lifestyle related cardio-metabolic risk factors in a high milk consuming population . Nutr J, 2018,17(1):110.

[875] John O D, du Preez R, Panchal S K, et al. Tropical foods as functional foods for metabolic syndrome . Food Funct, 2020,11(8):6946-6960.

[876] Johnson J R, Colombo D F, Gardner D, et al. Optimal dosing of penicillin G in the third trimester of pregnancy for prophylaxis against group B Streptococcus . Am J Obstet Gynecol, 2001,185(4):850-853.

[877] Johnson P, Calhoun D M, Stokes A N, et al. Of poisons and parasites-the defensive role of tetrodotoxin against infections in newts . J Anim Ecol, 2018,87(4):1192-1204.

[878] Johnson S. Clostridial constipation's broad pathology . Med Hypotheses, 2001,56(4):532-536.

[879] Johnston C, Godecker A, Shirley D, et al. Documented β-Lactam Allergy and Risk for Cesarean Surgical Site Infection. Infect Dis Obstet Gynecol, 2022,2022:5313948.

[880] Johri A M, Heyland D K, Hétu M F, et al. Carnitine therapy for the treatment of metabolic syndrome and cardiovascular disease: evidence and controversies . Nutr Metab Cardiovasc Dis, 2014,24(8):808-814.

[881] Joo S S, Won T J, Nam S Y, et al. Therapeutic advantages of medicinal herbs fermented with Lactobacillus plantarum, in topical application and its activities on atopic dermatitis . Phytother Res, 2009,23(7):913-919.

[882] Jordan J, Moeller R, Chakraborty S, et al. Pressure From the Bugs Within . Hypertension, 2019,73(5):977-979.

[883] Jose P A, Raj D. Gut microbiota in hypertension. Curr Opin Nephrol Hypertens, 2015,24(5):403-409.

[884] Joshi S R, Standl E, Tong N, et al. Therapeutic potential of α-glucosidase inhibitors in type 2 diabetes mellitus: an evidence-based review . Expert Opin Pharmacother, 2015,16(13):1959-1981.

[885] Joshipura K J, Muñoz-Torres F J, Morou-Bermudez E, et al. Over-the-counter mouthwash use and risk of pre-diabetes/diabetes . Nitric Oxide, 2017,71:14-20.

[886] Jovanovich A, Isakova T, Stubbs J. Microbiome and Cardiovascular Disease in CKD . Clin J Am Soc Nephrol, 2018,13(10):1598-1604.

[887] Ju Z, Li J, Lu Q, et al. Identification and quantitative investigation of the effects of intestinal microflora on the metabolism and pharmacokinetics of notoginsenoside Fc assayed by liquid chromatography with electrospray ionization tandem mass spectrometry . J Sep Sci, 2019,42(9):1740-1749.

[888] Julián-Jiménez A, Timón Z J, Laserna M E, et al. [Diagnostic and prognostic power of biomarkers to improve the management of community acquired pneumonia in the emergency department] . Enferm Infecc Microbiol Clin, 2014,32(4):225-235.

[889] Juul F, Vaidean G, Parekh N. Ultra-processed Foods and Cardiovascular Diseases: Potential Mechanisms of Action . Adv Nutr, 2021,12(5):1673-1680.

[890] Kaartinen K, Safa A, Kotha S, et al. Complement dysregulation in glomerulonephritis. Semin Immunol, 2019,45:101331.

[891] Kaiser J E, Bakian A V, Silver R M, et al. Clinical Variables Associated With Adverse Maternal Outcomes in Puerperal Group A Streptococci Infection . Obstet Gynecol, 2018,132(1):179-184.

[892] Kalantar-Zadeh K, Ward S A, Kalantar-Zadeh K, et al. Considering the Effects of Microbiome and Diet on SARS-CoV-2 Infection: Nanotechnology Roles. ACS Nano, 2020,14(5):5179-5182.

[893] Kamińska M, Aliko A, Hellvard A, et al. Effects of statins on multispecies oral biofilm identify simvastatin as a drug candidate targeting Porphyromonas gingivalis . J Periodontol, 2019,90(6):637-646.

[894] Kamleh R, Olabi A, Toufeili I, et al. The effect of partial substitution of NaCl with KCl on the physicochemical, microbiological and sensory properties of Akkawi cheese. J Sci Food Agric, 2015,95(9):1940-1948.

[895] Kanbay M, Yilmaz S, Dincer N, et al. Antidiuretic Hormone and Serum Osmolarity Physiology and Related Outcomes: What Is Old, What Is New, and What Is Unknown? . J Clin Endocrinol Metab, 2019,104(11):5406-5420.

[896] Kang M J, Ko G S, Oh D G, et al. Role of metabolism by intestinal microbiota in pharmacokinetics of oral baicalin . Arch Pharm Res, 2014,37(3):371-378.

[897] Kang Y, Cai Y. Gut microbiota and hypertension: From pathogenesis to new therapeutic strategies. Clin Res Hepatol Gastroenterol, 2018,42(2):110-117.

[898] Kano M, Takayanagi T, Harada K, et al. Bioavailability of isoflavones after ingestion of soy beverages in healthy adults. J Nutr, 2006,136(9):2291-2296.

[899] Kaplan E L, Anthony B F, Chapman S S, et al. Epidemic acute glomerulonephritis associated with type 49 streptococcal pyoderma. I. Clinical and laboratory findings . Am J Med, 1970,48(1):9-27.

[900] Karalok Z S, Taskin B D, Ozturk Z, et al. Childhood peripheral facial palsy. Childs Nerv Syst, 2018,34(5):911-917.

[901] Karalus N C, Cursons R T, Leng R A, et al. Community acquired pneumonia: aetiology and prognostic index evaluation . Thorax, 1991,46(6):413-418.

[902] Karbach S H, Schönfelder T, Brandão I, et al. Gut Microbiota Promote Angiotensin II-Induced Arterial Hypertension and Vascular Dysfunction . J Am Heart Assoc, 2016,5(9).

[903] Karl J P, Fu X, Dolnikowski G G, et al. Quantification of phylloquinone and menaquinones in feces, serum, and food by high-performance liquid chromatography-mass spectrometry . J Chromatogr B Analyt Technol Biomed Life Sci, 2014,963:128-133.

[904] Kasahara K, Rey F E. The emerging role of gut microbial metabolism on cardiovascular disease. Curr Opin Microbiol, 2019,50:64-70.

[905] Kashtanova D A, Tkacheva O N, Doudinskaya E N, et al. Gut Microbiota in Patients with Different Metabolic Statuses: Moscow Study . Microorganisms, 2018,6(4).

[906] Kassaian N, Feizi A, Aminorroaya A, et al. Probiotic and synbiotic supplementation could improve metabolic syndrome in prediabetic adults: A randomized controlled trial . Diabetes Metab Syndr, 2019,13(5):2991-2996.

[907] Kassan A, Ait-Aissa K, Kassan M. Hypothalamic miR-204 Induces Alteration of Heart Electrophysiology and Neurogenic Hypertension by Regulating the Sympathetic Nerve Activity: Potential Role of Microbiota . Cureus, 2021,13(10):e18783.

[908] Kastenbauer S, Koedel U, Becker B F, et al. Pneumococcal meningitis in the rat: evaluation of peroxynitrite scavengers for adjunctive therapy . Eur J Pharmacol, 2002,449(1-2):177-181.

[909] Kastenbauer S, Koedel U, Brzoska T, et al. Failure of alpha-melanocyte stimulating hormone to attenuate cerebral complications in experimental pneumococcal meningitis . J Neuroimmunol, 2001,116(1):56-61.

[910] Kathrani A, Fascetti A J, Larsen J A, et al. Whole-Blood Taurine Concentrations in Cats With Intestinal Disease. J Vet Intern Med, 2017,31(4):1067-1073.

[911] Katsi V, Didagelos M, Skevofilax S, et al. GUT Microbiome-GUT Dysbiosis-Arterial Hypertension: New Horizons. Curr Hypertens Rev, 2019,15(1):40-46.

[912] Katsimichas T, Antonopoulos A S, Katsimichas A, et al. The intestinal microbiota and cardiovascular disease. Cardiovasc Res, 2019,115(10):1471-1486.

[913] Katsukura Y, Tsuchiya T, Abe N, et al. Inhibition of aging changes by lyophilized Streptococcus faecalis in diet . Exp Mol Pathol, 1983,38(3):297-309.

[914] Katz J, Gao H. The Alzheimer-E. coli Axis: What Can We Learn from an Electronic Health Record Platform . J Alzheimers Dis, 2021,84(2):717-721.

[915] Kaufer H, Spengler D M, Noyes F R, et al. Orthopaedic implications of the drug subculture . J Trauma, 1974,14(10):853-867.

[916] Kaussen T, Srinivasan P K, Afify M, et al. Influence of two different levels of intra-abdominal hypertension on bacterial translocation in a porcine model . Ann Intensive Care, 2012,2 Suppl 1(Suppl 1):S17.

[917] Kawabata S, Takagaki M, Nakamura H, et al. Dysbiosis of Gut Microbiome Is Associated With Rupture of Cerebral Aneurysms . Stroke, 2022,53(3):895-903.

[918] Kawasaki S, Aoki N, Kikuchi H, et al. Clinical and microbiological evaluation of hemodialysis-associated pneumonia (HDAP): should HDAP be included in healthcare-associated pneumonia? . J Infect Chemother, 2011,17(5):640-645.

[919] Kawata A, Sakihama N, Osato Y, et al. [Toxic shock-like syndrome presenting as phlegmon of the neck: a case report] . Nihon Jibiinkoka Gakkai Kaiho, 2005,108(8):810-813.

[920] Kaye D M, Shihata W A, Jama H A, et al. Deficiency of Prebiotic Fiber and Insufficient Signaling Through Gut Metabolite-Sensing Receptors Leads to Cardiovascular Disease . Circulation, 2020,141(17):1393-1403.

[921] Kayser B D, Prifti E, Lhomme M, et al. Elevated serum ceramides are linked with obesity-associated gut dysbiosis and impaired glucose metabolism . Metabolomics, 2019,15(11):140.

[922] Kaysser L. Built to bind: biosynthetic strategies for the formation of small-molecule protease inhibitors . Nat Prod Rep, 2019,36(12):1654-1686.

[923] Keller C, Wei P, Wancewicz B, et al. Extraction optimization for combined metabolomics, peptidomics, and proteomics analysis of gut microbiota samples . J Mass Spectrom, 2021,56(4):e4625.

[924] Kellow N J, Coughlan M T, Savige G S, et al. Effect of dietary prebiotic supplementation on advanced glycation, insulin resistance and inflammatory biomarkers in adults with pre-diabetes: a study protocol for a double-blind placebo-controlled randomised crossover clinical trial . BMC Endocr Disord, 2014,14:55.

[925] Kelly P, McPartlin J, Scott J. A combined high-performance liquid chromatographic-microbiological assay for serum folic acid . Anal Biochem, 1996,238(2):179-183.

[926] Kelly T N, Bazzano L A, Ajami N J, et al. Gut Microbiome Associates With Lifetime Cardiovascular Disease Risk Profile Among Bogalusa Heart Study Participants . Circ Res, 2016,119(8):956-964.

[927] Kemble J V. PH changes on the surface of burns . Br J Plast Surg, 1975,28(3):181-184.

[928] Keshavarzi S, MacDougall M, Lulic D, et al. Clinical experience with the surgicel family of absorbable hemostats (oxidized regenerated cellulose) in neurosurgical applications: a review . Wounds, 2013,25(6):160-167.

[929] Kessous R, Weintraub A Y, Sergienko R, et al. Bacteruria with group-B streptococcus: is it a risk factor for adverse pregnancy outcomes?. J Matern Fetal Neonatal Med, 2012,25(10):1983-1986.

[930] Keziah S M, Devi C S. Fibrinolytic and ACE Inhibitory Activity of Nattokinase Extracted from Bacillus subtilis VITMS 2: A Strain Isolated from Fermented Milk of Vigna unguiculata . Protein J, 2021,40(6):876-890.

[931] Khaddour K, Sikora A, Tahir N, et al. Case Report: The Importance of Novel Coronavirus Disease (COVID-19) and Coinfection with Other Respiratory Pathogens in the Current Pandemic . Am J Trop Med Hyg, 2020,102(6):1208-1209.

[932] Khaja M, Adler D, Lominadze G. Expressive aphasia caused by Streptococcus intermedius brain abscess in an immunocompetent patient . Int Med Case Rep J, 2017,10:25-30.

[933] Khalesi S, Sun J, Buys N, et al. Effect of probiotics on blood pressure: a systematic review and meta-analysis of randomized, controlled trials . Hypertension, 2014,64(4):897-903.

[934] Khan F Y. Streptococcus agalactiae Meningitis in Adult Patient: A Case Report and Literature Review . Case Rep Infect Dis, 2016,2016:6183602.

[935] Khan I, Khan I, Jianye Z, et al. Exploring blood microbial communities and their influence on human cardiovascular disease . J Clin Lab Anal, 2022,36(4):e24354.

[936] Khan M Z, Tahir D, Kichloo A, et al. Pyogenic Liver Abscess and Sepsis Caused by Streptococcus constellatus in the Immunocompetent Host . Cureus, 2020,12(8):e9802.

[937] Khan R N, Maner-Smith K, A O J, et al. At the heart of microbial conversations: endocannabinoids and the microbiome in cardiometabolic risk . Gut Microbes, 2021,13(1):1-21.

[938] Khan S R, van der Burgh A C, Peeters R P, et al. Determinants of Serum Immunoglobulin Levels: A Systematic Review and Meta-Analysis . Front Immunol, 2021,12:664526.

[939] Khani S, Hosseini H M, Taheri M, et al. Probiotics as an alternative strategy for prevention and treatment of human diseases: a review . Inflamm Allergy Drug Targets, 2012,11(2):79-89.

[940] Kholod D, Shkurupii D. Gastrointestinal insufficiency syndrome in intensive care of newborn: literature review. Wiad Lek, 2019,72(11 cz 1):2182-2186.

[941] Khurana M, Lal J, Kamboj V P, et al. Pharmacokinetic interaction of tetracycline with centchroman in healthy female volunteers . Drugs R D, 2003,4(5):293-299.

[942] Khurana S, Mewara A, Verma S, et al. Central nervous system infection with Acanthamoeba in a malnourished child . BMJ Case Rep, 2012,2012.

[943] Kim E D, Lee H S, Kim K T, et al. Antioxidant and Angiotensin-Converting Enzyme (ACE) Inhibitory Activities of Yogurt Supplemented with Lactiplantibacillus plantarum NK181 and Lactobacillus delbrueckii KU200171 and Sensory Evaluation . Foods, 2021,10(10).

[944] Kim H S. Do an Altered Gut Microbiota and an Associated Leaky Gut Affect COVID-19 Severity?. mBio, 2021,12(1).

[945] Kim I S, Hwang C W, Yang W S, et al. Current Perspectives on the Physiological Activities of Fermented Soybean-Derived Cheonggukjang . Int J Mol Sci, 2021,22(11).

[946] Kim M S, Hwang S S, Park E J, et al. Strict vegetarian diet improves the risk factors associated with metabolic diseases by modulating gut microbiota and reducing intestinal inflammation . Environ Microbiol Rep, 2013,5(5):765-775.

[947] Kim M, Huda M N, Bennett B J. Sequence meets function-microbiota and cardiovascular disease. Cardiovasc Res, 2022,118(2):399-412.

[948] Kim S Y. Production of Fermented Kale Juices with Lactobacillus Strains and Nutritional Composition . Prev Nutr Food Sci, 2017,22(3):231-236.

[949] Kim S, Goel R, Kumar A, et al. Imbalance of gut microbiome and intestinal epithelial barrier dysfunction in patients with high blood pressure . Clin Sci (Lond), 2018,132(6):701-718.

[950] Kim S, Rigatto K, Gazzana M B, et al. Altered Gut Microbiome Profile in Patients With Pulmonary Arterial Hypertension. Hypertension, 2020,75(4):1063-1071.

[951] Kim Y, Keogh J B, Clifton P M. Benefits of Nut Consumption on Insulin Resistance and Cardiovascular Risk Factors: Multiple Potential Mechanisms of Actions. Nutrients, 2017,9(11).

[952] Kim-Sing A, Kays M B, James V E, et al. Intravenous streptomycin use in a patient infected with high-level, gentamicin-resistant Streptococcus faecalis . Ann Pharmacother, 1993,27(6):712-714.

[953] Kinlay S, Michel T, Leopold J A. The Future of Vascular Biology and Medicine. Circulation, 2016,133(25):2603-2609.

[954] Kirkpatrick A W, Hamilton D R, McKee J L, et al. Do we have the guts to go? The abdominal compartment, intra-abdominal hypertension, the human microbiome and exploration class space missions . Can J Surg, 2020,63(6):E581-E593.

[955] Klapdor B, Ewig S, Pletz M W, et al. Community-acquired pneumonia in younger patients is an entity on its own . Eur Respir J, 2012,39(5):1156-1161.

[956] Ko C Y, Hu A K, Chou D, et al. Analysis of oral microbiota in patients with obstructive sleep apnea-associated hypertension . Hypertens Res, 2019,42(11):1692-1700.

[957] Ko C Y, Hu A K, Zhang L, et al. Alterations of oral microbiota in patients with obstructive sleep apnea-hypopnea syndrome treated with continuous positive airway pressure: a pilot study . Sleep Breath, 2021.

[958] Ko C Y, Su H Z, Zhang L, et al. Disturbances of the Gut Microbiota, Sleep Architecture, and mTOR Signaling Pathway in Patients with Severe Obstructive Sleep Apnea-Associated Hypertension . Int J Hypertens, 2021,2021:9877053.

[959] Kocarnik J M, Compton K, Dean F E, et al. Cancer Incidence, Mortality, Years of Life Lost, Years Lived With Disability, and Disability-Adjusted Life Years for 29 Cancer Groups From 2010 to 2019: A Systematic Analysis for the Global Burden of Disease Study 2019 . JAMA Oncol, 2022,8(3):420-444.

[960] Koch C D, Gladwin M T, Freeman B A, et al. Enterosalivary nitrate metabolism and the microbiome: Intersection of microbial metabolism, nitric oxide and diet in cardiac and pulmonary vascular health . Free Radic Biol Med, 2017,105:48-67.

[961] Koedel U, Angele B, Rupprecht T, et al. Toll-like receptor 2 participates in mediation of immune response in experimental pneumococcal meningitis . J Immunol, 2003,170(1):438-444.

[962] Kok H, Jureen R, Soon C Y, et al. Colon cancer presenting as Streptococcus gallolyticus infective endocarditis . Singapore Med J, 2007,48(2):e43-e45.

[963] Kokayi A J. Septic Shock Secondary to a Pyogenic Liver Abscess Following Complicated Appendicitis. Cureus, 2021,13(9):e18359.

[964] Kolho K L, Pessia A, Jaakkola T, et al. Faecal and Serum Metabolomics in Paediatric Inflammatory Bowel Disease. J Crohns Colitis, 2017,11(3):321-334.

[965] Komorniak N, Stachowska E. [Surgical treatment of obesity from the nutritional point - opportunities and dangers] . Pol Merkur Lekarski, 2018,45(269):179-184.

[966] Konishi T, Kusakabe S, Hino A, et al. Low diversity of gut microbiota in the early phase of post-bone marrow transplantation increases the risk of chronic graft-versus-host disease . Bone Marrow Transplant, 2021,56(7):1728-1731.

[967] Konjar Š, Pavšič M, Veldhoen M. Regulation of Oxygen Homeostasis at the Intestinal Epithelial Barrier Site. Int J Mol Sci, 2021,22(17).

[968] Konopelski P, Konop M, Perlejewski K, et al. Genetically determined hypertensive phenotype affects gut microbiota composition, but not vice versa . J Hypertens, 2021,39(9):1790-1799.

[969] Kontou P, Kuti J L, Nicolau D P. Validation of the Infectious Diseases Society of America/American Thoracic Society criteria to predict severe community-acquired pneumonia caused by Streptococcus pneumoniae . Am J Emerg Med, 2009,27(8):968-974.

[970] Koppe L, Beddhu S, Chauveau P, et al. A call for a better understanding of the role of dietary amino acids and post-translational protein modifications of the microbiome in the progression of CKD . Nephrol Dial Transplant, 2021,36(8):1357-1360.

[971] Korhonen H, Pihlanto A. Technological options for the production of health-promoting proteins and peptides derived from milk and colostrum . Curr Pharm Des, 2007,13(8):829-843.

[972] Kori M, Itoh K, Inada Y, et al. Synthesis and angiotensin converting enzyme-inhibitory activity of N-[(1S)-1-carboxy-5-(4-piperidyl)pentyl]-L-alanine derivatives . Chem Pharm Bull (Tokyo), 1994,42(3):580-585.

[973] Korzets A, Ori Y, Zevin D, et al. Group A streptococcal bacteraemia and necrotizing faciitis in a renal transplant patient: a case for intravenous immunoglobulin therapy . Nephrol Dial Transplant, 2002,17(1):150-152.

[974] Kotsis V, Nilsson P, Grassi G, et al. New developments in the pathogenesis of obesity-induced hypertension. J Hypertens, 2015,33(8):1499-1508.

[975] Kouris-Blazos A, Belski R. Health benefits of legumes and pulses with a focus on Australian sweet lupins. Asia Pac J Clin Nutr, 2016,25(1):1-17.

[976] Koyama M, Naramoto K, Nakajima T, et al. Purification and identification of antihypertensive peptides from fermented buckwheat sprouts . J Agric Food Chem, 2013,61(12):3013-3021.

[977] Koziner V B. [The polysaccharide dextran, its biological action and practical application. 1. General information on dextran, its discovery and methods of obtaining it] . Usp Sovrem Biol, 1966,62(2):197-214.

[978] Kozłowska D, Myśliwiec H, Kiluk P, et al. Clinical and epidemiological assessment of patients hospitalized for primary and recurrent erysipelas . Przegl Epidemiol, 2016,70(4):575-584.

[979] Kramer A H, Bleck T P. Neurocritical care of patients with central nervous system infections. Curr Treat Options Neurol, 2008,10(3):201-211.

[980] Krause P J, Owens N J, Nightingale C H, et al. Penetration of amoxicillin, cefaclor, erythromycin-sulfisoxazole, and trimethoprim-sulfamethoxazole into the middle ear fluid of patients with chronic serous otitis media . J Infect Dis, 1982,145(6):815-821.

[981] Krishnamoorthy R, Adisa A R, Periasamy V S, et al. Colonic Bacteria-Transformed Catechin Metabolite Response to Cytokine Production by Human Peripheral Blood Mononuclear Cells . Biomolecules, 2019,9(12).

[982] Kubo M. Diurnal Rhythmicity Programs of Microbiota and Transcriptional Oscillation of Circadian Regulator, NFIL3. Front Immunol, 2020,11:552188.

[983] Kuka J, Liepinsh E, Makrecka-Kuka M, et al. Suppression of intestinal microbiota-dependent production of pro-atherogenic trimethylamine N-oxide by shifting L-carnitine microbial degradation . Life Sci, 2014,117(2):84-92.

[984] Kumar D, Mukherjee S S, Chakraborty R, et al. The emerging role of gut microbiota in cardiovascular diseases. Indian Heart J, 2021,73(3):264-272.

[985] Kumar H, Schütz F, Bhardwaj K, et al. Recent advances in the concept of paraprobiotics: Nutraceutical/functional properties for promoting children health . Crit Rev Food Sci Nutr, 2021:1-16.

[986] Kurokawa Y, Hashi K, Fujishige M, et al. [Spinal subdural empyema diagnosed by MRI and recovered by conservative treatment] . No To Shinkei, 1989,41(5):513-517.

[987] Kurosawa M T, Nakamura Y, Yamamoto N, et al. Effects of Val-Pro-Pro and Ile-Pro-Pro on nondipper patients: a preliminary study . J Med Food, 2011,14(5):538-542.

[988] Kusuhara S, Ito M, Sato T, et al. Intracellular GSH of Streptococcus thermophilus shows anti-oxidative activity against low-density lipoprotein oxidation in vitro and in a hyperlipidaemic hamster model . Benef Microbes, 2018,9(1):143-152.

[989] Kuwal A, Joshi V, Dutt N, et al. A Prospective Study of Bacteriological Etiology in Hospitalized Acute Exacerbation of COPD Patients: Relationship with Lung Function and Respiratory Failure . Turk Thorac J, 2018,19(1):19-27.

[990] Kwon Y N, Kim Y J. Gut-Brain-Microbiota Axis and Hypertension: A Literature Review. Curr Pharm Des, 2021,27(37):3939-3946.

[991] Ladeiras-Lopes R, Bucciarelli-Ducci C. Dysfunctional Postprandial Flow Changes, Adverse Cardiac Remodeling, and Hypertension: Follow Your Heart but Trust Your Gut? . Circ Cardiovasc Imaging, 2019,12(11):e9981.

[992] Ladjimi H, Gounelle J C, Auchere D. Effect of diet on folates levels and distribution in selected tissues of the rat . Arch Int Physiol Biochim Biophys, 1992,100(1):67-72.

[993] Laiakis E C, Morris G A, Fornace A J, et al. Metabolomic analysis in severe childhood pneumonia in the Gambia, West Africa: findings from a pilot study . PLoS One, 2010,5(9).

[994] Lakshmanan A P, Shatat I F, Zaidan S, et al. Bifidobacterium reduction is associated with high blood pressure in children with type 1 diabetes mellitus . Biomed Pharmacother, 2021,140:111736.

[995] Lal C V, Ambalavanan N. Cellular and humoral biomarkers of Bronchopulmonary Dysplasia. Early Hum Dev, 2017,105:35-39.

[996] Lam D W, LeRoith D. Metabolic Syndrome . 2000.

[997] Lamaa N, Bromberg R, Foroughi M, et al. Severe aortic regurgitation masked as sepsis-induced ARDS in a patient with Streptococcus agalactiae endocarditis . BMJ Case Rep, 2018,11(1).

[998] Lambert M, Thorup A C, Hansen E, et al. Combined Red Clover isoflavones and probiotics potently reduce menopausal vasomotor symptoms . PLoS One, 2017,12(6):e176590.

[999] LaMonte M J, Gordon J H, Diaz-Moreno P, et al. Oral Microbiome Is Associated With Incident Hypertension Among Postmenopausal Women . J Am Heart Assoc, 2022,11(6):e21930.

[1000] Lang R, Folman Y, Ravid M, et al. Penetration of ceftriaxone into the intervertebral disc. J Bone Joint Surg Am, 1994,76(5):689-691.

[1001] Larsen L, Nielsen T H, Nordström C H, et al. Patterns of cerebral tissue oxygen tension and cytoplasmic redox state in bacterial meningitis . Acta Anaesthesiol Scand, 2019,63(3):329-336.

[1002] Lässiger-Herfurth A, Pontarollo G, Grill A, et al. The Gut Microbiota in Cardiovascular Disease and Arterial Thrombosis. Microorganisms, 2019,7(12).

[1003] Laterre P F, Garber G, Levy H, et al. Severe community-acquired pneumonia as a cause of severe sepsis: data from the PROWESS study . Crit Care Med, 2005,33(5):952-961.

[1004] Latimer M E, L'Etoile N, Seidlitz J, et al. Therapeutic plasma apheresis as a treatment for 35 severely ill children and adolescents with pediatric autoimmune neuropsychiatric disorders associated with streptococcal infections . J Child Adolesc Psychopharmacol, 2015,25(1):70-75.

[1005] Lau E, Neves J S, Ferreira-Magalhães M, et al. Probiotic Ingestion, Obesity, and Metabolic-Related Disorders: Results from NHANES, 1999-2014 . Nutrients, 2019,11(7).

[1006] Lau K, Srivatsav V, Rizwan A, et al. Bridging the Gap between Gut Microbial Dysbiosis and Cardiovascular Diseases . Nutrients, 2017,9(8).

[1007] Laursen A, Sluijs I, Boer J, et al. Substitutions between dairy products and risk of stroke: results from the European Investigation into Cancer and Nutrition-Netherlands (EPIC-NL) cohort . Br J Nutr, 2019,121(12):1398-1404.

[1008] Lavefve L, Marasini D, Carbonero F. Microbial Ecology of Fermented Vegetables and Non-Alcoholic Drinks and Current Knowledge on Their Impact on Human Health . Adv Food Nutr Res, 2019,87:147-185.

[1009] LeBel M, Pellerin M, Bergeron M G. Serum bactericidal activity of ceftazidime increased by netilmicin. Drug Intell Clin Pharm, 1985,19(12):932-936.

[1010] Leber B, Mayrhauser U, Rybczynski M, et al. Innate immune dysfunction in acute and chronic liver disease . Wien Klin Wochenschr, 2009,121(23-24):732-744.

[1011] Ledingham I M, McArdle C S. Prospective study of the treatment of septic shock . Lancet, 1978,1(8075):1194-1197.

[1012] Lee C S, Chen M J, Chiou Y H, et al. Invasive pneumococcal pneumonia is the major cause of paediatric haemolytic-uraemic syndrome in Taiwan . Nephrology (Carlton), 2012,17(1):48-52.

[1013] Lee E W, Lee A E, Saab S, et al. Retrograde Transvenous Obliteration (RTO): A New Treatment Option for Hepatic Encephalopathy . Dig Dis Sci, 2020,65(9):2483-2491.

[1014] Lee H S, Kim M R, Park Y, et al. Fermenting red ginseng enhances its safety and efficacy as a novel skin care anti-aging ingredient: in vitro and animal study . J Med Food, 2012,15(11):1015-1023.

[1015] Lee Y M, Skurk T, Hennig M, et al. Effect of a milk drink supplemented with whey peptides on blood pressure in patients with mild hypertension . Eur J Nutr, 2007,46(1):21-27.

[1016] Leelahavanichkul A, Panpetch W, Worasilchai N, et al. Evaluation of gastrointestinal leakage using serum (1→3)-β-D-glucan in a Clostridium difficile murine model . FEMS Microbiol Lett, 2016,363(18).

[1017] Leeming R J, Pollock A, Melville L J, et al. Measurement of 5-methyltetrahydrofolic acid in man by high-performance liquid chromatography . Metabolism, 1990,39(9):902-904.

[1018] Leneuve-Dorilas M, Favre A, Louis A, et al. Risk Factors for Very Preterm Births in French Guiana: The Burden of Induced Preterm Birth . AJP Rep, 2019,9(1):e44-e53.

[1019] Leng Y, Jiang C, Xing X, et al. Prevention of Severe Intestinal Barrier Dysfunction Through a Single-Species Probiotics is Associated With the Activation of Microbiome-Mediated Glutamate-Glutamine Biosynthesis . Shock, 2021,55(1):128-137.

[1020] Leong K, Jayasinghe T N, Derraik J, et al. Protocol for the Gut Bugs Trial: a randomised double-blind placebo-controlled trial of gut microbiome transfer for the treatment of obesity in adolescents . BMJ Open, 2019,9(4):e26174.

[1021] Lequeu B, Guilland J C, Klepping J. Measurement of plasma pyridoxal 5'-phosphate by combination of an enzymatic assay with high-performance liquid chromatography/electrochemistry . Anal Biochem, 1985,149(2):296-300.

[1022] Leszczyńska A, Skrzypczyk P, Leszczyńska B, et al. [Zwiększenie częstości zachorowania na ostre popaciorkowcowe kłębuszkowe zapalenie nerek w pierwszej połowie roku 2018 - doświadczenie jednego ośrodka] . Pol Merkur Lekarski, 2019,46(273):115-121.

[1023] Leszek J, Mikhaylenko E V, Belousov D M, et al. The Links between Cardiovascular Diseases and Alzheimer's Disease . Curr Neuropharmacol, 2021,19(2):152-169.

[1024] Leustean A M, Ciocoiu M, Sava A, et al. Implications of the Intestinal Microbiota in Diagnosing the Progression of Diabetes and the Presence of Cardiovascular Complications . J Diabetes Res, 2018,2018:5205126.

[1025] Levy H. Comparison of Ballard catheter bronchoalveolar lavage with bronchoscopic bronchoalveolar lavage . Chest, 1994,106(6):1753-1756.

[1026] Lewandowski K, Kaniewska M, Karłowicz K, et al. The effectiveness of microencapsulated sodium butyrate at reducing symptoms in patients with irritable bowel syndrome . Prz Gastroenterol, 2022,17(1):28-34.

[1027] Lewis C V, Taylor W R. Intestinal barrier dysfunction as a therapeutic target for cardiovascular disease . Am J Physiol Heart Circ Physiol, 2020,319(6):H1227-H1233.

[1028] Lewis-Mikhael A M, Davoodvandi A, Jafarnejad S. Effect of Lactobacillusplantarum containing probiotics on blood pressure: A systematic review and meta-analysis . Pharmacol Res, 2020,153:104663.

[1029] Leyrolle Q, Cserjesi R, Mulders M, et al. Specific gut microbial, biological, and psychiatric profiling related to binge eating disorders: A cross-sectional study in obese patients . Clin Nutr, 2021,40(4):2035-2044.

[1030] Lezutekong J N, Nikhanj A, Oudit G Y. Imbalance of gut microbiome and intestinal epithelial barrier dysfunction in cardiovascular disease . Clin Sci (Lond), 2018,132(8):901-904.

[1031] Li C, Xiao P, Lin D, et al. Risk Factors for Intestinal Barrier Impairment in Patients With Essential Hypertension . Front Med (Lausanne), 2020,7:543698.

[1032] Li D, Achkar J P, Haritunians T, et al. A Pleiotropic Missense Variant in SLC39A8 Is Associated With Crohn's Disease and Human Gut Microbiome Composition . Gastroenterology, 2016,151(4):724-732.

[1033] Li D, Lu Y, Yuan S, et al. Gut microbiota-derived metabolite Trimethylamine-N-oxide (TMAO) and multiple health outcomes: an umbrella review and updated meta-analysis . Am J Clin Nutr, 2022.

[1034] Li H B, Xu M L, Du MM, et al. Curcumin ameliorates hypertension via gut-brain communication in spontaneously hypertensive rat . Toxicol Appl Pharmacol, 2021,429:115701.

[1035] Li H B, Yang T, Richards E M, et al. Maternal Treatment With Captopril Persistently Alters Gut-Brain Communication and Attenuates Hypertension of Male Offspring . Hypertension, 2020,75(5):1315-1324.

[1036] Li H, Liu B, Song J, et al. Characteristics of Gut Microbiota in Patients with Hypertension and/or Hyperlipidemia: A Cross-Sectional Study on Rural Residents in Xinxiang County, Henan Province . Microorganisms, 2019,7(10).

[1037] Li H, Xu H, Li Y, et al. Alterations of gut microbiota contribute to the progression of unruptured intracranial aneurysms . Nat Commun, 2020,11(1):3218.

[1038] Li J X, Gray B M, Oliver J R, et al. Delayed thromboxane synthesis inhibition, but not cholinergic blockade, reverses group B streptococcus-induced pulmonary hypertension . Dev Pharmacol Ther, 1992,19(1):40-49.

[1039] Li J X, Gray B M, Pritchard D G, et al. Capsular type-specific polysaccharide partially inhibits group B Streptococcus-induced pulmonary hypertension . Am Rev Respir Dis, 1993,148(1):152-157.

[1040] Li J, Raizada M K, Richards E M. Gut-brain-bone marrow axis in hypertension . Curr Opin Nephrol Hypertens, 2021,30(2):159-165.

[1041] Li J, Sun F, Guo Y, et al. High-Salt Diet Gets Involved in Gastrointestinal Diseases through the Reshaping of Gastroenterological Milieu . Digestion, 2019,99(4):267-274.

[1042] Li J, Wang D, Sun J. Application of fecal microbial transplantation in hepatic encephalopathy after transjugular intrahepatic portosystemic shunt . Medicine (Baltimore), 2022,101(3):e28584.

[1043] Li J, Xu Y, Cai Y, et al. Association of Differential Metabolites With Small Intestinal Microflora and Maternal Outcomes in Subclinical Hypothyroidism During Pregnancy . Front Cell Infect Microbiol, 2021,11:779659.

[1044] Li J, Yang X, Zhou X, et al. The Role and Mechanism of Intestinal Flora in Blood Pressure Regulation and Hypertension Development . Antioxid Redox Signal, 2021,34(10):811-830.

[1045] Li J, Zhao F, Wang Y, et al. Gut microbiota dysbiosis contributes to the development of hypertension . Microbiome, 2017,5(1):14.

[1046] Li J, Zuo K, Zhang J, et al. Shifts in gut microbiome and metabolome are associated with risk of recurrent atrial fibrillation . J Cell Mol Med, 2020,24(22):13356-13369.

[1047] Li M, Li K, Tang S, et al. Restoration of the gut microbiota is associated with a decreased risk of hepatic encephalopathy after TIPS . JHEP Rep, 2022,4(5):100448.

[1048] Li P, Cai X, Xiao N, et al. Sacha inchi (Plukenetia volubilis L.) shell extract alleviates hypertension in association with the regulation of gut microbiota . Food Funct, 2020,11(9):8051-8067.

[1049] Li Q, Gao B, Siqin B, et al. Gut Microbiota: A Novel Regulator of Cardiovascular Disease and Key Factor in the Therapeutic Effects of Flavonoids . Front Pharmacol, 2021,12:651926.

[1050] Li S, Bu T, Zheng J, et al. Preparation, Bioavailability, and Mechanism of Emerging Activities of Ile-Pro-Pro and Val-Pro-Pro . Compr Rev Food Sci Food Saf, 2019,18(4):1097-1110.

[1051] Li Y, Hsieh C H, Lai C W, et al. Tyramine detection using PEDOT:PSS/AuNPs/1-methyl-4-mercaptopyridine modified screen-printed carbon electrode with molecularly imprinted polymer solid phase extraction . Biosens Bioelectron, 2017,87:142-149.

[1052] Li Y, Salih I R, Chi H L, et al. Altered Gut Microbiota is Involved in the Anti-Hypertensive Effects of Vitamin C in Spontaneously Hypertensive Rat . Mol Nutr Food Res, 2021,65(7):e2000885.

[1053] Li Y, Zhao D, Qian M, et al. Amlodipine, an anti-hypertensive drug, alleviates non-alcoholic fatty liver disease by modulating gut microbiota . Br J Pharmacol, 2022,179(9):2054-2077.

[1054] Li Y, Zhou G, Peng Y, et al. Screening and identification of three typical phenylethanoid glycosides metabolites from Cistanches Herba by human intestinal bacteria using UPLC/Q-TOF-MS . J Pharm Biomed Anal, 2016,118:167-176.

[1055] Liang T, Wu L, Xi Y, et al. Probiotics supplementation improves hyperglycemia, hypercholesterolemia, and hypertension in type 2 diabetes mellitus: An update of meta-analysis . Crit Rev Food Sci Nutr, 2021,61(10):1670-1688.

[1056] Liang X, Li Y, Xiong K, et al. Demodex Infection Changes Ocular Surface Microbial Communities, in Which Meibomian Gland Dysfunction May Play a Role . Ophthalmol Ther, 2021,10(3):601-617.

[1057] Liang Y, Jin X, Huang Y, et al. Development and application of a real-time polymerase chain reaction assay for detection of a novel gut bacteriophage (crAssphage) . J Med Virol, 2018,90(3):464-468.

[1058] Lieske J C. Is the intestinal microbiome a modifiable cofactor in the development of hyperoxaluria after Roux-en-Y gastric bypass? . Surg Obes Relat Dis, 2017,13(7):1157-1158.

[1059] Lieske J C. Probiotics for prevention of urinary stones . Ann Transl Med, 2017,5(2):29.

[1060] Lieske J C, Goldfarb D S, De Simone C, et al. Use of a probiotic to decrease enteric hyperoxaluria . Kidney Int, 2005,68(3):1244-1249.

[1061] Lieske J C, Tremaine W J, De Simone C, et al. Diet, but not oral probiotics, effectively reduces urinary oxalate excretion and calcium oxalate supersaturation . Kidney Int, 2010,78(11):1178-1185.

[1062] Light R B. Indomethacin and acetylsalicylic acid reduce intrapulmonary shunt in experimental pneumococcal pneumonia . Am Rev Respir Dis, 1986,134(3):520-525.

[1063] Limm-Chan B, Musgrave J, Lau R, et al. Incidence of Acute Post-Streptococcal Glomerulonephritis in Hawai'i and Factors Affecting Length of Hospitalization . Hawaii J Health Soc Welf, 2020,79(5):149-152.

[1064] Lin B Y, Lin W D, Huang C K, et al. Changes of gut microbiota between different weight reduction programs . Surg Obes Relat Dis, 2019,15(5):749-758.

[1065] Lin C L. Stroke and diets - A review . Tzu Chi Med J, 2021,33(3):238-242.

[1066] Lin F Y, Troendle J F. Hypothesis: Neonatal respiratory distress may be related to asymptomatic colonization with group B streptococci . Pediatr Infect Dis J, 2006,25(10):884-888.

[1067] Lin J N, Chang L L, Lai C H, et al. Clinical and molecular characteristics of invasive and noninvasive skin and soft tissue infections caused by group A Streptococcus . J Clin Microbiol, 2011,49(10):3632-3637.

[1068] Lin P P, Hsieh Y M, Kuo W W, et al. Inhibition of cardiac hypertrophy by probiotic-fermented purple sweet potato yogurt in spontaneously hypertensive rat hearts . Int J Mol Med, 2012,30(6):1365-1375.

[1069] Lin Y C, Wang H Y, Kuo Y C, et al. Gut-flora metabolites is not associated with synchronous carotid artery plaque and non-alcoholic fatty liver disease in asymptomatic adults: A STROBE-compliant article . Medicine (Baltimore), 2021,100(34):e27048.

[1070] Lin Y, Wu S H, Wang X H, et al. Associations of imbalance of intestinal flora with severity of disease, inflammatory factors, adiponectin, and vascular endothelial function of hypertension patients . Kaohsiung J Med Sci, 2022,38(2):165-173.

[1071] Linares D M, Gómez C, Renes E, et al. Lactic Acid Bacteria and Bifidobacteria with Potential to Design Natural Biofunctional Health-Promoting Dairy Foods . Front Microbiol, 2017,8:846.

[1072] Linares D M, O'Callaghan T F, O'Connor P M, et al. Streptococcus thermophilus APC151 Strain Is Suitable for the Manufacture of Naturally GABA-Enriched Bioactive Yogurt . Front Microbiol, 2016,7:1876.

[1073] Lindquist L, Bentin R, Hedström K G, et al. Experimental meningitis in the rabbit. I. Arterial blood pressure and acid-base balance during halothane anesthesia and in situ freezing of the brain . Acta Neurol Scand, 1987,75(6):400-404.

[1074] Lindquist L, Wibom R, Lundbergh P, et al. Experimental meningitis in the rabbit. II. Cerebral energy metabolism in relation to increased cerebrospinal fluid concentrations of lactate . Acta Neurol Scand, 1987,75(6):405-409.

[1075] Linz D, Gawałko M, Sanders P, et al. Does gut microbiota affect atrial rhythm? Causalities and speculations . Eur Heart J, 2021,42(35):3521-3525.

[1076] Liong M T. Probiotics: a critical review of their potential role as antihypertensives, immune modulators, hypocholesterolemics, and perimenopausal treatments . Nutr Rev, 2007,65(7):316-328.

[1077] Lippi G, Favaloro E J, Cervellin G. Hemostatic properties of the lymph: relationships with occlusion and thrombosis . Semin Thromb Hemost, 2012,38(2):213-221.

[1078] Listernick R, Klein-Gitelman M, Chadwick E, et al. A 7-year-old girl with arthritis and hypertension . Pediatr Ann, 2004,33(12):802, 804-806.

[1079] Liu B, Piao X, Niu W, et al. Kuijieyuan Decoction Improved Intestinal Barrier Injury of Ulcerative Colitis by Affecting TLR4-Dependent PI3K/AKT/NF-κB Oxidative and Inflammatory Signaling and Gut Microbiota . Front Pharmacol, 2020,11:1036.

[1080] Liu F, Ling Z, Xiao Y, et al. Alterations of Urinary Microbiota in Type 2 Diabetes Mellitus with Hypertension and/or Hyperlipidemia . Front Physiol, 2017,8:126.

[1081] Liu G, Feng W, Li D, et al. The Mga Regulon but Not Deoxyribonuclease Sda1 of Invasive M1T1 Group A Streptococcus Contributes to In Vivo Selection of CovRS Mutations and Resistance to Innate Immune Killing Mechanisms . Infect Immun, 2015,83(11):4293-4303.

[1082] Liu H M, Lin X, Meng X H, et al. Integrated metagenome and metabolome analyses of blood pressure studies in early postmenopausal Chinese women . J Hypertens, 2021,39(9):1800-1809.

[1083] Liu H, Zhuang J, Tang P, et al. The Role of the Gut Microbiota in Coronary Heart Disease . Curr Atheroscler Rep, 2020,22(12):77.

[1084] Liu J, Li T, Wu H, et al. Lactobacillus rhamnosus GG strain mitigated the development of obstructive sleep apnea-induced hypertension in a high salt diet via regulating TMAO level and CD4(+) T cell induced-type I inflammation . Biomed Pharmacother, 2019,112:108580.

[1085] Liu J, Zhao M, Zhou J, et al. Simultaneous targeted analysis of trimethylamine-N-oxide, choline, betaine, and carnitine by high performance liquid chromatography tandem mass spectrometry . J Chromatogr B Analyt Technol Biomed Life Sci, 2016,1035:42-48.

[1086] Liu L, He X, Feng Y. Coronary heart disease and intestinal microbiota . Coron Artery Dis, 2019,30(5):384-389.

[1087] Liu M, Hanks T S, Zhang J, et al. Defects in ex vivo and in vivo growth and sensitivity to osmotic stress of group A Streptococcus caused by interruption of response regulator gene vicR . Microbiology (Reading), 2006,152(Pt 4):967-978.

[1088] Liu X Y, Fan M L, Wang H Y, et al. Metabolic profile and underlying improved bio-activity of Fructus aurantii immaturus by human intestinal bacteria . Food Funct, 2017,8(6):2193-2201.

[1089] Liu X, Lu S, Shao Y, et al. Disorders of gut microbiota in children with Tetralogy of Fallot . Transl Pediatr, 2022,11(3):385-395.

[1090] Liu X, Shao Y, Sun J, et al. Egg consumption improves vascular and gut microbiota function without increasing inflammatory, metabolic, and oxidative stress markers . Food Sci Nutr, 2022,10(1):295-304.

[1091] Liu Y, Croft K D, Hodgson J M, et al. Mechanisms of the protective effects of nitrate and nitrite in cardiovascular and metabolic diseases . Nitric Oxide, 2020,96:35-43.

[1092] Liu Y, Jiang Q, Liu Z, et al. Alteration of Gut Microbiota Relates to Metabolic Disorders in Primary Aldosteronism Patients . Front Endocrinol (Lausanne), 2021,12:667951.

[1093] Liu Y, Ju Y, Cui L, et al. Association between Dietary Fiber Intake and Incidence of Depression and Anxiety in Patients with Essential Hypertension . Nutrients, 2021,13(11).

[1094] Liu Y, Zhang F M, Hu W Z. Hypertension: microbiota-targeting treatment . Chin Med J (Engl), 2020,133(11):1353-1354.

[1095] Lo R S, Austin A S, Freeman J G. Is there a role for probiotics in liver disease? . ScientificWorldJournal, 2014,2014:874768.

[1096] Loesche W J, Schork A, Terpenning M S, et al. Assessing the relationship between dental disease and coronary heart disease in elderly U.S. veterans . J Am Dent Assoc, 1998,129(3):301-311.

[1097] Lollo P, Morato P N, Moura C S, et al. Hypertension parameters are attenuated by the continuous consumption of probiotic Minas cheese . Food Res Int, 2015,76(Pt 3):611-617.

[1098] Lopes C R, de Beaufort A J, Gesink B J, et al. Inhalation of nitric oxide: effect on cerebral hemodynamics and activity, and antioxidant status in the newborn lamb . Biol Neonate, 1996,69(4):284-292.

[1099] Lopes H F, Corrêa-Giannella M L, Consolim-Colombo F M, et al. Visceral adiposity syndrome . Diabetol Metab Syndr, 2016,8:40.

[1100] Lopez J P, Roque J, Torres J, et al. Severe retinal hemorrhages in infants with aggressive, fatal Streptococcus pneumoniae meningitis . J AAPOS, 2010,14(1):97-98.

[1101] Lordan R, Vidal N P, Huong P T, et al. Yoghurt fermentation alters the composition and antiplatelet properties of milk polar lipids . Food Chem, 2020,332:127384.

[1102] Lorenzo O, Crespo-Yanguas M, Hang T, et al. Addition of Probiotics to Anti-Obesity Therapy by Percutaneous Electrical Stimulation of Dermatome T6. A Pilot Study . Int J Environ Res Public Health, 2020,17(19).

[1103] Losacco M, Gallerani R, Gobbetti M, et al. Production of active angiotensin-I converting enzyme inhibitory peptides derived from bovine beta-casein by recombinant DNA technologies . Biotechnol J, 2007,2(11):1425-1434.

[1104] Losso J N, Losso M N, Toc M, et al. The Young Age and Plant-Based Diet Hypothesis for Low SARS-CoV-2 Infection and COVID-19 Pandemic in Sub-Saharan Africa . Plant Foods Hum Nutr, 2021,76(3):270-280.

[1105] Losurdo L, Quintieri L, Caputo L, et al. Cloning and expression of synthetic genes encoding angiotensin-I converting enzyme (ACE)-inhibitory bioactive peptides in Bifidobacterium pseudocatenulatum . FEMS Microbiol Lett, 2013,340(1):24-32.

[1106] Louca P, Menni C, Padmanabhan S. Genomic Determinants of Hypertension With a Focus on Metabolomics and the Gut Microbiome . Am J Hypertens, 2020,33(6):473-481.

[1107] Louca P, Nogal A, Wells P M, et al. Gut microbiome diversity and composition is associated with hypertension in women . J Hypertens, 2021,39(9):1810-1816.

[1108] Louis-Jean S, Martirosyan D. Nutritionally Attenuating the Human Gut Microbiome To Prevent and Manage Metabolic Syndrome . J Agric Food Chem, 2019,67(46):12675-12684.

[1109] Lu D, Wang J, Zhang H, et al. Renal denervation improves chronic intermittent hypoxia induced hypertension and cardiac fibrosis and balances gut microbiota . Life Sci, 2020,262:118500.

[1110] Lu D, Yao X, Abulimiti A, et al. Profiling of lung microbiota in the patients with obstructive sleep apnea . Medicine (Baltimore), 2018,97(26):e11175.

[1111] Lu S, Shao L, Zhang Y, et al. Predictive Value of Gut Microbiome for Cognitive Impairment in Patients with Hypertension . Dis Markers, 2021,2021:1683981.

[1112] Luan S, Zhang S, Pan L, et al. Salivary microbiota analysis of patients with membranous nephropathy . Mol Med Rep, 2022,25(5).

[1113] Lucas R, Yue Q, Alli A, et al. The Lectin-like Domain of TNF Increases ENaC Open Probability through a Novel Site at the Interface between the Second Transmembrane and C-terminal Domains of the α-Subunit . J Biol Chem, 2016,291(45):23440-23451.

[1114] Lucking E F, O'Connor K M, Strain C R, et al. Chronic intermittent hypoxia disrupts cardiorespiratory homeostasis and gut microbiota composition in adult male guinea-pigs . EBioMedicine, 2018,38:191-205.

[1115] Lucock M D, Hartley R, Smithells R W. A rapid and specific HPLC-electrochemical method for the determination of endogenous 5-methyltetrahydrofolic acid in plasma using solid phase sample preparation with internal standardization . Biomed Chromatogr, 1989,3(2):58-63.

[1116] Lucock M D, Wild J, Smithells R W, et al. In vivo characterization of the absorption and biotransformation of pteroylmonoglutamic acid in man: a model for future studies . Biochem Med Metab Biol, 1989,42(1):30-42.

[1117] Ludemann J P, Poskitt K, Singhal A. Intracranial hypertension secondary to sigmoid sinus compression by group A streptococcal epidural abscess . J Laryngol Otol, 2010,124(1):93-95.

[1118] Lund H L, Schwarze P E, Thelle D S, et al. Low levels of antibodies for the oral bacterium Tannerella forsythia predict cardiovascular disease mortality in men with myocardial infarction: A prospective cohort study . Med Hypotheses, 2020,138:109575.

[1119] Luo J W, Lin C H, Zhu Y B, et al. Association of Tongue Bacterial Flora and Subtypes of Liver-Fire Hyperactivity Syndrome in Hypertensive Patients . Evid Based Complement Alternat Med, 2018,2018:9536924.

[1120] Lurbe E, Ingelfinger J. Developmental and Early Life Origins of Cardiometabolic Risk Factors: Novel Findings and Implications . Hypertension, 2021,77(2):308-318.

[1121] Luthold R V, Fernandes G R, Franco-de-Moraes A C, et al. Gut microbiota interactions with the immunomodulatory role of vitamin D in normal individuals . Metabolism, 2017,69:76-86.

[1122] Lv L J, Li S H, Li S C, et al. Early-Onset Preeclampsia Is Associated With Gut Microbial Alterations in Antepartum and Postpartum Women . Front Cell Infect Microbiol, 2019,9:224.

[1123] Lye H S, Kuan C Y, Ewe J A, et al. The improvement of hypertension by probiotics: effects on cholesterol, diabetes, renin, and phytoestrogens . Int J Mol Sci, 2009,10(9):3755-3775.

[1124] Lyte J M. Eating for 3.8 × 10(13): Examining the Impact of Diet and Nutrition on the Microbiota-Gut-Brain Axis Through the Lens of Microbial Endocrinology . Front Endocrinol (Lausanne), 2018,9:796.

[1125] M N P. Early Vascular Ageing - A Concept in Development . Eur Endocrinol, 2015,11(1):26-31.

[1126] Ma J, Li H. The Role of Gut Microbiota in Atherosclerosis and Hypertension . Front Pharmacol, 2018,9:1082.

[1127] Ma W, Li Y, Heianza Y, et al. Associations of Bowel Movement Frequency with Risk of Cardiovascular Disease and Mortality among US Women . Sci Rep, 2016,6:33005.

[1128] Ma Y, Xu X, Li M, et al. Gut microbiota promote the inflammatory response in the pathogenesis of systemic lupus erythematosus . Mol Med, 2019,25(1):35.

[1129] Maalej B, Ben A M, Jallouli M, et al. [Post-streptococcal glomerulonephritis in the south of Tunisia: A 12-year retrospective review] . Nephrol Ther, 2018,14(7):518-522.

[1130] Machate D J, Figueiredo P S, Marcelino G, et al. Fatty Acid Diets: Regulation of Gut Microbiota Composition and Obesity and Its Related Metabolic Dysbiosis . Int J Mol Sci, 2020,21(11).

[1131] Macneal P, Milroy C. Paronychia Drainage . 2022.

[1132] Madhi S A, Briner C, Maswime S, et al. Causes of stillbirths among women from South Africa: a prospective, observational study . Lancet Glob Health, 2019,7(4):e503-e512.

[1133] Madhi S A, Pathirana J, Baillie V, et al. An Observational Pilot Study Evaluating the Utility of Minimally Invasive Tissue Sampling to Determine the Cause of Stillbirths in South African Women . Clin Infect Dis, 2019,69(Suppl 4):S342-S350.

[1134] Madhur M S, Elijovich F, Alexander M R, et al. Hypertension: Do Inflammation and Immunity Hold the Key to Solving this Epidemic? . Circ Res, 2021,128(7):908-933.

[1135] Madsen B S, Havelund T, Krag A. Targeting the gut-liver axis in cirrhosis: antibiotics and non-selective β-blockers . Adv Ther, 2013,30(7):659-670.

[1136] Maeda H, Zhu X, Omura K, et al. Effects of an exopolysaccharide (kefiran) on lipids, blood pressure, blood glucose, and constipation . Biofactors, 2004,22(1-4):197-200.

[1137] Maeda H, Zhu X, Suzuki S, et al. Structural characterization and biological activities of an exopolysaccharide kefiran produced by Lactobacillus kefiranofaciens WT-2B(T) . J Agric Food Chem, 2004,52(17):5533-5538.

[1138] Maeno M, Yamamoto N, Takano T. Identification of an antihypertensive peptide from casein hydrolysate produced by a proteinase from Lactobacillus helveticus CP790 . J Dairy Sci, 1996,79(8):1316-1321.

[1139] Mafra D, Kalantar-Zadeh K, Moore L W. New Tricks for Old Friends: Treating Gut Microbiota of Patients With CKD . J Ren Nutr, 2021,31(5):433-437.

[1140] Magalhães N S, Savino W, Silva P, et al. Gut Microbiota Dysbiosis Is a Crucial Player for the Poor Outcomes for COVID-19 in Elderly, Diabetic and Hypertensive Patients . Front Med (Lausanne), 2021,8:644751.

[1141] Magruder M, Edusei E, Zhang L, et al. Gut commensal microbiota and decreased risk for Enterobacteriaceae bacteriuria and urinary tract infection . Gut Microbes, 2020,12(1):1805281.

[1142] Magruder M, Sholi A N, Gong C, et al. Gut uropathogen abundance is a risk factor for development of bacteriuria and urinary tract infection . Nat Commun, 2019,10(1):5521.

[1143] Maharaj S, Seegobin K, Chrzanowski S, et al. Acute glomerulonephritis secondary to Streptococcus anginosus . BMJ Case Rep, 2018,2018.

[1144] Mahé E, Zimmermann U. [Significant improvement in ulcerative necrobiosis lipoidica with doxycycline] . Ann Dermatol Venereol, 2011,138(10):686-688.

[1145] Maifeld A, Bartolomaeus H, Löber U, et al. Fasting alters the gut microbiome reducing blood pressure and body weight in metabolic syndrome patients . Nat Commun, 2021,12(1):1970.

[1146] Majcherczyk P A, Langen H, Heumann D, et al. Digestion of Streptococcus pneumoniae cell walls with its major peptidoglycan hydrolase releases branched stem peptides carrying proinflammatory activity . J Biol Chem, 1999,274(18):12537-12543.

[1147] Malin S G, Shavva V S, Tarnawski L, et al. Functions of acetylcholine-producing lymphocytes in immunobiology . Curr Opin Neurobiol, 2020,62:115-121.

[1148] Malkov S V, Markelov V V, Polozov G Y, et al. Significant delay of lethal outcome in cancer patients due to peroral administration of Bacillus oligonitrophilus KU-1 . ScientificWorldJournal, 2006,6:2177-2187.

[1149] Man A, Li H, Xia N. Resveratrol and the Interaction between Gut Microbiota and Arterial Remodelling . Nutrients, 2020,12(1).

[1150] Manandhar I, Alimadadi A, Aryal S, et al. Gut microbiome-based supervised machine learning for clinical diagnosis of inflammatory bowel diseases . Am J Physiol Gastrointest Liver Physiol, 2021,320(3):G328-G337.

[1151] Maness D L, Martin M, Mitchell G. Poststreptococcal Illness: Recognition and Management . Am Fam Physician, 2018,97(8):517-522.

[1152] Manithody C S, Van Nispen J, Murali V, et al. Role of Bile Acids and Gut Microbiota in Parenteral Nutrition Associated Injury . J Hum Nutr (Carson City), 2020,4(1).

[1153] Mann G V. Studies of a surfactant and cholesteremia in the Maasai . Am J Clin Nutr, 1974,27(5):464-469.

[1154] Mansour T R, Alam Y, Dahbour L, et al. Streptococcus Mutans: A Potential Risk Factor in Recurrent Hemorrhagic Stroke . Cureus, 2017,9(5):e1264.

[1155] Manzoor S, Wani S M, Ahmad M S, et al. Role of probiotics and prebiotics in mitigation of different diseases . Nutrition, 2022,96:111602.

[1156] Marchi-Alves L M, Freitas D, de Andrade D, et al. Characterization of Oral Microbiota in Removable Dental Prosthesis Users: Influence of Arterial Hypertension . Biomed Res Int, 2017,2017:3838640.

[1157] Marcone S, Belton O, Fitzgerald D J. Milk-derived bioactive peptides and their health promoting effects: a potential role in atherosclerosis . Br J Clin Pharmacol, 2017,83(1):152-162.

[1158] Marino M, Del B C, Martini D, et al. A Review of Registered Clinical Trials on Dietary (Poly)Phenols: Past Efforts and Possible Future Directions . Foods, 2020,9(11).

[1159] Marques C, Meireles M, Norberto S, et al. High-fat diet-induced obesity Rat model: a comparison between Wistar and Sprague-Dawley Rat . Adipocyte, 2016,5(1):11-21.

[1160] Marques F Z. Missing Heritability of Hypertension and Our Microbiome . Circulation, 2018,138(14):1381-1383.

[1161] Marques F Z, Jama H A, Tsyganov K, et al. Guidelines for Transparency on Gut Microbiome Studies in Essential and Experimental Hypertension . Hypertension, 2019,74(6):1279-1293.

[1162] Marques F Z, Mackay C R, Kaye D M. Beyond gut feelings: how the gut microbiota regulates blood pressure . Nat Rev Cardiol, 2018,15(1):20-32.

[1163] Marrie T J, Low D E, De Carolis E. A comparison of bacteremic pneumococcal pneumonia with nonbacteremic community-acquired pneumonia of any etiology--results from a Canadian multicentre study . Can Respir J, 2003,10(7):368-374.

[1164] Marrie T J, Peeling R W, Reid T, et al. Chlamydia species as a cause of community-acquired pneumonia in Canada . Eur Respir J, 2003,21(5):779-784.

[1165] Marshall-Jones Z V, Baillon M L, Croft J M, et al. Effects of Lactobacillus acidophilus DSM13241 as a probiotic in healthy adult cats . Am J Vet Res, 2006,67(6):1005-1012.

[1166] Martin X D, Duvoisin B. Ocular complications of the Fernand-Widal triad and its therapy . Ophthalmologica, 2003,217(2):160-163.

[1167] Martinčič A, Cemazar M, Sersa G, et al. A novel method for speciation of Pt in human serum incubated with cisplatin, oxaliplatin and carboplatin by conjoint liquid chromatography on monolithic disks with UV and ICP-MS detection . Talanta, 2013,116:141-148.

[1168] Martínez M C, Andriantsitohaina R. Extracellular Vesicles in Metabolic Syndrome . Circ Res, 2017,120(10):1674-1686.

[1169] Martínez-Huélamo M, Tulipani S, Jáuregui O, et al. Sensitive and Rapid UHPLC-MS/MS for the Analysis of Tomato Phenolics in Human Biological Samples . Molecules, 2015,20(11):20409-20425.

[1170] Martínez-Moragón E, García F L, Serra S B, et al. [Community-acquired pneumonia among the elderly: differences between patients living at home and in nursing homes] . Arch Bronconeumol, 2004,40(12):547-552.

[1171] Martingano D, Renson A, Rogoff S, et al. Daily gentamicin using ideal body weight demonstrates lower risk of postpartum endometritis and increased chance of successful outcome compared with traditional 8-hour dosing for the treatment of intrapartum chorioamnionitis . J Matern Fetal Neonatal Med, 2019,32(19):3204-3208.

[1172] Martins L B, Malheiros S A, Teixeira A L. The link between nutrition and Alzheimer's disease: from prevention to treatment . Neurodegener Dis Manag, 2021,11(2):155-166.

[1173] Marto N, Morello J, Antunes A, et al. A simple method to measure sulfonation in man using paracetamol as probe drug . Sci Rep, 2021,11(1):9036.

[1174] Marungruang N, Tovar J, Björck I, et al. Improvement in cardiometabolic risk markers following a multifunctional diet is associated with gut microbial taxa in healthy overweight and obese subjects . Eur J Nutr, 2018,57(8):2927-2936.

[1175] Marzoog B. Lipid behavior in metabolic syndrome pathophysiology . Curr Diabetes Rev, 2021.

[1176] Marzullo P, Di Renzo L, Pugliese G, et al. From obesity through gut microbiota to cardiovascular diseases: a dangerous journey . Int J Obes Suppl, 2020,10(1):35-49.

[1177] Mas-Capdevila A, Teichenne J, Domenech-Coca C, et al. Effect of Hesperidin on Cardiovascular Disease Risk Factors: The Role of Intestinal Microbiota on Hesperidin Bioavailability . Nutrients, 2020,12(5).

[1178] Masek K, Paegelow I, Rasková H, et al. Hemodynamic effects of group A Streptococcus mucopeptide . Proc Soc Exp Biol Med, 1973,144(3):1020-1024.

[1179] Mashaqi S, Gozal D. Obstructive Sleep Apnea and Systemic Hypertension: Gut Dysbiosis as the Mediator? . J Clin Sleep Med, 2019,15(10):1517-1527.

[1180] Maskarinec G, Hullar M, Monroe K R, et al. Fecal Microbial Diversity and Structure Are Associated with Diet Quality in the Multiethnic Cohort Adiposity Phenotype Study . J Nutr, 2019,149(9):1575-1584.

[1181] Maslennikov R, Ivashkin V, Efremova I, et al. Gut-liver axis in cirrhosis: Are hemodynamic changes a missing link? . World J Clin Cases, 2021,9(31):9320-9332.

[1182] Masouris I, Klein M, Dyckhoff S, et al. Inhibition of DAMP signaling as an effective adjunctive treatment strategy in pneumococcal meningitis . J Neuroinflammation, 2017,14(1):214.

[1183] Massarani E, Nardi D, Degen L, et al. Antiviral compounds. 8. Aminoacethydrazones of aromatic alpha-ketoaldehydes . J Med Chem, 1966,9(4):617-618.

[1184] Mastan A, Bharadwaj R, Kushwaha R K, et al. Functional Fungal Endophytes in Coleus forskohlii Regulate Labdane Diterpene Biosynthesis for Elevated Forskolin Accumulation in Roots . Microb Ecol, 2019,78(4):914-926.

[1185] Matamala J M, Núñez C, Ogrodnik R, et al. [Bifrontal cerebritis and brain abscess caused by Sreptococcus anginosus group: report of one case] . Rev Med Chil, 2013,141(1):109-113.

[1186] Matías-Pérez D, Hernández-Bautista E, García-Montalvo I A. Intermittent fasting may optimize intestinal microbiota, adipocyte status and metabolic health . Asia Pac J Clin Nutr, 2022,31(1):16-23.

[1187] Matsumoto T, Kojima M, Takayanagi K, et al. Role of S-Equol, Indoxyl Sulfate, and Trimethylamine N-Oxide on Vascular Function . Am J Hypertens, 2020,33(9):793-803.

[1188] Matsuoka T, Shimizu T, Minagawa T, et al. First case of an invasive Bacteroides dorei infection detected in a patient with a mycotic aortic aneurysm-raising a rebellion of major indigenous bacteria in humans: a case report and review . BMC Infect Dis, 2021,21(1):625.

[1189] Matsutomo T. Potential benefits of garlic and other dietary supplements for the management of hypertension . Exp Ther Med, 2020,19(2):1479-1484.

[1190] Matsuzaki K, Takigami K, Matsuura H, et al. [Infected Thoracic Aortic Aneurysm Secondary to the Purulent Pericarditis;Report of a Case] . Kyobu Geka, 2018,71(12):1023-1026.

[1191] May A M, Riede F N, Riede U N. Acute subepicardial infarction associated with severe septic shock--insight in myocardial perfusion . Pathol Res Pract, 2010,206(6):401-404.

[1192] Mazel F. Living the high life: Could gut microbiota matter for adaptation to high altitude? . Mol Ecol, 2019,28(9):2119-2121.

[1193] Mazidi M, Mikhailidis D P, Sattar N, et al. Consumption of dairy product and its association with total and cause specific mortality - A population-based cohort study and meta-analysis . Clin Nutr, 2019,38(6):2833-2845.

[1194] Mazidi M, Rezaie P, Kengne A P, et al. Gut microbiome and metabolic syndrome . Diabetes Metab Syndr, 2016,10(2 Suppl 1):S150-S157.

[1195] Mazzoccoli G, De Cosmo S, Mazza T. The Biological Clock: A Pivotal Hub in Non-alcoholic Fatty Liver Disease Pathogenesis . Front Physiol, 2018,9:193.

[1196] McCarthy C G, Saha P, Golonka R M, et al. Innate Immune Cells and Hypertension: Neutrophils and Neutrophil Extracellular Traps (NETs) . Compr Physiol, 2021,11(1):1575-1589.

[1197] McCoy J A, Elovitz M A, Alby K, et al. Association of Obesity With Maternal and Cord Blood Penicillin Levels in Women With Group B Streptococcus Colonization . Obstet Gynecol, 2020,136(4):756-764.

[1198] McGrattan A M, McGuinness B, McKinley M C, et al. Diet and Inflammation in Cognitive Ageing and Alzheimer's Disease . Curr Nutr Rep, 2019,8(2):53-65.

[1199] McKeown N M, Livingston K A, Sawicki C M, et al. Evidence mapping to assess the available research on fiber, whole grains, and health . Nutr Rev, 2020,78(Suppl 1):37-42.

[1200] McKernan D P, Fitzgerald P, Dinan T G, et al. The probiotic Bifidobacterium infantis 35624 displays visceral antinociceptive effects in the rat . Neurogastroenterol Motil, 2010,22(9):1029-1035, e268.

[1201] McMartin K E. Increased urinary folate excretion and decreased plasma folate levels in the rat after acute ethanol treatment . Alcohol Clin Exp Res, 1984,8(2):172-178.

[1202] Meadow W L, Meus P J. Hemodynamic consequences of tolazoline in neonatal group B streptococcal bacteremia: an animal model . Pediatr Res, 1984,18(10):960-965.

[1203] Meadow W L, Meus P J. Early and late hemodynamic consequences of group B beta streptococcal sepsis in piglets: effects on systemic, pulmonary, and mesenteric circulations . Circ Shock, 1986,19(4):347-356.

[1204] Meadow W L, Meus P J. Unsuspected mesenteric hypoperfusion despite apparent hemodynamic recovery in the early phase of septic shock in piglets . Circ Shock, 1985,15(2):123-129.

[1205] Meadow W L, Rudinsky B F, Strates E. Selective elevation of systemic blood pressure by epinephrine during sepsis-induced pulmonary hypertension in piglets . Pediatr Res, 1986,20(9):872-875.

[1206] Meadow W L, Rudinsky B F, Strates E. Effects of phenylephrine on systemic and pulmonary artery pressure during sepsis-induced pulmonary hypertension in piglets . Dev Pharmacol Ther, 1986,9(4):249-259.

[1207] Meadow W L, Rudinsky B F, Strates E, et al. Oxygen delivery, oxygen consumption, and metabolic acidosis during group B streptococcal sepsis in piglets . Pediatr Res, 1987,22(5):509-512.

[1208] Meadow W, Rudinsky B, Bell A, et al. Effects of inhibition of endothelium-derived relaxation factor on hemodynamics and oxygen utilization during group B streptococcal sepsis in piglets . Crit Care Med, 1995,23(4):705-714.

[1209] Mecha E, Feliciano R P, Rodriguez-Mateos A, et al. Human bioavailability of phenolic compounds found in common beans: the use of high-resolution MS to evaluate inter-individual variability . Br J Nutr, 2020,123(3):273-292.

[1210] Mehta G, Gustot T, Mookerjee R P, et al. Inflammation and portal hypertension - the undiscovered country . J Hepatol, 2014,61(1):155-163.

[1211] Mehta G, Mookerjee R P. Breaking bad - the two sides of gut microbiota in portal hypertension . Liver Int, 2014,34(9):1295-1297.

[1212] Meijnikman A S, Aydin O, Prodan A, et al. Distinct differences in gut microbial composition and functional potential from lean to morbidly obese subjects . J Intern Med, 2020,288(6):699-710.

[1213] Meijvis S C, Cornips M C, Endeman H, et al. Prognostic value of serum angiotensin-converting enzyme activity for outcome of community-acquired pneumonia . Clin Chem Lab Med, 2011,49(9):1525-1532.

[1214] Melini F, Melini V, Luziatelli F, et al. Health-Promoting Components in Fermented Foods: An Up-to-Date Systematic Review . Nutrients, 2019,11(5).

[1215] Mell B, Jala V R, Mathew A V, et al. Evidence for a link between gut microbiota and hypertension in the Dahl rat . Physiol Genomics, 2015,47(6):187-197.

[1216] Mena-Sánchez G, Babio N, Becerra-Tomás N, et al. Association between dairy product consumption and hyperuricemia in an elderly population with metabolic syndrome . Nutr Metab Cardiovasc Dis, 2020,30(2):214-222.

[1217] Mena-Sánchez G, Babio N, Martínez-González M Á, et al. Fermented dairy products, diet quality, and cardio-metabolic profile of a Mediterranean cohort at high cardiovascular risk . Nutr Metab Cardiovasc Dis, 2018,28(10):1002-1011.

[1218] Mendeloff J, Stallion A, Hutton M, et al. Aortic aneurysm resulting from umbilical artery catheterization: case report, literature review, and management algorithm . J Vasc Surg, 2001,33(2):419-424.

[1219] Mendelsohn A R, Larrick J W. Dietary modification of the microbiome affects risk for cardiovascular disease . Rejuvenation Res, 2013,16(3):241-244.

[1220] Menke J A, Giacoia G P, Jockin H. Group B beta hemolytic streptococcal sepsis and the idiopathic respiratory distress syndrome: a comparison . J Pediatr, 1979,94(3):467-471.

[1221] Menni C, Lin C, Cecelja M, et al. Gut microbial diversity is associated with lower arterial stiffness in women . Eur Heart J, 2018,39(25):2390-2397.

[1222] Mensah G A, Brown A, Pratt C A. Nutrition Disparities and Cardiovascular Health . Curr Atheroscler Rep, 2020,22(4):15.

[1223] Merra G, Noce A, Marrone G, et al. Influence of Mediterranean Diet on Human Gut Microbiota . Nutrients, 2020,13(1).

[1224] Mersil M, Marzouk M, Labeeb H. Monomicrobial Necrotizing Fasciitis of the Breast: A Case Managed by Partial Mastectomy and Hydrogel Dressing . Cureus, 2021,13(9):e17891.

[1225] Mertineit C, Samlalsingh-Parker J, Glibetic M, et al. Nitric oxide, prostaglandins, and impaired cerebral blood flow autoregulation in group B streptococcal neonatal meningitis . Can J Physiol Pharmacol, 2000,78(3):217-227.

[1226] Messinger-Rapport B J, Cruz-Oliver D M, Thomas D R, et al. Clinical update on nursing home medicine: 2012 . J Am Med Dir Assoc, 2012,13(7):581-594.

[1227] Meyer K, Lulla A, Debroy K, et al. Association of the Gut Microbiota With Cognitive Function in Midlife . JAMA Netw Open, 2022,5(2):e2143941.

[1228] Meyer M W, Gong K, Herzberg M C. Streptococcus sanguis-induced platelet clotting in rabbits and hemodynamic and cardiopulmonary consequences . Infect Immun, 1998,66(12):5906-5914.

[1229] Miao T, Yu Y, Sun J, et al. Decrease in abundance of bacteria of the genus Bifidobacterium in gut microbiota may be related to pre-eclampsia progression in women from East China . Food Nutr Res, 2021,65.

[1230] Michael D R, Jack A A, Masetti G, et al. A randomised controlled study shows supplementation of overweight and obese adults with lactobacilli and bifidobacteria reduces bodyweight and improves well-being . Sci Rep, 2020,10(1):4183.

[1231] Michels N, Zouiouich S, Vanderbauwhede B, et al. Human microbiome and metabolic health: An overview of systematic reviews . Obes Rev, 2022,23(4):e13409.

[1232] Middelveld R J, Alving K. Synergistic septicemic action of the gram-positive bacterial cell wall components peptidoglycan and lipoteichoic acid in the pig in vivo . Shock, 2000,13(4):297-306.

[1233] Migacz-Gruszka K, Branicki W, Obtulowicz A, et al. What's New in the Pathophysiology of Alopecia Areata? The Possible Contribution of Skin and Gut Microbiome in the Pathogenesis of Alopecia - Big Opportunities, Big Challenges, and Novel Perspectives . Int J Trichology, 2019,11(5):185-188.

[1234] Miglis M G, Muppidi S. The microbiome in autonomic medicine and other updates in recent autonomic research . Clin Auton Res, 2019,29(4):361-362.

[1235] Mihalache D, Luca V, Hurmuzache M E, et al. [The streptococcal and staphylococcal toxic shock syndrome. Comments on 4 cases] . Rev Med Chir Soc Med Nat Iasi, 1995,99(3-4):231-234.

[1236] Mikirova N A, Casciari J J, Hunninghake R E, et al. Effect of weight reduction on cardiovascular risk factors and CD34-positive cells in circulation . Int J Med Sci, 2011,8(6):445-452.

[1237] Milner A D. Nitric oxide . Eur J Pediatr, 1994,153(9 Suppl 2):S7-S11.

[1238] Milsom I, Arvidsson L, Ekelund P, et al. Factors influencing vaginal cytology, pH and bacterial flora in elderly women . Acta Obstet Gynecol Scand, 1993,72(4):286-291.

[1239] Minodier P, Bréaud J, Bérard E. [E. coli acute pyelonephritis: prophylaxis] . Arch Pediatr, 2012,19 Suppl 3:S117-S123.

[1240] Minter M R, Hinterleitner R, Meisel M, et al. Antibiotic-induced perturbations in microbial diversity during post-natal development alters amyloid pathology in an aged APP(SWE)/PS1(ΔE9) murine model of Alzheimer's disease . Sci Rep, 2017,7(1):10411.

[1241] Mirpuri J. Evidence for maternal diet-mediated effects on the offspring microbiome and immunity: implications for public health initiatives . Pediatr Res, 2021,89(2):301-306.

[1242] Mirsadykov D A. Cerebrospinal fluid shunt malfunction not associated with ventricular enlargement. A case report and literature review . Zh Vopr Neirokhir Im N N Burdenko, 2016,80(4):81-88.

[1243] Mirsaeidi M, Ebrahimi G, Allen M B, et al. Pneumococcal vaccine and patients with pulmonary diseases . Am J Med, 2014,127(9):881-886.

[1244] Mirzayi C, Renson A, Zohra F, et al. Reporting guidelines for human microbiome research: the STORMS checklist . Nat Med, 2021,27(11):1885-1892.

[1245] Misaki T, Naka S, Hatakeyama R, et al. Presence of Streptococcus mutans strains harbouring the cnm gene correlates with dental caries status and IgA nephropathy conditions . Sci Rep, 2016,6:36455.

[1246] Mishima E, Abe T. Role of the microbiota in hypertension and antihypertensive drug metabolism . Hypertens Res, 2022,45(2):246-253.

[1247] Mishima R S, Elliott A D, Sanders P, et al. Gastrointestinal sodium absorption, microbiome, and hypertension . Nat Rev Cardiol, 2017,14(11):693.

[1248] Mishima R S, Hohl M, Linz B, et al. Too Fatty, Too Salty, Too Western . Hypertension, 2018,72(5):1078-1080.

[1249] Mishra A K, Dubey V, Ghosh A R. Obesity: An overview of possible role(s) of gut hormones, lipid sensing and gut microbiota . Metabolism, 2016,65(1):48-65.

[1250] Mitsuoka T. Intestinal flora and human health . Asia Pac J Clin Nutr, 1996,5(1):2-9.

[1251] Mitten E K, Baffy G. Microbiota transplantation in portal hypertension: promises and pitfalls . Clin Sci (Lond), 2022,136(6):425-429.

[1252] Miyamoto J, Kasubuchi M, Nakajima A, et al. The role of short-chain fatty acid on blood pressure regulation . Curr Opin Nephrol Hypertens, 2016,25(5):379-383.

[1253] Miyamoto J, Ohue-Kitano R, Mukouyama H, et al. Ketone body receptor GPR43 regulates lipid metabolism under ketogenic conditions . Proc Natl Acad Sci U S A, 2019,116(47):23813-23821.

[1254] Mobegi F M, Leong L E, Thompson F, et al. Intestinal microbiology shapes population health impacts of diet and lifestyle risk exposures in Torres Strait Islander communities . Elife, 2020,9.

[1255] Modares M, Tabari M. Phlegmonous gastritis complicated by abdominal compartment syndrome: a case report . BMC Surg, 2021,21(1):5.

[1256] Moghadamrad S, Hassan M, McCoy K D, et al. Attenuated fibrosis in specific pathogen-free microbiota in experimental cholestasis- and toxin-induced liver injury . FASEB J, 2019,33(11):12464-12476.

[1257] Mohamed M, Patel S, Plavnik K, et al. Retrospective Analysis of Septic Arthritis Caused by Intra-Articular Viscosupplementation and Steroid Injections in a Single Outpatient Center . J Clin Med Res, 2019,11(7):480-483.

[1258] Mohan C R, Hoballah J J, Martinasevic M, et al. The aortic polytetrafluoroethylene graft: further experience . Eur J Vasc Endovasc Surg, 1996,11(2):158-163.

[1259] Mohotti J E, Chan S Q, Nonaka F. A case of bilateral endogenous bacterial endophthalmitis from Streptococcus pneumoniae bacteraemia . Med J Aust, 2016,204(2):79-80.

[1260] Mok C C. Metabolic syndrome and systemic lupus erythematosus: the connection . Expert Rev Clin Immunol, 2019,15(7):765-775.

[1261] Moldovan D C, Ismaiel A, Fagoonee S, et al. Gut microbiota and cardiovascular diseases axis . Minerva Med, 2022,113(1):189-199.

[1262] Molinos L, Zalacain R, Menéndez R, et al. Sensitivity, Specificity, and Positivity Predictors of the Pneumococcal Urinary Antigen Test in Community-Acquired Pneumonia . Ann Am Thorac Soc, 2015,12(10):1482-1489.

[1263] Mölzer C, Heissigerova J, Wilson H M, et al. Immune Privilege: The Microbiome and Uveitis . Front Immunol, 2020,11:608377.

[1264] Montandon S A, Jornayvaz F R. Effects of Antidiabetic Drugs on Gut Microbiota Composition . Genes (Basel), 2017,8(10).

[1265] Montgomery D M, Stedman C M, Robichaux A R, et al. Cord blood gas patterns identifying newborns at increased risk of group B streptococcal sepsis . Obstet Gynecol, 1991,78(5 Pt 1):774-777.

[1266] Moraleda C, Benmessaoud R, Esteban J, et al. Prevalence, antimicrobial resistance and serotype distribution of group B streptococcus isolated among pregnant women and newborns in Rabat, Morocco . J Med Microbiol, 2018,67(5):652-661.

[1267] Morato-Martínez M, López-Plaza B, Santurino C, et al. A Dairy Product to Reconstitute Enriched with Bioactive Nutrients Stops Bone Loss in High-Risk Menopausal Women without Pharmacological Treatment . Nutrients, 2020,12(8).

[1268] Moreno-Montoro M, Olalla-Herrera M, Rufián-Henares J Á, et al. Antioxidant, ACE-inhibitory and antimicrobial activity of fermented goat milk: activity and physicochemical property relationship of the peptide components . Food Funct, 2017,8(8):2783-2791.

[1269] Morou-Bermúdez E, Torres-Colón J E, Bermúdez N S, et al. Pathways Linking Oral Bacteria, Nitric Oxide Metabolism, and Health . J Dent Res, 2022:503877883.

[1270] Morris D J, Brem A S, Odermatt A. Modulation of 11β-hydroxysteroid dehydrogenase functions by the cloud of endogenous metabolites in a local microenvironment: The glycyrrhetinic acid-like factor (GALF) hypothesis . J Steroid Biochem Mol Biol, 2021,214:105988.

[1271] Mosalem O, Rous F A, Al-Abcha A, et al. Pyomyositis as an Unusual Presentation of Colonic Adenocarcinoma . Perm J, 2020,25:1.

[1272] Moszak M, Szulińska M, Walczak-Gałęzewska M, et al. Nutritional Approach Targeting Gut Microbiota in NAFLD-To Date . Int J Environ Res Public Health, 2021,18(4).

[1273] Moutsoglou D M. 2021 American Thoracic Society BEAR Cage Winning Proposal: Microbiome Transplant in Pulmonary Arterial Hypertension . Am J Respir Crit Care Med, 2022,205(1):13-16.

[1274] Moyle P M, Olive C, Good M F, et al. Method for the synthesis of highly pure vaccines using the lipid core peptide system . J Pept Sci, 2006,12(12):800-807.

[1275] Muder R R. Pneumonia in residents of long-term care facilities: epidemiology, etiology, management, and prevention . Am J Med, 1998,105(4):319-330.

[1276] Mukohda M, Nakamura S, Takeya K, et al. Streptococcal Exotoxin Streptolysin O Causes Vascular Endothelial Dysfunction Through PKCβ Activation . J Pharmacol Exp Ther, 2021,379(2):117-124.

[1277] Mullins A P, Arjmandi B H. Health Benefits of Plant-Based Nutrition: Focus on Beans in Cardiometabolic Diseases . Nutrients, 2021,13(2).

[1278] Münch N S, Fang H Y, Ingermann J, et al. High-Fat Diet Accelerates Carcinogenesis in a Mouse Model of Barrett's Esophagus via Interleukin 8 and Alterations to the Gut Microbiome . Gastroenterology, 2019,157(2):492-506.

[1279] Münzel T, Sørensen M, Daiber A. Transportation noise pollution and cardiovascular disease . Nat Rev Cardiol, 2021,18(9):619-636.

[1280] Murad A J, Cohen R V, de Godoy E P, et al. A Prospective Single-Arm Trial of Modified Long Biliopancreatic and Short Alimentary Limbs Roux-En-Y Gastric Bypass in Type 2 Diabetes Patients with Mild Obesity . Obes Surg, 2018,28(3):599-605.

[1281] Murakami M, Iwamoto J, Honda A, et al. Detection of Gut Dysbiosis due to Reduced Clostridium Subcluster XIVa Using the Fecal or Serum Bile Acid Profile . Inflamm Bowel Dis, 2018,24(5):1035-1044.

[1282] Muralidharan R, Rabinstein A A, Wijdicks E F. Cervicomedullary injury after pneumococcal meningitis with brain edema . Arch Neurol, 2011,68(4):513-516.

[1283] Muralitharan R R, Jama H A, Xie L, et al. Microbial Peer Pressure: The Role of the Gut Microbiota in Hypertension and Its Complications . Hypertension, 2020,76(6):1674-1687.

[1284] Muralitharan R R, Nakai M E, Marques F Z. The conundrum of the gut microbiome and blood pressure: the importance of studying sex and ethnicity . Eur Heart J, 2020,41(44):4268-4270.

[1285] Murphy K, O'Donovan A N, Caplice N M, et al. Exploring the Gut Microbiota and Cardiovascular Disease . Metabolites, 2021,11(8).

[1286] Murphy T D, Gibson R L, Standaert T A, et al. Effect of group B streptococcal sepsis on diaphragmatic function in young piglets . Pediatr Res, 1993,33(1):10-14.

[1287] Murphy T D, Gibson R L, Standaert T A, et al. Diaphragmatic failure during group B streptococcal sepsis in piglets: the role of thromboxane A2 . J Appl Physiol (1985), 1995,78(2):491-498.

[1288] Murphy T D, Mayock D E, Standaert T A, et al. Group B streptococcus has no effect on piglet diaphragmatic force generation . Am Rev Respir Dis, 1992,145(2 Pt 1):471-475.

[1289] Mushtaq N, Hussain S, Zhang S, et al. Molecular characterization of alterations in the intestinal microbiota of patients with grade 3 hypertension . Int J Mol Med, 2019,44(2):513-522.

[1290] Mustafa F, Chopra H, Baig A A, et al. Edible Mushrooms as Novel Myco-Therapeutics: Effects on Lipid Level, Obesity and BMI . J Fungi (Basel), 2022,8(2).

[1291] Nadkarni M A, Deshpande N P, Wilkins M R, et al. Intra-species variation within Lactobacillus rhamnosus correlates to beneficial or harmful outcomes: lessons from the oral cavity . BMC Genomics, 2020,21(1):661.

[1292] Nadon C A, Ismond M A, Holley R. Biogenic amines in vacuum-packaged and carbon dioxide-controlled atmosphere-packaged fresh pork stored at -1.50 degrees C . J Food Prot, 2001,64(2):220-227.

[1293] Nagayama Y, Iwasaki S, Yamaguchi H, et al. [Infective endocarditis successfully treated by early medical therapy in a patient with Henoch-Schönlein purpura nephritis under oral steroid therapy] . Nihon Jinzo Gakkai Shi, 2007,49(4):452-458.

[1294] Nakai M, Ribeiro R V, Stevens B R, et al. Essential Hypertension Is Associated With Changes in Gut Microbial Metabolic Pathways: A Multisite Analysis of Ambulatory Blood Pressure . Hypertension, 2021,78(3):804-815.

[1295] Nakajima H, Takewaki F, Hashimoto Y, et al. The Effects of Metformin on the Gut Microbiota of Patients with Type 2 Diabetes: A Two-Center, Quasi-Experimental Study . Life (Basel), 2020,10(9).

[1296] Nakajima Y, Takenaka K, Watanabe F, et al. [A patient with mitral stenosis due to infective endocarditis] . J Cardiol, 1997,29 Suppl 2:125-128.

[1297] Nakamura Y, Watanabe H, Tanaka A, et al. Effect of Increased Daily Water Intake and Hydration on Health in Japanese Adults . Nutrients, 2020,12(4).

[1298] Nakamura Y, Yamamoto N, Sakai K, et al. Antihypertensive effect of sour milk and peptides isolated from it that are inhibitors to angiotensin I-converting enzyme . J Dairy Sci, 1995,78(6):1253-1257.

[1299] Nakao M, Yamamoto I, Maruyama Y, et al. 33 Years of Peritoneal Dialysis-Associated Peritonitis: A Single-Center Study in Japan . Ther Apher Dial, 2016,20(1):60-65.

[1300] Nakashima S, Miyamoto A, Takahashi Y, et al. Mendelson's syndrome complicated by bacterial aspiration pneumonia triggered by right putamen bleeding: A case report . Respir Med Case Rep, 2021,33:101466.

[1301] Namazi G, Gupta S, Einarsson J I. Deep Pelvic Side Wall Anatomy: A Case of Laparoscopic Management of Vaginal Vault Fistula to the Presacral Area . J Minim Invasive Gynecol, 2022.

[1302] Naqvi S, Asar T O, Kumar V, et al. A cross-talk between gut microbiome, salt and hypertension . Biomed Pharmacother, 2021,134:111156.

[1303] Naruszewicz M, Johansson M L, Zapolska-Downar D, et al. Effect of Lactobacillus plantarum 299v on cardiovascular disease risk factors in smokers . Am J Clin Nutr, 2002,76(6):1249-1255.

[1304] Narva M, Nevala R, Poussa T, et al. The effect of Lactobacillus helveticus fermented milk on acute changes in calcium metabolism in postmenopausal women . Eur J Nutr, 2004,43(2):61-68.

[1305] Nasiri G, Bastani A, Haji-Aghamohammadi A A, et al. Effects of probiotic and alpha-lipoic acid supplements, separately or in combination on the anthropometric indicators and maintenance of weight in overweight individuals . Clin Nutr ESPEN, 2021,41:242-248.

[1306] Natarajan N, Hori D, Flavahan S, et al. Microbial short chain fatty acid metabolites lower blood pressure via endothelial G protein-coupled receptor 41 . Physiol Genomics, 2016,48(11):826-834.

[1307] Navarrete C T, Devia C, Lessa A C, et al. The role of endothelin converting enzyme inhibition during group B streptococcus-induced pulmonary hypertension in newborn piglets . Pediatr Res, 2003,54(3):387-392.

[1308] Navarrete D, Patil S, Dandachi D. Acute Streptococcus constellatus Pyogenic Liver Abscess Due to an Atypical Presentation of Sigmoid Diverticulitis Complicated by Pericolonic Abscess . Cureus, 2020,12(10):e10940.

[1309] Nawaz K, David S M, Murugesh E, et al. Identification and in silico characterization of a novel peptide inhibitor of angiotensin converting enzyme from pigeon pea (Cajanus cajan) . Phytomedicine, 2017,36:1-7.

[1310] Ndlangisa K M, du Plessis M, Allam M, et al. Two cases of serotypeable and non-serotypeable variants of Streptococcus pneumoniae detected simultaneously during invasive disease . BMC Microbiol, 2016,16(1):126.

[1311] Nebbia S, Lamberti C, Lo B G, et al. Antimicrobial Potential of Food Lactic Acid Bacteria: Bioactive Peptide Decrypting from Caseins and Bacteriocin Production . Microorganisms, 2020,9(1).

[1312] Neill A M, Martin I R, Weir R, et al. Community acquired pneumonia: aetiology and usefulness of severity criteria on admission . Thorax, 1996,51(10):1010-1016.

[1313] Nelson M, Stankard B, Greco J, et al. Point of Care Ultrasound Diagnosis of Empyema . J Emerg Med, 2016,51(2):140-143.

[1314] New F N, Baer B R, Clark A G, et al. Collective effects of human genomic variation on microbiome function . Sci Rep, 2022,12(1):3839.

[1315] Ng C C, Wang C Y, Wang Y P, et al. Lactic acid bacterial fermentation on the production of functional antioxidant herbal Anoectochilus formosanus Hayata . J Biosci Bioeng, 2011,111(3):289-293.

[1316] Nguyen H, Gathercole J L, Day L, et al. Differences in peptide generation following in vitro gastrointestinal digestion of yogurt and milk from cow, sheep and goat . Food Chem, 2020,317:126419.

[1317] Nguyen T, Jin Y Y, Chung H J, et al. Pharmabiotics as an Emerging Medication for Metabolic Syndrome and Its Related Diseases . Molecules, 2017,22(10).

[1318] Ni J, Fu C, Huang R, et al. Metabolic syndrome cannot mask the changes of faecal microbiota compositions caused by primary hepatocellular carcinoma . Lett Appl Microbiol, 2021,73(1):73-80.

[1319] Nichols A W. Probiotics and athletic performance: a systematic review . Curr Sports Med Rep, 2007,6(4):269-273.

[1320] Nicolau D P, Sutherland C A, Arguedas A, et al. Pharmacokinetics of cefprozil in plasma and middle ear fluid: in children undergoing treatment for acute otitis media . Paediatr Drugs, 2007,9(2):119-123.

[1321] Nicoletti A, Ponziani F R, Biolato M, et al. Intestinal permeability in the pathogenesis of liver damage: From non-alcoholic fatty liver disease to liver transplantation . World J Gastroenterol, 2019,25(33):4814-4834.

[1322] Nie J, Xie L, Zhao B X, et al. Serum Trimethylamine N-Oxide Concentration Is Positively Associated With First Stroke in Hypertensive Patients . Stroke, 2018,49(9):2021-2028.

[1323] Nie X, Chen J, Ma X, et al. A metagenome-wide association study of gut microbiome and visceral fat accumulation . Comput Struct Biotechnol J, 2020,18:2596-2609.

[1324] Nikkhah A. Eating time modulations of physiology and health: life lessons from human and ruminant models . Iran J Basic Med Sci, 2012,15(4):891-899.

[1325] Nili F, McLeod L, O'Connell C, et al. Outcomes of pregnancies in women with suspected antiphospholipid syndrome . J Neonatal Perinatal Med, 2013,6(3):225-230.

[1326] Noble J M, Scarmeas N, Celenti R S, et al. Serum IgG antibody levels to periodontal microbiota are associated with incident Alzheimer disease . PLoS One, 2014,9(12):e114959.

[1327] Noce A, Marrone G, Di Daniele F, et al. Impact of Gut Microbiota Composition on Onset and Progression of Chronic Non-Communicable Diseases . Nutrients, 2019,11(5).

[1328] Nogal A, Valdes A M, Menni C. The role of short-chain fatty acids in the interplay between gut microbiota and diet in cardio-metabolic health . Gut Microbes, 2021,13(1):1-24.

[1329] Nögel S C, Chada M, Schmidt A M, et al. Parecoxib does not suppress thromboxane synthesis in newborn piglets with group B streptococcal sepsis . Prostaglandins Other Lipid Mediat, 2009,90(1-2):7-12.

[1330] Nogueira-de-Almeida C A, Del C L, Ferraz I S, et al. COVID-19 and obesity in childhood and adolescence: a clinical review . J Pediatr (Rio J), 2020,96(5):546-558.

[1331] Noor R, Naz A, Maniha S M, et al. Microorganisms and cardiovascular diseases: importance of gut bacteria . Front Biosci (Landmark Ed), 2021,26(5):22-28.

[1332] Nordby J A. Neurological presentation of poststreptococcal glomerulonephritis . Clin Pediatr (Phila), 1997,36(2):105-108.

[1333] Noris M, Bresin E, Mele C, et al. Genetic Atypical Hemolytic-Uremic Syndrome . 1993.

[1334] Norlander A E, Madhur M S, Harrison D G. The immunology of hypertension . J Exp Med, 2018,215(1):21-33.

[1335] Norman K, Pirlich M. Gastrointestinal tract in liver disease: which organ is sick? . Curr Opin Clin Nutr Metab Care, 2008,11(5):613-619.

[1336] Nova P, Pimenta-Martins A, Laranjeira S J, et al. Health benefits and bioavailability of marine resources components that contribute to health - what's new? . Crit Rev Food Sci Nutr, 2020,60(21):3680-3692.

[1337] Nowiński A, Ufnal M. Trimethylamine N-oxide: A harmful, protective or diagnostic marker in lifestyle diseases? . Nutrition, 2018,46:7-12.

[1338] Nozu T, Okumura T. Pathophysiological Commonality Between Irritable Bowel Syndrome and Metabolic Syndrome: Role of Corticotropin-releasing Factor-Toll-like Receptor 4-Proinflammatory Cytokine Signaling . J Neurogastroenterol Motil, 2022,28(2):173-184.

[1339] Nuli R, Cai J, Kadeer A, et al. Integrative Analysis Toward Different Glucose Tolerance-Related Gut Microbiota and Diet . Front Endocrinol (Lausanne), 2019,10:295.

[1340] Nuñez-Cornejo C, Borrás-Blasco J, Gracia-Perez A, et al. Septic shock and community-acquired pneumonia associated with etanercept therapy . Int J Clin Pharmacol Ther, 2008,46(4):193-197.

[1341] Nussberger J. [Blood pressure lowering tripeptides derived from milk protein] . Ther Umsch, 2007,64(3):177-179.

[1342] Obaroakpo J U, Liu L, Zhang S, et al. α-Glucosidase and ACE dual inhibitory protein hydrolysates and peptide fractions of sprouted quinoa yoghurt beverages inoculated with Lactobacillus casei . Food Chem, 2019,299:124985.

[1343] Obrenovich M E, Donskey C J, Schiefer I T, et al. Quantification of phenolic acid metabolites in humans by LC-MS: a structural and targeted metabolomics approach . Bioanalysis, 2018,10(19):1591-1608.

[1344] O'Brien W F, Golden S M, Bibro M C, et al. Short-term responses in neonatal lambs after infusion of group B streptococcal extract . Obstet Gynecol, 1985,65(6):802-806.

[1345] O'Connor C T, Zaman J, McCarthy J, et al. Rheumatic pericarditis: a rare cause of constrictive pericarditis . BMJ Case Rep, 2021,14(1).

[1346] O'Connor K M, Lucking E F, Cryan J F, et al. Bugs, breathing and blood pressure: microbiota-gut-brain axis signalling in cardiorespiratory control in health and disease . J Physiol, 2020,598(19):4159-4179.

[1347] O'Connor S, Chouinard-Castonguay S, Gagnon C, et al. Prebiotics in the management of components of the metabolic syndrome . Maturitas, 2017,104:11-18.

[1348] Oda K, Yatera K, Fujino Y, et al. Respiratory comorbidities and risk of mortality in hospitalized patients with idiopathic pulmonary fibrosis . Respir Investig, 2018,56(1):64-71.

[1349] O'Donovan A N, Herisson F M, Fouhy F, et al. Gut microbiome of a porcine model of metabolic syndrome and HF-pEF . Am J Physiol Heart Circ Physiol, 2020,318(3):H590-H603.

[1350] Ogawa-Ochiai K, Kawasaki K. Panax ginseng for Frailty-Related Disorders: A Review . Front Nutr, 2018,5:140.

[1351] Oh W. Early onset neonatal group B streptococcal sepsis . Am J Perinatol, 2013,30(2):143-147.

[1352] Ohsawa N, Nakaoka Y, Kubokawa S I, et al. Subacute effusive-constrictive pericarditis: Echocardiography-guided diagnosis and management . J Cardiol Cases, 2017,16(1):14-17.

[1353] Ohtaki H, Ohkusu K, Ohta H, et al. A case of sepsis caused by Streptococcus canis in a dog owner: a first case report of sepsis without dog bite in Japan . J Infect Chemother, 2013,19(6):1206-1209.

[1354] Ohue-Kitano R, Yasuoka Y, Goto T, et al. α-Linolenic acid-derived metabolites from gut lactic acid bacteria induce differentiation of anti-inflammatory M2 macrophages through G protein-coupled receptor 40 . FASEB J, 2018,32(1):304-318.

[1355] Okabe M, Tsuboi N, Yokoo T, et al. A case of idiopathic membranoproliferative glomerulonephritis with a transient glomerular deposition of nephritis-associated plasmin receptor antigen . Clin Exp Nephrol, 2012,16(2):337-341.

[1356] Okabe T, Kumagai N, Serizawa H, et al. [A case of toxic shock-like syndrome due to Streptococcus pyogenes] . Kansenshogaku Zasshi, 1995,69(9):1012-1016.

[1357] Okamoto T, Hatakeyama S, Imai A, et al. The association between gut microbiome and erectile dysfunction: a community-based cross-sectional study in Japan . Int Urol Nephrol, 2020,52(8):1421-1428.

[1358] Okazaki T, Hifumi T, Manabe A, et al. Invasive group B streptococcal infection in a patient with post splenectomy for hypersplenism secondary to liver cirrhosis and portal hypertension . World J Emerg Med, 2016,7(1):68-70.

[1359] Okesene-Gafa K A, Moore A E, Jordan V, et al. Probiotic treatment for women with gestational diabetes to improve maternal and infant health and well-being . Cochrane Database Syst Rev, 2020,6(6):D12970.

[1360] Oketch-Rabah H A, Madden E F, Roe A L, et al. United States Pharmacopeia (USP) Safety Review of Gamma-Aminobutyric Acid (GABA) . Nutrients, 2021,13(8).

[1361] Oki T, Kano M, Watanabe O, et al. Effect of consuming a purple-fleshed sweet potato beverage on health-related biomarkers and safety parameters in Caucasian subjects with elevated levels of blood pressure and liver function biomarkers: a 4-week, open-label, non-comparative trial . Biosci Microbiota Food Health, 2016,35(3):129-136.

[1362] Okubo K, Koido N, Obana M, et al. [A case of brain abscess accompanied with sudden-onset hemiplegia as initial manifestation] . Kansenshogaku Zasshi, 1998,72(11):1232-1235.

[1363] Olaniyi K S, Moodley J, Mahabeer Y, et al. Placental Microbial Colonization and Its Association With Pre-eclampsia . Front Cell Infect Microbiol, 2020,10:413.

[1364] Olivares P, Pacheco A, Aranha L N, et al. Gut microbiota of adults with different metabolic phenotypes . Nutrition, 2021,90:111293.

[1365] Oliveira A C, Richards E M, Raizada M K. Pulmonary hypertension: Pathophysiology beyond the lung . Pharmacol Res, 2020,151:104518.

[1366] Oliveira A J, de Farias L D, Mafra V, et al. The Angiotensin Converting Enzyme 2 (ACE2), Gut Microbiota, and Cardiovascular Health . Protein Pept Lett, 2017,24(9):827-832.

[1367] Oliveira L P, Guimarães V, Oliveira J R, et al. Genetic deletion of the angiotensin-(1-7) receptor Mas leads to alterations in gut villi length modulating TLR4/PI3K/AKT and produces microbiome dysbiosis . Neuropeptides, 2020,82:102056.

[1368] Oliveira M C, Oliveira V M, Vieira A C, et al. In vivo assessment of the effect of an adhesive for complete dentures on colonisation of Candida species . Gerodontology, 2010,27(4):303-307.

[1369] Oliveira P R, Leonhardt M C, Carvalho V C, et al. Incidence and risk factors associated with infection after intramedullary nailing of femoral and tibial diaphyseal fractures: Prospective study . Injury, 2018,49(10):1905-1911.

[1370] O'Morain V L, Ramji D P. The Potential of Probiotics in the Prevention and Treatment of Atherosclerosis . Mol Nutr Food Res, 2020,64(4):e1900797.

[1371] Ondarza M A, Sotelo F. Neutral glycolipids in adult rabbit blood and analysis of their function as specific receptors for micro-organisms . Biomed Chromatogr, 1996,10(1):6-10.

[1372] O'Neal H J, Niven A S, Karam G H. Critical Illness in Patients With Asplenia . Chest, 2016,150(6):1394-1402.

[1373] Ong B B, Gole G A, Robertson T, et al. Retinal hemorrhages associated with meningitis in a child with a congenital disorder of glycosylation . Forensic Sci Med Pathol, 2009,5(4):307-312.

[1374] Onishi J C, Häggblom M M, Shapses S A. Can Dietary Fatty Acids Affect the COVID-19 Infection Outcome in Vulnerable Populations? . mBio, 2020,11(4).

[1375] Onyszkiewicz M, Gawrys-Kopczynska M, Konopelski P, et al. Butyric acid, a gut bacteria metabolite, lowers arterial blood pressure via colon-vagus nerve signaling and GPR41/43 receptors . Pflugers Arch, 2019,471(11-12):1441-1453.

[1376] Onyszkiewicz M, Jaworska K, Ufnal M. Short chain fatty acids and methylamines produced by gut microbiota as mediators and markers in the circulatory system . Exp Biol Med (Maywood), 2020,245(2):166-175.

[1377] Organ C L, Otsuka H, Bhushan S, et al. Choline Diet and Its Gut Microbe-Derived Metabolite, Trimethylamine N-Oxide, Exacerbate Pressure Overload-Induced Heart Failure . Circ Heart Fail, 2016,9(1):e2314.

[1378] Orrego-Lagarón N, Martínez-Huélamo M, Vallverdú-Queralt A, et al. High gastrointestinal permeability and local metabolism of naringenin: influence of antibiotic treatment on absorption and metabolism . Br J Nutr, 2015,114(2):169-180.

[1379] Osawa H, Sugihara N, Ukiya T, et al. Metabolic Syndrome, Lifestyle, and Dental Caries in Japanese School Children . Bull Tokyo Dent Coll, 2015,56(4):233-241.

[1380] Osto M, Lutz T A. Translational value of animal models of obesity-Focus on dogs and cats . Eur J Pharmacol, 2015,759:240-252.

[1381] Osuna-Prieto F J, Martinez-Tellez B, Ortiz-Alvarez L, et al. Elevated plasma succinate levels are linked to higher cardiovascular disease risk factors in young adults . Cardiovasc Diabetol, 2021,20(1):151.

[1382] O'Toole D K. Characteristics and use of okara, the soybean residue from soy milk production--a review . J Agric Food Chem, 1999,47(2):363-371.

[1383] Ou Y, Zhang C, Yao M, et al. Gut Flora: Novel Therapeutic Target of Chinese Medicine for the Treatment of Cardiovascular Diseases . Evid Based Complement Alternat Med, 2019,2019:3719596.

[1384] Ouwehand A C. A review of dose-responses of probiotics in human studies . Benef Microbes, 2017,8(2):143-151.

[1385] Ovalle A, Kakarieka E, Rencoret G, et al. [Risk factors for preterm deliveries in a public hospital] . Rev Med Chil, 2012,140(1):19-29.

[1386] Overby H B, Ferguson J F. Gut Microbiota-Derived Short-Chain Fatty Acids Facilitate Microbiota:Host Cross talk and Modulate Obesity and Hypertension . Curr Hypertens Rep, 2021,23(2):8.

[1387] Ozkurt Z, Erol S, Ertek M, et al. [Streptococcal toxic shock syndrome: a case report] . Mikrobiyol Bul, 2003,37(4):309-312.

[1388] Pacifici A, Pacifici L, Nuzzolese M, et al. The alteration of stress-related physiological parameters after probiotics administration in oral surgeons with different degrees of surgical experience . Clin Ter, 2020,171(3):e197-e208.

[1389] Padmanabhan S, Joe B. Towards Precision Medicine for Hypertension: A Review of Genomic, Epigenomic, and Microbiomic Effects on Blood Pressure in Experimental Rat Models and Humans . Physiol Rev, 2017,97(4):1469-1528.

[1390] Paganin F, Bouvet O, Chanez P, et al. Evaluation of the effects of ambroxol on the ofloxacin concentrations in bronchial tissues in COPD patients with infectious exacerbation . Biopharm Drug Dispos, 1995,16(5):393-401.

[1391] Paganin F, Lilienthal F, Bourdin A, et al. Severe community-acquired pneumonia: assessment of microbial aetiology as mortality factor . Eur Respir J, 2004,24(5):779-785.

[1392] Paju S, Pietiäinen M, Liljestrand J M, et al. Carotid artery calcification in panoramic radiographs associates with oral infections and mortality . Int Endod J, 2021,54(1):15-25.

[1393] Pakhomov N, Baugh J A. The role of diet-derived short-chain fatty acids in regulating cardiac pressure overload . Am J Physiol Heart Circ Physiol, 2021,320(2):H475-H486.

[1394] Palma L, Vaisbich-Guimarães M H, Sridharan M, et al. Thrombotic microangiopathy in children . Pediatr Nephrol, 2022:1-14.

[1395] Palmu J, Lahti L, Niiranen T. Targeting Gut Microbiota to Treat Hypertension: A Systematic Review . Int J Environ Res Public Health, 2021,18(3).

[1396] Palmu J, Salosensaari A, Havulinna A S, et al. Association Between the Gut Microbiota and Blood Pressure in a Population Cohort of 6953 Individuals . J Am Heart Assoc, 2020,9(15):e16641.

[1397] Panahi S, Tremblay A. The Potential Role of Yogurt in Weight Management and Prevention of Type 2 Diabetes . J Am Coll Nutr, 2016,35(8):717-731.

[1398] Panpetch W, Chancharoenthana W, Bootdee K, et al. Lactobacillus rhamnosus L34 Attenuates Gut Translocation-Induced Bacterial Sepsis in Murine Models of Leaky Gut . Infect Immun, 2018,86(1).

[1399] Pardo V, Strauss J, Kramer H, et al. Nephropathy associated with sickle cell anemia: an autologous immune complex nephritis. II. Clinicopathologic study of seven patients . Am J Med, 1975,59(5):650-659.

[1400] Park J S, Seo J H, Youn H S. Gut microbiota and clinical disease: obesity and nonalcoholic Fatty liver disease . Pediatr Gastroenterol Hepatol Nutr, 2013,16(1):22-27.

[1401] Park J Y, Awji E G, Suh J W, et al. Pharmacokinetics, pharmacokinetic-pharmacodynamic relationship, and withdrawal period of amoxicillin sodium in olive flounder (Paralichthys olivaceus) . Xenobiotica, 2016,46(6):522-529.

[1402] Park S R, Lee H W, Hong J W, et al. Enhancement of the killing effect of low-temperature plasma on Streptococcus mutans by combined treatment with gold nanoparticles . J Nanobiotechnology, 2014,12:29.

[1403] Pasini E, Aquilani R, Testa C, et al. Pathogenic Gut Flora in Patients With Chronic Heart Failure . JACC Heart Fail, 2016,4(3):220-227.

[1404] Pasternak J D, Fulford M, Gunnarsson T, et al. An unexpected intracranial pressure crisis: infant brain abscess of unusual aetiology . Childs Nerv Syst, 2009,25(3):377-381.

[1405] Patrignani M, Rinaldi G J, Lupano C E. In vivo effects of Maillard reaction products derived from biscuits . Food Chem, 2016,196:204-210.

[1406] Patz J J, Helm M C, Higgins R M, et al. Peri-operative, intravenous clindamycin may improve the resolution rate of hypertension after Roux-en-Y gastric bypass in morbidly obese patients . Surg Endosc, 2019,33(12):3984-3989.

[1407] Paul M, Phillips J G, Renye J J. Short communication: Measuring the angiotensin-converting enzyme inhibitory activity of an 8-amino acid (8mer) fragment of the C12 antihypertensive peptide . J Dairy Sci, 2016,99(5):3263-3266.

[1408] Paul O. Background of the prevention of cardiovascular disease. II. Arteriosclerosis, hypertension, and selected risk factors . Circulation, 1989,80(1):206-214.

[1409] Pauly T H, Aziz S M, Horstman S J, et al. Impact of prostaglandin and thromboxane synthesis blockade on disposition of group B streptococcus in lung and liver of intact piglet . Pediatr Res, 1992,31(1):14-17.

[1410] Pauly T H, Bowdy B D, Haven C A, et al. Evidence for hydroxyl radical involvement in group B streptococcus-induced pulmonary hypertension and arterial hypoxemia in young piglets . Pediatr Res, 1988,24(6):735-739.

[1411] Pauly T H, Smith M, Gillespie M. Bilirubin as an antioxidant: effect on group B streptococci-induced pulmonary hypertension in infant piglets . Biol Neonate, 1991,60(5):320-326.

[1412] Payancé A, Rautou P E. Cirrhosis regression: extrahepatic angiogenesis and liver hyperarterialization persist . Clin Sci (Lond), 2018,132(12):1341-1343.

[1413] Pea F, Pavan F, Lugatti E, et al. Pharmacokinetic and pharmacodynamic aspects of oral moxifloxacin 400 mg/day in elderly patients with acute exacerbation of chronic bronchitis . Clin Pharmacokinet, 2006,45(3):287-295.

[1414] Pea F, Pavan F, Nascimben E, et al. Levofloxacin disposition in cerebrospinal fluid in patients with external ventriculostomy . Antimicrob Agents Chemother, 2003,47(10):3104-3108.

[1415] Pedersen B K, Bruunsgaard H, Ostrowski K, et al. Cytokines in aging and exercise . Int J Sports Med, 2000,21 Suppl 1:S4-S9.

[1416] Pedersen M, Brandt C T, Knudsen G M, et al. Cerebral blood flow autoregulation in early experimental S. pneumoniae meningitis . J Appl Physiol (1985), 2007,102(1):72-78.

[1417] Peevy K J, Chartrand S A, Wiseman H J, et al. Myocardial dysfunction in group B streptococcal shock . Pediatr Res, 1985,19(6):511-513.

[1418] Peevy K J, Panus P, Longenecker G L, et al. Prostaglandin synthetase inhibition in group B streptococcal shock: hematologic and hemodynamic effects . Pediatr Res, 1986,20(9):864-866.

[1419] Peh A, O'Donnell J A, Broughton B, et al. Gut Microbiota and Their Metabolites in Stroke: A Double-Edged Sword . Stroke, 2022:A121036800.

[1420] Pei R, Martin D A, DiMarco D M, et al. Evidence for the effects of yogurt on gut health and obesity . Crit Rev Food Sci Nutr, 2017,57(8):1569-1583.

[1421] Pekkala S, Munukka E, Kong L, et al. Toll-like receptor 5 in obesity: the role of gut microbiota and adipose tissue inflammation . Obesity (Silver Spring), 2015,23(3):581-590.

[1422] Peñas E, Diana M, Frias J, et al. A multistrategic approach in the development of sourdough bread targeted towards blood pressure reduction . Plant Foods Hum Nutr, 2015,70(1):97-103.

[1423] Peng J, Xiao X, Hu M, et al. Interaction between gut microbiome and cardiovascular disease . Life Sci, 2018,214:153-157.

[1424] Pereg D, Kotliroff A, Gadoth N, et al. Probiotics for patients with compensated liver cirrhosis: a double-blind placebo-controlled study . Nutrition, 2011,27(2):177-181.

[1425] Pereira Á, de Farias D, de Queiroz B B, et al. Influence of a Co-culture of Streptococcus thermophilus and Lactobacillus casei on the Proteolysis and ACE-Inhibitory Activity of a Beverage Based on Reconstituted Goat Whey Powder . Probiotics Antimicrob Proteins, 2019,11(1):273-282.

[1426] Perelló R, Miró O, Marcos M A, et al. Predicting bacteremic pneumonia in HIV-1-infected patients consulting the ED . Am J Emerg Med, 2010,28(4):454-459.

[1427] Pérez-Matute P, Íñiguez M, Villanueva-Millán M J, et al. Short-term effects of direct-acting antiviral agents on inflammation and gut microbiota in hepatitis C-infected patients . Eur J Intern Med, 2019,67:47-58.

[1428] Perlot T, Penninger J M. ACE2 - from the renin-angiotensin system to gut microbiota and malnutrition . Microbes Infect, 2013,15(13):866-873.

[1429] Peromet M, Labbe M, Yourassowsky E, et al. Anaerobic bacteria isolated from decubitus ulcers . Infection, 1973,1(4):205-207.

[1430] Persaud R R, Azad M B, Chari R S, et al. Perinatal antibiotic exposure of neonates in Canada and associated risk factors: a population-based study . J Matern Fetal Neonatal Med, 2015,28(10):1190-1195.

[1431] Persson R E, Persson G R, Kiyak H A, et al. Oral health and medical status in dentate low-income older persons . Spec Care Dentist, 1998,18(2):70-77.

[1432] Pessione E. Lactic acid bacteria contribution to gut microbiota complexity: lights and shadows . Front Cell Infect Microbiol, 2012,2:86.

[1433] Pessione E, Cirrincione S. Bioactive Molecules Released in Food by Lactic Acid Bacteria: Encrypted Peptides and Biogenic Amines . Front Microbiol, 2016,7:876.

[1434] Pestana-Oliveira N, Nahey D B, Hartson R, et al. DOCA-salt hypertension and the role of the OVLT-sympathetic-gut microbiome axis . Clin Exp Pharmacol Physiol, 2021,48(4):490-497.

[1435] Peters M J, Tuwairqi K W, Farah M G. A Case of Infected Left Atrial Myxoma Presenting as ST-Elevation Myocardial Infarction (STEMI) . Am J Case Rep, 2019,20:1930-1935.

[1436] Petersen A Ø, Julienne H, Hyötyläinen T, et al. Conjugated C-6 hydroxylated bile acids in serum relate to human metabolic health and gut Clostridia species . Sci Rep, 2021,11(1):13252.

[1437] Petyaev I M, Bashmakov Y K. Could cheese be the missing piece in the French paradox puzzle? . Med Hypotheses, 2012,79(6):746-749.

[1438] Pevsner-Fischer M, Blacher E, Tatirovsky E, et al. The gut microbiome and hypertension . Curr Opin Nephrol Hypertens, 2017,26(1):1-8.

[1439] Peyrol J, Riva C, Amiot M J. Hydroxytyrosol in the Prevention of the Metabolic Syndrome and Related Disorders . Nutrients, 2017,9(3).

[1440] Pfeiffer C M, Gregory J R. Enzymatic deconjugation of erythrocyte polyglutamyl folates during preparation for folate assay: investigation with reversed-phase liquid chromatography . Clin Chem, 1996,42(11):1847-1854.

[1441] Pfenninger J, Tschaeppeler H, Wagner B P, et al. The paradox of adult respiratory distress syndrome in neonates . Pediatr Pulmonol, 1991,10(1):18-24.

[1442] Pfeuffer M, Schrezenmeir J. Milk and the metabolic syndrome . Obes Rev, 2007,8(2):109-118.

[1443] Phelan M, Kerins D. The potential role of milk-derived peptides in cardiovascular disease . Food Funct, 2011,2(3-4):153-167.

[1444] Philips C A, Ahamed R, Rajesh S, et al. Update on diagnosis and management of sepsis in cirrhosis: Current advances . World J Hepatol, 2020,12(8):451-474.

[1445] Philips C A, Augustine P, Ganesan K, et al. The role of gut microbiota in clinical complications, disease severity, and treatment response in severe alcoholic hepatitis . Indian J Gastroenterol, 2022.

[1446] Philips C A, Augustine P, Padsalgi G, et al. Only in the darkness can you see the stars: Severe alcoholic hepatitis and higher grades of acute-on-chronic liver failure . J Hepatol, 2019,70(3):550-551.

[1447] Philips C A, Phadke N, Ganesan K, et al. Healthy donor faecal transplant for corticosteroid non-responsive severe alcoholic hepatitis . BMJ Case Rep, 2017,2017.

[1448] Philips C A, Rajesh S, Nair D C, et al. Hepatocellular Carcinoma in 2021: An Exhaustive Update . Cureus, 2021,13(11):e19274.

[1449] Philips J R, Li J X, Gray B M, et al. Role of capsule in pulmonary hypertension induced by group B streptococcus . Pediatr Res, 1992,31(4 Pt 1):386-390.

[1450] Philips J R, Lyrene R K, Godoy G, et al. Hemodynamic responses of chronically instrumented piglets to bolus injections of group B streptococci . Pediatr Res, 1988,23(1):81-85.

[1451] Pierce G L, Roy S J, Gimblet C J. The Gut-Arterial Stiffness Axis: Is TMAO a Novel Target to Prevent Age-Related Aortic Stiffening? . Hypertension, 2021,78(2):512-515.

[1452] Pietropaoli D, Del P R, Ferri C, et al. Definition of hypertension-associated oral pathogens in NHANES . J Periodontol, 2019,90(8):866-876.

[1453] Pignatelli P, Fabietti G, Ricci A, et al. How Periodontal Disease and Presence of Nitric Oxide Reducing Oral Bacteria Can Affect Blood Pressure . Int J Mol Sci, 2020,21(20).

[1454] Pijls K E, Jonkers D M, Elamin E E, et al. Intestinal epithelial barrier function in liver cirrhosis: an extensive review of the literature . Liver Int, 2013,33(10):1457-1469.

[1455] Pimenta F S, Luaces-Regueira M, Ton A M, et al. Mechanisms of Action of Kefir in Chronic Cardiovascular and Metabolic Diseases . Cell Physiol Biochem, 2018,48(5):1901-1914.

[1456] Piñero F, Vazquez M, Baré P, et al. A different gut microbiome linked to inflammation found in cirrhotic patients with and without hepatocellular carcinoma . Ann Hepatol, 2019,18(3):480-487.

[1457] Pinheiro J M, Pitt B R, Gillis C N. Roles of platelet-activating factor and thromboxane in group B Streptococcus-induced pulmonary hypertension in piglets . Pediatr Res, 1989,26(5):420-424.

[1458] Pinto S W, Mastroianni-Kirsztajn G, Sesso R. Ten-Year Follow-up of Patients with Epidemic Post Infectious Glomerulonephritis . PLoS One, 2015,10(5):e125313.

[1459] Pinto S W, Sesso R, Vasconcelos E, et al. Follow-up of patients with epidemic poststreptococcal glomerulonephritis . Am J Kidney Dis, 2001,38(2):249-255.

[1460] Pişkin N, Aydemir H, Oztoprak N, et al. [Factors effecting the duration of hospitalization and mortality in patients with community-acquired pneumonia] . Mikrobiyol Bul, 2009,43(4):597-606.

[1461] Pistollato F, Forbes-Hernandez T Y, Iglesias R C, et al. Effects of caloric restriction on immunosurveillance, microbiota and cancer cell phenotype: Possible implications for cancer treatment . Semin Cancer Biol, 2021,73:45-57.

[1462] Pistollato F, Iglesias R C, Ruiz R, et al. Nutritional patterns associated with the maintenance of neurocognitive functions and the risk of dementia and Alzheimer's disease: A focus on human studies . Pharmacol Res, 2018,131:32-43.

[1463] Pite H, Aguiar L, Morello J, et al. Metabolic Dysfunction and Asthma: Current Perspectives . J Asthma Allergy, 2020,13:237-247.

[1464] Piuri G, Zocchi M, Della P M, et al. Magnesium in Obesity, Metabolic Syndrome, and Type 2 Diabetes . Nutrients, 2021,13(2).

[1465] Plaza-Díaz J, Solis-Urra P, Aragón-Vela J, et al. Insights into the Impact of Microbiota in the Treatment of NAFLD/NASH and Its Potential as a Biomarker for Prognosis and Diagnosis . Biomedicines, 2021,9(2).

[1466] Pluznick J. A novel SCFA receptor, the microbiota, and blood pressure regulation . Gut Microbes, 2014,5(2):202-207.

[1467] Pluznick J L. Renal and cardiovascular sensory receptors and blood pressure regulation . Am J Physiol Renal Physiol, 2013,305(4):F439-F444.

[1468] Pluznick J L, Protzko R J, Gevorgyan H, et al. Olfactory receptor responding to gut microbiota-derived signals plays a role in renin secretion and blood pressure regulation . Proc Natl Acad Sci U S A, 2013,110(11):4410-4415.

[1469] Poca M A, Sahuquillo J, Arribas M, et al. Fiberoptic intraparenchymal brain pressure monitoring with the Camino V420 monitor: reflections on our experience in 163 severely head-injured patients . J Neurotrauma, 2002,19(4):439-448.

[1470] Polak P, Snopkova S, Husa P. [Polymicrobial brain abscess in hereditary haemorrhagic telangiectasia (Osler's disease)] . Dtsch Med Wochenschr, 2012,137(33):1635-1638.

[1471] Poll B G, Cheema M U, Pluznick J L. Gut Microbial Metabolites and Blood Pressure Regulation: Focus on SCFAs and TMAO . Physiology (Bethesda), 2020,35(4):275-284.

[1472] Poll B G, Xu J, Jun S, et al. Acetate, a Short-Chain Fatty Acid, Acutely Lowers Heart Rate and Cardiac Contractility Along with Blood Pressure . J Pharmacol Exp Ther, 2021,377(1):39-50.

[1473] Polla D, Astafurov K, Hawy E, et al. A Pilot Study to Evaluate the Oral Microbiome and Dental Health in Primary Open-Angle Glaucoma . J Glaucoma, 2017,26(4):320-327.

[1474] Polverino E, Rosales-Mayor E, Benegas M, et al. Pneumonic and non-pneumonic exacerbations in bronchiectasis: Clinical and microbiological differences . J Infect, 2018,77(2):99-106.

[1475] Ponziani F R, Gerardi V, Pecere S, et al. Effect of rifaximin on gut microbiota composition in advanced liver disease and its complications . World J Gastroenterol, 2015,21(43):12322-12333.

[1476] Ponziani F R, Zocco M A, Cerrito L, et al. Bacterial translocation in patients with liver cirrhosis: physiology, clinical consequences, and practical implications . Expert Rev Gastroenterol Hepatol, 2018,12(7):641-656.

[1477] Poon-King T, Svartman M, Mohammed I, et al. Epidemic acute nephritis with reappearance of M-type 55 streptococci in Trinidad . Lancet, 1973,1(7801):475-479.

[1478] Pornrattanarungsi S, Eursiriwan S, Amornchaicharoensuk Y, et al. Concomitant rapidly progressive glomerulonephritis and acute rheumatic fever after streptococcus infection: a case report . Paediatr Int Child Health, 2022:1-5.

[1479] Pose E, Napoleone L, Amin A, et al. Safety of two different doses of simvastatin plus rifaximin in decompensated cirrhosis (LIVERHOPE-SAFETY): a randomised, double-blind, placebo-controlled, phase 2 trial . Lancet Gastroenterol Hepatol, 2020,5(1):31-41.

[1480] Potì F, Santi D, Spaggiari G, et al. Polyphenol Health Effects on Cardiovascular and Neurodegenerative Disorders: A Review and Meta-Analysis . Int J Mol Sci, 2019,20(2).

[1481] Praagman J, Dalmeijer G W, van der Schouw Y T, et al. The relationship between fermented food intake and mortality risk in the European Prospective Investigation into Cancer and Nutrition-Netherlands cohort . Br J Nutr, 2015,113(3):498-506.

[1482] Prados-Bo A, Gómez-Martínez S, Nova E, et al. [Role of probiotics in obesity management] . Nutr Hosp, 2015,31 Suppl 1:10-18.

[1483] Prager O, Friedman A, Nebenzahl Y M. Role of neural barriers in the pathogenesis and outcome of Streptococcus pneumoniae meningitis . Exp Ther Med, 2017,13(3):799-809.

[1484] Prakash A, Raj R, Jacob A, et al. Pressure Ulcer Associated with Testicular Prosthesis as a Rare Cause of Spinal Epidural Abscess . Case Rep Infect Dis, 2019,2019:9090462.

[1485] Prakash S, Urbanska A M. Colon-targeted delivery of live bacterial cell biotherapeutics including microencapsulated live bacterial cells . Biologics, 2008,2(3):355-378.

[1486] Prat H, Araos P, Michea L. [Role of inflammation in hypertension] . Rev Med Chil, 2021,149(2):255-262.

[1487] Prentice A M. Dairy products in global public health . Am J Clin Nutr, 2014,99(5 Suppl):1212S-1216S.

[1488] Pribble C G, Shaddy R E. Intra-aortic balloon counterpulsation in newborn lambs infected with group B streptococcus . ASAIO Trans, 1991,37(1):33-37.

[1489] Prieto I, Hidalgo M, Segarra A B, et al. Influence of a diet enriched with virgin olive oil or butter on mouse gut microbiota and its correlation to physiological and biochemical parameters related to metabolic syndrome . PLoS One, 2018,13(1):e190368.

[1490] Prisco S Z, Eklund M, Moutsoglou D M, et al. Intermittent Fasting Enhances Right Ventricular Function in Preclinical Pulmonary Arterial Hypertension . J Am Heart Assoc, 2021,10(22):e22722.

[1491] Pruyn S C. Acute necrotizing fasciitis of the endopelvic fascia . Obstet Gynecol, 1978,52(1 Suppl):25-45.

[1492] Puupponen-Pimiä R, Seppänen-Laakso T, Kankainen M, et al. Effects of ellagitannin-rich berries on blood lipids, gut microbiota, and urolithin production in human subjects with symptoms of metabolic syndrome . Mol Nutr Food Res, 2013,57(12):2258-2263.

[1493] Qamar A A. Probiotics in Nonalcoholic Fatty Liver Disease, Nonalcoholic Steatohepatitis, and Cirrhosis . J Clin Gastroenterol, 2015,49 Suppl 1:S28-S32.

[1494] Qi D, Nie X L, Zhang J J. The effect of probiotics supplementation on blood pressure: a systemic review and meta-analysis . Lipids Health Dis, 2020,19(1):79.

[1495] Qi Y Z, Yang X S, Jiang Y H, et al. Study of the Mechanism Underlying the Antihypertensive Effects of Eucommia ulmoides and Tribulus terrestris Based on an Analysis of the Intestinal Microbiota and Metabonomics . Biomed Res Int, 2020,2020:4261485.

[1496] Qi Y, Aranda J M, Rodriguez V, et al. Impact of antibiotics on arterial blood pressure in a patient with resistant hypertension - A case report . Int J Cardiol, 2015,201:157-158.

[1497] Qi Y, Kim S, Richards E M, et al. Gut Microbiota: Potential for a Unifying Hypothesis for Prevention and Treatment of Hypertension . Circ Res, 2017,120(11):1724-1726.

[1498] Qian Y, Ge Q, Zuo W, et al. Maxillofacial space infection experience and risk factors: a retrospective study of 222 cases . Ir J Med Sci, 2021,190(3):1045-1053.

[1499] Qin Q, Yan S, Yang Y, et al. A Metagenome-Wide Association Study of the Gut Microbiome and Metabolic Syndrome . Front Microbiol, 2021,12:682721.

[1500] Quast M B, Carr C M, Hooten W M. Multilevel lumbar spine infection due to poor dentition in an immunocompetent adult: a case report . J Med Case Rep, 2017,11(1):328.

[1501] Queipo-Ortuño M I, Boto-Ordóñez M, Murri M, et al. Influence of red wine polyphenols and ethanol on the gut microbiota ecology and biochemical biomarkers . Am J Clin Nutr, 2012,95(6):1323-1334.

[1502] Quessy S, Dubreuil J D, Jacques M, et al. Increase of capsular material thickness following in vivo growth of virulent Streptococcus suis serotype 2 strains . FEMS Microbiol Lett, 1994,115(1):19-26.

[1503] R M R, Marques F Z. Diet-related gut microbial metabolites and sensing in hypertension . J Hum Hypertens, 2021,35(2):162-169.

[1504] Rabelo N N, Teixeira M J, Figueiredo E G. Letter by Rabelo et al Regarding Article "Potential Influences of Gut Microbiota on the Formation of Intracranial Aneurysm" . Hypertension, 2019,74(1):e1.

[1505] Rabiei S, Shakerhosseini R, Saadat N. The effects of symbiotic therapy on anthropometric measures, body composition and blood pressure in patient with metabolic syndrome: a triple blind RCT . Med J Islam Repub Iran, 2015,29:213.

[1506] Radwan S, Gilfillan D, Eklund B, et al. A comparative study of the gut microbiome in Egyptian patients with Type I and Type II diabetes . PLoS One, 2020,15(9):e238764.

[1507] Rahman A, Abou-Foul A K, Yusaf A, et al. Necrotising myositis, the deadly impersonator . Case Rep Surg, 2014,2014:485651.

[1508] Rai A K, Sanjukta S, Jeyaram K. Production of angiotensin I converting enzyme inhibitory (ACE-I) peptides during milk fermentation and their role in reducing hypertension . Crit Rev Food Sci Nutr, 2017,57(13):2789-2800.

[1509] Raizada M K, Joe B, Bryan N S, et al. Report of the National Heart, Lung, and Blood Institute Working Group on the Role of Microbiota in Blood Pressure Regulation: Current Status and Future Directions . Hypertension, 2017.

[1510] Raj D S, Sohn M B, Charytan D M, et al. The Microbiome and p-Inulin in Hemodialysis: A Feasibility Study . Kidney360, 2021,2(3):445-455.

[1511] Ramanathan G, Abeyaratne A, Sundaram M, et al. Analysis of clinical presentation, pathological spectra, treatment and outcomes of biopsy-proven acute postinfectious glomerulonephritis in adult indigenous people of the Northern Territory of Australia . Nephrology (Carlton), 2017,22(5):403-411.

[1512] Ramdath D D, Padhi E M, Sarfaraz S, et al. Beyond the Cholesterol-Lowering Effect of Soy Protein: A Review of the Effects of Dietary Soy and Its Constituents on Risk Factors for Cardiovascular Disease . Nutrients, 2017,9(4).

[1513] Ramineni S, Bandi V K. Clinicopathological profile and outcomes of infection-related glomerulonephritis in adults . Clin Nephrol, 2021,95(2):93-98.

[1514] Ramírez J H, Parra B, Gutierrez S, et al. Biomarkers of cardiovascular disease are increased in untreated chronic periodontitis: a case control study . Aust Dent J, 2014,59(1):29-36.

[1515] Rammos C, Hendgen-Cotta U B, Totzeck M, et al. Impact of dietary nitrate on age-related diastolic dysfunction . Eur J Heart Fail, 2016,18(6):599-610.

[1516] Randhawa E, Woytanowski J, Sibliss K, et al. Streptococcus pyogenes and invasive central nervous system infection . SAGE Open Med Case Rep, 2018,6:2050313X-18775584X.

[1517] Randi B A, Ninomiya D A, Nicodemo E L, et al. Recurrent bacteremia after injection of N-butyl-2-cyanoacrylate for treatment of bleeding gastric varices: a case report and review of the literature . BMC Res Notes, 2015,8:692.

[1518] Rao B K, Kumar P, Rao S, et al. Bactericidal effect of ultraviolet C (UVC), direct and filtered through transparent plastic, on gram-positive cocci: an in vitro study . Ostomy Wound Manage, 2011,57(7):46-52.

[1519] Rashidi A, Ebadi M, Rehman T U, et al. Lasting shift in the gut microbiota in patients with acute myeloid leukemia . Blood Adv, 2022.

[1520] Rastad H, Ejtahed H S, Mahdavi-Ghorabi A, et al. Factors associated with the poor outcomes in diabetic patients with COVID-19 . J Diabetes Metab Disord, 2020,19(2):1293-1302.

[1521] Ravn-Haren G, Dragsted L O, Buch-Andersen T, et al. Intake of whole apples or clear apple juice has contrasting effects on plasma lipids in healthy volunteers . Eur J Nutr, 2013,52(8):1875-1889.

[1522] Razavi A C, Potts K S, Kelly T N, et al. Sex, gut microbiome, and cardiovascular disease risk . Biol Sex Differ, 2019,10(1):29.

[1523] Razmpoosh E, Zare S, Fallahzadeh H, et al. Effect of a low energy diet, containing a high protein, probiotic condensed yogurt, on biochemical and anthropometric measurements among women with overweight/obesity: A randomised controlled trial . Clin Nutr ESPEN, 2020,35:194-200.

[1524] Redding G J, Gibson R L, Standaert T A, et al. Regional pulmonary blood flow in piglets during group B streptococcal bacteremia . Am Rev Respir Dis, 1990,141(5 Pt 1):1209-1213.

[1525] Reddy B R. Noncaloric Benefits of Carbohydrates . Nestle Nutr Inst Workshop Ser, 2015,82:27-37.

[1526] Reho J J, Nakagawa P, Mouradian G J, et al. Methods for the Comprehensive in vivo Analysis of Energy Flux, Fluid Homeostasis, Blood Pressure, and Ventilatory Function in Rodents . Front Physiol, 2022,13:855054.

[1527] Reimer R A, Wharton S, Green T J, et al. Effect of a functional fibre supplement on glycemic control when added to a year-long medically supervised weight management program in adults with type 2 diabetes . Eur J Nutr, 2021,60(3):1237-1251.

[1528] Reinhardt C, Reigstad C S, Bäckhed F. Intestinal microbiota during infancy and its implications for obesity . J Pediatr Gastroenterol Nutr, 2009,48(3):249-256.

[1529] Reintam B A, Malbrain M, Regli A. Abdominal pressure and gastrointestinal function: an inseparable couple? . Anaesthesiol Intensive Ther, 2017,49(2):146-158.

[1530] Reintam B A, Preiser J C, Fruhwald S, et al. Gastrointestinal dysfunction in the critically ill: a systematic scoping review and research agenda proposed by the Section of Metabolism, Endocrinology and Nutrition of the European Society of Intensive Care Medicine . Crit Care, 2020,24(1):224.

[1531] Rhys-Jones D, Climie R E, Gill P A, et al. Microbial Interventions to Control and Reduce Blood Pressure in Australia (MICRoBIA): rationale and design of a double-blinded randomised cross-over placebo controlled trial . Trials, 2021,22(1):496.

[1532] Riachy M A. Streptococcus pneumoniae causing septic arthritis with shock and revealing multiple myeloma . BMJ Case Rep, 2011,2011.

[1533] Ribeiro A G, Mill J G, Cade N V, et al. Associations of Dairy Intake with Arterial Stiffness in Brazilian Adults: The Brazilian Longitudinal Study of Adult Health (ELSA-Brasil) . Nutrients, 2018,10(6).

[1534] Rice D R, Plaunt A J, Turkyilmaz S, et al. Evaluation of [¹¹¹In]-labeled zinc-dipicolylamine tracers for SPECT imaging of bacterial infection . Mol Imaging Biol, 2015,17(2):204-213.

[1535] Richards E M, Li J, Stevens B R, et al. Gut Microbiome and Neuroinflammation in Hypertension . Circ Res, 2022,130(3):401-417.

[1536] Richards E M, Pepine C J, Raizada M K, et al. The Gut, Its Microbiome, and Hypertension . Curr Hypertens Rep, 2017,19(4):36.

[1537] Richter C K, Skulas-Ray A C, Champagne C M, et al. Plant protein and animal proteins: do they differentially affect cardiovascular disease risk? . Adv Nutr, 2015,6(6):712-728.

[1538] Ried K. Garlic lowers blood pressure in hypertensive subjects, improves arterial stiffness and gut microbiota: A review and meta-analysis . Exp Ther Med, 2020,19(2):1472-1478.

[1539] Ried K, Travica N, Sali A. The Effect of Kyolic Aged Garlic Extract on Gut Microbiota, Inflammation, and Cardiovascular Markers in Hypertensives: The GarGIC Trial . Front Nutr, 2018,5:122.

[1540] Riedl R A, Atkinson S N, Burnett C, et al. The Gut Microbiome, Energy Homeostasis, and Implications for Hypertension . Curr Hypertens Rep, 2017,19(4):27.

[1541] Rincón D, Vaquero J, Hernando A, et al. Oral probiotic VSL#3 attenuates the circulatory disturbances of patients with cirrhosis and ascites . Liver Int, 2014,34(10):1504-1512.

[1542] Rinella M E, Tacke F, Sanyal A J, et al. Report on the AASLD/EASL joint workshop on clinical trial endpoints in NAFLD . J Hepatol, 2019,71(4):823-833.

[1543] Ring A, Braun J S, Pohl J, et al. Group B streptococcal beta-hemolysin induces mortality and liver injury in experimental sepsis . J Infect Dis, 2002,185(12):1745-1753.

[1544] Rinott E, Youngster I, Yaskolka M A, et al. Effects of Diet-Modulated Autologous Fecal Microbiota Transplantation on Weight Regain . Gastroenterology, 2021,160(1):158-173.

[1545] Rizzello C G, De Angelis M, Di Cagno R, et al. Highly efficient gluten degradation by lactobacilli and fungal proteases during food processing: new perspectives for celiac disease . Appl Environ Microbiol, 2007,73(14):4499-4507.

[1546] Roberton N R. Management of hyaline membrane disease . Arch Dis Child, 1979,54(11):838-844.

[1547] Robinson C A, Kellar J Z, Stehr R C. An 84-Year-Old Man with Acute Atraumatic Compartment Syndrome of the Upper Extremity Due to Streptococcus pyogenes Cellulitis . Am J Case Rep, 2021,22:e929176.

[1548] Robles-Vera I, de la Visitación N, Toral M, et al. Probiotic Bifidobacterium breve prevents DOCA-salt hypertension . FASEB J, 2020,34(10):13626-13640.

[1549] Robles-Vera I, Toral M, de la Visitación N, et al. Changes to the gut microbiota induced by losartan contributes to its antihypertensive effects . Br J Pharmacol, 2020,177(9):2006-2023.

[1550] Robles-Vera I, Toral M, Duarte J. Microbiota and Hypertension: Role of the Sympathetic Nervous System and the Immune System . Am J Hypertens, 2020,33(10):890-901.

[1551] Robles-Vera I, Toral M, Romero M, et al. Antihypertensive Effects of Probiotics . Curr Hypertens Rep, 2017,19(4):26.

[1552] Rodríguez-Figueroa J C, González-Córdova A F, Astiazaran-García H, et al. Antihypertensive and hypolipidemic effect of milk fermented by specific Lactococcus lactis strains . J Dairy Sci, 2013,96(7):4094-4099.

[1553] Rodríguez-González G L, Castro-Rodríguez D C, Zambrano E. Pregnancy and Lactation: A Window of Opportunity to Improve Individual Health . Methods Mol Biol, 2018,1735:115-144.

[1554] Rodríguez-Muñoz L, García-Galván Ó, González-Soto M Á, et al. Toxic shock syndrome caused by Streptococcus dysgalactiae subsp. equisimilis in a Mexican preschool patient . Bol Med Hosp Infant Mex, 2019,76(5):237-240.

[1555] Rodríguez-Vera D, Vergara-Castañeda A, Lazcano-Orozco D K, et al. Inflammation Parameters Associated with Metabolic Disorders: Relationship Between Diet and Microbiota . Metab Syndr Relat Disord, 2021,19(9):469-482.

[1556] Rogers G B, van der Gast C J, Bruce K D, et al. Ascitic microbiota composition is correlated with clinical severity in cirrhosis with portal hypertension . PLoS One, 2013,8(9):e74884.

[1557] Rojas J, Green R S, Hellerqvist C G, et al. Studies on group B beta-hemolytic Streptococcus. II. Effects on pulmonary hemodynamics and vascular permeability in unanesthetized sheep . Pediatr Res, 1981,15(6):899-904.

[1558] Rojas J, Larsson L E, Hellerqvist C G, et al. Pulmonary hemodynamic and ultrastructural changes associated with Group B streptococcal toxemia in adult sheep and newborn lambs . Pediatr Res, 1983,17(12):1002-1008.

[1559] Rojas J, Larsson L E, Ogletree M L, et al. Effects of cyclooxygenase inhibition on the response to group B streptococcal toxin in sheep . Pediatr Res, 1983,17(2):107-110.

[1560] Rojas J, Palme C, Ogletree M L, et al. Effects of methylprednisolone on the response to group B streptococcal toxin in sheep . Pediatr Res, 1984,18(11):1141-1144.

[1561] Rosa D D, Dias M, Grześkowiak Ł M, et al. Milk kefir: nutritional, microbiological and health benefits . Nutr Res Rev, 2017,30(1):82-96.

[1562] Rosa D D, Grześkowiak Ł M, Ferreira C L, et al. Kefir reduces insulin resistance and inflammatory cytokine expression in an animal model of metabolic syndrome . Food Funct, 2016,7(8):3390-3401.

[1563] Rose A V, Boreskie K F, Hay J L, et al. Protocol for the WARM Hearts study: examining cardiovascular disease risk in middle-aged and older women - a prospective, observational cohort study . BMJ Open, 2021,11(5):e44227.

[1564] Rosier B T, Moya-Gonzalvez E M, Corell-Escuin P, et al. Isolation and Characterization of Nitrate-Reducing Bacteria as Potential Probiotics for Oral and Systemic Health . Front Microbiol, 2020,11:555465.

[1565] Rosier B T, Takahashi N, Zaura E, et al. The Importance of Nitrate Reduction for Oral Health . J Dent Res, 2022:513894294.

[1566] Ross A B, Bruce S J, Blondel-Lubrano A, et al. A whole-grain cereal-rich diet increases plasma betaine, and tends to decrease total and LDL-cholesterol compared with a refined-grain diet in healthy subjects . Br J Nutr, 2011,105(10):1492-1502.

[1567] Rostagno C, Carone E, Stefàno P L. Role of mitral valve repair in active infective endocarditis: long term results . J Cardiothorac Surg, 2017,12(1):29.

[1568] Rotz S J, Sangwan N, Nagy M, et al. Fecal microbiota of adolescent and young adult cancer survivors and metabolic syndrome: an exploratory study . Pediatr Hematol Oncol, 2022:1-15.

[1569] Ruan Z, Li J, Liu F, et al. Study design, general characteristics of participants, and preliminary findings from the metabolome, microbiome, and dietary salt intervention study (MetaSalt) . Chronic Dis Transl Med, 2021,7(4):227-234.

[1570] Rubens C E, Raff H V, Jackson J C, et al. Pathophysiology and histopathology of group B streptococcal sepsis in Macaca nemestrina primates induced after intraamniotic inoculation: evidence for bacterial cellular invasion . J Infect Dis, 1991,164(2):320-330.

[1571] Rubio V Y, Cagmat J G, Wang G P, et al. Analysis of Tryptophan Metabolites in Serum Using Wide-Isolation Strategies for UHPLC-HRMS/MS . Anal Chem, 2020,92(3):2550-2557.

[1572] Rudinsky B F, Komar K J, Strates E, et al. Neither nitroglycerin nor nitroprusside selectively reduces sepsis-induced pulmonary hypertension in piglets . Crit Care Med, 1987,15(12):1127-1130.

[1573] Rudinsky B F, Lozon M, Bell A, et al. Group B streptococcal sepsis impairs cerebral vascular reactivity to acute hypercarbia in piglets . Pediatr Res, 1996,39(1):55-63.

[1574] Rudinsky B, Meadow W. Hemodynamic effects of combining epinephrine with nitroglycerin or nitroprusside during group B streptococcal sepsis in piglets . Am J Perinatol, 1992,9(5-6):435-440.

[1575] Rui X, Wen D, Li W, et al. Enrichment of ACE inhibitory peptides in navy bean (Phaseolus vulgaris) using lactic acid bacteria . Food Funct, 2015,6(2):622-629.

[1576] Rumbold A R, Bailie R S, Si D, et al. Delivery of maternal health care in Indigenous primary care services: baseline data for an ongoing quality improvement initiative . BMC Pregnancy Childbirth, 2011,11:16.

[1577] Runkle B, Goldberg R N, Streitfeld M M, et al. Cardiovascular changes in group B streptococcal sepsis in the piglet: response to indomethacin and relationship to prostacyclin and thromboxane A2 . Pediatr Res, 1984,18(9):874-878.

[1578] Ruscica M, Corsini A, Ferri N, et al. Clinical approach to the inflammatory etiology of cardiovascular diseases . Pharmacol Res, 2020,159:104916.

[1579] Russel S M, Valle V, Spagni G, et al. Physiologic Mechanisms of Type II Diabetes Mellitus Remission Following Bariatric Surgery: a Meta-analysis and Clinical Implications . J Gastrointest Surg, 2020,24(3):728-741.

[1580] Rustia A J, Paterson J S, Best G, et al. Microbial disruption in the gut promotes cerebral endothelial dysfunction . Physiol Rep, 2021,9(21):e15100.

[1581] Ryou M, Stylopoulos N, Baffy G. Nonalcoholic fatty liver disease and portal hypertension . Explor Med, 2020,1:149-169.

[1582] Rzaska M, Niewiadomski S, Karwacki Z. Molecular mechanisms of bacterial infections of the central nervous system . Anaesthesiol Intensive Ther, 2017,49(5):387-392.

[1583] Saade G R. Human immunodeficiency virus (HIV)-related pulmonary complications in pregnancy . Semin Perinatol, 1997,21(4):336-350.

[1584] Sadeghzadeh J, Vakili A, Sameni H R, et al. The Effect of Oral Consumption of Probiotics in Prevention of Heart Injury in a Rat Myocardial Infarction Model: a Histopathological, Hemodynamic and Biochemical Evaluation . Iran Biomed J, 2017,21(3):174-181.

[1585] Sadek A A, Mohamad M A, Ali S H, et al. Diagnostic value of lumbar puncture among infants and children presenting with fever and convulsions . Electron Physician, 2016,8(4):2255-2262.

[1586] Saetre T, Höiby E A, Aspelin T, et al. Aminoethyl-isothiourea inhibits the increase in plasma endothelin-1 caused by serogroup A streptococci and prolongs survival in rat peritoneal sepsis . Shock, 2001,15(6):446-452.

[1587] Saetre T, Hoiby E A, Aspelin T, et al. Acute serogroup A streptococcal shock: A porcine model . J Infect Dis, 2000,182(1):133-141.

[1588] Saetre T, Lindgaard A K, Lyberg T. Systemic activation of coagulation and fibrynolysis in a porcine model of serogroup A streptococcal shock . Blood Coagul Fibrinolysis, 2000,11(5):433-438.

[1589] Safadieh L, Sharara-Chami R, Dabbagh O. Paroxysmal autonomic instability with dystonia after pneumococcal meningoencephalitis . Case Rep Med, 2012,2012:965932.

[1590] Safari O, Ejtahed H S, Namazi N, et al. Association of short stature and obesity with cardio-metabolic risk factors in Iranian children and adolescents: the CASPIAN-V study . J Diabetes Metab Disord, 2021,20(2):1137-1144.

[1591] Sahebkar A, Serban M C, Gluba-Brzózka A, et al. Lipid-modifying effects of nutraceuticals: An evidence-based approach . Nutrition, 2016,32(11-12):1179-1192.

[1592] Sahin I, Acar S, Ozaydın I, et al. [Investigation of the effects of probiotic bacteria on bacterial translocation that developed during diagnostic laparoscopy: an experimental study] . Mikrobiyol Bul, 2012,46(4):660-670.

[1593] Said S A. Characteristics of Congenital Coronary Artery Fistulas Complicated with Infective Endocarditis: Analysis of 25 Reported Cases . Congenit Heart Dis, 2016,11(6):756-765.

[1594] Sajdel-Sulkowska E M. A Dual-Route Perspective of SARS-CoV-2 Infection: Lung- vs. Gut-specific Effects of ACE-2 Deficiency . Front Pharmacol, 2021,12:684610.

[1595] Saji N, Murotani K, Sato N, et al. Relationship Between Plasma Neurofilament Light Chain, Gut Microbiota, and Dementia: A Cross-Sectional Study . J Alzheimers Dis, 2022.

[1596] Saka N, Seo T, Kashiba K, et al. [Case of toxic shock-like syndrome affecting the neck] . Nihon Jibiinkoka Gakkai Kaiho, 2006,109(9):703-706.

[1597] Sakurai Y, Kubota N, Yamauchi T, et al. Role of Insulin Resistance in MAFLD . Int J Mol Sci, 2021,22(8).

[1598] Saldías F, Mardónez J M, Marchesse M, et al. [Community-acquired pneumonia in hospitalized adult patients. Clinical presentation and prognostic factors] . Rev Med Chil, 2002,130(12):1373-1382.

[1599] Saldías P F, Reyes B T, Sáez B J, et al. [Clinical predictors of bacteremia in immunocompetent adult patients hospitalized for community-acquired pneumonia] . Rev Med Chil, 2015,143(5):553-561.

[1600] Saldías P F, Viviani G P, Pulgar B D, et al. [Prognostic factors and mortality in immunocompetent adult patients hospitalized with community-acquired pneumococcal pneumonia] . Rev Med Chil, 2009,137(12):1545-1552.

[1601] Saldías P F, O'Brien S A, Gederlini G A, et al. [Community-acquired pneumonia requiring hospitalization in immunocompetent elderly patients: clinical features, prognostic factors and treatment] . Arch Bronconeumol, 2003,39(8):333-340.

[1602] Sallé G, Canlet C, Cortet J, et al. Integrative biology defines novel biomarkers of resistance to strongylid infection in horses . Sci Rep, 2021,11(1):14278.

[1603] Salmenkari H, Korpela R, Vapaatalo H. Renin-angiotensin system in intestinal inflammation-Angiotensin inhibitors to treat inflammatory bowel diseases? . Basic Clin Pharmacol Toxicol, 2021,129(3):161-172.

[1604] Salvado R, Santos-Minguez S, Agudo-Conde C, et al. Gut microbiota composition and arterial stiffness measured by pulse wave velocity: case-control study protocol (MIVAS study) . BMJ Open, 2021,11(2):e38933.

[1605] Samakoses R, Suwanpakdee D, Watanaveeradej V, et al. Cerebrospinal fluid lymphocytosis in an infant with acute Streptococcus pnuemoniae meningitis: a case report . J Med Assoc Thai, 2010,93 Suppl 5:S49-S52.

[1606] Samanta A, Patra A, Mandal S, et al. Hypoxia: A cause of acute renal failure and alteration of gastrointestinal microbial ecology . Saudi J Kidney Dis Transpl, 2018,29(4):879-888.

[1607] Samaroo-Campbell J, Hashmi A, Thawani R, et al. Isolated Pulmonic Valve Endocarditis . Am J Case Rep, 2019,20:151-153.

[1608] Sanada T J, Hosomi K, Shoji H, et al. Gut microbiota modification suppresses the development of pulmonary arterial hypertension in an SU5416/hypoxia rat model . Pulm Circ, 2020,10(3):765618565.

[1609] Sánchez-Miralles A, Castellanos G, Badenes R, et al. [Abdominal compartment syndrome and acute intestinal distress syndrome] . Med Intensiva, 2013,37(2):99-109.

[1610] Sanchez-Niño M D, Aguilera-Correa J J, Politei J, et al. Unraveling the drivers and consequences of gut microbiota disruption in Fabry disease: the lyso-Gb3 link . Future Microbiol, 2020,15:227-231.

[1611] Sanchez-Rodriguez E, Egea-Zorrilla A, Plaza-Díaz J, et al. The Gut Microbiota and Its Implication in the Development of Atherosclerosis and Related Cardiovascular Diseases . Nutrients, 2020,12(3).

[1612] Sandberg K, Engelhardt B, Hellerqvist C, et al. Pulmonary response to group B streptococcal toxin in young lambs . J Appl Physiol (1985), 1987,63(5):2024-2030.

[1613] Sanders M E. Considerations for use of probiotic bacteria to modulate human health . J Nutr, 2000,130(2S Suppl):384S-390S.

[1614] Sanduzzi A, Canora A, Belfiore P, et al. Impact of 13Valent Vaccine for Prevention of Pneumococcal Diseases in Children and Adults at Risk: Possible Scenarios in Campania Region . Infect Disord Drug Targets, 2019,19(4):403-408.

[1615] Şanlier N, Gökcen B B, Sezgin A C. Health benefits of fermented foods . Crit Rev Food Sci Nutr, 2019,59(3):506-527.

[1616] Santisteban M M, Kim S, Pepine C J, et al. Brain-Gut-Bone Marrow Axis: Implications for Hypertension and Related Therapeutics . Circ Res, 2016,118(8):1327-1336.

[1617] Santisteban M M, Qi Y, Zubcevic J, et al. Hypertension-Linked Pathophysiological Alterations in the Gut . Circ Res, 2017,120(2):312-323.

[1618] Sanz F, Restrepo M I, Fernández-Fabrellas E, et al. Does prolonged onset of symptoms have a prognostic significance in community-acquired pneumonia? . Respirology, 2014,19(7):1073-1079.

[1619] Saputri F A, Kang D, Kusuma A, et al. Lactobacillus plantarum IS-10506 probiotic administration increases amlodipine absorption in a rabbit model . J Int Med Res, 2018,46(12):5004-5010.

[1620] Sarafian M H, Lewis M R, Pechlivanis A, et al. Bile acid profiling and quantification in biofluids using ultra-performance liquid chromatography tandem mass spectrometry . Anal Chem, 2015,87(19):9662-9670.

[1621] Saraya T, Nunokawa H, Ohkuma K, et al. A Novel Diagnostic Scoring System to Differentiate between Legionella pneumophila Pneumonia and Streptococcus pneumoniae Pneumonia . Intern Med, 2018,57(17):2479-2487.

[1622] Sasahara J, Kikuchi A, Takakuwa K, et al. Antibody responses to Porphyromonas gingivalis outer membrane protein in the first trimester . Aust N Z J Obstet Gynaecol, 2009,49(2):137-141.

[1623] Sata Y, Marques F Z, Kaye D M. The Emerging Role of Gut Dysbiosis in Cardio-metabolic Risk Factors for Heart Failure . Curr Hypertens Rep, 2020,22(5):38.

[1624] Satlin M J, Westblade L F, Lee J R. Avoiding infections in transplant recipients: does the gut microbiota have a key role? . Expert Rev Clin Immunol, 2020,16(2):113-115.

[1625] Savard P, Lamarche B, Paradis M E, et al. Impact of Bifidobacterium animalis subsp. lactis BB-12 and, Lactobacillus acidophilus LA-5-containing yoghurt, on fecal bacterial counts of healthy adults . Int J Food Microbiol, 2011,149(1):50-57.

[1626] Savignac H M, Corona G, Mills H, et al. Prebiotic feeding elevates central brain derived neurotrophic factor, N-methyl-D-aspartate receptor subunits and D-serine . Neurochem Int, 2013,63(8):756-764.

[1627] Sawada H, Furushiro M, Hirai K, et al. Purification and characterization of an antihypertensive compound from Lactobacillus casei . Agric Biol Chem, 1990,54(12):3211-3219.

[1628] Sawicka B, Skiba D, Pszczółkowski P, et al. Jerusalem artichoke (Helianthus tuberosus L.) as a medicinal plant and its natural products . Cell Mol Biol (Noisy-le-grand), 2020,66(4):160-177.

[1629] Scaioli E, Colecchia A, Marasco G, et al. Pathophysiology and Therapeutic Strategies for Symptomatic Uncomplicated Diverticular Disease of the Colon . Dig Dis Sci, 2016,61(3):673-683.

[1630] Schacht R G, Gluck M C, Gallo G R, et al. Progression to uremia after remission of acute poststreptococcal glomerulonephritis . N Engl J Med, 1976,295(18):977-981.

[1631] Schadewaldt P, Hummel W, Trautvetter U, et al. A convenient enzymatic method for the determination of 4-methyl-2-oxopentanoate in plasma: comparison with high performance liquid chromatographic analysis . Clin Chim Acta, 1989,183(2):171-182.

[1632] Schär M Y, Corona G, Soycan G, et al. Excretion of Avenanthramides, Phenolic Acids and their Major Metabolites Following Intake of Oat Bran . Mol Nutr Food Res, 2018,62(2).

[1633] Scheiring J, Rosales A, Zimmerhackl L B. Clinical practice. Today's understanding of the haemolytic uraemic syndrome . Eur J Pediatr, 2010,169(1):7-13.

[1634] Schiattarella G G, Sannino A, Esposito G, et al. Diagnostics and therapeutic implications of gut microbiota alterations in cardiometabolic diseases . Trends Cardiovasc Med, 2019,29(3):141-147.

[1635] Schierwagen R, Alvarez-Silva C, Madsen M, et al. Circulating microbiome in blood of different circulatory compartments . Gut, 2019,68(3):578-580.

[1636] Schiffrin E L. Compendium on Hypertension: The Microbiome, Inflammation and Oxidative Stress, the Sympathetic Nervous System and Treatment of Hypertension . Am J Hypertens, 2020,33(10):889.

[1637] Schiffrin E L. Hypertension in 2017: Novel mechanisms of hypertension and vascular dysfunction . Nat Rev Nephrol, 2018,14(2):73-74.

[1638] Schirmer M, Kumar V, Netea M G, et al. The causes and consequences of variation in human cytokine production in health . Curr Opin Immunol, 2018,54:50-58.

[1639] Schlienger J L, Paillard F, Lecerf J M, et al. Effect on blood lipids of two daily servings of Camembert cheese. An intervention trial in mildly hypercholesterolemic subjects . Int J Food Sci Nutr, 2014,65(8):1013-1018.

[1640] Schlievert P M, Case L C, Nemeth K A, et al. Alpha and beta chains of hemoglobin inhibit production of Staphylococcus aureus exotoxins . Biochemistry, 2007,46(50):14349-14358.

[1641] Schmidt H, Stuertz K, Chen V, et al. Glycerol does not reduce neuronal damage in experimental Streptococcus pneumoniae meningitis in rabbits . Inflammopharmacology, 1998,6(1):19-26.

[1642] Schmidt V, Wolter M, Lenschow U, et al. [Lactobacillus paracasei endocarditis in an 18-yeard-old patient with trisomy 21, atrioventricular septal defect and Eisenmenger complex: therapeutic problems] . Klin Padiatr, 2001,213(1):35-38.

[1643] Schreiber M D, Covert R F, Torgerson L J. Hemodynamic effects of heat-killed group B beta-hemolytic streptococcus in newborn lambs: role of leukotriene D4 . Pediatr Res, 1992,31(2):121-126.

[1644] Schreiber M D, Covert R F, Torgerson L J. Effect of aminophylline on the pulmonary and systemic hemodynamic response to group B beta-hemolytic Streptococcus and leukotriene D4 in newborn lambs . J Dev Physiol, 1992,17(4):195-200.

[1645] Schwedhelm E, von Lucadou M, Peine S, et al. Trimethyllysine, vascular risk factors and outcome in acute ischemic stroke (MARK-STROKE) . Amino Acids, 2021,53(4):555-561.

[1646] Schwienbacher M, Treml B, Pinna A, et al. Tolerability of inhaled N-chlorotaurine in an acute pig streptococcal lower airway inflammation model . BMC Infect Dis, 2011,11:231.

[1647] Scofield D, Black J, Wittenburg L, et al. Endometrial tissue and blood plasma concentration of ceftiofur and metabolites following intramuscular administration of ceftiofur crystalline free acid to mares . Equine Vet J, 2014,46(5):606-610.

[1648] Scrivo R, Gerardi M C, Rutigliano I, et al. Polymyalgia rheumatica and diverticular disease: just two distinct age-related disorders or more? Results from a case-control study . Clin Rheumatol, 2018,37(9):2573-2577.

[1649] Scudiero O, Pero R, Ranieri A, et al. Childhood obesity: an overview of laboratory medicine, exercise and microbiome . Clin Chem Lab Med, 2020,58(9):1385-1406.

[1650] Sears M R, O'Donoghue J M, Fisher H K, et al. Effect of experimental pneumococcal meningitis on respiration and circulation in the rabbit . J Clin Invest, 1974,54(1):18-23.

[1651] Sebastián M G, Angel S H, Irene V Z, et al. Abnormal maternal body mass index and obstetric and neonatal outcome . J Matern Fetal Neonatal Med, 2012,25(3):308-312.

[1652] Seedat M A, Feldman C, Skoularigis J, et al. A study of acute community-acquired pneumonia, including details of cardiac changes . Q J Med, 1993,86(10):669-675.

[1653] Sehatzadeh S. Influenza and pneumococcal vaccinations for patients with chronic obstructive pulmonary disease (COPD): an evidence-based review . Ont Health Technol Assess Ser, 2012,12(3):1-64.

[1654] Seifert H. The clinical importance of microbiological findings in the diagnosis and management of bloodstream infections . Clin Infect Dis, 2009,48 Suppl 4:S238-S245.

[1655] Sekine T, Nagai H, Hamada-Sato N. Antihypertensive and Probiotic Effects of Hidakakombu (Saccharina angustata) Fermented by Lacticaseibacillus casei 001 . Foods, 2021,10(9).

[1656] Sekkarie A, Welsh J A, Vos M B. Carbohydrates and diet patterns in nonalcoholic fatty liver disease in children and adolescents . Curr Opin Clin Nutr Metab Care, 2018,21(4):283-288.

[1657] Senthong V, Kiatchoosakun S, Wongvipaporn C, et al. Gut microbiota-generated metabolite, trimethylamine-N-oxide, and subclinical myocardial damage: a multicenter study from Thailand . Sci Rep, 2021,11(1):14963.

[1658] Sepp E, Julge K, Mikelsaar M, et al. Intestinal microbiota and immunoglobulin E responses in 5-year-old Estonian children . Clin Exp Allergy, 2005,35(9):1141-1146.

[1659] Sequi-Canet J M, Sala-Langa M J, Collar D C J. [Perinatal factors affecting the detection of otoacoustic emissions in vaginally delivered, healthy newborns, during the first 48 hours of life] . Acta Otorrinolaringol Esp, 2014,65(1):1-7.

[1660] Serena C, Ceperuelo-Mallafré V, Keiran N, et al. Elevated circulating levels of succinate in human obesity are linked to specific gut microbiota . ISME J, 2018,12(7):1642-1657.

[1661] Serradilla M M, Oliver G J, Palomares C A, et al. Metabolic syndrome, non-alcoholic fatty liver disease and hepatocarcinoma . Rev Esp Enferm Dig, 2020,112(2):133-138.

[1662] Serrano L, Ruiz L A, Martinez-Indart L, et al. Non-bacteremic pneumococcal pneumonia: general characteristics and early predictive factors for poor outcome . Infect Dis (Lond), 2020,52(9):603-611.

[1663] Sesso R, Pinto S W. Five-year follow-up of patients with epidemic glomerulonephritis due to Streptococcus zooepidemicus . Nephrol Dial Transplant, 2005,20(9):1808-1812.

[1664] Sesso R, Wyton S, Pinto L. Epidemic glomerulonephritis due to Streptococcus zooepidemicus in Nova Serrana, Brazil . Kidney Int Suppl, 2005(97):S132-S136.

[1665] Séverin S, Wenshui X. Milk biologically active components as nutraceuticals: review . Crit Rev Food Sci Nutr, 2005,45(7-8):645-656.

[1666] Shabanzadeh D M. New determinants for gallstone disease?  . Dan Med J, 2018,65(2).

[1667] Shabbir U, Rubab M, Daliri E B, et al. Curcumin, Quercetin, Catechins and Metabolic Diseases: The Role of Gut Microbiota . Nutrients, 2021,13(1).

[1668] Shah R D, Tang Z Z, Chen G, et al. Soy food intake associates with changes in the metabolome and reduced blood pressure in a gut microbiota dependent manner . Nutr Metab Cardiovasc Dis, 2020,30(9):1500-1511.

[1669] Shan Q, Zhu X, Liu S, et al. Pharmacokinetics of cefquinome in tilapia (Oreochromis niloticus) after a single intramuscular or intraperitoneal administration . J Vet Pharmacol Ther, 2015,38(6):601-605.

[1670] Shannon E, Conlon M, Hayes M. Seaweed Components as Potential Modulators of the Gut Microbiota . Mar Drugs, 2021,19(7).

[1671] Shanson D C, Shehata A, Tadayon M, et al. Comparison of intravenous teicoplanin with intramuscular amoxycillin for the prophylaxis of streptococcal bacteraemia in dental patients . J Antimicrob Chemother, 1987,20(1):85-93.

[1672] Shao F, Xin F Z, Yang C G, et al. The impact of microbial immune enteral nutrition on the patients with acute radiation enteritis in bowel function and immune status . Cell Biochem Biophys, 2014,69(2):357-361.

[1673] Sharafedtinov K K, Plotnikova O A, Alexeeva R I, et al. Hypocaloric diet supplemented with probiotic cheese improves body mass index and blood pressure indices of obese hypertensive patients--a randomized double-blind placebo-controlled pilot study . Nutr J, 2013,12:138.

[1674] Sharafetdinov K, Plotnikova O A, Alekseeva R I, et al. [Influence of a low-calorie diet with inclusion of probiotic product containing bacterias Lactobacillus plantarum Tensia DSM 21380 on clinical and metabolic characteristics in patients with obesity and arterial hypertension] . Vopr Pitan, 2012,81(1):80-85.

[1675] Sharafi S, Nateghi L. Optimization of gamma-aminobutyric acid production by probiotic bacteria through response surface methodology . Iran J Microbiol, 2020,12(6):584-591.

[1676] Sharma A, Mannuru D, Matta A, et al. Rare complication of ceftriaxone therapy: drug-induced thrombocytopenia (DITP) . BMJ Case Rep, 2021,14(9).

[1677] Sharma R K, Oliveira A C, Yang T, et al. Gut Pathology and Its Rescue by ACE2 (Angiotensin-Converting Enzyme 2) in Hypoxia-Induced Pulmonary Hypertension . Hypertension, 2020,76(1):206-216.

[1678] Sharma R K, Oliveira A C, Yang T, et al. Pulmonary arterial hypertension-associated changes in gut pathology and microbiota . ERJ Open Res, 2020,6(3).

[1679] Sharma R K, Yang T, Oliveira A C, et al. Microglial Cells Impact Gut Microbiota and Gut Pathology in Angiotensin II-Induced Hypertension . Circ Res, 2019,124(5):727-736.

[1680] Sharma S, Tripathi P. Gut microbiome and type 2 diabetes: where we are and where to go? . J Nutr Biochem, 2019,63:101-108.

[1681] Shay J W, Homma N, Zhou R, et al. Abstracts from the 3rd International Genomic Medicine Conference (3rd IGMC 2015) : Jeddah, Kingdom of Saudi Arabia. 30 November - 3 December 2015 . BMC Genomics, 2016,17 Suppl 6(Suppl 6):487.

[1682] Sheeran P W, Maass D L, White D J, et al. Aspiration pneumonia-induced sepsis increases cardiac dysfunction after burn trauma . J Surg Res, 1998,76(2):192-199.

[1683] Sheng S, Chen J, Zhang Y, et al. Structural and Functional Alterations of Gut Microbiota in Males With Hyperuricemia and High Levels of Liver Enzymes . Front Med (Lausanne), 2021,8:779994.

[1684] Sherf-Dagan S, Zelber-Sagi S, Buch A, et al. Prospective Longitudinal Trends in Body Composition and Clinical Outcomes 3 Years Following Sleeve Gastrectomy . Obes Surg, 2019,29(12):3833-3841.

[1685] Sheykhsaran E, Abbasi A, Ebrahimzadeh L H, et al. Gut microbiota and obesity: an overview of microbiota to microbial-based therapies . Postgrad Med J, 2022.

[1686] Shi H, Zhang B, Abo-Hamzy T, et al. Restructuring the Gut Microbiota by Intermittent Fasting Lowers Blood Pressure . Circ Res, 2021,128(9):1240-1254.

[1687] Shi Q, Dai L, Zhao Q, et al. A review on the effect of gut microbiota on metabolic diseases . Arch Microbiol, 2022,204(3):192.

[1688] Shi Z, Papier K, Yiengprugsawan V, et al. Dietary patterns associated with hypertension risk among adults in Thailand: 8-year findings from the Thai Cohort Study . Public Health Nutr, 2019,22(2):307-313.

[1689] Shi Z, Yin Y, Li C, et al. Lipocalin-2-induced proliferative endoplasmic reticulum stress participates in Kawasaki disease-related pulmonary arterial abnormalities . Sci China Life Sci, 2021,64(6):1000-1012.

[1690] Shiga Y, Aoki S, Hosomi N, et al. cnm-Positive Streptococcus mutans and diffusion-weighted imaging hyperintensities in acute intracerebral hemorrhage . Eur J Neurol, 2021,28(5):1581-1589.

[1691] Shikata F, Shimada K, Sato H, et al. Potential Influences of Gut Microbiota on the Formation of Intracranial Aneurysm . Hypertension, 2019,73(2):491-496.

[1692] Shin J H, Sim M, Lee J Y, et al. Lifestyle and geographic insights into the distinct gut microbiota in elderly women from two different geographic locations . J Physiol Anthropol, 2016,35(1):31.

[1693] Shiry N, Shomali T, Soltanian S, et al. Comparative single-dose pharmacokinetics of orally administered florfenicol in rainbow trout (Oncorhynchus mykiss, Walbaum, 1792) at health and experimental infection with Streptococcus iniae or Lactococcus garvieae . J Vet Pharmacol Ther, 2019,42(2):214-221.

[1694] Shobako N, Ohinata K. Anti-Hypertensive Effects of Peptides Derived from Rice Bran Protein . Nutrients, 2020,12(10).

[1695] Shomorony I, Cirulli E T, Huang L, et al. An unsupervised learning approach to identify novel signatures of health and disease from multimodal data . Genome Med, 2020,12(1):7.

[1696] Shook L A, Pauly T H, Marple S L, et al. Group B streptococcus promotes oxygen radical-dependent thromboxane accumulation in young piglets . Pediatr Res, 1990,27(4 Pt 1):349-352.

[1697] Short B L, Miller M K, Pan J. Group B streptococcal (GBSS) newborn septic shock model: the role of prostaglandins . Prog Clin Biol Res, 1988,264:333-336.

[1698] Shu G, Shi X, Chen H, et al. Optimization of Nutrient Composition for Producing ACE Inhibitory Peptides from Goat Milk Fermented by Lactobacillus bulgaricus LB6 . Probiotics Antimicrob Proteins, 2019,11(2):723-729.

[1699] Šiarnik P, Klobučníková K, Mucska I, et al. Obstructive sleep apnea and hypertension: the role of gut microbiome . Vnitr Lek, 2020,66(7):415-419.

[1700] Siener R, Bade D J, Hesse A, et al. Dietary hyperoxaluria is not reduced by treatment with lactic acid bacteria . J Transl Med, 2013,11:306.

[1701] Sihag J, Di Marzo V. (Wh)olistic (E)ndocannabinoidome-Microbiome-Axis Modulation through (N)utrition (WHEN) to Curb Obesity and Related Disorders . Lipids Health Dis, 2022,21(1):9.

[1702] Sikand G, Severson T. Top 10 dietary strategies for atherosclerotic cardiovascular risk reduction . Am J Prev Cardiol, 2020,4:100106.

[1703] Sikora M, Kiss N, Stec A, et al. Trimethylamine N-Oxide, a Gut Microbiota-Derived Metabolite, Is Associated with Cardiovascular Risk in Psoriasis: A Cross-Sectional Pilot Study . Dermatol Ther (Heidelb), 2021,11(4):1277-1289.

[1704] Siltari A, Kivimäki A S, Ehlers P I, et al. Effects of milk casein derived tripeptides on endothelial enzymes in vitro; a study with synthetic tripeptides . Arzneimittelforschung, 2012,62(10):477-481.

[1705] Silva H, Balthazar C F, Silva R, et al. Sodium reduction and flavor enhancer addition in probiotic prato cheese: Contributions of quantitative descriptive analysis and temporal dominance of sensations for sensory profiling . J Dairy Sci, 2018,101(10):8837-8846.

[1706] Silveira-Nunes G, Durso D F, Jr L, et al. Hypertension Is Associated With Intestinal Microbiota Dysbiosis and Inflammation in a Brazilian Population . Front Pharmacol, 2020,11:258.

[1707] Simbrunner B, Mandorfer M, Trauner M, et al. Gut-liver axis signaling in portal hypertension . World J Gastroenterol, 2019,25(39):5897-5917.

[1708] Singh M, Hardin S J, George A K, et al. Epigenetics, 1-Carbon Metabolism, and Homocysteine During Dysbiosis . Front Physiol, 2020,11:617953.

[1709] Singhi S, Järvinen A, Peltola H. Increase in serum osmolality is possible mechanism for the beneficial effects of glycerol in childhood bacterial meningitis . Pediatr Infect Dis J, 2008,27(10):892-896.

[1710] Singhi S, Singhi P, Baranwal A K. Bacterial meningitis in children: critical care needs . Indian J Pediatr, 2001,68(8):737-747.

[1711] Sinniah R, Javier A R, Ku G. The pathology of mesangial IgA nephritis with clinical correlation . Histopathology, 1981,5(5):469-490.

[1712] Siqueira F, Ferreira E M, de Matos C I, et al. Prevalence of colonisation by group B streptococcus in pregnant patients in Taguatinga, Federal District, Brazil: a cross-sectional study . Arch Gynecol Obstet, 2019,299(3):703-711.

[1713] Sirchak I. [Comparative evaluation of the effectiveness of different schemes of correction the portal system pressure in patients with liver cirrhosis] . Lik Sprava, 2013(3):32-39.

[1714] Siregar D, Rianda D, Irwinda R, et al. Associations between diet quality, blood pressure, and glucose levels among pregnant women in the Asian megacity of Jakarta . PLoS One, 2020,15(11):e242150.

[1715] Sirtori C R, Arnoldi A, Cicero A F. Nutraceuticals for blood pressure control . Ann Med, 2015,47(6):447-456.

[1716] Sirtori C R, Pavanello C, Calabresi L, et al. Nutraceutical approaches to metabolic syndrome . Ann Med, 2017,49(8):678-697.

[1717] Sivamaruthi B S, Fern L A, Rashidah P H I D, et al. The influence of probiotics on bile acids in diseases and aging . Biomed Pharmacother, 2020,128:110310.

[1718] Sivamaruthi B S, Kesika P, Chaiyasut C. The Influence of Supplementation of Anthocyanins on Obesity-Associated Comorbidities: A Concise Review . Foods, 2020,9(6).

[1719] Skagen K, Trøseid M, Ueland T, et al. The Carnitine-butyrobetaine-trimethylamine-N-oxide pathway and its association with cardiovascular mortality in patients with carotid atherosclerosis . Atherosclerosis, 2016,247:64-69.

[1720] Skarke C, Lahens N F, Rhoades S D, et al. A Pilot Characterization of the Human Chronobiome . Sci Rep, 2017,7(1):17141.

[1721] Skrzypecki J, Izdebska J, Kamińska A, et al. Glaucoma patients have an increased level of trimethylamine, a toxic product of gut bacteria, in the aqueous humor: a pilot study . Int Ophthalmol, 2021,41(1):341-347.

[1722] SMELLIE J M, HODSON C J, EDWARDS D, et al. CLINICAL AND RADIOLOGICAL FEATURES OF URINARY INFECTION IN CHILDHOOD . Br Med J, 1964,2(5419):1222-1226.

[1723] Smiljanec K, Lennon S L. Sodium, hypertension, and the gut: does the gut microbiota go salty? . Am J Physiol Heart Circ Physiol, 2019,317(6):H1173-H1182.

[1724] Smit S, Szymańska E, Kunz I, et al. Nutrikinetic modeling reveals order of genistein phase II metabolites appearance in human plasma . Mol Nutr Food Res, 2014,58(11):2111-2121.

[1725] Smith C, Arregui L M, Promnitz D A, et al. Septic shock in the Intensive Care Unit, Hillbrow Hospital, Johannesburg . S Afr Med J, 1991,80(4):181-184.

[1726] Snelson M, R M R, Dinakis E, et al. Renal ACE2 (Angiotensin-Converting Enzyme 2) Expression Is Modulated by Dietary Fiber Intake, Gut Microbiota, and Their Metabolites . Hypertension, 2021,77(6):e53-e55.

[1727] Soedamah-Muthu S S, Verberne L D, Ding E L, et al. Dairy consumption and incidence of hypertension: a dose-response meta-analysis of prospective cohort studies . Hypertension, 2012,60(5):1131-1137.

[1728] Solé A, Jordan I, Bobillo S, et al. Venoarterial extracorporeal membrane oxygenation support for neonatal and pediatric refractory septic shock: more than 15 years of learning . Eur J Pediatr, 2018,177(8):1191-1200.

[1729] Søndertoft N B, Vogt J K, Arumugam M, et al. The intestinal microbiome is a co-determinant of the postprandial plasma glucose response . PLoS One, 2020,15(9):e238648.

[1730] Sonestedt E, Wirfält E, Wallström P, et al. Dairy products and its association with incidence of cardiovascular disease: the Malmö diet and cancer cohort . Eur J Epidemiol, 2011,26(8):609-618.

[1731] Song A, Scott I U, Flynn H J, et al. Delayed-onset bleb-associated endophthalmitis: clinical features and visual acuity outcomes . Ophthalmology, 2002,109(5):985-991.

[1732] Song S C, An Y M, Shin J H, et al. Beneficial effects of a probiotic blend on gastrointestinal side effects induced by leflunomide and amlodipine in a rat model . Benef Microbes, 2017,8(5):801-808.

[1733] Song X, Xiong Z, Kong L, et al. Relationship Between Putative eps Genes and Production of Exopolysaccharide in Lactobacillus casei LC2W . Front Microbiol, 2018,9:1882.

[1734] Song Z, Wang Y, Zhang F, et al. Calcium Signaling Pathways: Key Pathways in the Regulation of Obesity . Int J Mol Sci, 2019,20(11).

[1735] Soraci A L, Perez D S, Martinez G, et al. Disodium-fosfomycin pharmacokinetics and bioavailability in post weaning piglets . Res Vet Sci, 2011,90(3):498-502.

[1736] Sorensen G K, Redding G J, Truog W E. Mechanisms of pulmonary gas exchange abnormalities during experimental group B streptococcal infusion . Pediatr Res, 1985,19(9):922-926.

[1737] Soriano G, Sánchez E, Guarner C. [Probiotics in liver diseases] . Nutr Hosp, 2013,28(3):558-563.

[1738] Sorribas M, Jakob M O, Yilmaz B, et al. FXR modulates the gut-vascular barrier by regulating the entry sites for bacterial translocation in experimental cirrhosis . J Hepatol, 2019,71(6):1126-1140.

[1739] Soto-Barreras U, Olvera-Rubio J O, Loyola-Rodriguez J P, et al. Peripheral arterial disease associated with caries and periodontal disease . J Periodontol, 2013,84(4):486-494.

[1740] Souders C N, Zubcevic J, Martyniuk C J. Tumor Necrosis Factor Alpha and the Gastrointestinal Epithelium: Implications for the Gut-Brain Axis and Hypertension . Cell Mol Neurobiol, 2022,42(2):419-437.

[1741] Soukup S T, Al-Maharik N, Botting N, et al. Quantification of soy isoflavones and their conjugative metabolites in plasma and urine: an automated and validated UHPLC-MS/MS method for use in large-scale studies . Anal Bioanal Chem, 2014,406(24):6007-6020.

[1742] Souza M H, Helito C P, Oliva G B, et al. Clinical and epidemiological characteristics of septic arthritis of the hip, 2006 to 2012, a seven-year review . Clinics (Sao Paulo), 2014,69(7):464-468.

[1743] Sowinski K M, Lucksiri A, Kays M B, et al. Levofloxacin pharmacokinetics in ESRD and removal by the cellulose acetate high performance-210 hemodialyzer . Am J Kidney Dis, 2003,42(2):342-349.

[1744] Spahis S, Borys J M, Levy E. Metabolic Syndrome as a Multifaceted Risk Factor for Oxidative Stress . Antioxid Redox Signal, 2017,26(9):445-461.

[1745] Spanos P K, Simmons R L, Kjellstrand C M, et al. Screening potential related transplant donors for renal disease . Lancet, 1974,1(7859):645-649.

[1746] Sparenborg J D, Brems J A, Wood A M, et al. Fournier's gangrene: a modern analysis of predictors of outcomes . Transl Androl Urol, 2019,8(4):374-378.

[1747] Spearman C W, Afihene M, Betiku O, et al. Epidemiology, risk factors, social determinants of health, and current management for non-alcoholic fatty liver disease in sub-Saharan Africa . Lancet Gastroenterol Hepatol, 2021,6(12):1036-1046.

[1748] Spence J D. Reducing the Risk of Stroke in Patients with Impaired Renal Function: Nutritional Issues . J Stroke Cerebrovasc Dis, 2021,30(9):105376.

[1749] Spence J D, Azarpazhooh M R, Larsson S C, et al. Stroke Prevention in Older Adults: Recent Advances . Stroke, 2020,51(12):3770-3777.

[1750] Špičák J, Kučera M, Suchánková G. Diverticular disease: diagnosis and treatment . Vnitr Lek, 2018,64(6):621-634.

[1751] Stach S C, Brizot M L, Liao A W, et al. Placental transfer of IgG antibodies specific to Klebsiella and Pseudomonas LPS and to group B Streptococcus in twin pregnancies . Scand J Immunol, 2015,81(2):135-141.

[1752] Stass H, Kubitza D, Schühly U. Pharmacokinetics, safety and tolerability of moxifloxacin, a novel 8-methoxyfluoroquinolone, after repeated oral administration . Clin Pharmacokinet, 2001,40 Suppl 1:1-9.

[1753] Steffen H M, Demir M. [Gut Microbiome and Cardiovascular Disease] . Dtsch Med Wochenschr, 2019,144(14):957-963.

[1754] Stegmayr B, Björck S, Holm S, et al. Septic shock induced by group A streptococcal infection: clinical and therapeutic aspects . Scand J Infect Dis, 1992,24(5):589-597.

[1755] Steib A, Jacoberger B, Von Bandel M, et al. Concentrations in plasma and tissue penetration of ceftriaxone and ornidazole during liver transplantation . Antimicrob Agents Chemother, 1993,37(9):1873-1876.

[1756] Steib C J, Schewe J, Gerbes A L. Infection as a Trigger for Portal Hypertension . Dig Dis, 2015,33(4):570-576.

[1757] Stevens B R, Pepine C J, Richards E M, et al. Depressive hypertension: A proposed human endotype of brain/gut microbiome dysbiosis . Am Heart J, 2021,239:27-37.

[1758] Stevens D L, Bryant A E, Hackett S P, et al. Group A streptococcal bacteremia: the role of tumor necrosis factor in shock and organ failure . J Infect Dis, 1996,173(3):619-626.

[1759] Steves C J, Jackson M A. Response to: Population-Based Gut Microbiome Associations With Hypertension . Circ Res, 2018,123(11):1188-1189.

[1760] Stewart C J, Mansbach J M, Ajami N J, et al. Serum Metabolome Is Associated With the Nasopharyngeal Microbiota and Disease Severity Among Infants With Bronchiolitis . J Infect Dis, 2019,219(12):2005-2014.

[1761] Stewart D C, Rubiano A, Santisteban M M, et al. Hypertension-linked mechanical changes of rat gut . Acta Biomater, 2016,45:296-302.

[1762] Stewart J P. The Histopathology of Mastoiditis . Proc R Soc Med, 1928,21(10):1743-1758.

[1763] Stillson J E, Bunch C M, Thomas A V, et al. Pathologic fracture and hardware failure in Streptococcus anginosus femoral osteomyelitis: Case report . Ann Med Surg (Lond), 2021,67:102478.

[1764] Stone L P, Stone P M, Rydbom E A, et al. Customized nutritional enhancement for pregnant women appears to lower incidence of certain common maternal and neonatal complications: an observational study . Glob Adv Health Med, 2014,3(6):50-55.

[1765] Stowasser M. Aldosterone, gut microbiome and hypertension: selected papers from APCH 2019 . J Hum Hypertens, 2021,35(2):109.

[1766] Stratta P, Musetti C, Barreca A, et al. New trends of an old disease: the acute post infectious glomerulonephritis at the beginning of the new millenium . J Nephrol, 2014,27(3):229-239.

[1767] Stratton J E, Hutkins R W, Taylor S L. Biogenic Amines in Cheese and other Fermented Foods: A Review . J Food Prot, 1991,54(6):460-470.

[1768] Stroustrup A, Weintraub A S, Cadet C T, et al. Group B Streptococcus exposure and self-limited respiratory distress in late preterm and term neonates . Neonatology, 2013,104(3):210-215.

[1769] Sturm A, Noppeney R, Reimer J, et al. [AIDS and non-Hodgkin's lymphoma: initial cardiac manifestations of highly malignant B-cell lymphoma 18 years after HIV infection] . Dtsch Med Wochenschr, 2001,126(13):364-366.

[1770] Su C Y, Tsai T C, Wu K H, et al. Liver Cirrhosis Predisposes One to Complicated Deep Neck Infection: Retrospective Analysis of 161 Cases . J Acute Med, 2019,9(1):1-7.

[1771] Su L, Hong Z, Zhou T, et al. Health improvements of type 2 diabetic patients through diet and diet plus fecal microbiota transplantation . Sci Rep, 2022,12(1):1152.

[1772] Suadoni M T. Correspondence on 'A. Grylls, K. Seidler, J. Neil, Link between microbiota and hypertension: Focus on LPS/TLR4 pathway in endothelial dysfunction and vascular inflammation, and therapeutic implication of probiotics' . Biomed Pharmacother, 2021,138:111432.

[1773] Sudha M R, Bhonagiri S, Kumar M A. Oral consumption of potential probiotic Saccharomyces boulardii strain Unique 28 in patients with acute diarrhoea: a clinical report . Benef Microbes, 2012,3(2):145-150.

[1774] Sudha M R, Bhonagiri S, Kumar M A. Efficacy of Bacillus clausii strain UBBC-07 in the treatment of patients suffering from acute diarrhoea . Benef Microbes, 2013,4(2):211-216.

[1775] Sugawara J, Ochi D, Yamashita R, et al. Maternity Log study: a longitudinal lifelog monitoring and multiomics analysis for the early prediction of complicated pregnancy . BMJ Open, 2019,9(2):e25939.

[1776] Sugimoto N, Yamagishi Y, Hirai J, et al. Invasive pneumococcal disease caused by mucoid serotype 3 Streptococcus pneumoniae: a case report and literature review . BMC Res Notes, 2017,10(1):21.

[1777] Sugiyama M. [Histamine metabolism via histamine nucleotides] . Nihon Yakurigaku Zasshi, 1967,63(5):315-326.

[1778] Suguihara C, Goldberg R N, Hehre D, et al. Effect of cyclooxygenase and lipoxygenase products on pulmonary function in group B streptococcal sepsis . Pediatr Res, 1987,22(4):478-482.

[1779] Sullivan J L, Ochs H D, Schiffman G, et al. Immune response after splenectomy . Lancet, 1978,1(8057):178-181.

[1780] Sultan S, Huma N, Butt M S, et al. Therapeutic potential of dairy bioactive peptides: A contemporary perspective . Crit Rev Food Sci Nutr, 2018,58(1):105-115.

[1781] Sumida K, Lau W L, Kalantar-Zadeh K, et al. Novel intestinal dialysis interventions and microbiome modulation to control uremia . Curr Opin Nephrol Hypertens, 2022,31(1):82-91.

[1782] Sumida K, Molnar M Z, Potukuchi P K, et al. Constipation and risk of death and cardiovascular events . Atherosclerosis, 2019,281:114-120.

[1783] Summer A, Formaggioni P, Franceschi P, et al. Cheese as Functional Food: The Example of Parmigiano Reggiano and Grana Padano . Food Technol Biotechnol, 2017,55(3):277-289.

[1784] Sun G, Yin Z, Liu N, et al. Gut microbial metabolite TMAO contributes to renal dysfunction in a mouse model of diet-induced obesity . Biochem Biophys Res Commun, 2017,493(2):964-970.

[1785] Sun S, Lulla A, Sioda M, et al. Gut Microbiota Composition and Blood Pressure . Hypertension, 2019,73(5):998-1006.

[1786] Sun T, Zhang Y, Yin J, et al. Association of Gut Microbiota-Dependent Metabolite Trimethylamine N-Oxide with First Ischemic Stroke . J Atheroscler Thromb, 2021,28(4):320-328.

[1787] Susheela A K, Toteja G S. Prevention & control of fluorosis & linked disorders: Developments in the 21(st) Century - Reaching out to patients in the community & hospital settings for recovery . Indian J Med Res, 2018,148(5):539-547.

[1788] Susic D F, Wang L, Roberts L M, et al. The P4 Study: Postpartum Maternal and Infant Faecal Microbiome 6 Months After Hypertensive Versus Normotensive Pregnancy . Front Cell Infect Microbiol, 2022,12:646165.

[1789] Susic D, Davis G, O' S A, et al. Microbiome Understanding in Maternity Study (MUMS), an Australian prospective longitudinal cohort study of maternal and infant microbiota: study protocol . BMJ Open, 2020,10(9):e40189.

[1790] Sussman N L. Treatment of Overt Hepatic Encephalopathy . Clin Liver Dis, 2015,19(3):551-563.

[1791] Suwandecha T, Srichana T, Balekar N, et al. Novel antimicrobial peptide specifically active against Porphyromonas gingivalis . Arch Microbiol, 2015,197(7):899-909.

[1792] Suzzi G, Gardini F. Biogenic amines in dry fermented sausages: a review . Int J Food Microbiol, 2003,88(1):41-54.

[1793] Sweeney M R, McPartlin J, Weir D G, et al. Measurements of sub-nanomolar concentrations of unmetabolised folic acid in serum . J Chromatogr B Analyt Technol Biomed Life Sci, 2003,788(1):187-191.

[1794] Swithers S E. Artificial sweeteners are not the answer to childhood obesity . Appetite, 2015,93:85-90.

[1795] Syrjänen J, Valtonen V V, Iivanainen M, et al. Preceding infection as an important risk factor for ischaemic brain infarction in young and middle aged patients . Br Med J (Clin Res Ed), 1988,296(6630):1156-1160.

[1796] Sze C, Pressler M, Lee J R, et al. The gut, vaginal, and urine microbiome in overactive bladder: a systematic review . Int Urogynecol J, 2022.

[1797] Szurlej B, Bidiuk J. Peripheral arterial embolism and pyrexia in a young patient without comorbidities . Pol Merkur Lekarski, 2020,48(287):346-348.

[1798] Tabayashi A, Komiya T, Tsuneyoshi H, et al. [Valve Replacement for Infective Endocarditis of Pulmonary Valve;Report of a Case] . Kyobu Geka, 2017,70(13):1115-1119.

[1799] Tachalov V V, Orekhova L Y, Kudryavtseva T V, et al. Making a complex dental care tailored to the person: population health in focus of predictive, preventive and personalised (3P) medical approach . EPMA J, 2021,12(2):129-140.

[1800] Taché Y, Saavedra J M. Introduction to the Special Issue "The Brain-Gut Axis" . Cell Mol Neurobiol, 2022,42(2):311-313.

[1801] Tada H, Takamura M, Kawashiri M A. The Effect of Diet on Cardiovascular Disease, Heart Disease, and Blood Vessels . Nutrients, 2022,14(2).

[1802] Tagliazucchi D, Baldaccini A, Martini S, et al. Cultivable non-starter lactobacilli from ripened Parmigiano Reggiano cheeses with different salt content and their potential to release anti-hypertensive peptides . Int J Food Microbiol, 2020,330:108688.

[1803] Tain Y L, Lee W C, Wu K, et al. Resveratrol Prevents the Development of Hypertension Programmed by Maternal Plus Post-Weaning High-Fructose Consumption through Modulation of Oxidative Stress, Nutrient-Sensing Signals, and Gut Microbiota . Mol Nutr Food Res, 2018:e1800066.

[1804] Takagi T, Naito Y, Kashiwagi S, et al. Changes in the Gut Microbiota are Associated with Hypertension, Hyperlipidemia, and Type 2 Diabetes Mellitus in Japanese Subjects . Nutrients, 2020,12(10).

[1805] Takano N, Yatabe M S, Yatabe J, et al. Fatal Fournier's gangrene caused by Clostridium ramosum in a patient with central diabetes insipidus and insulin-dependent diabetes mellitus: a case report . BMC Infect Dis, 2018,18(1):363.

[1806] Takano T. Anti-hypertensive activity of fermented dairy products containing biogenic peptides . Antonie Van Leeuwenhoek, 2002,82(1-4):333-340.

[1807] Takano Y, Matsuyama H, Fujita A, et al. [A case of urgent aortic valve replacement for infective endocarditis in pregnancy] . Masui, 2003,52(10):1086-1088.

[1808] Takeoka M, Takahashi T. Infectious and inflammatory disorders of the circulatory system and stroke in childhood . Curr Opin Neurol, 2002,15(2):159-164.

[1809] Takewaki F, Nakajima H, Takewaki D, et al. Habitual Dietary Intake Affects the Altered Pattern of Gut Microbiome by Acarbose in Patients with Type 2 Diabetes . Nutrients, 2021,13(6).

[1810] Taladrid D, de Celis M, Belda I, et al. Hypertension- and glycaemia-lowering effects of a grape-pomace-derived seasoning in high-cardiovascular risk and healthy subjects. Interplay with the gut microbiome . Food Funct, 2022,13(4):2068-2082.

[1811] Talarico V, Aloe M, Monzani A, et al. Hemolytic uremic syndrome in children . Minerva Pediatr, 2016,68(6):441-455.

[1812] Tan X, Zhou Y, Xu L, et al. The predictors of necrotizing enterocolitis in newborns with low birth weight: A retrospective analysis . Medicine (Baltimore), 2022,101(7):e28789.

[1813] Tanaka M. Improving obesity and blood pressure . Hypertens Res, 2020,43(2):79-89.

[1814] Tanaka M, Itoh H. Hypertension as a Metabolic Disorder and the Novel Role of the Gut . Curr Hypertens Rep, 2019,21(8):63.

[1815] Tandon P, Moncrief K, Madsen K, et al. Effects of probiotic therapy on portal pressure in patients with cirrhosis: a pilot study . Liver Int, 2009,29(7):1110-1115.

[1816] Tang L M, Su Y J, Lai Y C. The evaluation of microbiology and prognosis of fournier's gangrene in past five years . Springerplus, 2015,4(1):14.

[1817] Tang W H, Kitai T, Hazen S L. Gut Microbiota in Cardiovascular Health and Disease . Circ Res, 2017,120(7):1183-1196.

[1818] Tang W H, Wang Z, Shrestha K, et al. Intestinal microbiota-dependent phosphatidylcholine metabolites, diastolic dysfunction, and adverse clinical outcomes in chronic systolic heart failure . J Card Fail, 2015,21(2):91-96.

[1819] Tang W, Li D Y, Hazen S L. Dietary metabolism, the gut microbiome, and heart failure . Nat Rev Cardiol, 2019,16(3):137-154.

[1820] Taniguchi H, Tanisawa K, Sun X, et al. Effects of short-term endurance exercise on gut microbiota in elderly men . Physiol Rep, 2018,6(23):e13935.

[1821] Tansy M F, Austin R W, Venturella V S. In vitro demonstration of a vasodepressant enhanced by lactobacillus casei . J Dent Res, 1967,46(5):1108.

[1822] Tao W, Maass D L, Johnston W E, et al. Murine in vivo myocardial contractile dysfunction after burn injury is exacerbated by pneumonia sepsis . Shock, 2005,24(5):495-499.

[1823] Tao X, Wang N, Qin W. Gut Microbiota and Hepatocellular Carcinoma . Gastrointest Tumors, 2015,2(1):33-40.

[1824] Tapper E B, Jiang Z G, Patwardhan V R. Refining the ammonia hypothesis: a physiology-driven approach to the treatment of hepatic encephalopathy . Mayo Clin Proc, 2015,90(5):646-658.

[1825] Tarpey M N, Graybar G B, Lyrene R K, et al. Thromboxane synthesis inhibition reverses group B Streptococcus-induced pulmonary hypertension . Crit Care Med, 1987,15(7):644-647.

[1826] Tasic V, Polenakovic M. Acute poststreptococcal glomerulonephritis following circumcision . Pediatr Nephrol, 2000,15(3-4):274-275.

[1827] Täuber M G, Burroughs M, Niemöller U M, et al. Differences of pathophysiology in experimental meningitis caused by three strains of Streptococcus pneumoniae . J Infect Dis, 1991,163(4):806-811.

[1828] Täuber M G, Sande M A. Pathogenesis of bacterial meningitis: contributions by experimental models in rabbits . Infection, 1984,12 Suppl 1:S3-S10.

[1829] Taylor W R, Takemiya K. Hypertension Opens the Flood Gates to the Gut Microbiota . Circ Res, 2017,120(2):249-251.

[1830] Tennant I, Harding H, Nelson M, et al. Microbial isolates from patients in an intensive care unit, and associated risk factors . West Indian Med J, 2005,54(4):225-231.

[1831] Thenappan T, Khoruts A, Chen Y, et al. Can intestinal microbiota and circulating microbial products contribute to pulmonary arterial hypertension? . Am J Physiol Heart Circ Physiol, 2019,317(5):H1093-H1101.

[1832] Thiem U, Heppner H J, Pientka L. Elderly patients with community-acquired pneumonia: optimal treatment strategies . Drugs Aging, 2011,28(7):519-537.

[1833] Tholstrup T. Dairy products and cardiovascular disease . Curr Opin Lipidol, 2006,17(1):1-10.

[1834] Thomas D J, Husmann R J, Villamar M, et al. Lactobacillus rhamnosus HN001 attenuates allergy development in a pig model . PLoS One, 2011,6(2):e16577.

[1835] Thomas M S, Fernandez M L. Trimethylamine N-Oxide (TMAO), Diet and Cardiovascular Disease . Curr Atheroscler Rep, 2021,23(4):12.

[1836] Thompson G R, Crawford G E. Pneumorachis caused by metastatic gas gangrene . Diagn Microbiol Infect Dis, 2009,63(1):108-110.

[1837] Thongprayoon C, Cheungpasitporn W, Srivali N, et al. Renal involvements in reported cases of Streptococcus bovis endocarditis . Int J Cardiol, 2015,181:179.

[1838] Thongprayoon C, Kaewput W, Hatch S T, et al. Effects of Probiotics on Inflammation and Uremic Toxins Among Patients on Dialysis: A Systematic Review and Meta-Analysis . Dig Dis Sci, 2019,64(2):469-479.

[1839] Thushara R M, Gangadaran S, Solati Z, et al. Cardiovascular benefits of probiotics: a review of experimental and clinical studies . Food Funct, 2016,7(2):632-642.

[1840] Tian M, Wang X, Xiao Y, et al. A rare case of diabetic hand ulcer caused by Streptococcus agalactiae . Int J Low Extrem Wounds, 2012,11(3):174-176.

[1841] Tian Y, Cai J, Allman E L, et al. Quantitative Analysis of Bile Acid with UHPLC-MS/MS . Methods Mol Biol, 2021,2194:291-300.

[1842] Tien K J, Chen T C, Hsieh M C, et al. Acute suppurative thyroiditis with deep neck infection: a case report . Thyroid, 2007,17(5):467-469.

[1843] Tirandaz H, Ebrahim-Habibi M B, Moradveisi B, et al. Microbiota potential for the treatment of sexual dysfunction . Med Hypotheses, 2018,115:46-49.

[1844] Tokarek J, Gadzinowska J, Młynarska E, et al. What Is the Role of Gut Microbiota in Obesity Prevalence? A Few Words about Gut Microbiota and Its Association with Obesity and Related Diseases . Microorganisms, 2021,10(1).

[1845] Tokumasu H, Watabe S, Tokumasu S. Effect of hemodiafiltration therapy in a low-birthweight infant with congenital sepsis . Pediatr Int, 2016,58(3):237-240.

[1846] Tokunaga T. [A present situation of Foods for Specified Health Use (FOSHU) in Japan] . Nihon Yakurigaku Zasshi, 1997,110 Suppl 1:17P-22P.

[1847] Tomarelli R G, Donoso F A. [Infectious glomerulonephritis and pleuropneumonia due to Streptococcus pneumoniae. Pediatric clinical case] . Arch Argent Pediatr, 2020,118(2):e208-e210.

[1848] Tomás C C, Oliveira E, Sousa D, et al. Proceedings of the 3rd IPLeiria's International Health Congress : Leiria, Portugal. 6-7 May 2016 . BMC Health Serv Res, 2016,16 Suppl 3(Suppl 3):200.

[1849] Tomas-Barberán F, Osorio C. Advances in Health-Promoting Food Ingredients . J Agric Food Chem, 2019,67(33):9121-9123.

[1850] Tomasova L, Grman M, Ondrias K, et al. The impact of gut microbiota metabolites on cellular bioenergetics and cardiometabolic health . Nutr Metab (Lond), 2021,18(1):72.

[1851] Tomasova L, Konopelski P, Ufnal M. Gut Bacteria and Hydrogen Sulfide: The New Old Players in Circulatory System Homeostasis . Molecules, 2016,21(11).

[1852] Tomlinson C W, Dhalla N S. Alterations in myocardial function during bacterial infective cardiomyopathy . Am J Cardiol, 1976,37(3):373-381.

[1853] Tomsett K I, Barrett H L, Dekker E E, et al. Dietary Fiber Intake Alters Gut Microbiota Composition but Does Not Improve Gut Wall Barrier Function in Women with Future Hypertensive Disorders of Pregnancy . Nutrients, 2020,12(12).

[1854] Tonomura S, Gyanwali B. Cerebral microbleeds in vascular dementia from clinical aspects to host-microbial interaction . Neurochem Int, 2021,148:105073.

[1855] Tonomura S, Ihara M, Friedland R P. Microbiota in cerebrovascular disease: A key player and future therapeutic target . J Cereb Blood Flow Metab, 2020,40(7):1368-1380.

[1856] Toral M, Robles-Vera I, de la Visitación N, et al. Critical Role of the Interaction Gut Microbiota - Sympathetic Nervous System in the Regulation of Blood Pressure . Front Physiol, 2019,10:231.

[1857] Toral M, Robles-Vera I, Romero M, et al. Lactobacillus fermentum CECT5716: a novel alternative for the prevention of vascular disorders in a mouse model of systemic lupus erythematosus . FASEB J, 2019,33(9):10005-10018.

[1858] Toral M, Romero M, Rodríguez-Nogales A, et al. Lactobacillus fermentum Improves Tacrolimus-Induced Hypertension by Restoring Vascular Redox State and Improving eNOS Coupling . Mol Nutr Food Res, 2018:e1800033.

[1859] Torino M I, Limón R I, Martínez-Villaluenga C, et al. Antioxidant and antihypertensive properties of liquid and solid state fermented lentils . Food Chem, 2013,136(2):1030-1037.

[1860] Tosukhowong A, Visessanguan W, Pumpuang L, et al. Biogenic amine formation in Nham, a Thai fermented sausage, and the reduction by commercial starter culture, Lactobacillus plantarum BCC 9546 . Food Chem, 2011,129(3):846-853.

[1861] Touyz R M. Gut Dysbiosis-Induced Hypertension Is Ameliorated by Intermittent Fasting . Circ Res, 2021,128(9):1255-1257.

[1862] Touyz R M, Camargo L L. Microglia, the Missing Link in the Brain-Gut-Hypertension Axis . Circ Res, 2019,124(5):671-673.

[1863] Touyz R M, Schiffrin E L. A Compendium on Hypertension: New Advances and Future Impact . Circ Res, 2021,128(7):803-807.

[1864] Tovar J, Nilsson A, Johansson M, et al. A diet based on multiple functional concepts improves cardiometabolic risk parameters in healthy subjects . Nutr Metab (Lond), 2012,9:29.

[1865] Towers R J, Fagan P K, Talay S R, et al. Evolution of sfbI encoding streptococcal fibronectin-binding protein I: horizontal genetic transfer and gene mosaic structure . J Clin Microbiol, 2003,41(12):5398-5406.

[1866] Toya T, Corban M T, Marrietta E, et al. Coronary artery disease is associated with an altered gut microbiome composition . PLoS One, 2020,15(1):e227147.

[1867] Toya T, Ozcan I, Corban M T, et al. Compositional change of gut microbiome and osteocalcin expressing endothelial progenitor cells in patients with coronary artery disease . PLoS One, 2021,16(3):e249187.

[1868] Tranah T H, Edwards L A, Schnabl B, et al. Targeting the gut-liver-immune axis to treat cirrhosis . Gut, 2021,70(5):982-994.

[1869] Trebicka J, Bork P, Krag A, et al. Utilizing the gut microbiome in decompensated cirrhosis and acute-on-chronic liver failure . Nat Rev Gastroenterol Hepatol, 2021,18(3):167-180.

[1870] Trebicka J, Macnaughtan J, Schnabl B, et al. The microbiota in cirrhosis and its role in hepatic decompensation . J Hepatol, 2021,75 Suppl 1(Suppl 1):S67-S81.

[1871] Tribble G D, Angelov N, Weltman R, et al. Frequency of Tongue Cleaning Impacts the Human Tongue Microbiome Composition and Enterosalivary Circulation of Nitrate . Front Cell Infect Microbiol, 2019,9:39.

[1872] Trigueros L, Peña S, Ugidos A V, et al. Food ingredients as anti-obesity agents: a review . Crit Rev Food Sci Nutr, 2013,53(9):929-942.

[1873] Trimigno A, Khakimov B, Savorani F, et al. Human urine (1)H NMR metabolomics reveals alterations of protein and carbohydrate metabolism when comparing habitual Average Danish diet vs. healthy New Nordic diet . Nutrition, 2020,79-80:110867.

[1874] Tripathi A K, Ray A K, Mishra S K. Molecular and pharmacological aspects of piperine as a potential molecule for disease prevention and management: evidence from clinical trials . Beni Suef Univ J Basic Appl Sci, 2022,11(1):16.

[1875] Tripp M L, Dahlberg C J, Eliason S, et al. A Low-Glycemic, Mediterranean Diet and Lifestyle Modification Program with Targeted Nutraceuticals Reduces Body Weight, Improves Cardiometabolic Variables and Longevity Biomarkers in Overweight Subjects: A 13-Week Observational Trial . J Med Food, 2019,22(5):479-489.

[1876] Trøseid M, Manner I W, Pedersen K K, et al. Microbial translocation and cardiometabolic risk factors in HIV infection . AIDS Res Hum Retroviruses, 2014,30(6):514-522.

[1877] Trošt K, Ulaszewska M M, Stanstrup J, et al. Host: Microbiome co-metabolic processing of dietary polyphenols - An acute, single blinded, cross-over study with different doses of apple polyphenols in healthy subjects . Food Res Int, 2018,112:108-128.

[1878] Truog W E, Gibson R L, Juul S E, et al. Neonatal group B streptococcal sepsis: effects of late treatment with dazmegrel . Pediatr Res, 1988,23(4):352-356.

[1879] Truog W E, Sorensen G K, Standaert T A, et al. Effects of the thromboxane synthetase inhibitor, dazmegrel (UK 38,485), on pulmonary gas exchange and hemodynamics in neonatal sepsis . Pediatr Res, 1986,20(5):481-486.

[1880] Tsai W C, Chang L K, Lin T C. Streptococcus constellatus causing myocardial abscess complicated by cerebritis . J Microbiol Immunol Infect, 2004,37(1):63-66.

[1881] Tseng C H. The Relationship between Diabetes Mellitus and Gastric Cancer and the Potential Benefits of Metformin: An Extensive Review of the Literature . Biomolecules, 2021,11(7).

[1882] Tung Y T, Hsu Y J, Liao C C, et al. Physiological and Biochemical Effects of Intrinsically High and Low Exercise Capacities Through Multiomics Approaches . Front Physiol, 2019,10:1201.

[1883] Tung Y T, Lee B H, Liu C F, et al. Optimization of culture condition for ACEI and GABA production by lactic acid bacteria . J Food Sci, 2011,76(9):M585-M591.

[1884] Tunick M H, Van Hekken D L. Dairy Products and Health: Recent Insights . J Agric Food Chem, 2015,63(43):9381-9388.

[1885] Tunovic E, Gawaziuk J, Bzura T, et al. Necrotizing fasciitis: a six-year experience . J Burn Care Res, 2012,33(1):93-100.

[1886] Tureen J H, Dworkin R J, Kennedy S L, et al. Loss of cerebrovascular autoregulation in experimental meningitis in rabbits . J Clin Invest, 1990,85(2):577-581.

[1887] Turkulov V, Ruzic M, Lendak D, et al. Non-cirrhotic Portal Hypertension Associated with Didanosine and Streptococcus agalactiae Infection: A Case Report . Rev Recent Clin Trials, 2016,11(2):152-155.

[1888] Turpeinen A M, Ehlers P I, Kivimäki A S, et al. Ile-Pro-Pro and Val-Pro-Pro tripeptide-containing milk product has acute blood pressure lowering effects in mildly hypertensive subjects . Clin Exp Hypertens, 2011,33(6):388-396.

[1889] Turpeinen A M, Ikonen M, Kivimäki A S, et al. A spread containing bioactive milk peptides Ile-Pro-Pro and Val-Pro-Pro, and plant sterols has antihypertensive and cholesterol-lowering effects . Food Funct, 2012,3(6):621-627.

[1890] Turpin W, Humblot C, Thomas M, et al. Lactobacilli as multifaceted probiotics with poorly disclosed molecular mechanisms . Int J Food Microbiol, 2010,143(3):87-102.

[1891] Tuteja S, Ferguson J F. Gut Microbiome and Response to Cardiovascular Drugs . Circ Genom Precis Med, 2019,12(9):421-429.

[1892] Tuttolomondo A, Simonetta I, Daidone M, et al. Metabolic and Vascular Effect of the Mediterranean Diet . Int J Mol Sci, 2019,20(19).

[1893] Tyner E, Oropeza M, Figueroa J, et al. Childhood Hypertension and Effects on Cognitive Functions: Mechanisms and Future Perspectives . CNS Neurol Disord Drug Targets, 2019,18(9):677-686.

[1894] Tzoulaki I, Iliou A, Mikros E, et al. An Overview of Metabolic Phenotyping in Blood Pressure Research . Curr Hypertens Rep, 2018,20(9):78.

[1895] Uchiyama K, Takami S, Suzuki H, et al. Efficacy and safety of short-term therapy with indigo naturalis for ulcerative colitis: An investigator-initiated multicenter double-blind clinical trial . PLoS One, 2020,15(11):e241337.

[1896] Udongwo N, Fareen N, Abe T, et al. Iatrogenic Infective Endocarditis With Septic Emboli: An Unusual Complication of Intracardiac Manipulation . J Med Cases, 2022,13(2):71-75.

[1897] Ulloa-Gutierrez R, Dobson S, Forbes J. Group A streptococcal subdural empyema as a complication of varicella . Pediatrics, 2005,115(1):e112-e114.

[1898] Umemori Y, Murai R, Asanuma K, et al. Luteinizing hormone (LH) formed a complex with an immunoglobulin G caused abnormally high levels of LH: A case report . Clin Biochem, 2021,93:33-35.

[1899] Underwood M A, Wedgwood S, Lakshminrusimha S, et al. Somatic growth and the risks of bronchopulmonary dysplasia and pulmonary hypertension: connecting epidemiology and physiology (1) . Can J Physiol Pharmacol, 2019,97(3):197-205.

[1900] Undritsov M I, Vasil'Eva G K, Frolov E P, et al. [Kininogen content of rabbit blood serum during immediate streptococcal allergy] . Biull Eksp Biol Med, 1970,70(7):39-42.

[1901] Unnikrishnan D, Delacruz M A, Saha A, et al. Case of acute paraspinal pyomyositis in an elderly diabetic secondary to spread from urinary tract infection . BMJ Case Rep, 2018,2018.

[1902] Unson M D, Newton G L, Davis C, et al. An immunoassay for the detection and quantitative determination of mycothiol . J Immunol Methods, 1998,214(1-2):29-39.

[1903] Upadrasta A, Madempudi R S. Probiotics and blood pressure: current insights . Integr Blood Press Control, 2016,9:33-42.

[1904] Usinger L, Ibsen H, Jensen L T. Does fermented milk possess antihypertensive effect in humans? . J Hypertens, 2009,27(6):1115-1120.

[1905] Usinger L, Ibsen H, Linneberg A, et al. Human in vivo study of the renin-angiotensin-aldosterone system and the sympathetic activity after 8 weeks daily intake of fermented milk . Clin Physiol Funct Imaging, 2010,30(2):162-168.

[1906] Usinger L, Reimer C, Ibsen H. Fermented milk for hypertension . Cochrane Database Syst Rev, 2012(4):D8118.

[1907] Vaidya S S, Pandit S, Apte N V, et al. Tricuspid valve endocarditis following septic abortion . J Assoc Physicians India, 1986,34(2):157.

[1908] Vallejo F, Larrosa M, Escudero E, et al. Concentration and solubility of flavanones in orange beverages affect their bioavailability in humans . J Agric Food Chem, 2010,58(10):6516-6524.

[1909] Valles J M, Fekete R. Gradenigo syndrome: unusual consequence of otitis media . Case Rep Neurol, 2014,6(2):197-201.

[1910] Vallianou N G, Geladari E, Kounatidis D. Microbiome and hypertension: where are we now? . J Cardiovasc Med (Hagerstown), 2020,21(2):83-88.

[1911] Vamanu E, Gatea F, Sârbu I, et al. An In Vitro Study of the Influence of Curcuma longa Extracts on the Microbiota Modulation Process, In Patients with Hypertension . Pharmaceutics, 2019,11(4).

[1912] van der Zander K, Jäkel M, Bianco V, et al. Fermented lactotripeptides-containing milk lowers daytime blood pressure in high normal-to-mild hypertensive subjects . J Hum Hypertens, 2008,22(11):804-806.

[1913] van der Zwaan H B, Sieswerda G T, Krings G J, et al. Infectious stentitis after treatment of coarctation of the aorta: a case report . Eur Heart J Case Rep, 2020,4(3):1-5.

[1914] van Duynhoven J, Vaughan E E, van Dorsten F, et al. Interactions of black tea polyphenols with human gut microbiota: implications for gut and cardiovascular health . Am J Clin Nutr, 2013,98(6 Suppl):1631S-1641S.

[1915] Van Meulebroek L, De Paepe E, Vercruysse V, et al. Holistic Lipidomics of the Human Gut Phenotype Using Validated Ultra-High-Performance Liquid Chromatography Coupled to Hybrid Orbitrap Mass Spectrometry . Anal Chem, 2017,89(22):12502-12510.

[1916] van Son J, Serlie M J, Ståhlman M, et al. Plasma Imidazole Propionate Is Positively Correlated with Blood Pressure in Overweight and Obese Humans . Nutrients, 2021,13(8).

[1917] Van Winden K R, Bearden A, Kono N, et al. Low Bioactive Vitamin D Is Associated with Pregnancy-Induced Hypertension in a Cohort of Pregnant HIV-Infected Women Sampled Over a 23-Year Period . Am J Perinatol, 2020,37(14):1446-1454.

[1918] Vandevelde N M, Tulkens P M, Diaz I Y, et al. Characterisation of a collection of Streptococcus pneumoniae isolates from patients suffering from acute exacerbations of chronic bronchitis: in vitro susceptibility to antibiotics and biofilm formation in relation to antibiotic efflux and serotypes/serogroups . Int J Antimicrob Agents, 2014,44(3):209-217.

[1919] Vandroux D, Nkusu A M, Gauzere B A, et al. Necrotizing Skin and Soft Tissue Infections Admitted to Intensive Care Unit in Reunion Island: A Retrospective Cohort Study . Am J Trop Med Hyg, 2021,105(3):596-599.

[1920] Vanhatalo A, Blackwell J R, L'Heureux J E, et al. Nitrate-responsive oral microbiome modulates nitric oxide homeostasis and blood pressure in humans . Free Radic Biol Med, 2018,124:21-30.

[1921] Vanichanan J, Chávez V, Wanger A, et al. Carbapenem-resistant Lactobacillus intra-abdominal infection in a renal transplant recipient with a history of probiotic consumption . Infection, 2016,44(6):793-796.

[1922] Vasquez E C, Aires R, Ton A, et al. New Insights on the Beneficial Effects of the Probiotic Kefir on Vascular Dysfunction in Cardiovascular and Neurodegenerative Diseases . Curr Pharm Des, 2020,26(30):3700-3710.

[1923] Vasquez E C, Pereira T, Peotta V A, et al. Probiotics as Beneficial Dietary Supplements to Prevent and Treat Cardiovascular Diseases: Uncovering Their Impact on Oxidative Stress . Oxid Med Cell Longev, 2019,2019:3086270.

[1924] Vaziri N D. Gut microbial translocation in the pathogenesis of systemic inflammation in patients with end-stage renal disease . Dig Dis Sci, 2014,59(9):2020-2022.

[1925] Velasquez M T. Altered Gut Microbiota: A Link Between Diet and the Metabolic Syndrome . Metab Syndr Relat Disord, 2018,16(7):321-328.

[1926] Velasquez M T, Centron P, Barrows I, et al. Gut Microbiota and Cardiovascular Uremic Toxicities . Toxins (Basel), 2018,10(7).

[1927] Velasquez M T, Ramezani A, Manal A, et al. Trimethylamine N-Oxide: The Good, the Bad and the Unknown . Toxins (Basel), 2016,8(11).

[1928] Vendrame S, Klimis-Zacas D. Potential Factors Influencing the Effects of Anthocyanins on Blood Pressure Regulation in Humans: A Review . Nutrients, 2019,11(6).

[1929] Venkatesh K K, Glover A V, Vladutiu C J, et al. Association of chorioamnionitis and its duration with adverse maternal outcomes by mode of delivery: a cohort study . BJOG, 2019,126(6):719-727.

[1930] Vercelli C, Łebkowska-Wieruszewska B, Barbero R, et al. Pharmacokinetics of levofloxacin in non-lactating goats and evaluation of drug effects on resistance in coliform rectal flora . Res Vet Sci, 2020,133:283-288.

[1931] Verhaar B, Collard D, Prodan A, et al. Associations between gut microbiota, faecal short-chain fatty acids, and blood pressure across ethnic groups: the HELIUS study . Eur Heart J, 2020,41(44):4259-4267.

[1932] Verhaar B, Prodan A, Nieuwdorp M, et al. Gut Microbiota in Hypertension and Atherosclerosis: A Review . Nutrients, 2020,12(10).

[1933] Viaene L, Thijs L, Jin Y, et al. Heritability and clinical determinants of serum indoxyl sulfate and p-cresyl sulfate, candidate biomarkers of the human microbiome enterotype . PLoS One, 2014,9(5):e79682.

[1934] Viana P O, Ono E, Dinelli M I, et al. Maternally acquired IgG immunity in neonates born to renal transplanted women . Vaccine, 2015,33(27):3104-3109.

[1935] Viasus D, Garcia-Vidal C, Castellote J, et al. Community-acquired pneumonia in patients with liver cirrhosis: clinical features, outcomes, and usefulness of severity scores . Medicine (Baltimore), 2011,90(2):110-118.

[1936] Viasus D, Simonetti A F, Garcia-Vidal C, et al. Impact of antibiotic de-escalation on clinical outcomes in community-acquired pneumococcal pneumonia . J Antimicrob Chemother, 2017,72(2):547-553.

[1937] Villamor E, Pérez V F, Tamargo J, et al. Effects of group B Streptococcus on the responses to U46619, endothelin-1, and noradrenaline in isolated pulmonary and mesenteric arteries of piglets . Pediatr Res, 1996,40(6):827-833.

[1938] Villapol S. Gastrointestinal symptoms associated with COVID-19: impact on the gut microbiome . Transl Res, 2020,226:57-69.

[1939] Violi A, Cambiè G, Miraglia C, et al. Epidemiology and risk factors for diverticular disease . Acta Biomed, 2018,89(9-S):107-112.

[1940] Virseda-Berdices A, Brochado-Kith O, Díez C, et al. Blood microbiome is associated with changes in portal hypertension after successful direct-acting antiviral therapy in patients with HCV-related cirrhosis . J Antimicrob Chemother, 2022,77(3):719-726.

[1941] Vishwanathan K, Bartlett M G, Stewart J T. Determination of gatifloxacin in human plasma by liquid chromatography/electrospray tandem mass spectrometry . Rapid Commun Mass Spectrom, 2001,15(12):915-919.

[1942] von Schnakenburg C, Hufnagel M, Superti-Furga A, et al. [Successful continuous renal replacement therapy in a neonate with early-onset group B streptococcal sepsis and multi-organ dysfunction syndrome] . Klin Padiatr, 2009,221(4):251-253.

[1943] Vrdoljak J, Kumric M, Vilovic M, et al. Can Fasting Curb the Metabolic Syndrome Epidemic? . Nutrients, 2022,14(3).

[1944] Waghulde H, Cheng X, Galla S, et al. Attenuation of Microbiotal Dysbiosis and Hypertension in a CRISPR/Cas9 Gene Ablation Rat Model of GPER1 . Hypertension, 2018,72(5):1125-1132.

[1945] Wagner J, Harrison E M, Martinez D P M, et al. The composition and functional protein subsystems of the human nasal microbiome in granulomatosis with polyangiitis: a pilot study . Microbiome, 2019,7(1):137.

[1946] Walejko J M, Kim S, Goel R, et al. Gut microbiota and serum metabolite differences in African Americans and White Americans with high blood pressure . Int J Cardiol, 2018,271:336-339.

[1947] Walker M Y, Pratap S, Southerland J H, et al. Role of oral and gut microbiome in nitric oxide-mediated colon motility . Nitric Oxide, 2018,73:81-88.

[1948] Walsh J A. Disease problems in the Third World . Ann N Y Acad Sci, 1989,569:1-16.

[1949] Walshe N, Cabrera-Rubio R, Collins R, et al. A Multiomic Approach to Investigate the Effects of a Weight Loss Program on the Intestinal Health of Overweight Horses . Front Vet Sci, 2021,8:668120.

[1950] Wan C, Zhu C, Jin G, et al. Analysis of Gut Microbiota in Patients with Coronary Artery Disease and Hypertension . Evid Based Complement Alternat Med, 2021,2021:7195082.

[1951] Wan Y, Jiang J, Lu M, et al. Human milk microbiota development during lactation and its relation to maternal geographic location and gestational hypertensive status . Gut Microbes, 2020,11(5):1438-1449.

[1952] Wang C Z, Kim K E, Du GJ, et al. Ultra-performance liquid chromatography and time-of-flight mass spectrometry analysis of ginsenoside metabolites in human plasma . Am J Chin Med, 2011,39(6):1161-1171.

[1953] Wang C, Lu H, Liu M, et al. Effective Antibacterial and Antihemolysin Activities of Ellipticine Hydrochloride against Streptococcus suis in a Mouse Model . Appl Environ Microbiol, 2021,87(10).

[1954] Wang F X, Zhu N, Zhou F, et al. Natural Aporphine Alkaloids with Potential to Impact Metabolic Syndrome . Molecules, 2021,26(20).

[1955] Wang G, Hao M, Liu Q, et al. Protective effect of recombinant Lactobacillus plantarum against H(2)O(2)-induced oxidative stress in HUVEC cells . J Zhejiang Univ Sci B, 2021,22(5):348-365.

[1956] Wang G, Zhu J, Liu L, et al. Optimization for galactooligosaccharides synthesis: A potential alternative for gut health and immunity . Life Sci, 2020,245:117353.

[1957] Wang J M, Yang M X, Wu Q F, et al. Improvement of intestinal flora: accompany with the antihypertensive effect of electroacupuncture on stage 1 hypertension . Chin Med, 2021,16(1):7.

[1958] Wang L, Zhu Q, Lu A, et al. Sodium butyrate suppresses angiotensin II-induced hypertension by inhibition of renal (pro)renin receptor and intrarenal renin-angiotensin system . J Hypertens, 2017,35(9):1899-1908.

[1959] Wang M, Zhang Y, Miller D, et al. Microbial Reconstitution Reverses Early Female Puberty Induced by Maternal High-fat Diet During Lactation . Endocrinology, 2020,161(2).

[1960] Wang N, Ma S, Fu L. Gut Microbiota Dysbiosis as One Cause of Osteoporosis by Impairing Intestinal Barrier Function . Calcif Tissue Int, 2022,110(2):225-235.

[1961] Wang P, Dong Y, Jiao J, et al. Cigarette smoking status alters dysbiotic gut microbes in hypertensive patients . J Clin Hypertens (Greenwich), 2021,23(7):1431-1446.

[1962] Wang P, Dong Y, Zuo K, et al. Characteristics and variation of fecal bacterial communities and functions in isolated systolic and diastolic hypertensive patients . BMC Microbiol, 2021,21(1):128.

[1963] Wang R, Li M, Strappe P, et al. Preparation, structural characteristics and physiological property of resistant starch . Adv Food Nutr Res, 2021,95:1-40.

[1964] Wang S, Zhang L, Wang D, et al. Gut Microbiota Composition is Associated with Responses to Peanut Intervention in Multiple Parameters Among Adults with Metabolic Syndrome Risk . Mol Nutr Food Res, 2021,65(18):e2001051.

[1965] Wang X, Chen Z, Geng B, et al. The Bidirectional Signal Communication of Microbiota-Gut-Brain Axis in Hypertension . Int J Hypertens, 2021,2021:8174789.

[1966] Wang X, Liu Y, Wang Y, et al. Protective Effect of Coriander (Coriandrum sativum L.) on High-Fructose and High-Salt Diet-Induced Hypertension: Relevant to Improvement of Renal and Intestinal Function . J Agric Food Chem, 2022,70(12):3730-3744.

[1967] Wang X, Ma L K, Song Y N, et al. [Rapid Group B streptococcus screening methods in late pregnancy and the maternal-neonatal outcomes] . Zhonghua Yi Xue Za Zhi, 2016,96(15):1188-1191.

[1968] Wang Y, Ames N P, Tun H M, et al. High Molecular Weight Barley β-Glucan Alters Gut Microbiota Toward Reduced Cardiovascular Disease Risk . Front Microbiol, 2016,7:129.

[1969] Wang Y, Wang H, Howard A G, et al. Gut Microbiota and Host Plasma Metabolites in Association with Blood Pressure in Chinese Adults . Hypertension, 2021,77(2):706-717.

[1970] Warensjö E, Jansson J H, Cederholm T, et al. Biomarkers of milk fat and the risk of myocardial infarction in men and women: a prospective, matched case-control study . Am J Clin Nutr, 2010,92(1):194-202.

[1971] Watanabe H, Uruma T, Tazaki G, et al. Clinical factors associated with negative urinary antigen tests implemented for the diagnosis of community-acquired pneumococcal pneumonia in adult patients . Med Princ Pract, 2015,24(2):189-194.

[1972] Watanabe I, Okubo J. Function of the eustachian tube in an alternobaric environment. A study with special reference to the therapy of secretory otitis media in children . Acta Otolaryngol, 1987,103(5-6):387-394.

[1973] Watanabe M, Kurihara J, Suzuki S, et al. The influence of dietary peptide inhibitors of angiotensin-converting enzyme on the hypotensive effects of enalapril . J Pharm Health Care Sci, 2015,1:17.

[1974] Wawrzyniak N, Skrypnik K, Suliburska J. Dietary supplements in therapy to support weight reduction in obese patients . Acta Sci Pol Technol Aliment, 2022,21(1):67-80.

[1975] Weber C, Luehr M, Petrov G, et al. Impact of the 2009 ESC Guideline Change on Surgically Treated Infective Endocarditis . Ann Thorac Surg, 2022.

[1976] Weber G J, Pushpakumar S, Tyagi S C, et al. Homocysteine and hydrogen sulfide in epigenetic, metabolic and microbiota related renovascular hypertension . Pharmacol Res, 2016,113(Pt A):300-312.

[1977] Wedgwood S, Gerard K, Halloran K, et al. Intestinal Dysbiosis and the Developing Lung: The Role of Toll-Like Receptor 4 in the Gut-Lung Axis . Front Immunol, 2020,11:357.

[1978] Wedgwood S, Warford C, Agvatisiri S R, et al. The developing gut-lung axis: postnatal growth restriction, intestinal dysbiosis, and pulmonary hypertension in a rodent model . Pediatr Res, 2020,87(3):472-479.

[1979] Weeks J W, Myers S R, Lasher L, et al. Persistence of penicillin G benzathine in pregnant group B streptococcus carriers . Obstet Gynecol, 1997,90(2):240-243.

[1980] Weiss K, Low D E, Cortes L, et al. Clinical characteristics at initial presentation and impact of dual therapy on the outcome of bacteremic Streptococcus pneumoniae pneumonia in adults . Can Respir J, 2004,11(8):589-593.

[1981] Wellbelove Z, Walsh C, Barlow G D, et al. Comparing scoring systems for prediction of mortality in patients with bloodstream infection . QJM, 2021,114(2):105-110.

[1982] Wenzel U, Turner J E, Krebs C, et al. Immune Mechanisms in Arterial Hypertension . J Am Soc Nephrol, 2016,27(3):677-686.

[1983] Werny L, Colmorgen C, Becker-Pauly C. Regulation of meprin metalloproteases in mucosal homeostasis . Biochim Biophys Acta Mol Cell Res, 2022,1869(1):119158.

[1984] Weschenfelder C, Schaan D Q A, Lorenzon D S J, et al. Adipokines and Adipose Tissue-Related Metabolites, Nuts and Cardiovascular Disease . Metabolites, 2020,10(1).

[1985] Westling K, Julander I, Ljungman P, et al. Viridans group streptococci in blood culture isolates in a Swedish university hospital: antibiotic susceptibility and identification of erythromycin resistance genes . Int J Antimicrob Agents, 2006,28(4):292-296.

[1986] White A V, Hoy W E, McCredie D A. Childhood post-streptococcal glomerulonephritis as a risk factor for chronic renal disease in later life . Med J Aust, 2001,174(10):492-496.

[1987] Wicaksono D P, Washio J, Abiko Y, et al. Nitrite Production from Nitrate and Its Link with Lactate Metabolism in Oral Veillonella spp . Appl Environ Microbiol, 2020,86(20).

[1988] Widyarman A S, Theodorea C F, Udawatte N S, et al. Diversity of Oral Microbiome of Women From Urban and Rural Areas of Indonesia: A Pilot Study . Front Oral Health, 2021,2:738306.

[1989] Wiest R, Chen F, Cadelina G, et al. Effect of Lactobacillus-fermented diets on bacterial translocation and intestinal flora in experimental prehepatic portal hypertension . Dig Dis Sci, 2003,48(6):1136-1141.

[1990] Wilck N. What's for dinner? Why a close look at diet and microbiota is worthwhile in experimental hypertension research . Acta Physiol (Oxf), 2021,232(4):e13704.

[1991] Wilck N, Matus M G, Kearney S M, et al. Salt-responsive gut commensal modulates T(H)17 axis and disease . Nature, 2017,551(7682):585-589.

[1992] Wild J, Wenzel P. Myeloid cells, tissue homeostasis, and anatomical barriers as innate immune effectors in arterial hypertension . J Mol Med (Berl), 2021,99(3):315-326.

[1993] Wilde C G, Griffith J E, Marra M N, et al. Purification and characterization of human neutrophil peptide 4, a novel member of the defensin family . J Biol Chem, 1989,264(19):11200-11203.

[1994] Williamson G. The role of polyphenols in modern nutrition . Nutr Bull, 2017,42(3):226-235.

[1995] Willmott T, McBain A J, Humphreys G J, et al. Does the Oral Microbiome Play a Role in Hypertensive Pregnancies? . Front Cell Infect Microbiol, 2020,10:389.

[1996] Wilson R, Willis J, Gearry R B, et al. SunGold Kiwifruit Supplementation of Individuals with Prediabetes Alters Gut Microbiota and Improves Vitamin C Status, Anthropometric and Clinical Markers . Nutrients, 2018,10(7).

[1997] Wilson S. A healthy 47-year-old man dies of group A Streptococcus . J Emerg Nurs, 2009,35(5):442-444.

[1998] Winkelstein J A, Lambert G H, Swift A. Pneumococcal serum opsonizing activity in splenectomized children . J Pediatr, 1975,87(3):430-433.

[1999] Winrow A P. Successful treatment of neonatal purpura fulminans with epoprostenol . J R Soc Med, 1992,85(4):245.

[2000] Winther L, Guardabassi L, Baptiste K E, et al. Antimicrobial disposition in pulmonary epithelial lining fluid of horses. Part I. Sulfadiazine and trimethoprim . J Vet Pharmacol Ther, 2011,34(3):277-284.

[2001] Winther L, Honoré H S, Baptiste K E, et al. Antimicrobial disposition in pulmonary epithelial lining fluid of horses, part II. Doxycycline . J Vet Pharmacol Ther, 2011,34(3):285-289.

[2002] Witkin S S, Gravett M G, Haluska G J, et al. Induction of interleukin-1 receptor antagonist in rhesus monkeys after intraamniotic infection with group B streptococci or interleukin-1 infusion . Am J Obstet Gynecol, 1994,171(6):1668-1672.

[2003] Witte T S, Bergwerff A A, Scherpenisse P, et al. Ceftiofur derivates in serum and endometrial tissue after intramuscular administration in healthy mares . Theriogenology, 2010,74(3):466-472.

[2004] Witzenrath M, Gutbier B, Hocke A C, et al. Role of pneumolysin for the development of acute lung injury in pneumococcal pneumonia . Crit Care Med, 2006,34(7):1947-1954.

[2005] Witzenrath M, Gutbier B, Owen J S, et al. Role of platelet-activating factor in pneumolysin-induced acute lung injury . Crit Care Med, 2007,35(7):1756-1762.

[2006] Włodarczyk M, Śliżewska K. Efficiency of Resistant Starch and Dextrins as Prebiotics: A Review of the Existing Evidence and Clinical Trials . Nutrients, 2021,13(11).

[2007] Wolfschoon-Pombo A F, Klostermeyer H. Indoxylsulfate in milk . Z Lebensm Unters Forsch, 1986,182(2):103-106.

[2008] Wölnerhanssen B K, Meyer-Gerspach A C. [Health effects of sugar consumption and possible alternatives] . Ther Umsch, 2019,76(3):111-116.

[2009] Wong J, Piceno Y M, DeSantis T Z, et al. Expansion of urease- and uricase-containing, indole- and p-cresol-forming and contraction of short-chain fatty acid-producing intestinal microbiota in ESRD . Am J Nephrol, 2014,39(3):230-237.

[2010] Wouk N. End-Stage Renal Disease: Medical Management . Am Fam Physician, 2021,104(5):493-499.

[2011] Wright-Pascoe R, Roye-Green K, Bodonaik N. The medical management of diabetes mellitus with particular reference to the lower extremity: the Jamaican experience . West Indian Med J, 2001,50 Suppl 1:46-49.

[2012] Wu D, Ding L, Tang X, et al. Baicalin Protects Against Hypertension-Associated Intestinal Barrier Impairment in Part Through Enhanced Microbial Production of Short-Chain Fatty Acids . Front Pharmacol, 2019,10:1271.

[2013] Wu D, Tang X, Ding L, et al. Candesartan attenuates hypertension-associated pathophysiological alterations in the gut . Biomed Pharmacother, 2019,116:109040.

[2014] Wu G, Zheng Y, Zhou H, et al. Safety and pharmacokinetics of dicloxacillin in healthy Chinese volunteers following single and multiple oral doses . Drug Des Devel Ther, 2015,9:5687-5695.

[2015] Wu H, Lam T, Shum T F, et al. Hypotensive effect of captopril on deoxycorticosterone acetate-salt-induced hypertensive rat is associated with gut microbiota alteration . Hypertens Res, 2022,45(2):270-282.

[2016] Wu H, Rui X, Li W, et al. Whole-grain oats (Avena sativa L.) as a carrier of lactic acid bacteria and a supplement rich in angiotensin I-converting enzyme inhibitory peptides through solid-state fermentation . Food Funct, 2018,9(4):2270-2281.

[2017] Wu I H, Tsai M H, Lai M Y, et al. Incidence, clinical features, and implications on outcomes of neonatal late-onset sepsis with concurrent infectious focus . BMC Infect Dis, 2017,17(1):465.

[2018] Wu M C, Jan M S, Chiou J Y, et al. Constipation might be associated with risk of allergic rhinitis: A nationwide population-based cohort study . PLoS One, 2020,15(10):e239723.

[2019] Wu Q, Luo F, Wang X L, et al. Angiotensin I-converting enzyme inhibitory peptide: an emerging candidate for vascular dysfunction therapy . Crit Rev Biotechnol, 2021:1-20.

[2020] Wu Q, Shah N P. High γ-aminobutyric acid production from lactic acid bacteria: Emphasis on Lactobacillus brevis as a functional dairy starter . Crit Rev Food Sci Nutr, 2017,57(17):3661-3672.

[2021] Wu Q, Xu Z, Song S, et al. Gut microbiota modulates stress-induced hypertension through the HPA axis . Brain Res Bull, 2020,162:49-58.

[2022] Wu Q, Zhao Y, Zhang X, et al. A faster and simpler UPLC-MS/MS method for the simultaneous determination of trimethylamine N-oxide, trimethylamine and dimethylamine in different types of biological samples . Food Funct, 2019,10(10):6484-6491.

[2023] Wu S I, Wu C C, Tsai P J, et al. Psychobiotic Supplementation of PS128(TM) Improves Stress, Anxiety, and Insomnia in Highly Stressed Information Technology Specialists: A Pilot Study . Front Nutr, 2021,8:614105.

[2024] Wu W Y, Chou P L, Yang J C, et al. Silicon-containing water intake confers antioxidant effect, gastrointestinal protection, and gut microbiota modulation in the rodents . PLoS One, 2021,16(3):e248508.

[2025] Wu Y, He H, Cheng Z, et al. The Role of Neuropeptide Y and Peptide YY in the Development of Obesity via Gut-brain Axis . Curr Protein Pept Sci, 2019,20(7):750-758.

[2026] Wu Y, Xu H, Tu X, et al. The Role of Short-Chain Fatty Acids of Gut Microbiota Origin in Hypertension . Front Microbiol, 2021,12:730809.

[2027] Wu Z, Chen Y, Xiao T, et al. Epidemiology and risk factors of infective endocarditis in a tertiary hospital in China from 2007 to 2016 . BMC Infect Dis, 2020,20(1):428.

[2028] Wu Z, Pan D, Zhen X, et al. Angiotensin I-converting enzyme inhibitory peptides derived from bovine casein and identified by MALDI-TOF-MS/MS . J Sci Food Agric, 2013,93(6):1331-1337.

[2029] Wuerzner G, Peyrard S, Blanchard A, et al. The lactotripeptides isoleucine-proline-proline and valine-proline-proline do not inhibit the N-terminal or C-terminal angiotensin converting enzyme active sites in humans . J Hypertens, 2009,27(7):1404-1409.

[2030] Wyatt C M, Crowley S D. Intersection of salt- and immune-mediated mechanisms of hypertension in the gut microbiome . Kidney Int, 2018,93(3):532-534.

[2031] Xia W J, Xu M L, Yu X J, et al. Antihypertensive effects of exercise involve reshaping of gut microbiota and improvement of gut-brain axis in spontaneously hypertensive rat . Gut Microbes, 2021,13(1):1-24.

[2032] Xia Y, Yu J, Xu W, et al. Purification and characterization of angiotensin-I-converting enzyme inhibitory peptides isolated from whey proteins of milk fermented with Lactobacillus plantarum QS670 . J Dairy Sci, 2020,103(6):4919-4928.

[2033] Xiao J, Fogarty C, Wu T T, et al. Oral health and Candida carriage in socioeconomically disadvantaged US pregnant women . BMC Pregnancy Childbirth, 2019,19(1):480.

[2034] Xiao S, Fei N, Pang X, et al. A gut microbiota-targeted dietary intervention for amelioration of chronic inflammation underlying metabolic syndrome . FEMS Microbiol Ecol, 2014,87(2):357-367.

[2035] Xiao T, Yan A, Huang J D, et al. Comparative Peptidomic and Metatranscriptomic Analyses Reveal Improved Gamma-Amino Butyric Acid Production Machinery in Levilactobacillus brevis Strain NPS-QW 145 Cocultured with Streptococcus thermophilus Strain ASCC1275 during Milk Fermentation . Appl Environ Microbiol, 2020,87(1).

[2036] Xie G, Wang Y, Wang X, et al. Profiling of serum bile acids in a healthy Chinese population using UPLC-MS/MS . J Proteome Res, 2015,14(2):850-859.

[2037] Xiong S, Li Q, Liu D, et al. Gastrointestinal Tract: a Promising Target for the Management of Hypertension . Curr Hypertens Rep, 2017,19(4):31.

[2038] Xiong Y, Xiong Y, Zhu P, et al. The Role of Gut Microbiota in Hypertension Pathogenesis and the Efficacy of Antihypertensive Drugs . Curr Hypertens Rep, 2021,23(8):40.

[2039] Xu A A, Hoffman K, Gurwara S, et al. Oral Health and the Altered Colonic Mucosa-Associated Gut Microbiota . Dig Dis Sci, 2021,66(9):2981-2991.

[2040] Xu A, Hyman D, Lu L B. Cefazolin-Related Acute Interstitial Nephritis with Associated Nephrotic-Range Proteinuria: A Case Report . Drug Saf Case Rep, 2018,5(1):16.

[2041] Xu B, Fu J, Qiao Y, et al. Higher intake of microbiota-accessible carbohydrates and improved cardiometabolic risk factors: a meta-analysis and umbrella review of dietary management in patients with type 2 diabetes . Am J Clin Nutr, 2021,113(6):1515-1530.

[2042] Xu H, Wang H, Guan J, et al. Effects of continuous positive airway pressure on neurocognitive architecture and function in patients with obstructive sleep apnoea: study protocol for a multicentre randomised controlled trial . BMJ Open, 2017,7(5):e14932.

[2043] Xu H, Xiong M, Huang Q. [The study on COPD rat model produced by bacterial infection] . Zhonghua Jie He He Hu Xi Za Zhi, 1999,22(12):739-742.

[2044] Xu J, Yang Y. Gut microbiome and its meta-omics perspectives: profound implications for cardiovascular diseases . Gut Microbes, 2021,13(1):1936379.

[2045] Xu P, Li M, Zhang J, et al. Correlation of intestinal microbiota with overweight and obesity in Kazakh school children . BMC Microbiol, 2012,12:283.

[2046] Xu X, Hicks C, Li Y, et al. Purified cell wall from the probiotic bacterium Lactobacillus gasseri activates systemic inflammation and, at higher doses, produces lethality in a rat model . Crit Care, 2014,18(4):R140.

[2047] Xue J, Chi L, Tu P, et al. Detection of gut microbiota and pathogen produced N-acyl homoserine in host circulation and tissues . NPJ Biofilms Microbiomes, 2021,7(1):53.

[2048] Yale C E. Experimental strangulated intestinal obstruction . Surgery, 1969,66(2):338-344.

[2049] Yamagishi Y, Mikamo H. A retrospective study of health care-associated pneumonia patients at Aichi Medical University hospital . J Infect Chemother, 2011,17(6):756-763.

[2050] Yamamoto N. Antihypertensive peptides derived from food proteins . Biopolymers, 1997,43(2):129-134.

[2051] Yamamoto N, Akino A, Takano T. Antihypertensive effect of the peptides derived from casein by an extracellular proteinase from Lactobacillus helveticus CP790 . J Dairy Sci, 1994,77(4):917-922.

[2052] Yamamoto N, Ejiri M, Mizuno S. Biogenic peptides and their potential use . Curr Pharm Des, 2003,9(16):1345-1355.

[2053] Yamamoto N, Maeno M, Takano T. Purification and characterization of an antihypertensive peptide from a yogurt-like product fermented by Lactobacillus helveticus CPN4 . J Dairy Sci, 1999,82(7):1388-1393.

[2054] Yamamoto N, Takano T. Antihypertensive peptides derived from milk proteins . Nahrung, 1999,43(3):159-164.

[2055] Yamasaki-Yashiki S, Tachibana S, Asano Y. Determination of L-methionine using methionine-specific dehydrogenase for diagnosis of homocystinuria due to cystathionine β-synthase deficiency . Anal Biochem, 2012,428(2):143-149.

[2056] Yamashiro E, Asato Y, Taira K, et al. Necrotizing fasciitis caused by Streptococcus pneumoniae . J Dermatol, 2009,36(5):298-305.

[2057] Yamashiro K, Tanaka R, Urabe T, et al. Gut dysbiosis is associated with metabolism and systemic inflammation in patients with ischemic stroke . PLoS One, 2017,12(2):e171521.

[2058] Yan C, Zhang C, Cao X, et al. Intestinal Population in Host with Metabolic Syndrome during Administration of Chitosan and Its Derivatives . Molecules, 2020,25(24).

[2059] Yan L, Wang M, Chen J, et al. Effects of gut microbiome-targeted therapies on cardiometabolic outcomes in children and adolescents: A protocol for systematic review and meta-analysis . Medicine (Baltimore), 2020,99(31):e21612.

[2060] Yan Q, Gu Y, Li X, et al. Alterations of the Gut Microbiome in Hypertension . Front Cell Infect Microbiol, 2017,7:381.

[2061] Yan Q, Zhai W, Yang C, et al. The Relationship among Physical Activity, Intestinal Flora, and Cardiovascular Disease . Cardiovasc Ther, 2021,2021:3364418.

[2062] Yan X, Jin J, Su X, et al. Intestinal Flora Modulates Blood Pressure by Regulating the Synthesis of Intestinal-Derived Corticosterone in High Salt-Induced Hypertension . Circ Res, 2020,126(7):839-853.

[2063] Yang F, Chen H, Gao Y, et al. Gut microbiota-derived short-chain fatty acids and hypertension: Mechanism and treatment . Biomed Pharmacother, 2020,130:110503.

[2064] Yang G, He H Q, Chen G, et al. [Effect of traditional Chinese medicine in attenuating coronary heart disease and main risk factors by regulating gut micro-biota] . Zhongguo Zhong Yao Za Zhi, 2020,45(1):29-36.

[2065] Yang G, Jiang Y, Yang W, et al. Effective treatment of hypertension by recombinant Lactobacillus plantarum expressing angiotensin converting enzyme inhibitory peptide . Microb Cell Fact, 2015,14:202.

[2066] Yang J J, Shu X O, Herrington D M, et al. Circulating trimethylamine N-oxide in association with diet and cardiometabolic biomarkers: an international pooled analysis . Am J Clin Nutr, 2021,113(5):1145-1156.

[2067] Yang K T, Juang S E, Hung Y M, et al. The association between bowel resection and the risk of nontyphoidal salmonella infection: a nationwide propensity score-matched cohort study . Sci Rep, 2021,11(1):1414.

[2068] Yang M, Lao L. Emerging Applications of Metabolomics in Traditional Chinese Medicine Treating Hypertension: Biomarkers, Pathways and More . Front Pharmacol, 2019,10:158.

[2069] Yang M, Yu Z, Chen X, et al. Active Acupoints Differ from Inactive Acupoints in Modulating Key Plasmatic Metabolites of Hypertension: A Targeted Metabolomics Study . Sci Rep, 2018,8(1):17824.

[2070] Yang S, Ye K. Recent advances in understanding the adaptive evolution of metabolic genes and traits . Curr Opin Clin Nutr Metab Care, 2021,24(4):308-314.

[2071] Yang T. Komagataella pastoris: A new yeast probiotic for depression? . Pharmacol Res, 2021,171:105762.

[2072] Yang T, Aquino V, Lobaton G O, et al. Sustained Captopril-Induced Reduction in Blood Pressure Is Associated With Alterations in Gut-Brain Axis in the Spontaneously Hypertensive Rat . J Am Heart Assoc, 2019,8(4):e10721.

[2073] Yang T, Chakraborty S, Mandal J, et al. Microbiota and Metabolites as Factors Influencing Blood Pressure Regulation . Compr Physiol, 2021,11(2):1731-1757.

[2074] Yang T, Santisteban M M, Rodriguez V, et al. Gut dysbiosis is linked to hypertension . Hypertension, 2015,65(6):1331-1340.

[2075] Yang T, Zubcevic J. Gut-Brain Axis in Regulation of Blood Pressure . Front Physiol, 2017,8:845.

[2076] Yang W, Shao L, Heizhati M, et al. Oropharyngeal Microbiome in Obstructive Sleep Apnea: Decreased Diversity and Abundance . J Clin Sleep Med, 2019,15(12):1777-1788.

[2077] Yang Y H, Ngo C, Yeh I N, et al. Antibody Fc functional activity of intravenous immunoglobulin preparations treated with solvent-detergent for virus inactivation . Vox Sang, 1994,67(4):337-344.

[2078] Yang Y, Zheng L, Wang L, et al. Effects of high fructose and salt feeding on systematic metabonome probed via (1) H NMR spectroscopy . Magn Reson Chem, 2015,53(4):295-303.

[2079] Yano Y. Blood pressure management in an ecosystem context . Hypertens Res, 2020,43(10):989-994.

[2080] Yano Y, Niiranen T J. Gut Microbiome over a Lifetime and the Association with Hypertension . Curr Hypertens Rep, 2021,23(3):15.

[2081] Yao K, Wang S, Gaowa N, et al. Identification of the molecular mechanisms underlying brisket disease in Holstein heifers via microbiota and metabolome analyses . AMB Express, 2021,11(1):86.

[2082] Yao L, Seaton S C, Ndousse-Fetter S, et al. A selective gut bacterial bile salt hydrolase alters host metabolism . Elife, 2018,7.

[2083] Yao S, Yagi S, Uozumi R, et al. A High Portal Venous Pressure Gradient Increases Gut-Related Bacteremia and Consequent Early Mortality After Living Donor Liver Transplantation . Transplantation, 2018,102(4):623-631.

[2084] Yao Z, Zhao M, Gong Y, et al. Relation of Gut Microbes and L-Thyroxine Through Altered Thyroxine Metabolism in Subclinical Hypothyroidism Subjects . Front Cell Infect Microbiol, 2020,10:495.

[2085] Yeo L F, Aghakhanian F F, Tan J, et al. Health and saliva microbiomes of a semi-urbanized indigenous tribe in Peninsular Malaysia . F1000Res, 2019,8:175.

[2086] Yeo S K, Liong M T. Angiotensin I-converting enzyme inhibitory activity and bioconversion of isoflavones by probiotics in soymilk supplemented with prebiotics . Int J Food Sci Nutr, 2010,61(2):161-181.

[2087] Yi N Y, Davis J L, Salmon J H, et al. Ocular distribution and toxicity of intravitreal injection of triamcinolone acetonide in normal equine eyes . Vet Ophthalmol, 2008,11 Suppl 1:15-19.

[2088] Yi Y, Du L, Qin M, et al. Regulation of Atrial Fibrosis by the Bone . Hypertension, 2019,73(2):379-389.

[2089] Yilmaz B, Portugal S, Tran T M, et al. Gut microbiota elicits a protective immune response against malaria transmission . Cell, 2014,159(6):1277-1289.

[2090] Yokoyama K, Tsuchiya N, Yamauchi R, et al. Exploratory Research on the Relationship between Human Gut Microbiota and Portal Hypertension . Intern Med, 2020,59(17):2089-2094.

[2091] Yoo H H, Kim I S, Yoo D H, et al. Effects of orally administered antibiotics on the bioavailability of amlodipine: gut microbiota-mediated drug interaction . J Hypertens, 2016,34(1):156-162.

[2092] Yoo K H, Yoo C G, Kim S K, et al. Economic burden and epidemiology of pneumonia in Korean adults aged over 50 years . J Korean Med Sci, 2013,28(6):888-895.

[2093] Yoshizawa N. Acute glomerulonephritis . Intern Med, 2000,39(9):687-694.

[2094] Yost R L, Gotz V P. Effect of a lactobacillus preparation on the absorption of oral ampicillin . Antimicrob Agents Chemother, 1985,28(6):727-729.

[2095] Younes R, Bugianesi E. NASH in Lean Individuals . Semin Liver Dis, 2019,39(1):86-95.

[2096] Yu B, Wang J. The efficacy of parenteral nutrition (PN) and enteral nutrition (EN) supports in cirrhosis: A systematic review and network meta-analysis . Medicine (Baltimore), 2022,101(3):e28618.

[2097] Yu D, Nguyen S M, Yang Y, et al. Long-term diet quality is associated with gut microbiome diversity and composition among urban Chinese adults . Am J Clin Nutr, 2021,113(3):684-694.

[2098] Yu D, Yang Y, Long J, et al. Long-term Diet Quality and Gut Microbiome Functionality: A Prospective, Shotgun Metagenomic Study among Urban Chinese Adults . Curr Dev Nutr, 2021,5(4):b26.

[2099] Yu H W, Lin H C, Yang P H, et al. Group B streptococcal infection in Taiwan: maternal colonization and neonatal infection . Pediatr Neonatol, 2011,52(4):190-195.

[2100] Yu P H. Determination of plasma pyridoxal 5'-phosphate by an enzymatic-high-performance liquid chromatographic procedure . Anal Biochem, 1989,181(2):267-270.

[2101] Yu X, Zhang X, Jin H, et al. Zhengganxifeng Decoction Affects Gut Microbiota and Reduces Blood Pressure via Renin-Angiotensin System . Biol Pharm Bull, 2019,42(9):1482-1490.

[2102] Yu Y, Hong X. NF-kB Expression, Comparison of Intestinal Flora, Inflammation and Adiponectin and Clinical Significance in Prostate Cancer Patients . Clin Lab, 2021,67(8).

[2103] Yu Y, Mao G, Wang J, et al. Gut dysbiosis is associated with the reduced exercise capacity of elderly patients with hypertension . Hypertens Res, 2018,41(12):1036-1044.

[2104] Yudin M H, Caprara D, MacGillivray S J, et al. A Ten-Year Review of Antenatal Complications and Pregnancy Outcomes Among HIV-Positive Pregnant Women . J Obstet Gynaecol Can, 2016,38(1):35-40.

[2105] Yutsudo T, Murai H, Gonzalez J, et al. A new type of mitogenic factor produced by Streptococcus pyogenes . FEBS Lett, 1992,308(1):30-34.

[2106] Zachariassen G, Hyldig N, Joergensen J S, et al. The half-life and exposure of cefuroxime varied in newborn infants after a Caesarean section . Acta Paediatr, 2016,105(9):1074-1078.

[2107] Zaitsu M, Inada Y, Tashiro K, et al. Acute alcohol intoxication in a 15-day-old neonate . Pediatr Int, 2013,55(6):792-794.

[2108] Zak-Gołąb A, Olszanecka-Glinianowicz M, Kocełak P, et al. [The role of gut microbiota in the pathogenesis of obesity] . Postepy Hig Med Dosw (Online), 2014,68:84-90.

[2109] Zar H J, Apolles P, Argent A, et al. The etiology and outcome of pneumonia in human immunodeficiency virus-infected children admitted to intensive care in a developing country . Pediatr Crit Care Med, 2001,2(2):108-112.

[2110] Zeng C, Tan H. Gut Microbiota and Heart, Vascular Injury . Adv Exp Med Biol, 2020,1238:107-141.

[2111] Zeng Q, Li D, He Y, et al. Discrepant gut microbiota markers for the classification of obesity-related metabolic abnormalities . Sci Rep, 2019,9(1):13424.

[2112] Zeng Y M, Hu A K, Su H Z, et al. A review of the association between oral bacterial flora and obstructive sleep apnea-hypopnea syndrome comorbid with cardiovascular disease . Sleep Breath, 2020,24(4):1261-1266.

[2113] Zhang C, Zhang T, Lu W, et al. Altered Airway Microbiota Composition in Patients With Pulmonary Hypertension . Hypertension, 2020,76(5):1589-1599.

[2114] Zhang G X, Jin L, Jin H, et al. Influence of Dietary Components and Traditional Chinese Medicine on Hypertension: A Potential Role for Gut Microbiota . Evid Based Complement Alternat Med, 2021,2021:5563073.

[2115] Zhang J, Tang Q, Zhu L. Could the Gut Microbiota Serve as a Therapeutic Target in Ischemic Stroke? . Evid Based Complement Alternat Med, 2021,2021:1391384.

[2116] Zhang J, Zuo K, Fang C, et al. Altered synthesis of genes associated with short-chain fatty acids in the gut of patients with atrial fibrillation . BMC Genomics, 2021,22(1):634.

[2117] Zhang L H, Fang L G, Yang J, et al. [Infective endocarditis in patients with hypertrophic obstructive cardiomyopathy: five cases report] . Zhonghua Xin Xue Guan Bing Za Zhi, 2012,40(3):209-213.

[2118] Zhang L, Ko C Y, Zeng Y M. Immunoregulatory Effect of Short-Chain Fatty Acids from Gut Microbiota on Obstructive Sleep Apnea-Associated Hypertension . Nat Sci Sleep, 2022,14:393-405.

[2119] Zhang M W, Fan B S, Yu J G. Case Report: Miles Surgery Ameliorates High Blood Pressure in a Rectal Carcinoma Patient With Essential Hypertension . Front Cardiovasc Med, 2021,8:762959.

[2120] Zhang M, Sternberg M R, Pfeiffer C M. Harmonizing the Calibrator and Microorganism Used in the Folate Microbiological Assay Increases the Comparability of Serum and Whole-Blood Folate Results in a CDC Round-Robin Study . J Nutr, 2018,148(5):807-817.

[2121] Zhang W Q, Wang Y J, Zhang A, et al. TMA/TMAO in Hypertension: Novel Horizons and Potential Therapies . J Cardiovasc Transl Res, 2021,14(6):1117-1124.

[2122] Zhang W, Hartmann R, Tun H M, et al. Deletion of the Toll-Like Receptor 5 Gene Per Se Does Not Determine the Gut Microbiome Profile That Induces Metabolic Syndrome: Environment Trumps Genotype . PLoS One, 2016,11(3):e150943.

[2123] Zhang X, Sandhu A, Edirisinghe I, et al. An exploratory study of red raspberry (Rubus idaeus L.) (poly)phenols/metabolites in human biological samples . Food Funct, 2018,9(2):806-818.

[2124] Zhang X, Zhai Q, Wang J, et al. Variation of the Vaginal Microbiome During and after Pregnancy in Chinese Women . Genomics Proteomics Bioinformatics, 2022.

[2125] Zhang Y, Kumarasamy S, Mell B, et al. Vertical selection for nuclear and mitochondrial genomes shapes gut microbiota and modifies risks for complex diseases . Physiol Genomics, 2020,52(1):1-14.

[2126] Zhang Y, Li X, Sun Y, et al. Pharmacokinetics of S-epacadostat, an indoleamine 2,3-dioxygenase 1 inhibitor, in dog plasma and identification of its metabolites in vivo and in vitro . Biomed Chromatogr, 2021,35(12):e5226.

[2127] Zhang Z, Xu H, Zhao H, et al. Edgeworthia gardneri (Wall.) Meisn. water extract improves diabetes and modulates gut microbiota . J Ethnopharmacol, 2019,239:111854.

[2128] Zhang Z, Zhao J, Tian C, et al. Targeting the Gut Microbiota to Investigate the Mechanism of Lactulose in Negating the Effects of a High-Salt Diet on Hypertension . Mol Nutr Food Res, 2019,63(11):e1800941.

[2129] Zhao E, Wang D, Li N, et al. Clinical study on the diagnosis of porcine streptococcal meningitis with negative blood and cerebrospinal fluid culture by next-generation sequencing . Eur J Med Res, 2021,26(1):85.

[2130] Zhao H B, Jia L, Yan Q Q, et al. Effect of Clostridium butyricum and Butyrate on Intestinal Barrier Functions: Study of a Rat Model of Severe Acute Pancreatitis With Intra-Abdominal Hypertension . Front Physiol, 2020,11:561061.

[2131] Zhao H, Lu Z, Lu Y. The potential of probiotics in the amelioration of hyperuricemia . Food Funct, 2022,13(5):2394-2414.

[2132] Zhao J, Liu S, Yan J, et al. The Impact of Gut Microbiota on Post-Stroke Management . Front Cell Infect Microbiol, 2021,11:724376.

[2133] Zhao L, Lou H, Peng Y, et al. Comprehensive relationships between gut microbiome and faecal metabolome in individuals with type 2 diabetes and its complications . Endocrine, 2019,66(3):526-537.

[2134] Zhao X C, Zhao L, Sun X Y, et al. Excellent response of severe aplastic anemia to treatment of gut inflammation: A case report and review of the literature . World J Clin Cases, 2020,8(2):425-435.

[2135] Zhao Y Y, Tang D D, Chen H, et al. Urinary metabolomics and biomarkers of aristolochic acid nephrotoxicity by UPLC-QTOF/HDMS . Bioanalysis, 2015,7(6):685-700.

[2136] Zhao Z, Guo Z, Yin Z, et al. Gut Microbiota Was Involved in the Process of Liver Injury During Intra-Abdominal Hypertension . Front Physiol, 2021,12:790182.

[2137] Zheng S, Piao C, Liu Y, et al. Glycan Biosynthesis Ability of Gut Microbiota Increased in Primary Hypertension Patients Taking Antihypertension Medications and Potentially Promoted by Macrophage-Adenosine Monophosphate-Activated Protein Kinase . Front Microbiol, 2021,12:719599.

[2138] Zhong H J, Zeng H L, Cai Y L, et al. Washed Microbiota Transplantation Lowers Blood Pressure in Patients With Hypertension . Front Cell Infect Microbiol, 2021,11:679624.

[2139] Zhong L Y, Deng X Y, Huang Y, et al. [Pharmacodynamics and intestinal flora research on different processed products of Puerariae Lobatae Radix and Puerariae Thomsonii Radix] . Zhongguo Zhong Yao Za Zhi, 2021,46(17):4403-4409.

[2140] Zhou D D, Luo M, Shang A, et al. Antioxidant Food Components for the Prevention and Treatment of Cardiovascular Diseases: Effects, Mechanisms, and Clinical Studies . Oxid Med Cell Longev, 2021,2021:6627355.

[2141] Zhou J, Chen S, Ren J, et al. Association of enhanced circulating trimethylamine N-oxide with vascular endothelial dysfunction in periodontitis patients . J Periodontol, 2021.

[2142] Zhou P, Xie W, He S, et al. Ginsenoside Rb1 as an Anti-Diabetic Agent and Its Underlying Mechanism Analysis . Cells, 2019,8(3).

[2143] Zhou Q, Pang G, Zhang Z, et al. Association Between Gut Akkermansia and Metabolic Syndrome is Dose-Dependent and Affected by Microbial Interactions: A Cross-Sectional Study . Diabetes Metab Syndr Obes, 2021,14:2177-2188.

[2144] Zhou W, Cheng Y, Zhu P, et al. Implication of Gut Microbiota in Cardiovascular Diseases . Oxid Med Cell Longev, 2020,2020:5394096.

[2145] Zhou X, Li J, Guo J, et al. Gut-dependent microbial translocation induces inflammation and cardiovascular events after ST-elevation myocardial infarction . Microbiome, 2018,6(1):66.

[2146] Zhu L L, Ma Z J, Ren M, et al. Distinct Features of Gut Microbiota in High-Altitude Tibetan and Middle-Altitude Han Hypertensive Patients . Cardiol Res Pract, 2020,2020:1957843.

[2147] Zhu X P, Han G C, Chen Q, et al. Fatty liver is a sensitive early warning for hypertension and its complication in the Chinese population . Clin Exp Hypertens, 2022:1-7.

[2148] Zhu Y, Shui X, Liang Z, et al. Gut microbiota metabolites as integral mediators in cardiovascular diseases (Review) . Int J Mol Med, 2020,46(3):936-948.

[2149] Zhu Z, Xiong S, Liu D. The Gastrointestinal Tract: an Initial Organ of Metabolic Hypertension? . Cell Physiol Biochem, 2016,38(5):1681-1694.

[2150] Zhurakivska K, Troiano G, Caponio V, et al. Do Changes in Oral Microbiota Correlate With Plasma Nitrite Response? A Systematic Review . Front Physiol, 2019,10:1029.

[2151] Ziętek M, Celewicz Z, Szczuko M. Short-Chain Fatty Acids, Maternal Microbiota and Metabolism in Pregnancy . Nutrients, 2021,13(4).

[2152] Ziff O J, Shapiro A M. Iatrogenic sinistral hypertension complicating screening colonoscopy . Case Rep Surg, 2013,2013:695318.

[2153] Zong-Jie L, Zhen C. Effects of Metabolic Syndrome on Intestinal Flora, Inflammatory Factors, and Infants of Pregnant Patients . Clin Lab, 2020,66(10).

[2154] Zoumpopoulou G, Tzouvanou A, Mavrogonatou E, et al. Probiotic Features of Lactic Acid Bacteria Isolated from a Diverse Pool of Traditional Greek Dairy Products Regarding Specific Strain-Host Interactions . Probiotics Antimicrob Proteins, 2018,10(2):313-322.

[2155] Zubcevic J, Baker A, Martyniuk C J. Transcriptional networks in rodent models support a role for gut-brain communication in neurogenic hypertension: a review of the evidence . Physiol Genomics, 2017,49(7):327-338.

[2156] Zubcevic J, Joe B. Ain't No Sunshine When They're Gone: Rendering the Gut Microbiota "Homeless" by Cecectomy Reveals Their True Thermogenic Potential . Function (Oxf), 2021,2(3):b20.

[2157] Zubcevic J, Richards E M, Yang T, et al. Impaired Autonomic Nervous System-Microbiome Circuit in Hypertension . Circ Res, 2019,125(1):104-116.

[2158] Zununi V S, Barzegari A, Zuluaga M, et al. Myocardial infarction and gut microbiota: An incidental connection . Pharmacol Res, 2018,129:308-317.

[2159] Zuo K, Li J, Li K, et al. Disordered gut microbiota and alterations in metabolic patterns are associated with atrial fibrillation . Gigascience, 2019,8(6).

[2160] Zuo K, Li J, Wang P, et al. Duration of Persistent Atrial Fibrillation Is Associated with Alterations in Human Gut Microbiota and Metabolic Phenotypes . mSystems, 2019,4(6).

[2161] Zuo K, Li J, Xu Q, et al. Dysbiotic gut microbes may contribute to hypertension by limiting vitamin D production . Clin Cardiol, 2019,42(8):710-719.

[2162] Zuo K, Liu X, Wang P, et al. Metagenomic data-mining reveals enrichment of trimethylamine-N-oxide synthesis in gut microbiome in atrial fibrillation patients . BMC Genomics, 2020,21(1):526.

[2163] Zuo K, Yin X, Li K, et al. Different Types of Atrial Fibrillation Share Patterns of Gut Microbiota Dysbiosis . mSphere, 2020,5(2).

[2164] Zwarun A A. Effect of osmotic stabilizers on 14 CO 2 production by bacteria and blood . Appl Microbiol, 1973,25(4):589-591.
